# Selective formation of Pt_12_L_24_ nanospheres by ligand design†

**DOI:** 10.1039/d1sc01295a

**Published:** 2021-04-28

**Authors:** Eduard O. Bobylev, David A. Poole III, Bas de Bruin, Joost N. H. Reek

**Affiliations:** Van ‘t Hoff Institute for Molecular Sciences, University of Amsterdam Science Park 904 1098 XH Amsterdam The Netherlands j.n.h.reek@uva.nl

## Abstract

Supramolecular self-assemblies are used across various fields for different applications including their use as containers for catalysts, drugs and fluorophores. M_12_L_24_ spheres are among the most studied, as they offer plenty of space for functionalization, yielding systems with unique properties in comparison to their single components. Detailed studies on the formation of M_12_L_24_ structures using palladium cornerstones (that have generally dynamic coordination chemistry) aided in the development of synthetic protocols. The more robust platinum-based systems received thus far much less attention. The general use of platinum-based assemblies remains elusive as parameters and design principles of the ligand building blocks are not fully established. As platinum-based nanospheres are more robust due to the kinetically more stable nitrogen–platinum bond, we studied the sphere formation process in detail in order to develop descriptors for the formation of platinum-based nanospheres. In a systematic study, using time-dependent mass spectrometry, ^1^H-NMR and DOSY NMR, we identified new kinetically trapped intermediates during the formation of Pt_12_L_24_ spheres and we developed key parameters for selective formation of Pt_12_L_24_ spheres. Molecular mechanics calculations and experimental result support the importance of charge and steric bulk placed at the *endo*-site of the ditopic linker for selective sphere formation. Applicability of these principles is demonstrated by employing various ditopic ligands with different bend-angles for the synthesis of a range of Pt_2_L_4_, Pt_3_L_6_, Pt_4_L_8_ and Pt_12_L_24_ polyhedra with platinum cornerstones in excellent yields, thus paving the way for future applications of well-defined robust platinum nanospheres of different shapes and sizes with the general composition Pt_*n*_L_2*n*_.

## Abstract 2

We present a systematic study on the formation of Pt_12_L_24_ self-assemblies. By introducing steric bulk or charged moieties at the inside of the nanosphere Pt_12_L_24_ self-assemblies form in excellent selectivity as kinetically trapped intermediates are destabilized.

## Graphical abstract

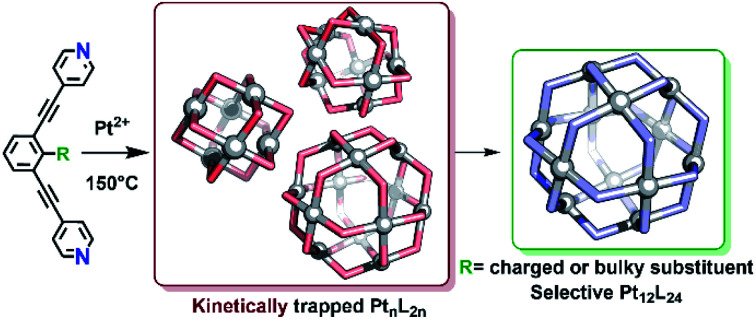

## Introduction

Inspired by the importance of self-assembly processes found in natural systems, supramolecular chemistry has been developed as a strategy to generate functional large molecular objects. In this context, chemists have succeeded in synthesizing a wide variety of polyhedral coordination cages with the general formula M_*n*_L_2*n*_, *n* being 2, 3, 4, 6, 8, 9, 10, 12, 24 and 30.^1–10^ These symmetrical structures are formed from the square-planar complexation of metal ions with ditopic pyridine linkers. Whereas for small structures of the composition M_2_L_4_ various metals have been utilized (*e.g.* M = Pd, Cu, Pt, Zn, Ni),^4,10–12^ only palladium and platinum based structures have been reported for larger assemblies based on ditopic pyridine ligands.^13–15^ Depending on the size of the self-assembled nanospheres, different applications are envisioned. Small M_2_L_4_ assemblies can be used as catalysts^16,17^ or as hosts to bind small guests.^18–20^ Larger structures, such as M_6_L_12_ or M_12_L_24_ assemblies, are able to incorporate bigger guests or even multiple guest molecules.^21–23^ The large inner (*endo*) volume of M_12_L_24_ spheres allows preorganization of up to 24 molecules. As such, it resembles a unique system to study transition metal complexes at high local concentration, which is beneficial when reactions proceed *via* dinuclear pathways or when catalyst and substrates are pre-organized.^21,22,24^ The effect of the second coordination sphere on catalytic reactions,^22^ tandem catalysis^25^ and spectroscopic cooperativity effects can also be studied.^26–29^

**Fig. 1:**
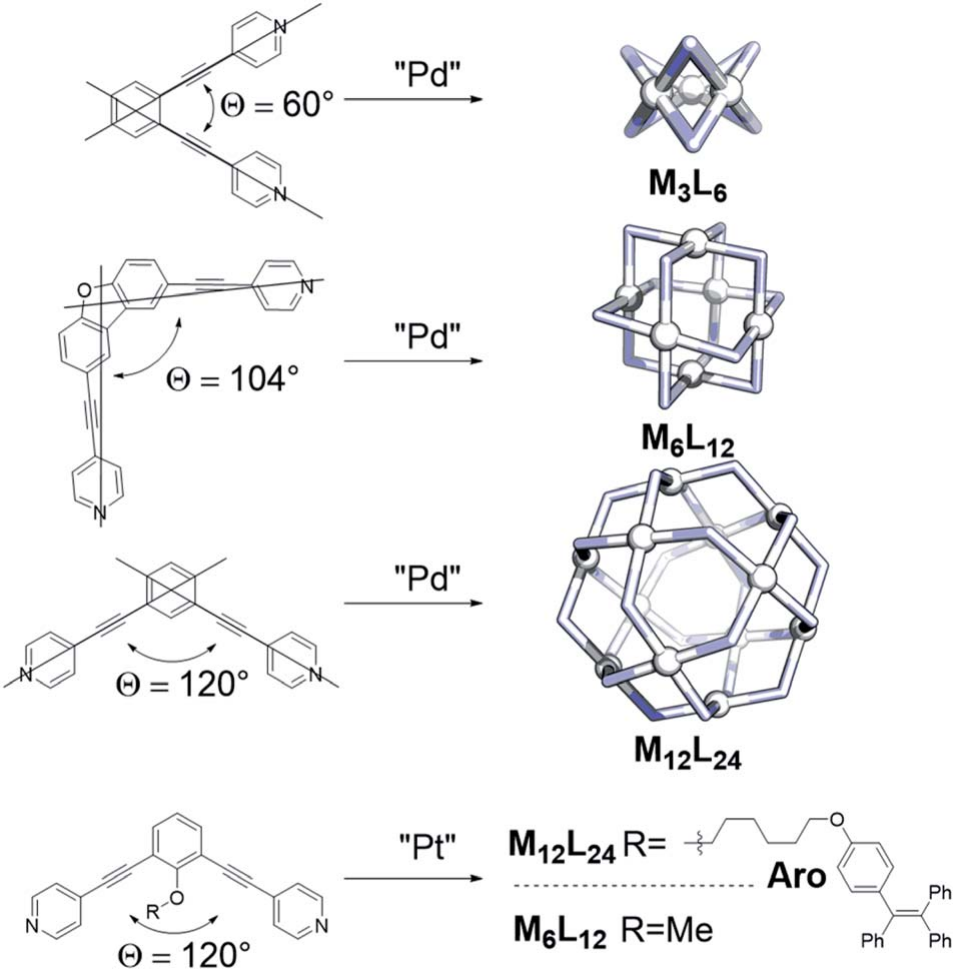
Selected examples demonstrating the bend angle approach for palladium-spheres; two literature known building blocks which show that the bend angle is not sufficient to predict formation of platinum spheres.

For several applications it is important to control the size of the sphere that is formed during the assembly process, but this can be challenging. Key parameters for selective palladium-based systems have been identified previously by the group of Fujita. One main design principle is the dihedral angle (bend angle) between the two pyridines of the applied linker (Fig. 1).^2^ This bend angle is locked at a certain degree by employing a rigid spacer between the central aromatic system and the pyridines (Fig. 1). The outcome of sphere formation is well-described by the bend angle strategy because of geometric constraints, with only few exceptions.^1,30^ Other parameters such as solvent, concentration and counterion of the palladium source are typically adjusted to the system requirements and generally do not play a major role in the selectivity of sphere formation.^15^

The dynamic pyridyl–palladium bond leads to dynamic cage behaviour, which make palladium-based spheres excellent candidates for studying cooperativity effects and structural features. However, palladium-spheres are not sufficiently stable for all applications.^23,24,31^ The analogous platinum systems display superior stability under a variety of conditions including the presence of coordinating substrates, due to the stronger and kinetically more stable Pt–N(pyridine) bonds.^24^ Moreover, platinum-spheres are less prone to degradation by reducing agents^23^ and display good fluorescent properties,^26–29^ making them promising candidates for biological applications.^32,33^ Research has been focused mainly on the formation of palladium-based systems^34–37^ and their application across the fields.^3,9,21,22,25,30,38–42^ Only a relatively small number of reports describe platinum-based M_*n*_L_2*n*_ analogues.^22–24,29,31,32,43,44^

The strong bond between Pt and pyridine allows limited conditions for product formation as sufficient dynamicity is required. Two examples, from the group of Fujita and the group of Stang, are depicted in Fig. 1 using a combination of high temperature and agents that destabilize the Pt–N bond.^29,31^ Interestingly, even though most structural features of the linkers such as the bend angle and electronic properties as well as similar synthetic protocols were employed, one of the ditopic ligands formed a Pt_6_L_12_ (R = Me) sphere^31^ and the other a Pt_12_L_24_ (R = **Aro**, Fig. 1) sphere.^29^ These examples demonstrate that the bend angle as practical parameter for the prediction of palladium based nanospheres is a poor or incomplete descriptor for platinum nanosphere formation. We therefore decided to investigate the factors determining platinum nanosphere formation in detail to guide their synthesis. The results are reported in this paper. In order to obtain a clear picture, the formation nanospheres was carefully monitored using MS-analysis and NMR techniques providing insights into sphere formation pathways. The metastable structures formed along the pathway to the formation of M_12_L_24_ assemblies were next used as a starting point to develop design principles for platinum spheres (combined with known principles guiding palladium sphere formation). With these descriptors, the outcome of sphere formations with platinum cornerstones can be predicted and ligands can be designed to yield desired structures in various shapes and sizes, as was confirmed in this study. Keeping in mind the numerous advantages of platinum-based systems, this work should stimulate further development of these more robust architectures in different applications.

## Results and discussion

### Model studies

**Fig. 2:**
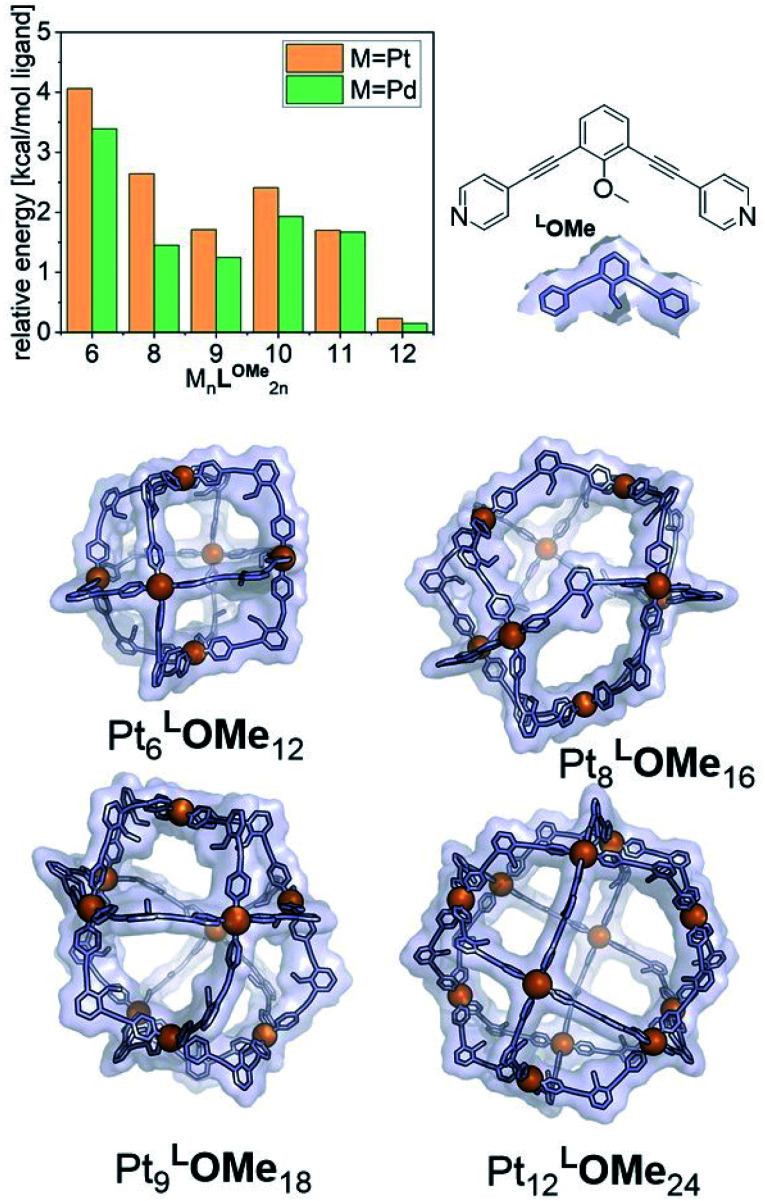
Examples of minimized structures for Pt_*n*_**LOMe**_2*n*_ spheres and the per-ligand energy of differently sized spheres relative to the minimum energy structure, cuboctohedral Pt_12_**LOMe**_24_. These energies include the Boltzmann weighted contributions of less favourable configurations such as the triangular bicopula form of the Pt_12_**LOMe**_24_.

The goal of our investigation was to selectively form Pt_12_L_24_ assemblies with broadly functionalizable building blocks. A relatively simple building block with acetylene linkers **LOMe** (Fig. 2) was chosen for initial studies. This building block has a bend angle of 120° geometrically required for M_12_L_24_ assemblies.^2,36^ Furthermore, it has no particular electronic or steric demands and can act as a starting point for *exo* or *endo* functionalization. Because spherical systems are typically prepared by thermodynamic control, molecular modelling was performed on the desired Pt_12_**LOMe**_24_ assembly and other possible spherical structures. These possible intermediate structures for platinum based M_12_L_24_ systems are based on observations made for the analogues palladium spheres. Five possible intermediates Pd_6_L_12_, Pd_8_L_16_, Pd_9_L_18_, Pd_10_L_20_ and Pd_11_L_22_, were predicted *in silico*.^36^ Two of these palladium-based structures (Pd_8_L_16_, Pd_9_L_18_) were trapped by ligand design and characterized in detail.^35^ We included all known and predicted intermediates in our molecular modelling. According to the minimized structures for all spherical objects of the general formula Pt_*n*_L_2*n*_ for *n* = 6–12 (Fig. 2, SI7† for details, description of the method can be found in ref. 37), the Pt_12_**LOMe**_24_ sphere represents the thermodynamic minimum. Because the Pt_12_**LOMe**_24_ assembly is thermodynamically even more favourable over other structures than was the case for the palladium analogue (Fig. 2), selective formation was expected when structures could form under thermodynamic control.

**Fig. 3:**
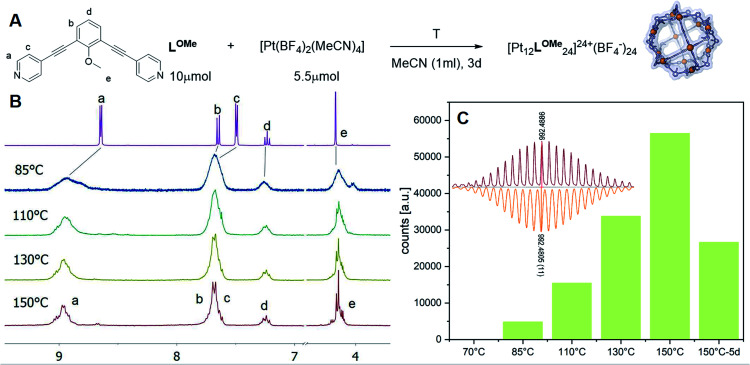
(A) Reaction scheme of the formation of [Pt_12_**LOMe**_24_]; (B) ^1^H-NMR spectra of the complexation of ligand **LOMe** at different temperatures; (C) Development of the assigned 11 + species of the [Pt_12_**LOMe**_24_] assembly at different temperatures (inset: measured spectra = red; calculated spectra = orange).

**Fig. 4:**
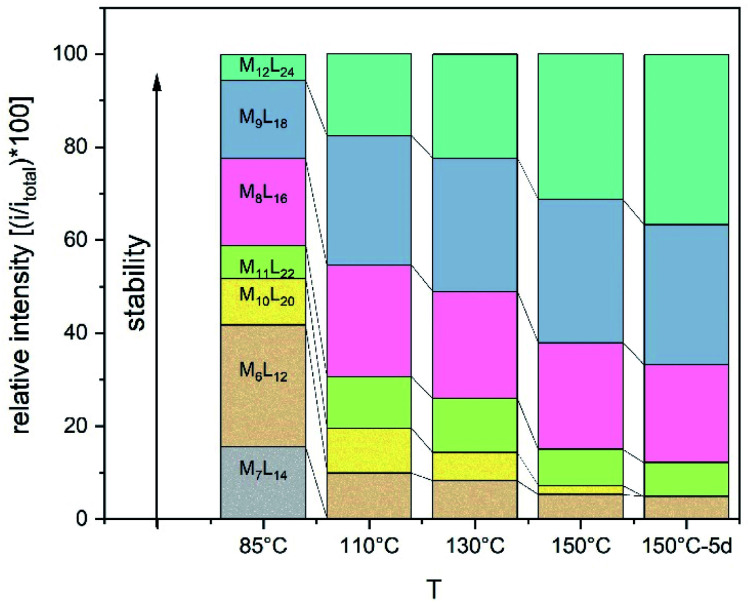
Development of unique signals assigned to each of Pt_*n*_**LOMe**_2*n*_ assemblies for *n* = 6–12 at different temperatures.

Experiments were set up to form the Pt_12_L_24_ assembly with **LOMe** under thermodynamic control by varying the reaction temperature. Sphere synthesis was performed by mixing 1 eq. of building block **LOMe** with 0.55 eq. [Pt(BF_4_)_2_(MeCN)_4_] in acetonitrile in closed high pressure tubes for 3 days at the desired temperature (Fig. 3A). Progress was monitored using ^1^H-NMR, DOSY and MS-analysis. At 70 °C, a downfield shift of the pyridine protons accompanied by broadening of the signals upon coordination to platinum was observed. The surprisingly broad signals are in contrast to the sharp signals obtained for the well-defined palladium-based sphere. A lower diffusion coefficient observed by DOSY indicates formation of structures in the size range of the M_12_L_24_ spheres (S65–S67†). However, no well-defined sphere structures were detected by MS analysis. The kinetically inert Pt–N bond may prevent formation of the thermodynamically favoured spheres due to kinetic trapping of less stable intermediates. Therefore, we increased the reaction temperature gradually. At 85 °C, the ^1^H-NMR and DOSY spectra remained similar to the spectra obtained at 70 °C (Fig. 3B, S74–S75†). MS analysis showed multiple signals corresponding to different charged states of the sphere with isotopic patterns corresponding to [Pt_12_(**LOMe**)_24_(BF_4_^−^)^24−*x*^]^*x*+^ (*x* = 7–11). Because these self-assembled spheres show a linear response with the concentration in the MS spectra^15^ (S113–S115†), provided that the parameters such as the ion strength and the solvent remain the same, the quantity of the desired sphere in solution can be monitored by the characteristic peaks of the M_12_L_24_ sphere, *i.e.* [Pt_12_(**LOMe**)_24_(BF_4_^−^)_13_]^11+^ (*m*/*z* = 992 Da) (Fig. 3C). Increasing the reaction temperature further to 110 °C, 130 °C and 150 °C led to relatively sharp signals in the ^1^H-NMR spectra (Fig. 3B). All signals are split into multiple sets, indicative for the presence of multiple species. The intensity of the identified 11^+^ species increased with increasing the reaction temperature (Fig. 3C, S69†). However, after prolonged heating at 150 °C, the signal became less intense (after 5 days). The decrease of counts is accompanied with a colour change of the solution (from colourless to orange), observation of new species in ^1^H-NMR (around 5 ppm, S73†) and appearance of free building block **LOMe**. Presumably the sphere disassembles by decomposition of both the platinum precursor to nanoparticles and the ligand **LOMe** to other adducts (S73†). Besides the desired Pt_12_(**LOMe**)_24_ also other structures can exist in solution as indicated by the multiple sets of signals in the ^1^H-NMR spectra. A detailed analysis of the MS data confirmed the presence of multiple spherical objects. Besides analogues to intermediates which have been identified/predicted for palladium as Pt_8_**LOMe**_16_, Pt_9_**LOMe**_18_, Pt_10_**LOMe**_20_ and Pt_11_**LOMe**_22_, also a non-Archimedean-solid type Pt_7_**LOMe**_14_ was observed. MSMS analysis of the M_12_L_24_ assembly show that these structures were not formed by fragmentation during the MS experiments (S116†). As such, it is important to analyse all multiple charged species, as otherwise some of the formed assemblies could be missed. Analysis of the even charged species may be complicated, because they can show an overlap of differently charged states of different assemblies (*e.g.*, overlap of [Pt_12_(**LOMe**)_24_(BF_4_^−^)_12_]^12+^ and [Pt_8_(**LOMe**)_16_(BF_4_^−^)_8_]^8+^). All structures indicated here show signals corresponding to [Pt_n_(**LOMe**)_2n_(BF_4_^−^)_2*n*−*x*_]^*x*+^ for multiple charged states with a matching isotope pattern (S34†). Calibration of the MS signals using of a mixture of nanospheres prepared at 150 °C for 3d (which gave overall the most counts, Table S19†) and measured at a range of concentrations (diluted with a Pd_12_**LOMe**_24_ sphere to maintain the ion concentration constant) show a linear correlation between the concentration of the different sized spheres and their intensity in the MS spectra (S113–S115†). With this correlation in hand, the relative amount of spheres formed with different methods can be compared. By following unique signals assigned to each of those assemblies over a range of different temperature, their relative stability was estimated (Fig. 4). Though this method is prone to quantitative errors (∼10% as described in S70–S71† from experiments in triplo), qualitative analysis showed excellent reproducibility (S70–S71†). At 85 °C, the dominant (highest intensity) species was assigned to Pt_6_**LOMe**_12_, similar to what has been found before in literature.^31^ After heating the sample for 3d at 110 °C the Pt_7_**LOMe**_14_ assembly disappears (least stable) and the M_6_L_12_ structure becomes less pronounced (moderately stable). Heating the sample at 150 °C led to disappearance of the M_10_L_20_ assembly. Spheres with 8, 9 and 12 platinum cornerstones increased constantly over the period of the study. The slope of increase was most dominant for the M_12_L_24_ assembly (most stable). After heating the sample for prolonged time at 150 °C or addition of 2-chloropyridine as a destabilizing agent (S72†), the M_8_L_16_ structure decreased more than the M_9_L_18_ assembly (more stable). The experimentally obtained relative stability is in good agreement to our computational results (see Fig. 4 and 2).

Because pure spheres could not be obtained, we attempted to purify the sphere from the mixtures by crystallization and column chromatography. Crystallization did not turn out fruitful using a number of different anti solvents (Et_2_O, THF, diisopropylether, chloroform). Interestingly, a mixture of nanospheres prepared at 150 °C, could be separated by column chromatography. Eluting with pure MeOH, using silica as the stationary phase afforded pure Pt_12_**LOMe**_24_ assembly according to MS analysis in small quantities (<0.5 mg, <1% yield; S119†). While optimizing the separation conditions (such as stationary phase, solvent, *etc.*) is not within the scope of this work, the experiment shows that the kinetic stability of platinum spheres sufficiently high for purification by column chromatography.

The formation of an Pt_12_L_24_ assembly with a simple acetylene bridged ligand has been achieved by adjusting the temperature. However, despite using destabilizing agents and elevated temperature, selective formation of the thermodynamically favoured Pt_12_L_24_ assembly was not possible using **LOMe**. Two initially kinetically trapped assemblies Pt_7_L_14_ and Pt_10_L_20_ disappeared by increasing the reaction temperature. Other spheres such as the Pt_9_L_18_ and Pt_8_L_16_ remained in solution regardless of the temperature applied. The relative energy gap between the M_9_L_18_, M_8_L_16_ and the M_12_L_24_ assembly is similar for both, platinum-based systems and the palladium-based analogues. It is possible to form Pd_12_L_24_ self-assemblies selectively with the relatively small thermodynamic gap between the desired Pd_12_L_24_ and the smaller sized structures (Pd_*n*_L_2*n*_ for *n* = 6–11). This shows that the thermodynamic difference (which is even bigger for Pt) should also allow the selective formation of Pt_12_L_24_ assemblies. Because the desired Pt_12_L_24_ assembly could not be formed by adjusting the temperature, intermediates of the general formula Pt_*n*_L_2*n*_ for *n* = 8, 9, 11 are kinetically trapped due to the stronger Pt–N bond, as they do not disappear upon increasing the reaction temperature (Fig. 4). In contrast to these observations made for platinum, palladium-based systems have a weaker Pd–N bond and can thus escape from a kinetic trapped mixture of intermediates, thus forming the thermodynamically most favoured Pd_12_L_24_ assembly selectively.

### Formation pathway and strategy

In order to develop a strategy to selectively form M_12_L_24_ assemblies, the formation pathway was studied further. The proposed pathway of palladium-based systems proceeds *via* an initially formed coordination oligomer and subsequently through a set of kinetically trapped small sized intermediates of the general formula M_*n*_L_2*n*_ (*n* = 6, 8 and 9). These Pd intermediates were found relatively stable by computation, but could only be analysed as short-lived intermediates with ^1^H-NMR spectroscopy and HR ESI-MS studies.^34^ It is not clear whether the thermodynamically controlled Pd_12_L_24_ assemblies form by a linear pathway from small sized spheres and intermediates or alternatively that the intermediates re-enter an oligomeric state and reform spheres.

**Fig. 5:**
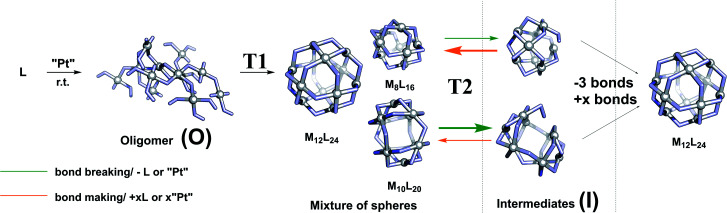
Proposed formation mechanism of platinum-based spheres (*T*1 = 85 °C; *T*2 = 150 °C); middle: example of a few spheres formed during this process (M_8_L_16_, M_12_L_24_ and M_10_L_20_); arrows indicate formation and deformation of most unstable spheres; productive pathways are depicted with bold arrows.

We observed after mixing of **LOMe** with the corresponding platinum salt at 70 °C (or lower) no higher charged species in the HR CSI-MS spectra, DOSY shows a low diffusion coefficient (S74–S77†) and broad signals were observed in the ^1^H-NMR spectra. The species formed in the initial period is likely oligomeric in nature (O) (Fig. 5). When this oligomeric material (O) is heated to 85 °C (or higher), a sharper ^1^H-NMR spectrum appears and also the HR CSI-MS spectra display signals attributed to various types of spheres with the general formula Pt_*n*_L_2*n*_. In a cage formation experiment carried out at 85 °C similar amounts of different spherical objects (*e.g.*, Pt_9_L_18_ and Pt_6_L_12_) are formed (Fig. 4). This implies that the initial oligomeric material (O) can fold in different ways forming randomly different sized spheres. Increasing the temperature to 150 °C causes the disappearance of the Pt_7_L_14_ and a decrease of the signals attributed to the Pt_6_L_12_ and Pt_10_L_20_ assemblies. These different Pt_*n*_L_2*n*_ assemblies thus represent kinetically unstable structures. Their decrease is accompanied with an increase of concentrations of the larger assemblies Pt_8_L_16_, Pt_9_L_18_ and Pt_12_L_24_ assemblies. This transition to the bigger assemblies can proceed *via* two slightly different pathways. (1) the initially formed intermediates (I) are further converted to the larger spheres. Herein, we combine all non- or over-saturated spherical structures which are derived from Pt_*n*_L_2*n*_ to these intermediates (I) (*e.g.*, Pt_*n*+1_L_2*n*_, Pt_*n*_L_2*n*+1_, Pt_*n*_L_2*n*−1_ and Pt_*n*_L_2*n*_MeCN_1_, Fig. 5). (2) the initial oligomer (O) can be re-formed from the intermediates, from which all types of spheres are formed again. In this scenario the intermediates and cages are in equilibrium *via* the oligomer state.

To arrive at a strategy that leads to selective formation of Pt_12_L_24_ assemblies, it is important to know how the observed small sized assemblies convert to the desired structure. The mechanism of conversion from intermediates to desired structure (route 1 or 2) has implications for the design rules of building blocks and conditions for the preparation of pure Pt_12_L_24_ assemblies. As such, additional exchange experiments under various conditions were carried out and analysed by NMR and HR CSI-MS. In these experiments we generated self-assembled structures based on slightly different building blocks **LOMe** and **LOBn** and we mixed the solutions at various stages of sphere formation and monitored the degree of exchange, resulting Pt_*n*_**LOMe**_2*n*−*x*_**LOBn**_*x*_ assemblies.

**Fig. 6:**
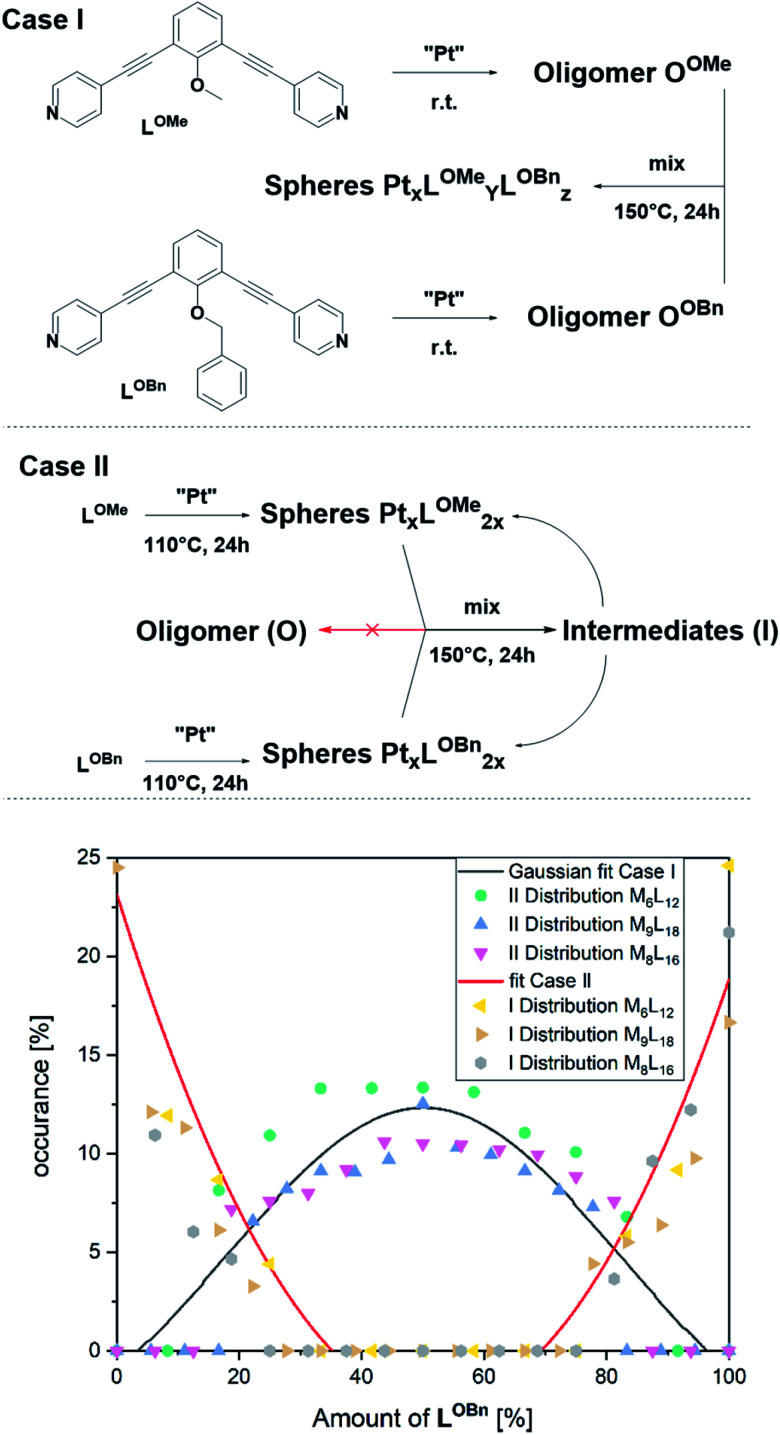
(Case I) Formation of two individual oligomers and the resulting statistical distribution of the ligands in the spherical objects obtained after heating a mix of oligomers; (Case II) Formation of a small number of individual spheres and the resulting non-statistical distribution of the ligands in the spherical objects obtained after heating; MS Distribution of the two ligands obtained by averaging the relative intensities of the respective M_6_L_12_, M_8_L_16_ and M_9_L_18_ assemblies (Case I gives a Gaussian distribution of the two applied ligands; Case II was fitted quadratically to give a line to guide the eye).

In a first experiment (Fig. 6, Case I), two oligomers O^OMe^ and O^OBn^ containing either **LOMe** or **LOBn** are formed in separate solutions by mixing the ligands with platinum precursor at room temperature. Coordination of platinum is confirmed by a downfield shift of the pyridine protons and DOSY shows that the formed structure has a lower diffusion coefficient in line with oligomer formation (S79–S81†). MS analysis of the individual oligomer mixtures showed no occurrence of spherical structures as multiple charged species were absent. The oligomers solutions were mixed in a 1 to 1 ratio and heated at 150 °C for one day. Analysis of the solution revealed a mixture of different sized spheres with a statistical distribution of the two ligands **LOMe** and **LOBn** as expected from building blocks with no self-sorting function (Fig. 6, S88–S91†). This experiment shows that building block exchange from the oligomer state occurs without kinetic barriers.

In the second experiment, the spheres of **LOMe** and **LOBn** are prepared in separate solutions at 110 °C for one day (Fig. 6, Case II), combined and further heated at 110 °C. Importantly, no changes are observed by MS analysis (S94†) indicating that building blocks do not exchange when the assemblies are in well-defined sphere states, in line with kinetic inertness of platinum assemblies up to 110 °C. However, if the mixture is heated at 150 °C more spherical structures (S95†) are formed showing building block exchange under these conditions. The formation of more spherical compounds at 150 °C is accompanied with slow exchange of single ligands (Fig. 6, S96–S99†). The exchange of single ligands is occurring for all type of assemblies (M_*n*_L_2*n*_ for *n* = 6–12) in a similar fashion. As the first experiment shows that in the oligomer state there is a full exchange of building blocks, and the second experiments shows only slow exchange of single building blocks from the spheres (similar result was also obtained mixing spherical sample of **LOMe** with oligomeric sample of **LOBn**, S102†), we can conclude that formation of platinum-based spheres from the oligomer state is irreversible. Indeed, such transition from sphere to oligomer requires the breaking of multiple Pt–pyridyl bonds.

At 150 °C, the exchange of single ligands is kinetically allowed. Importantly, this implies that formation of pure Pt_12_L_24_ spheres requires the direct conversion of formed intermediates to the desired Pt_12_L_24_.

As the conversion of lower to higher spheres is crucial, we studied it in more detail. Analysis of the structures shows that it is required to break (at least) 4 Pt–N bonds to obtain the Pt_12_L_24_ sphere from any of the smaller intermediates (section SI6†). The intermediates Pt_8_L_16_ and Pt_9_L_18_ spheres are kinetically inert as they remain in solution even at elevated temperatures. In the formation of Pt_12_L_24_ assemblies these therefore represent trapped states. Experiments show that at 150 °C exchange of single ligands from both the Pt_8_L_16_ and Pt_9_L_18_ spheres is possible, however, the conversion of these M_8_L_16_ and M_9_L_18_ sphere intermediates to the desired Pt_12_L_24_ is not possible (*e.g.*, Fig. 5 and section SI6†). As the palladium–pyridyl bond is weaker, these M_8_L_16_ and M_9_L_18_ intermediates do not represent trapped states for the formation of Pd_12_L_24_ spheres.

### Ligand design by using ditopic ligands with functional groups

**Fig. 7:**
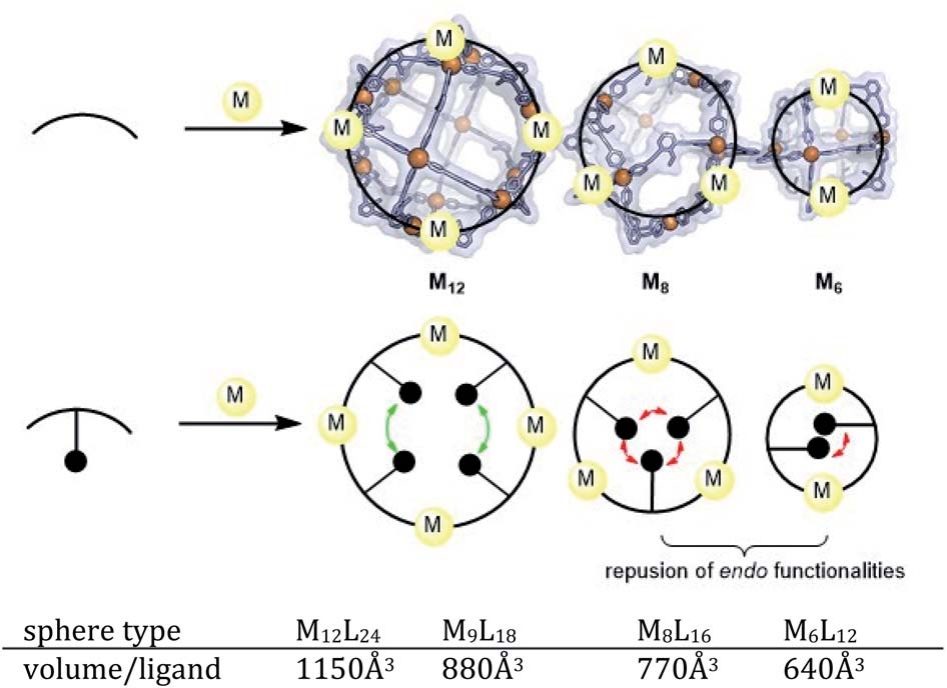
Schematic representation of our strategy for destabilization of small size spheres. Representation of M_6_L_12_, M_8_L_16_ and M_12_L_24_ nanospheres. The sphere frames are optimized at molecular mechanics level (MMFF) and shown in space-filling-style.

As some of the intermediates are kinetically trapped states we set-out a strategy for selective formation of Pt_12_L_24_ assemblies that relies on destabilization of these intermediate-sized structures. A look at the modelled spheres shows a clear growth of the interior space available per ligand with increasing sphere size (Fig. 7). For example, the volume available for the M_12_L_24_ sphere is 25% larger than that of the M_9_L_18_ intermediate state. By *endo* functionalization of the ditopic ligand building blocks, either with sterically bulk or charged functional groups, the smaller spheres are destabilized by steric hindrance (or charge repulsion) to a larger extend than the M_12_L_24_ spheres. These intermediates therefore might not form, or are destabilized such that they no longer form kinetic traps.

**Fig. 8:**
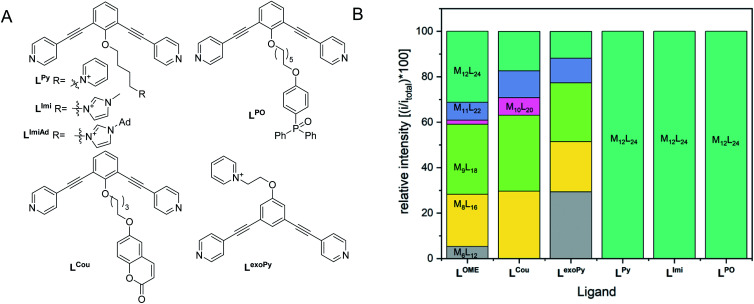
(Left) Structures of applied ligands in our investigation for selective formation of Pt_12_L_24_ spheres by steric or electrostatic repulsion; (Right) distribution of spherical structures obtained after complexation of the applied ligands with platinum precursor.

A set of ditopic ligands with additional functional groups was synthesized to test our hypothesis of selective formation of Pt_12_L_24_ spheres by a combination of electrostatic/steric repulsion, using the typically bend angle of 120°. The set (Fig. 8) contains two building blocks with charged units at the *endo* site (**LPy** and **LImi**), and building block with a charged unit at the *exo* site **LexoPy** to carefully study the effect of electrostatic repulsion. Two ligand building blocks, **LPO** and **LCou**, with bulky functional groups at the *endo* site were also used. All novel ligands were synthesized by a modular approach (section SI1†).

First, two sets of spherical structures (M_6_–M_12_) were modelled based on charged ligand **LPy** and the bulky ligand **LPO**. In agreement with our previous observations, also for these assemblies the M_12_L_24_ sphere represents the thermodynamic minimum. Assembly formation with **LPO** was performed with 0.55 eq. platinum precursor. After heating the sample for 2d at 150 °C, a single set of sharp signals was observed in the ^1^H-NMR spectra (S43–S46†). HR CSI-MS analysis of the sphere solution showed selective formation of the desired Pt_12_**LPO**_24_ structure (Fig. 8B, S47†). Signals of any other sphere (M_*n*_L_2*n*_) were absent in the MS spectrum. These experiments show that indeed the presence of steric groups at the endo site prevents the formation of kinetically trapped states. For the building block **LAro**, also discussed in the introduction (Fig. 1) the formation of the Pt_12_L_24_ species was reported, suggesting that also for this sphere the selective sphere formation is possible because of the presence of the bulky functional group present at the *endo* site. The coumarin functionalized building block **LCou**, which is sterically less demanding, was also used for the formation of spheres under identical conditions, and this yielded a mixture of spheres (Fig. 8, S39–S42†). This shows that the introduction of steric groups is a successful strategy to obtain Pt_12_L_24_ spheres in a selective fashion, provided that the groups introduced at the *endo* side are sufficiently large. As a small note, the presence of 24 units of **LPO** occupy the complete interior space of the Pt_12_L_24_ sphere, complicating applications in which space is required, for example the binding of guest molecules or utilize the system in catalytic transformations.

**Fig. 9:**
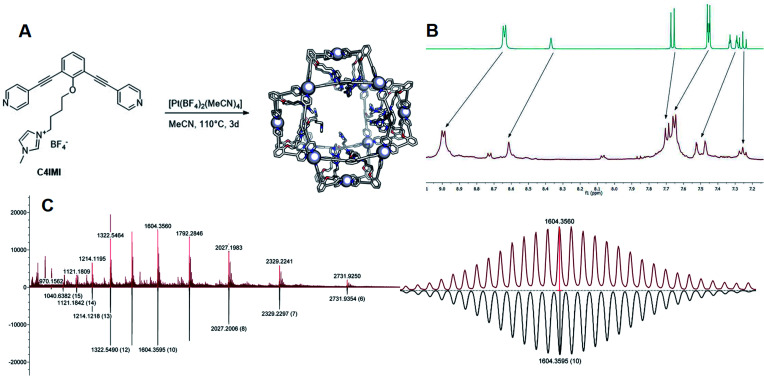
(A) Synthesis of the [Pt_12_(**LImi**)_24_] assembly, the sphere frames are optimized at molecular mechanics level (MMFF), nitrogen = blue, oxygen = red, carbon = white and the metal is depicted as white sphere; (B) ^1^H-NMR spectra of the ligand (top) and the self-assembly (bottom); (C) MS data of the self-assembly recorded at 60 °C in MeCN (top: measured; bottom: calculated) and zoom in into the 10+ species.

As a second strategy, we attempted to selectively form Pt_12_L_24_ spheres using charge repulsion. After heating a sample containing **LImi** and 0.55 eq. platinum precursor to 150 °C for 2d, only the desired Pt_12_L_24_ sphere was formed as shown by a single set of protons in ^1^H-NMR and HR CSI-MS analysis (Fig. 9 and 8). When sphere formation was attempted at 85 °C a small amount of the corresponding Pt_8_**LImi**_16_ and Pt_9_**LImi**_18_ assemblies were detected using MS analysis (S78†). These structures disappear upon heating, indicating an escape from the otherwise kinetically trapped assemblies (S52†). In contrast to this selective formation of a Pt_12_**LImi**_24_ assembly, utilizing the *exo* functionalized building block **LexoPy**, yielded a mixture of spheres when the same conditions for sphere formation were applied (Fig. 8, see S58–S60† for details). This experiment demonstrates the importance of *endo* functionalization for the successful selective sphere formation with platinum cornerstones, as *exo* functionalization does not take advantage of differences in the available inner-volume between the kinetically trapped smaller assemblies and the desired larger Pt_12_L_24_ spheres.

Having demonstrated that building blocks with the proper bend angle and with sufficient steric bulk or charges at the *endo* site of the ligand leads to selective formation of Pt_12_L_24_ assemblies at 150 °C, we decided to perform additional experiments (Fig. 8B). The general applicability with different positively charged groups was confirmed using a ligand bearing a different functional group **LPy** (Fig. 8, details S53–S57†). Mixing **LPy** with the platinum pre-cursor for 2d at 150 °C selectively yields the Pt_12_L_24_ sphere in pure form, as shown by MS analysis (S57†). Further-more, the ^1^H-NMR spectrum of the formed complex is well resolved with a downfield shift of the pyridine protons (S53–S56†). From our previous experiments using a similarly sized ligand **LCou**, the steric repulsion introduced by a single aromatic group is not sufficient for selective formation (Fig. 8). Because selective sphere formation using charge repulsion does not require bulky substituents, these building blocks render themselves suitable for further functionalization (available space in self-assembly, Fig. 9). This was previously demonstrated by the binding of 24 gold complexes in the guanidine based nanosphere.^22^ In addition, both, the imidazolium-based building block **LImi** as well as **LPy** can be further derivatized with functional groups of interest for guest uptake or catalytic investigations within well-defined Pt_12_L_24_ assemblies. Exemplary, an adamantane group was placed on the imidazolium linker (**LImiAd**) (Fig. 8), which yields together with platinum also Pt_12_**LImiAd**_24_ self-assemblies with good selectivity (S61–S64†), also indicating that there is sufficient space available for further chemistry.

### Application for other structures

**Fig. 10:**
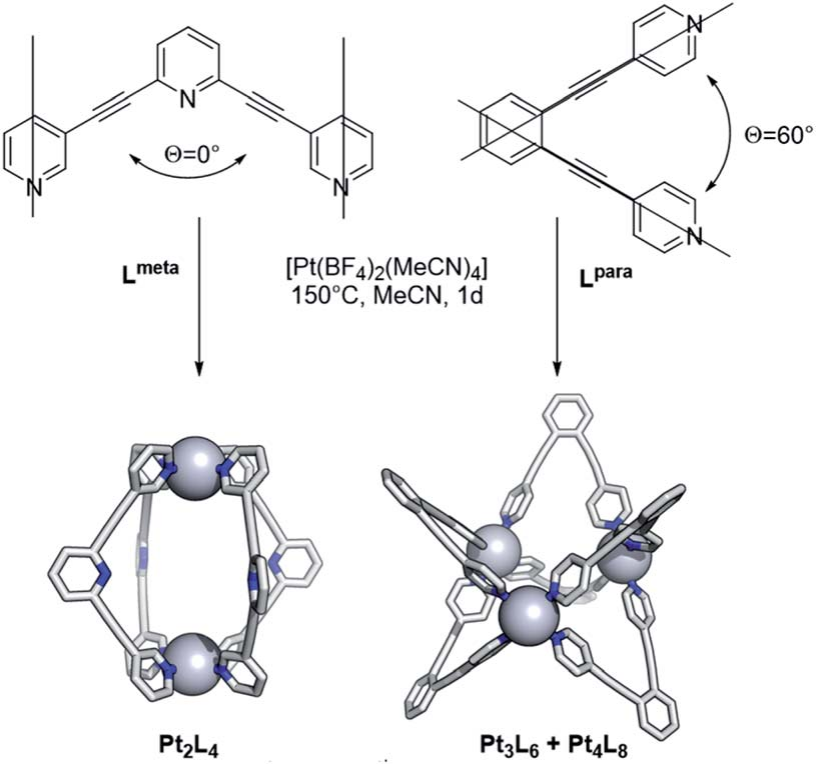
Complexation of **Lmeta** and **Lpara** at high temperature (*meta* and *para* refer to the connection of the pyridine ring with respect to the nitrogen atom).

After exploring the formation of Pt_12_L_24_ spheres, the methodology was expanded to other systems. As described before, the bend angle between the pyridine bonds allows selective formation of palladium based spherical objects of different size. We choose building blocks known for successful sphere formation of the palladium analogue with bend-angles of *Θ* = 0° and 60° (**Lmeta** and **Lpara**, Fig. 10).^1,13^ Because platinum spheres form through smaller size kinetically trapped structures at elevated temperatures, these two bend angles are ideal to check sphere formation as both form the smallest possible spherical assemblies, being the M_2_L_4_, M_3_L_6_ and M_4_L_8_ structures. Sphere formation was performed by mixing 1 eq. of building block **Lmeta** or **Lpara** with 0.55 eq. [Pt(BF_4_)_2_(MeCN)_4_] in acetonitrile at 150 °C for 24 h (Fig. 10, section SI5†). After this period sharp ^1^H-NMR spectra with a downfield shift of all pyridine protons upon coordination of platinum were observed. Furthermore, HR CSI-MS analysis and DOSY NMR supported the formation of the desired assemblies. In line with the experiments for Pt_12_L_24_ sphere formation, when reaction temperatures were applied up to 110 °C a mixture of assemblies was obtained (Fig. S106†), suggesting that also for these smaller spheres the presence of kinetically trapped states may prevent the formation of pure assemblies. The elevated temperature of 150 °C allowed formation of the small spherical objects in excellent yields (94% based on ^1^H-NMR, see section SI5†).

## Conclusions

Whereas it has previously been demonstrated that using the bend angle of ditopic pyridyl building blocks is an effective descriptor to predict the formation of the size of Pd_*n*_L_2*n*_, this descriptor alone is not sufficient for platinum analogues. A systematic study on the selective formation of platinum-based spheres using a combination of experimental and theoretical approaches is described. Due to the stronger and less dynamic platinum–nitrogen bond (compared to palladium–nitrogen), a high temperature is required to thermodynamically drive the equilibrium towards the most stable assemblies. Mixing ditopic ligands with the platinum precursor at 70 °C leads to mostly oligomer formation. Upon increasing the reaction temperature during the sphere formation, mixtures of various spheres are formed. Upon raising the temperature, some of the kinetically trapped intermediate Pt_*n*_L_2*n*_ species disappear from the reaction mixture, but a mixture of Pt_*n*_L_2*n*_ assemblies is still observed with simple (unfunctionalized) ligands. Sphere decomposition is observed before the thermodynamic Pt_12_L_24_ species is formed as the only nanosphere. Introducing the proper amount of steric bulk or an endohedral charge on the ligand gives rise to selective formation of well-defined Pt_12_L_24_ assemblies in excellent yield. These groups destabilize the smaller spheres that are the kinetic traps, such as the Pt_8_L_16_ and Pt_9_L_18_ and therefore an escape from these trapped states is possible. The herein presented charged building blocks can be easily modified to yield a variety of robust Pt_12_L_24_ assemblies in excellent yields. The synthetic strategy has been extended to other building blocks, including those with other bend angles that lead to other structures (Pt_2_L_4_–Pt_4_L_6_). MS was demonstrated to be an ideal tool to study complex sphere mixtures, helping to identify kinetically trapped intermediates pathing the way for strategies to escape those.

Within the field of supramolecular self-assemblies, features of platinum-based assemblies such as electronic and kinetic inertness, fluorescent properties and biological relevance are already well recognized. By providing a strategy for selective formation of Pt_*n*_L_2*n*_ assemblies with easily modifiable ligands, we hope to promote further research on platinum based self-assemblies and their applications across fields.

## Author contributions

Conceptualization: EOB, JNHR; formal analysis: EOB, DAP; funding acquisition: JNHR, BdB; investigation: EOB, DAP; supervision: JNHR; validation: EOB, DAP, BdB, JNHR; visualization: EOB, DAP; writing – original draft: EOB; writing – review & editing: EOB, DAP, BdB, JNHR.

## Conflicts of interest

There are no conflicts to declare.

## Supplementary Material

SC-012-D1SC01295A-s001

## References

[cit1] Chand D. K., Biradha K., Kawano M., Sakamoto S., Yamaguchi K., Fujita M. (2006). Chem.–Asian J..

[cit2] Fujita D., Ueda Y., Sato S., Yokoyama H., Mizuno N., Kumasaka T., Fujita M. (2016). Chem.

[cit3] Harris K., Sun Q. F., Sato S., Fujita M. (2013). J. Am. Chem. Soc..

[cit4] Kishi N., Li Z., Yoza K., Akita M., Yoshizawa M. (2011). J. Am. Chem. Soc..

[cit5] Liao P., Langloss B. W., Johnson A. M., Knudsen E. R., Tham F. S., Julian R. R., Hooley R. J. (2010). Chem. Commun..

[cit6] Mesquita L. M., Anhauser J., Bellaire D., Becker S., Lutzen A., Kubik S. (2019). Org. Lett..

[cit7] Suzuki K., Tominaga M., Kawano M., Fujita M. (2009). Chem. Commun..

[cit8] Sun Q. F., Iwasa J., Ogawa D., Ishido Y., Sato S., Ozeki T., Sei Y., Yamaguchi K., Fujita M. (2010). Science.

[cit9] Sato S., Ishido Y., Fujita M. (2009). J. Am. Chem. Soc..

[cit10] Tsutsui T., Catti L., Yoza K., Yoshizawa M. (2020). Chem. Sci..

[cit11] Li Z., Kishi N., Hasegawa K., Akita M., Yoshizawa M. (2011). Chem. Commun..

[cit12] Li Z., Kishi N., Yoza K., Akita M., Yoshizawa M. (2012). Chem.–Eur. J..

[cit13] Han M., Engelhard D. M., Clever G. H. (2014). Chem. Soc. Rev..

[cit14] Chakrabarty R., Mukherjee P. S., Stang P. J. (2011). Chem. Rev..

[cit15] Yoshizawa M., Klosterman J. K., Fujita M. (2009). Angew. Chem., Int. Ed..

[cit16] Marti-Centelles V., Lawrence A. L., Lusby P. J. (2018). J. Am. Chem. Soc..

[cit17] Spicer R. L., Stergiou A. D., Young T. A., Duarte F., Symes M. D., Lusby P. J. (2020). J. Am. Chem. Soc..

[cit18] Lewis J. E. M., Gavey E. L., Cameron S. A., Crowley J. D. (2012). Chem. Sci..

[cit19] Vasdev R. A. S., Gaudin L. F., Preston D., Jogy J. P., Giles G. I., Crowley J. D. (2018). Front. Chem..

[cit20] Schmidt A., Molano V., Hollering M., Pothig A., Casini A., Kuhn F. E. (2016). Chem.–Eur. J..

[cit21] Gonell S., Caumes X., Orth N., Ivanovic-Burmazovic I., Reek J. N. H. (2019). Chem. Sci..

[cit22] Wang Q. Q., Gonell S., Leenders S. H., Durr M., Ivanovic-Burmazovic I., Reek J. N. (2016). Nat. Chem..

[cit23] Zaffaroni R., Bobylev E. O., Plessius R., van der Vlugt J. I., Reek J. N. H. (2020). J. Am. Chem. Soc..

[cit24] Leenders S. H. A. M., Dürr M., Ivanović-Burmazović I., Reek J. N. H. (2016). Adv. Synth. Catal..

[cit25] Ueda Y., Ito H., Fujita D., Fujita M. (2017). J. Am. Chem. Soc..

[cit26] Acharyya K., Bhattacharyya S., Sepehrpour H., Chakraborty S., Lu S., Shi B., Li X., Mukherjee P. S., Stang P. J. (2019). J. Am. Chem. Soc..

[cit27] Pollock J. B., Cook T. R., Schneider G. L., Lutterman D. A., Davies A. S., Stang P. J. (2013). Inorg. Chem..

[cit28] Pollock J. B., Cook T. R., Stang P. J. (2012). J. Am. Chem. Soc..

[cit29] Yan X., Wei P., Liu Y., Wang M., Chen C., Zhao J., Li G., Saha M. L., Zhou Z., An Z., Li X., Stang P. J. (2019). J. Am. Chem. Soc..

[cit30] Liu C. L., Bobylev E. O., Fu Y., Poole 3rd D. A., Robeyns K., Fustin C. A., Garcia Y., Reek J. N. H., Singleton M. L. (2020). Chem.–Eur. J..

[cit31] Fujita D., Takahashi A., Sato S., Fujita M. (2011). J. Am. Chem. Soc..

[cit32] Kaiser F., Schmidt A., Heydenreuter W., Altmann P. J., Casini A., Sieber S. A., Kühn F. E. (2016). Eur. J. Inorg. Chem..

[cit33] Pothig A., Casini A. (2019). Theranostics.

[cit34] Kai S., Shigeta T., Kojima T., Hiraoka S. (2017). Chem.–Asian J..

[cit35] Fujita D., Yokoyama H., Ueda Y., Sato S., Fujita M. (2015). Angew. Chem., Int. Ed..

[cit36] Yoneya M., Tsuzuki S., Yamaguchi T., Sato S., Fujita M. (2014). ACS Nano.

[cit37] Poole D. A., Bobylev E. O., Mathew S., Reek J. N. H. (2020). Chem. Sci..

[cit38] Li H., Luo J., Liu T. (2016). Chem.–Eur. J..

[cit39] Zeng L., Xiao Y., Jiang J., Fang H., Ke Z., Chen L., Zhang J. (2019). Inorg. Chem..

[cit40] Zhukhovitskiy A. V., Zhong M., Keeler E. G., Michaelis V. K., Sun J. E., Hore M. J., Pochan D. J., Griffin R. G., Willard A. P., Johnson J. A. (2016). Nat. Chem..

[cit41] Wang M., Liu J., Luo T., Xue Y., Mao L., Stang P. J. (2021). Angew. Chem., Int. Ed..

[cit42] Jiang W.-L., Shen J.-C., Peng Z., Wu G.-Y., Yin G.-Q., Shi X., Yang H.-B. (2020). J. Mater. Chem. A.

[cit43] Puig E., Desmarets C., Gontard G., Rager M. N., Cooksy A. L., Amouri H. (2019). Inorg. Chem..

[cit44] Chand D. K., Balaji G., Manivannan R., Athilakshmi J. (2006). Tetrahedron Lett..

