# Supplementary material for: Selective formation of Pt12L24 nanospheres by ligand design
Source: Chem Sci. 2021 Apr 28;12(22):7696–705. doi: 10.1039/d1sc01295a (PMC8188466; doi:10.1039/d1sc01295a)
Supplement: SC-012-D1SC01295A-s001 [file SC-012-D1SC01295A-s001.pdf]

Electronic Supplementary Material (ESI) for Chemical Science.
This journal is © The Royal Society of Chemistry 2021

Supporting information for

Selective formation of $Pt_{12}L_{24}$ nanospheres by ligand design

Eduard. O. Bobylev,† David A. Poole III,† Bas de Bruin† and Joost N. H. Reek*†

† van 't Hoff Institute for Molecular Sciences, University of Amsterdam, Science Park 904, 1098 XH Amsterdam, the Netherlands.

List of content:

SI1:	Synthesis of building blocks	3
SI2:	Sphere synthesis	27
SI3:	Variable temperature studies	73
SI4:	Ligand exchange studies	84
SI5:	Application for other structures	104
SI6:	Possible formation pathways	108
SI7:	Computational Methods and calculated relative energies for different type of assemblies	109
SI8:	MS calibration of LOMe and LPy and MSMS analysis	110
SI9:	Colum chromatography on self-assembled $Pt_nL^{OMe}{}_{2n}$	116
SI10:	Hydrodynamic Radius Considerations	118

Materials and methods

General procedures: All synthetic procedures were carried out under an nitrogen atmosphere using standard Schlenk techniques. All commercially available chemicals were used as received without further purification. Solvents used for synthesis were dried, distilled and degassed with the most suitable method. Column chromatography was performed open to air using solvents as received.

Cryospray-ionization MS (CSI-MS): Mass spectra were collected on a HR-ToF Bruker Daltonik GmbH (Bremen, Germany) Impact II, an ESI-ToF MS capable of resolution of at least 40000 FWHM, which was coupled to a Bruker cryo-spray unit. Detection was in positive-ion mode and the source voltage was between 4 and 6 kV. The sample was introduced with a syringe pump at a flow rate of 18 μl/hr. The drying gas (N_2) was held at 40°C and the spray gas was held at 60°C. The machine was calibrated prior to every experiment via direct infusion of a TFA-Na solution, which provided a m/z range of singly charged peaks up to 3500 Da in both ion modes. Software acquisition Compass 2.0 for Otof series. Software processing m-mass.

Synthesis of building blocks (SI1)

Synthesis of $\mathbf{L^{OMe}}$

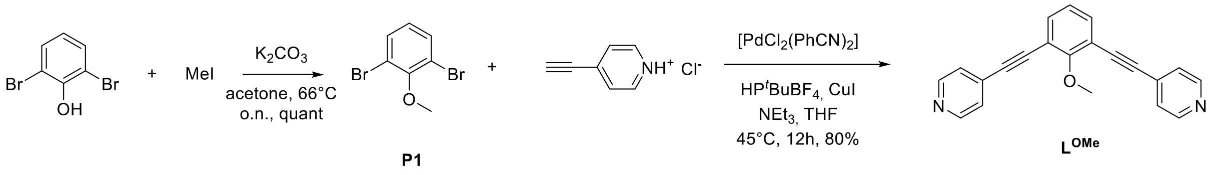

Scheme S1. Synthetic route for $\mathbf{L^{OMe}}$.

The building block $\mathbf{L^{OMe}}$ was synthesized according to a modified literature procedure.[1,2]

Briefly: Intermediate **P1** was synthesized by refluxing a solution of 2,6-dibromophenol (2 g, 8 mmol, 1 eq.), potassium carbonate (3.3 g, 23.8 mmol, 3 eq.) and MeI (1.7 g, 12 mmol, 1.5 eq.) in 100 mL acetone overnight. The volatiles were removed under reduced pressure. The solid was taken up into 200 mL DCM and washed with 3x100 mL $NaOH_{aq}$ (1M). The organic phase was separated, dried with Na_2SO_4 and the volatiles were removed under reduced pressure to afford **P1** as a colorless oil (2.1g, quant).

HP^tBuBF_4 (130 mg, 0.45 mmol, 0.12 eq.) and $Pd(PhCN)_2Cl_2$ (86 mg, 0.225 mmol, 0.06 eq.) were dissolved in a mixture of degassed THF (20 mL) and trimethylamine (20 mL). The solution was stirred 20 min at room temperature before 2,6-dibromo-methoxybenzen (1.00 g, 3.75 mmol, 1 eq.) and 4-ethynylpyridine hydrochloride (1.22 g, 9.39 mmol, 2.5 eq.) were added. The solution was gently heated up to 45°C and copper(I) iodide (30 mg, 0.15 mmol) was added. This mixture was stirred at 45 °C for 24 h under nitrogen atmosphere. The reaction mixture was poured into 400 mL ethylacetate, filtrated over a pad of sand and evaporated in *vacuo*. The crude product was purified by column chromatography on silica gel (DCM/MeOH = 99/1) to give $\mathbf{L^{OMe}}$ as a white powder (0.93g, 80%). ^{1}H NMR (400 MHz, Acetonitrile-d_3) δ 8.65 (d, J = 6.1 Hz, 4H), 7.65 (d, J = 7.7 Hz, 2H), 7.59 – 7.32 (m, 4H), 7.23 (t, J = 7.7 Hz, 1H), 4.17 (s, 3H). LogD(m^2/s) at 25°C (MeCN-d_3): -8.823 (d = 0.84 nm).

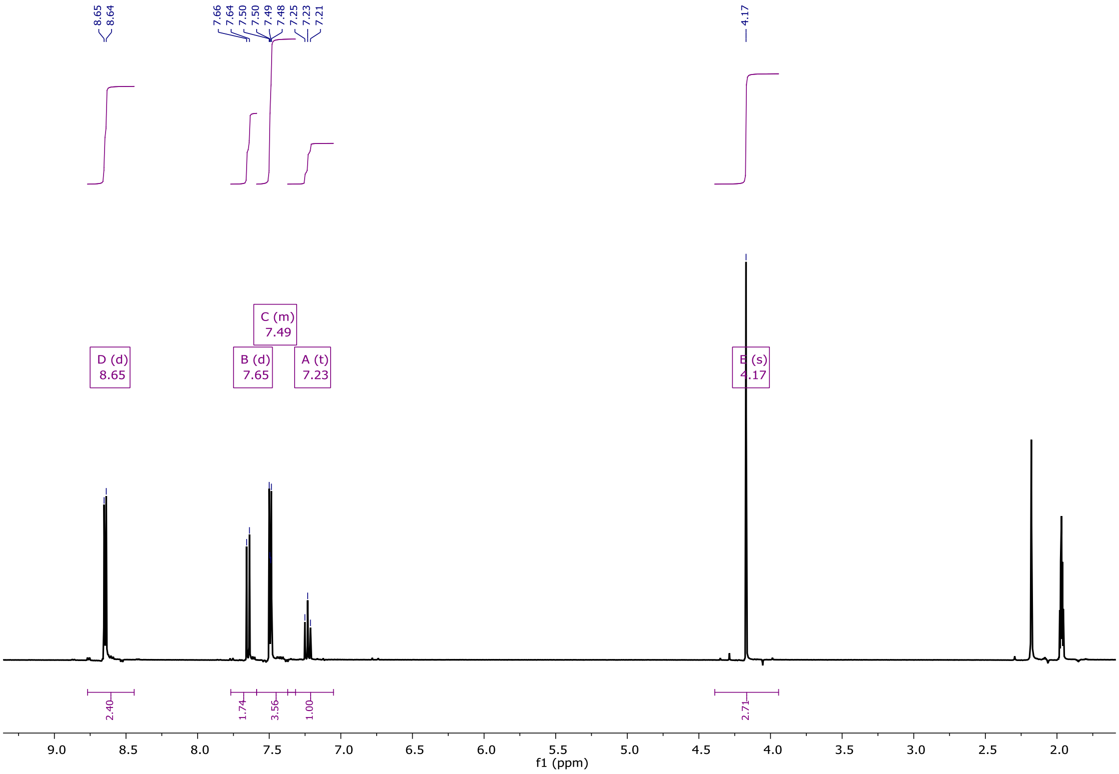

Figure S1. L^{OMe} building block, ^{1}H NMR in $MeCN\text{-}d_3$.

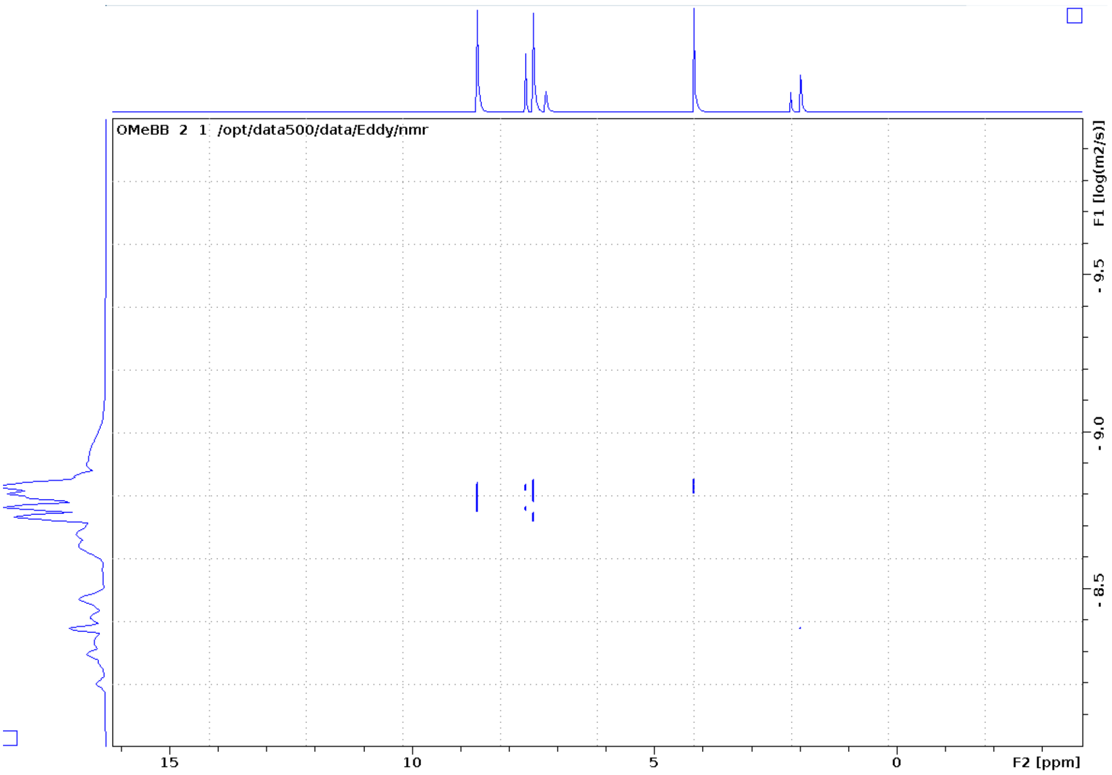

Figure S2. L^{OMe} building block, DOSY NMR in $MeCN\text{-}d_3$ at 25°C.

Synthesis of $\mathbf{L^{OBn}}$

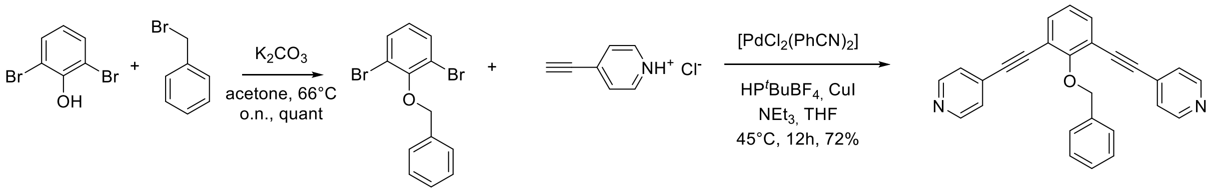

PBn

$\mathbf{L^{OBn}}$

Scheme S2. Synthetic route for $\mathbf{L^{OBn}}$.

The intermediate **PBn** was synthesized according to a modified literature procedure.[3]

Briefly: Intermediate **PBn** was synthesized by refluxing a solution of 2,6-dibromophenol (2 g, 8 mmol, 1 eq.), potassium carbonate (3.3 g, 23.8 mmol, 3 eq.) and benzylbromide (2.05 g, 12 mmol, 1.5 eq.) in 100 mL acetone overnight. The volatiles were removed under reduced pressure. The solid was taken up into 200 mL DCM and washed with 3x100 mL $NaOH_{aq}$ (1M). The organic phase was separated, dried with Na_2SO_4 and the volatiles were removed under reduced pressure to afford **PBn** as a colorless oil (2.7 g, quant).

HP^tBuBF_4 (130 mg, 0.45 mmol, 0.12 eq.) and $Pd(PhCN)_2Cl_2$ (86 mg, 0.225 mmol, 0.06 eq.) were dissolved in a mixture of degassed THF (20 mL) and trimethylamine (20 mL). The solution was stirred 20 min at room temperature before **PBn** (1.28 g, 3.75 mmol, 1 eq.) and 4-ethynylpyridine hydrochloride (1.22 g, 9.39 mmol, 2.5 eq.) were added. The solution was gently heated up to 45°C and copper(I) iodide (30 mg, 0.15 mmol) was added. This mixture was stirred at 45 °C for 24 h under nitrogen atmosphere. The reaction mixture was poured into 400 mL ethylacetate, filtrated over a pad of sand and evaporated in *vacuo*. The crude product was purified by column chromatography on silica gel (DCM/MeOH = 99/1) to give $\mathbf{^{L}OBn}$ as a white powder (1 g, 72%). ^1H NMR (400 MHz, Acetonitrile-d_3) δ 8.66 – 8.58 (m, 4H), 7.66 (d, *J* = 7.7 Hz, 2H), 7.62 – 7.56 (m, 2H), 7.43 – 7.35 (m, 6H), 7.25 (t, *J* = 7.7 Hz, 1H), 5.41 (s, 2H). ^{13}C NMR (101 MHz, CD_3CN) δ 161.02, 150.01, 137.13, 134.85, 130.49, 128.48, 128.31, 125.23, 124.43, 91.20, 89.15, 76.13. LogD(m^2/s) at 25°C (MeCN-d_3): -8.783 (d = 0.96 nm). HR-ESI-MS, calculated for $[C_{27}H_{18}N_2O]H^+$ 387.1492, obtained 387.1356.

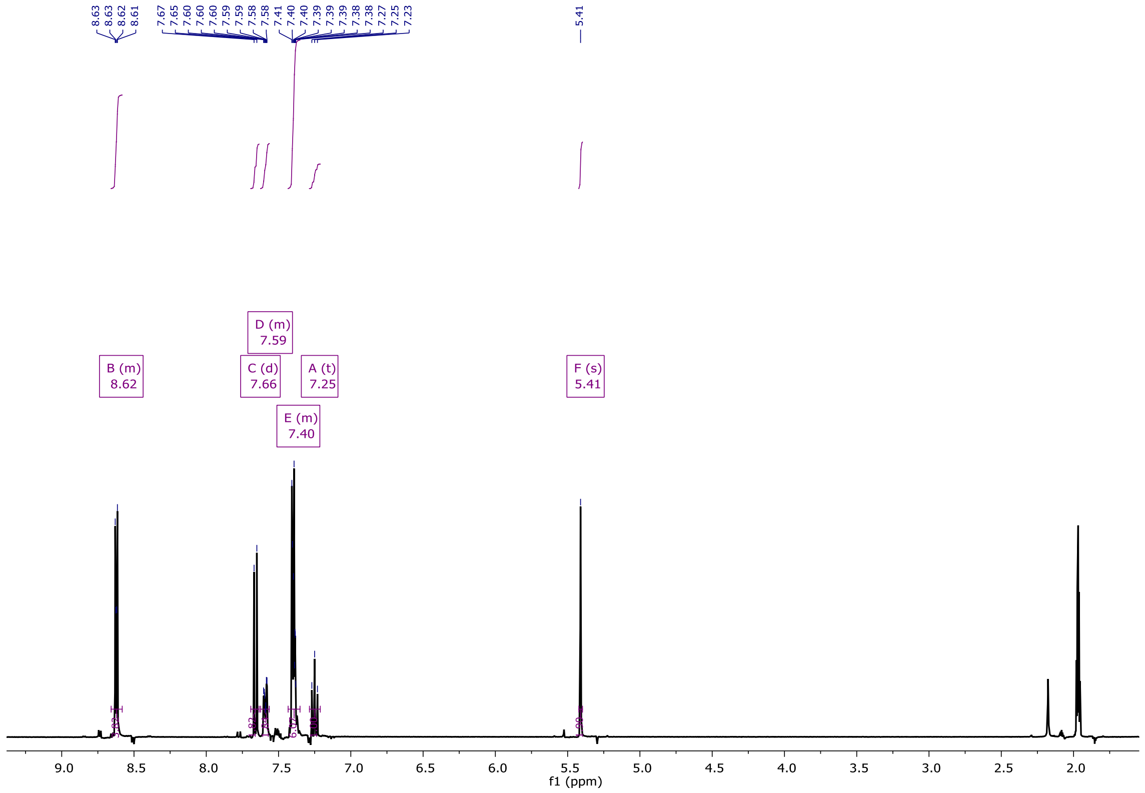

Figure S3. L^OBn building block, ^1H NMR in MeCN-d3.

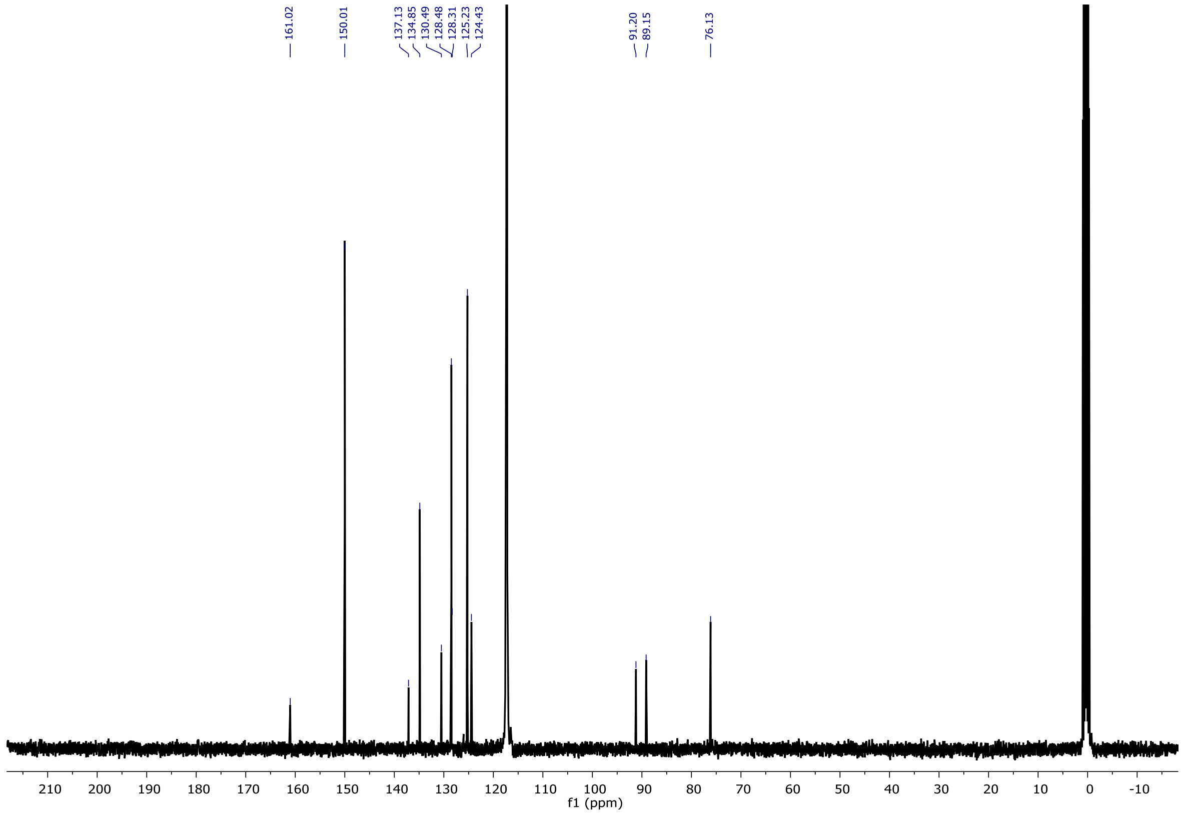

Figure S4. L^OBn building block, ^13C NMR in MeCN-d3.

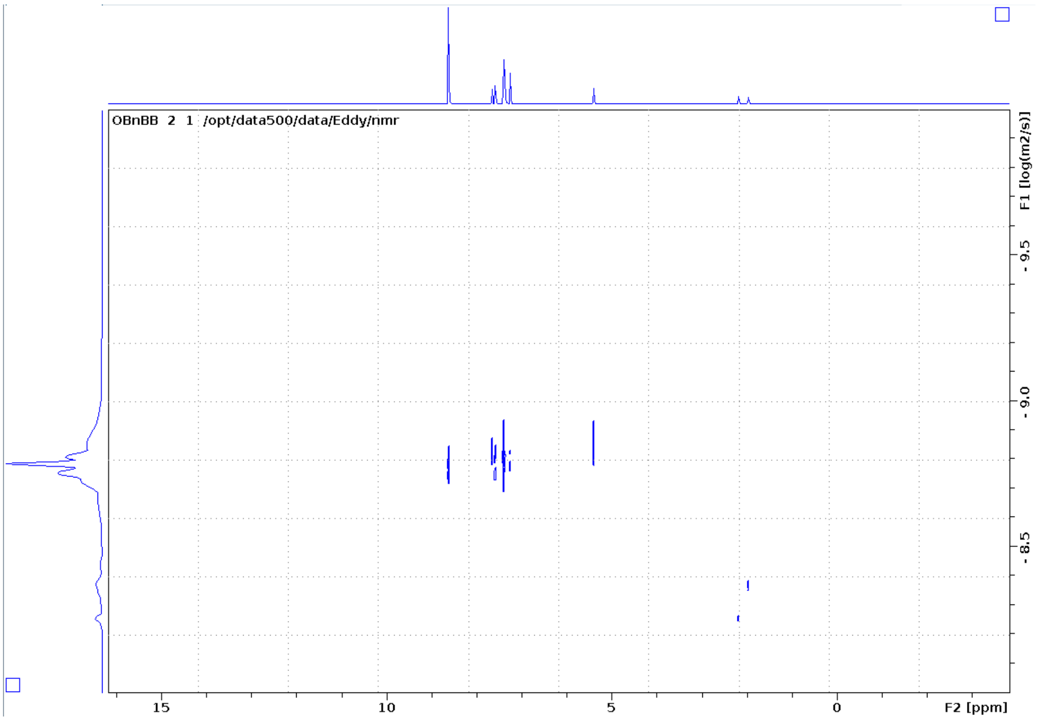

Figure S5. L^{OBn} building block, DOSY NMR in $MeCN-d_3$ at 25°C.

Synthesis of intermediate **I4**

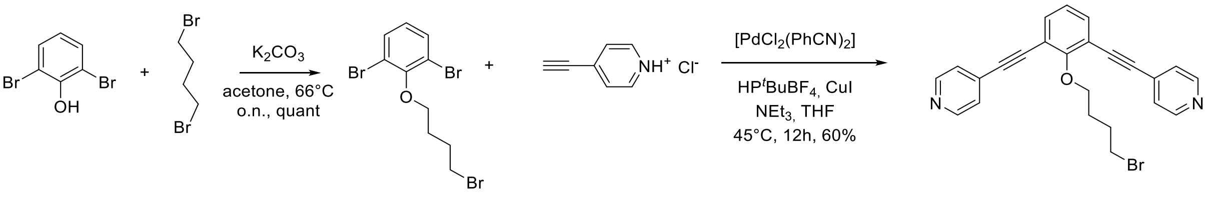

Scheme S3. Synthetic route for intermediate **I4**.

I4 was synthesized according to a slightly modified literature procedure.[4] Briefly, 2,6-dibromophenol (5g, 19.84 mmol, 1 eq.) was refluxed with 1,4-dibromobutane (85.7 g, 397 mmol, 20 eq.) and K_2CO_3 (8.2 g, 60 mmol, 3 eq.) in 100 mL acetone overnight. Then, all volatiles were removed under reduced pressure at 90°C to afford the first intermediate **P4** in quantitative yield (7.65 g).

To a degassed solution of $[Pd(PhCN)Cl_2]$ (126 mg, 0.33 mmol, 0.08 eq.) and $HP^{t}BuBF_4$ (190 mg, 0.66 mmol, 0.12 eq.) in 20 mL NEt_3 and 20 mL THF, **P4** (2.13 g, 5.5 mmol, 1 eq.) and 4-ethynylpyridine hydrochloride (2 g, 14.38 mmol, 2.5 eq.) were added. The mixture was heated to 45°C and CuI (42 mg, 0.22 mmol, 0.04 eq.) was added. After stirring the mixture at 45°C overnight, the solution was poured into 400 mL EtOAc and filtered over a pad of sand. The volatiles were removed under reduced pressure and the obtained crude material was purified by column chromatography (SiO_2, MeOH:DCM, 1:99 -> 4:96) to afford **I4** as a colorless oil (1.4 g, 60%). ^1H NMR (400 MHz, Chloroform-*d*) δ 8.73 – 8.63

(m, 4H), 7.60 (d, J = 7.7 Hz, 2H), 7.56 – 7.49 (m, 4H), 7.18 (t, J = 7.7 Hz, 1H), 4.39 (t, J = 6.0 Hz, 2H), 3.51 (t, J = 6.7 Hz, 2H), 2.31 – 2.21 (m, 2H), 2.14 – 2.00 (m, 2H).

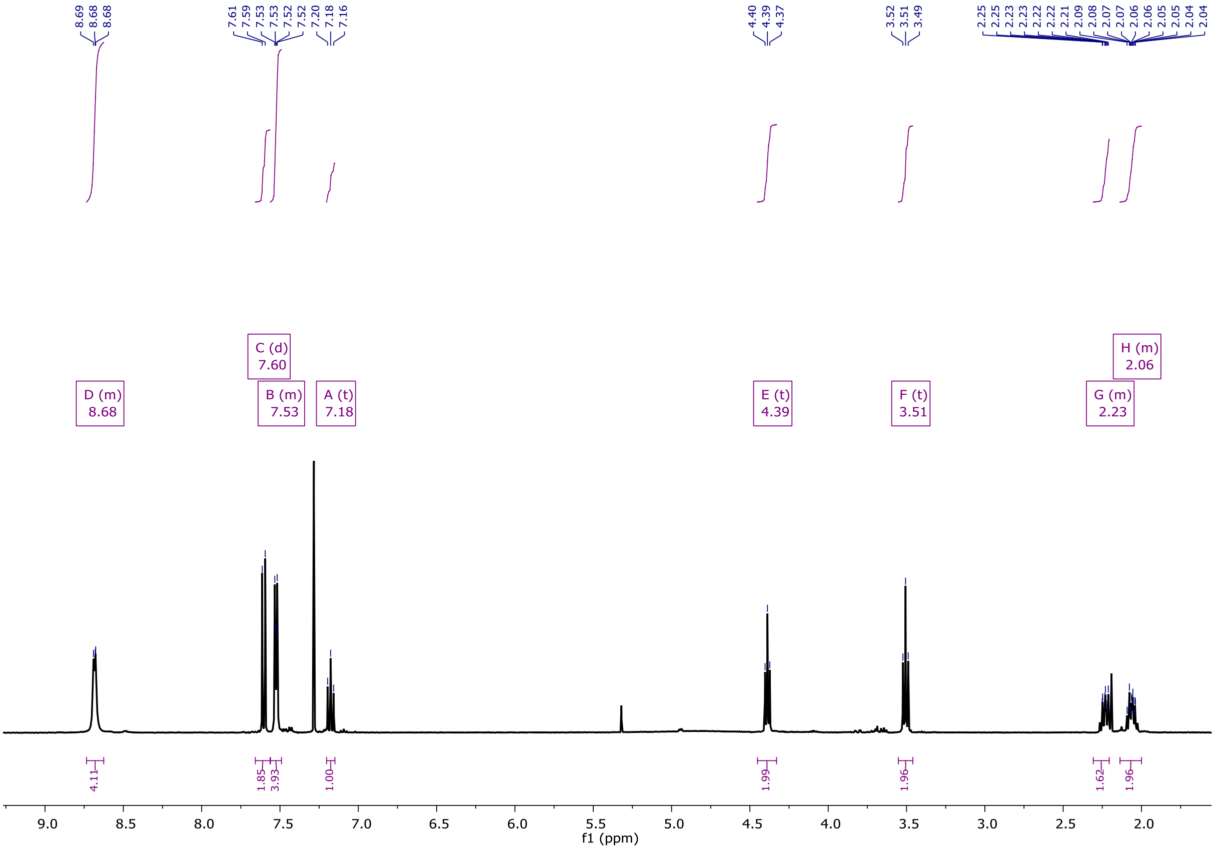

Figure S6. I4 precursor, ^{1}H NMR in MeCN-d$_3$.

Synthesis of **L^Imi^**, **L^Py^**, **L^Cou^** and **L^ImiAd^**.

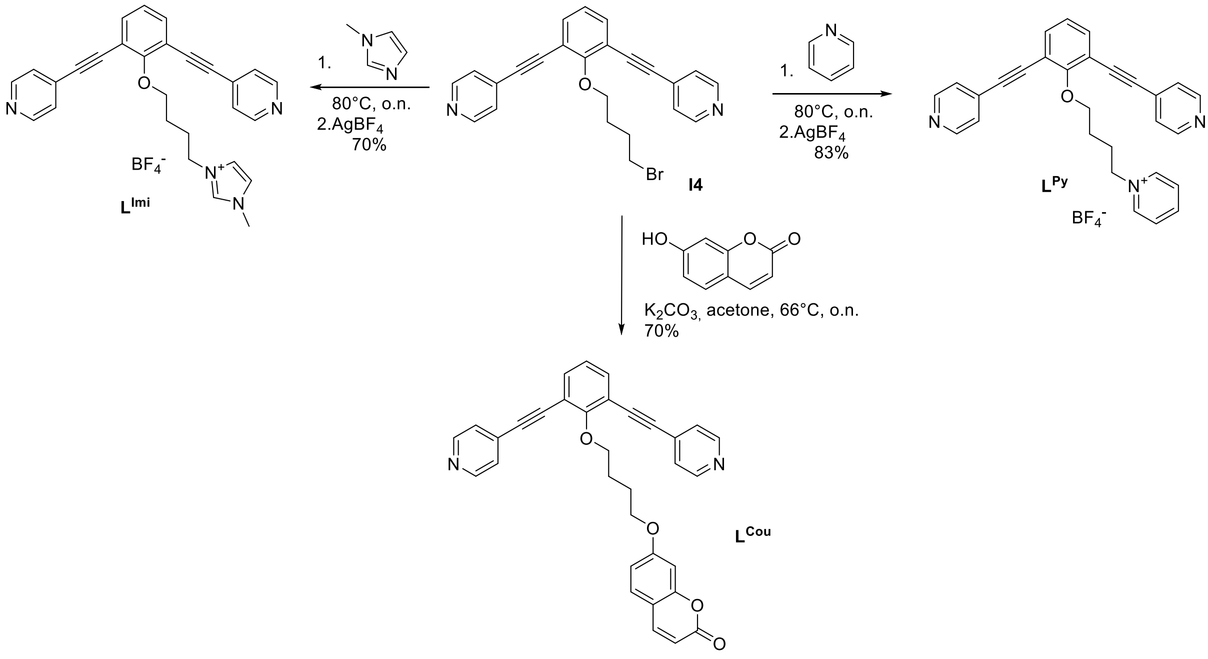

Scheme S4. Synthetic route for intermediate C4-linked building blocks **L^{Imi}**, **L^{Py}** and **L^{Cou}**.

L^{Imi}: To a solution of **I4** (0.7 g, 1.8 mmol, 1 eq.) in 10 mL MeCN, 1-methylimidazole (0.2 mL, 2.5 mmol, 1.5 eq.) was added. The mixture was heated at 90°C for 24 h. The solvent was removed under reduced pressure. The resulting oil was dissolved in a minimum amount of DCM and precipitated in 1L Et_2O to afford **C4IMIBr**. To a solution of **C4IMIBr** (100 mg, 0.19 mmol, 1 eq.) in 3 mL DCM and 3 mL MeCN, $AgBF_4$ (38 mg, 0.19 mmol, 1 eq.) were added in the dark. The suspension was stirred over night at room temperature. The solvent was removed under reduced pressure. The residue was dissolved in 5 mL DCM and filtered over a pad of celite. The volatiles were removed under reduced pressure to afford of **L^{Imi}** (71 mg, 0.13 mmol, 70%) as a bright beige oil. ^{1}H NMR (400 MHz, Acetonitrile-*d*3) δ 8.65 – 8.62 (m, 4H), 8.38 (d, *J* = 1.7 Hz, 1H), 7.66 (d, *J* = 7.7 Hz, 2H), 7.48 – 7.42 (m, 4H), 7.31 (dt, *J* = 15.6, 1.9 Hz, 2H), 7.25 (t, *J* = 7.7 Hz, 1H), 4.38 (t, *J* = 5.9 Hz, 2H), 4.23 (t, *J* = 7.4 Hz, 2H), 3.79 (s, 3H), 2.17 (tt, *J* = 8.4, 6.9 Hz, 2H), 1.94 – 1.86 (m, 2H). ^{13}C NMR (101 MHz, CD_3CN) δ 161.13, 150.08, 135.86, 134.91, 130.49, 125.23, 124.42, 123.69, 122.19, 116.75, 90.99, 89.02, 73.90, 54.35, 49.29, 35.85, 27.07, 26.62. ^{19}F NMR (282 MHz, $CDCl_3$) δ -147.43, -147.49. LogD(m^2/s) at 25°C (MeCN-d3): -9.011 (d = 1.30 nm). HR-ESI-MS, calculated for $C_{28}H_{25}N_4O$ 433.2023, obtained 433.1999.

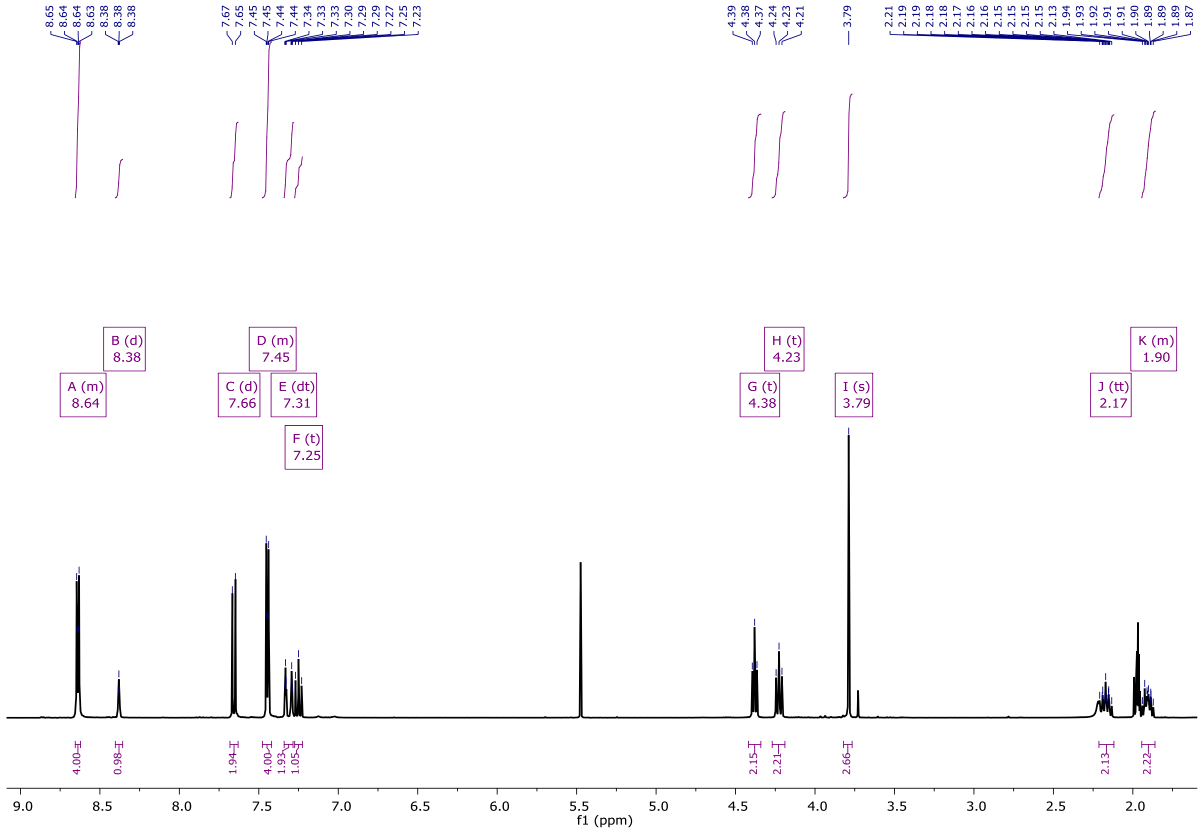

Figure S7. L^{Imi} building block, ^{1}H NMR in MeCN-d$_3$.

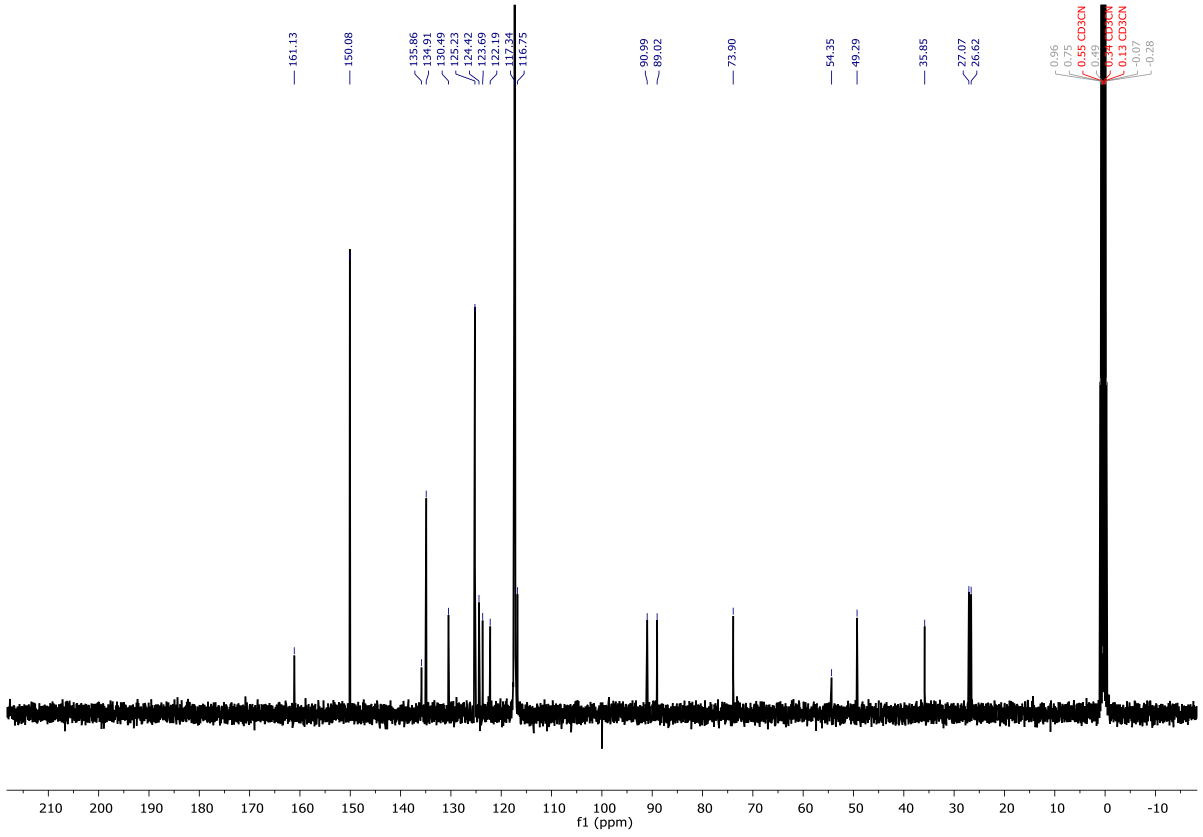

Figure S8. L^{Imi} building block, ^{13}C NMR in MeCN-d$_3$.

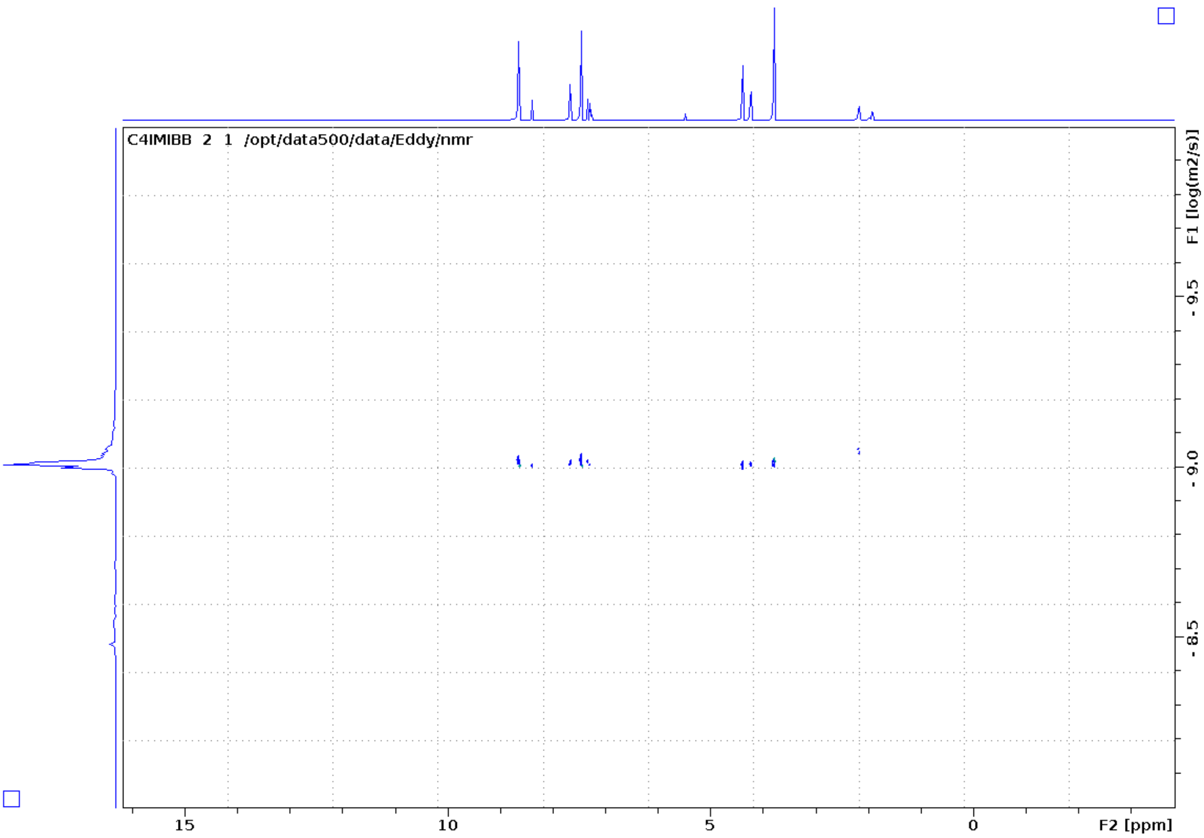

Figure S9. L^{Imi} building block, DOSY in MeCN-d$_3$ at 25°C.

L^{Py}: To a solution of **I4** (0.7 g, 1.8 mmol, 1 eq.) in 10 mL MeCN, 10 mL pyridine was added. The mixture was heated at 90°C for 24 h. The solvent was removed under reduced pressure. The resulting oil was dissolved in a minimum amount of DCM and precipitated in 1L Et_2O to afford **C4PyBr**. To a solution of **C4PyBr** (100 mg, 0.19 mmol, 1 eq.) in 3 mL DCM and 3 mL MeCN, $AgBF_4$ (38 mg, 0.19 mmol, 1 eq.) was added in the dark. The suspension was stirred over night at room temperature. The solvent was removed under reduced pressure. The residue was dissolved in 5 mL DCM and filtered over a pad of celite. The volatiles were removed under reduced pressure to afford **L^{Py}** (84 mg, 0.16 mmol, 83 %) as a bright brown oil. ^{1}H NMR (400 MHz, Acetonitrile-*d*$_3$) δ 8.76 – 8.72 (m, 2H), 8.63 – 8.61 (m, 4H), 8.46 (tt, *J* = 7.8, 1.4 Hz, 1H), 7.96 (t, *J* = 7.1 Hz, 2H), 7.65 (d, *J* = 7.8 Hz, 2H), 7.46 – 7.41 (m, 4H), 7.25 (t, *J* = 7.7 Hz, 1H), 4.71 – 4.62 (m, 2H), 4.39 (t, *J* = 5.9 Hz, 2H), 2.32 (tt, *J* = 9.3, 6.9 Hz, 2H). ^{13}C NMR (101 MHz, CD_3CN) δ 161.00, 150.09, 145.76, 144.40, 134.90, 130.43, 128.34, 125.22, 124.47, 116.75, 91.01, 88.97, 73.79, 61.41, 28.38, 26.47. LogD(m^2/s) at 25°C (MeCN-d$_3$): -8.946 (d = 1.12 nm). HR-ESI-MS, calculated for $C_{29}H_{24}N_3O$ 430.1913, obtained 430.1876.

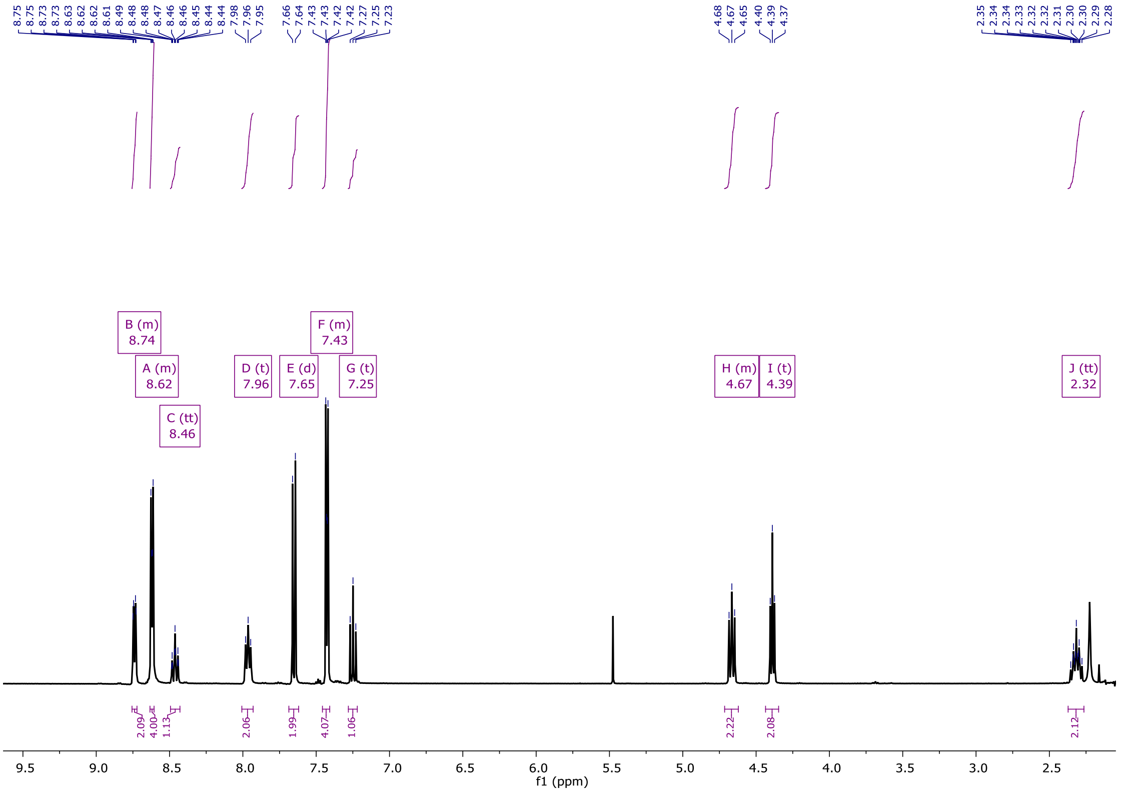

Figure S10. L^{Py} building block, ^1H NMR in MeCN-d_3.

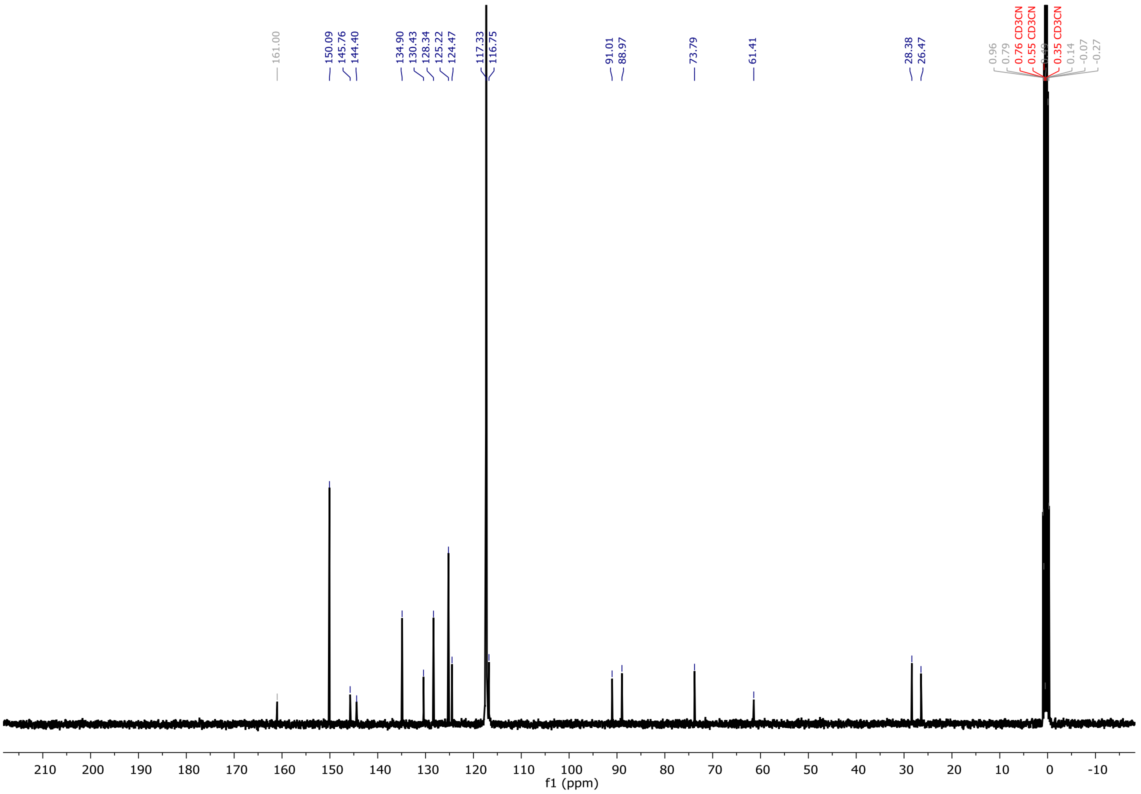

Figure S11. L^{Py} building block, ^{13}C NMR in MeCN-d_3.

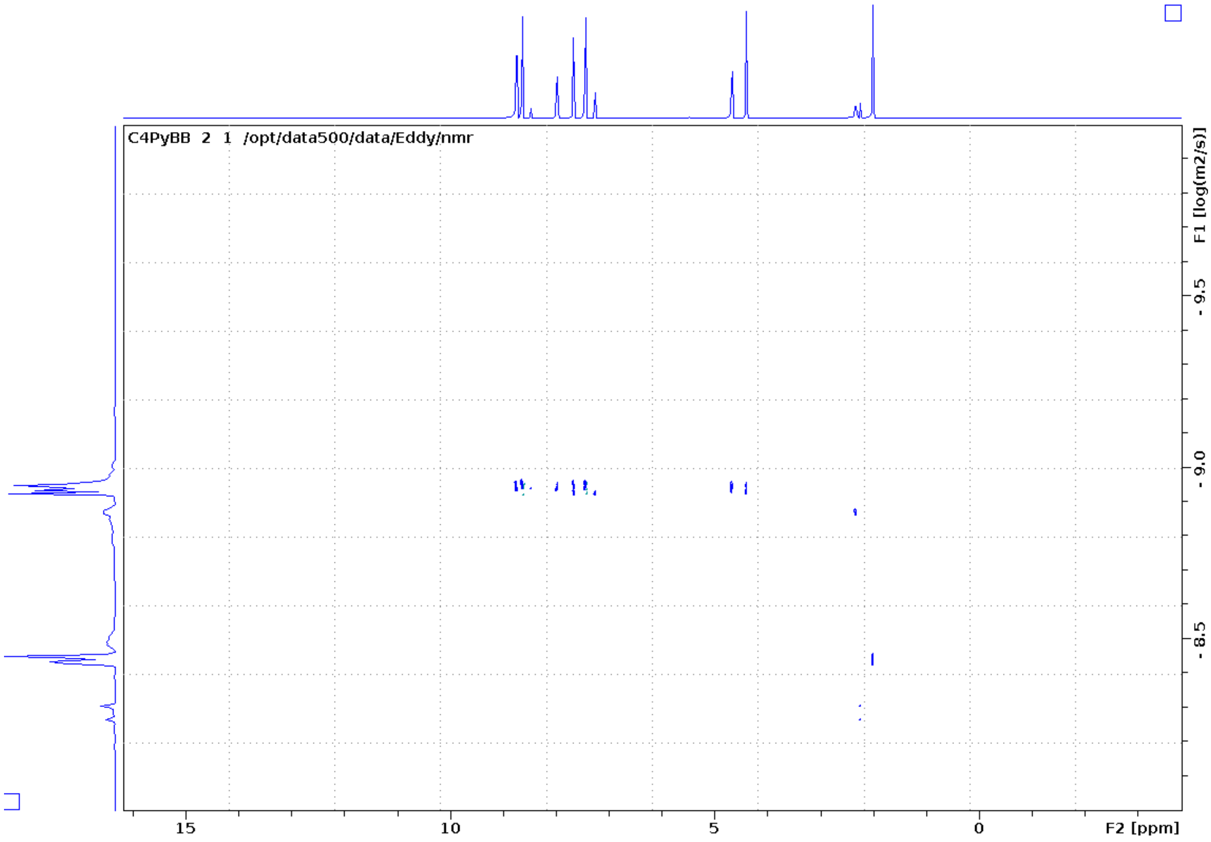

Figure S12. L^{Py} building block, DOSY in MeCN-d_3 at 25°C.

L^{Cou}: To a solution of **I4** (0.2 g, 0.46 mmol, 1 eq.) in 30 mL acetone, umbelliferone (83 mg, 0.51 mmol, 1.1 eq.) and K_2CO_3 (0.19 g, 1.4 mmol, 3 eq.) were added. The mixture was heated to reflux for 24 h. The solvent was removed under reduced pressure. The resulting solid was dissolved in 100 mL water and 100 mL DCM. The organic phase was separated and washed with 3x100 mL 1N NaOH. The organic phase was dried with $NaSO_4$ and the volatiles were removed under reduced pressure to afford **L^{Cou}** (165 mg, 0.32 mmol, 70 %) as a white solid. ^{1}H NMR (400 MHz, Acetonitrile-*d*$_3$) δ 8.63 – 8.54 (m, 4H), 7.79 (d, *J* = 9.5 Hz, 1H), 7.65 (d, *J* = 7.7 Hz, 2H), 7.49 – 7.42 (m, 5H), 7.23 (t, *J* = 7.7 Hz, 1H), 6.85 – 6.76 (m, 2H), 6.21 (d, *J* = 9.5 Hz, 1H), 4.45 (t, *J* = 5.9 Hz, 2H), 4.15 (t, *J* = 6.3 Hz, 2H), 2.16 – 2.03 (m, 4H). ^{13}C NMR (101 MHz, CD_3CN) δ 162.11, 161.49, 160.73, 155.86, 149.99, 143.75, 134.89, 130.51, 129.19, 125.19, 124.13, 116.71, 112.67, 101.15, 89.14, 74.41, 68.34, 26.75, 25.69. LogD(m^2/s) at 25°C (MeCN-d_3): -8.881 (d = 0.96 nm). HR-ESI-MS, calculated for $[C_{33}H_{24}N_2O_4]H^+$ 513.1809, obtained 513.1788.

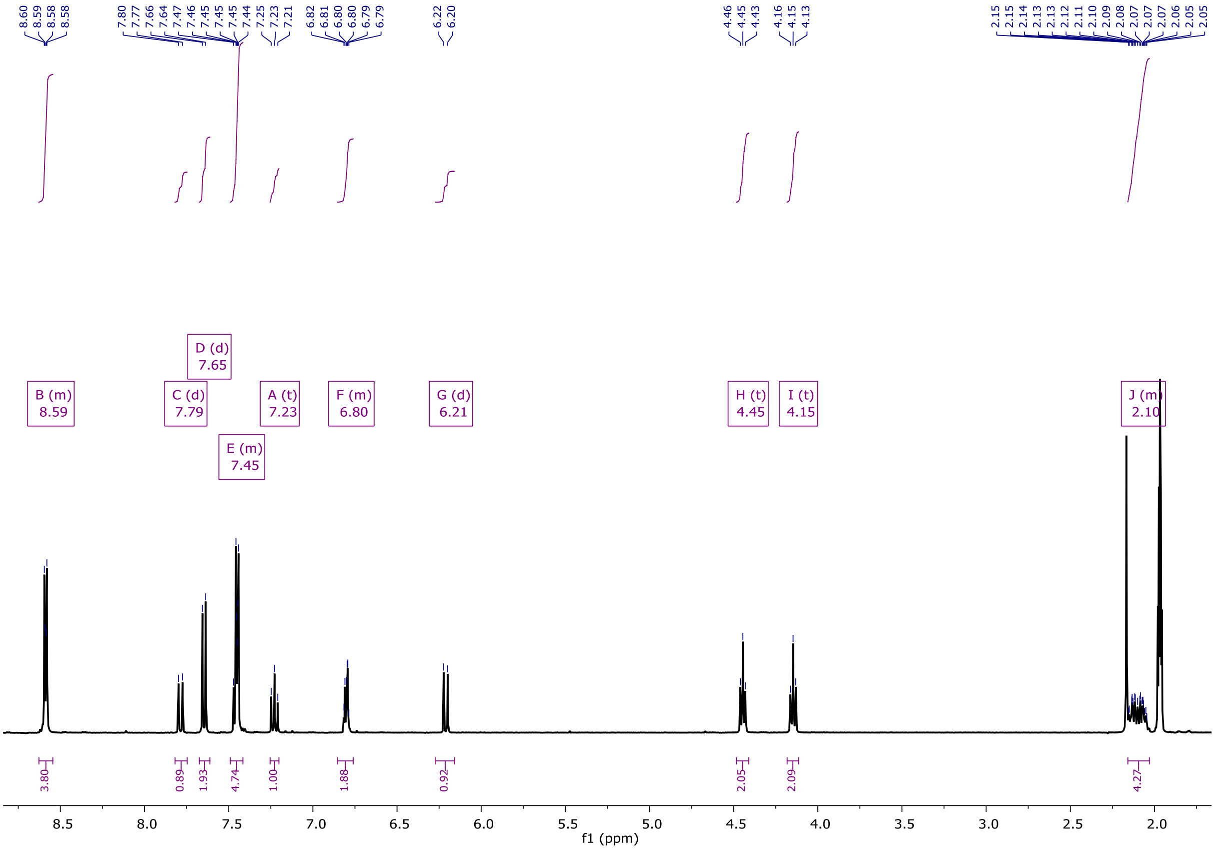

Figure S13. L^Cou building block, ^{1}H NMR in MeCN-d3.

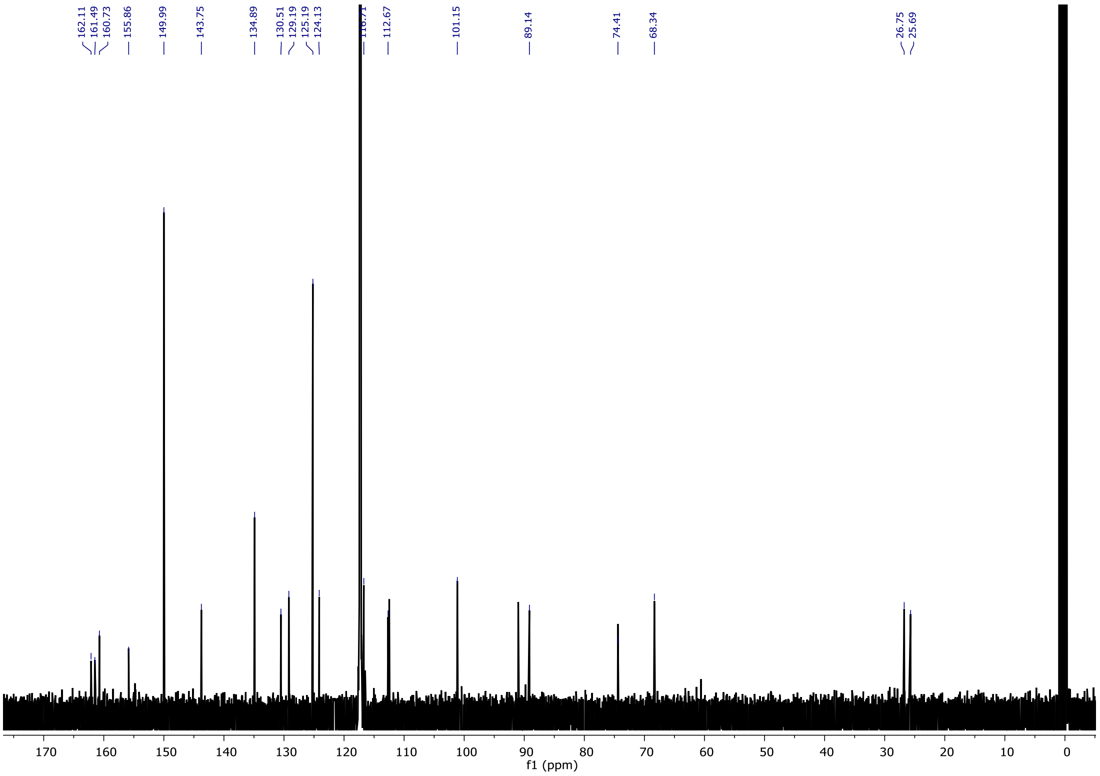

Figure S14. L^Cou building block, ^{13}C NMR in MeCN-d3.

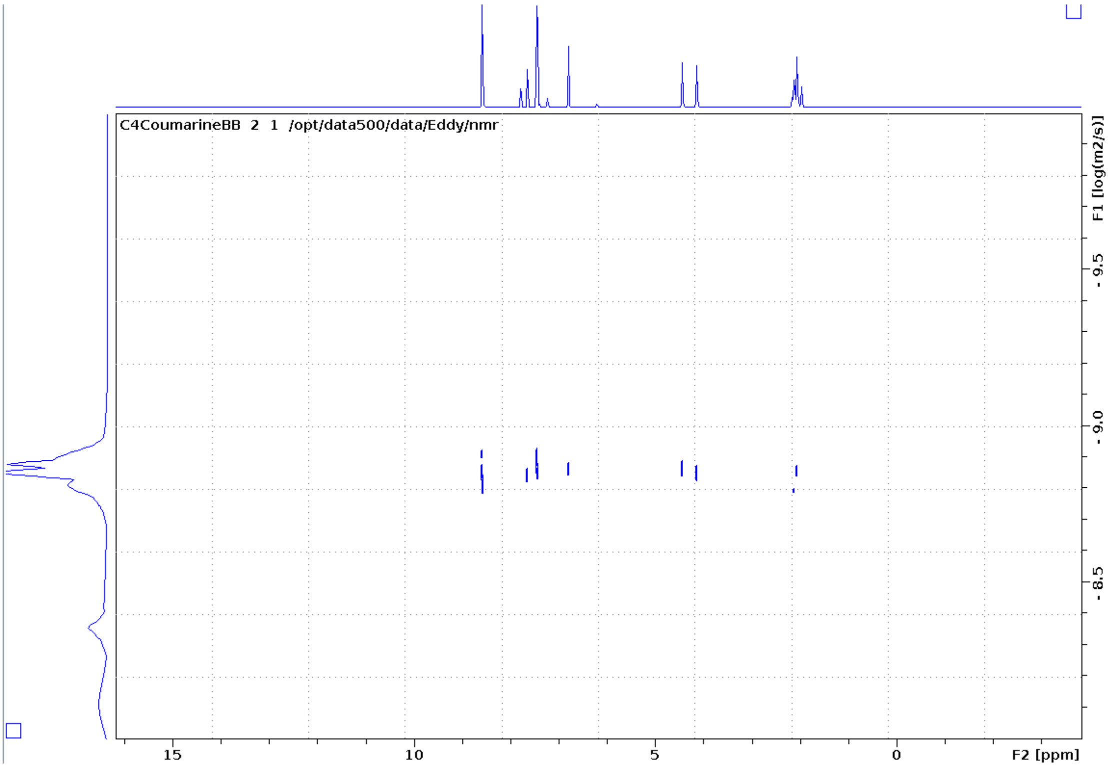

Figure S15. $\mathbf{L^{Cou}}$ building block, DOSY in MeCN-d_3 at 25°C.

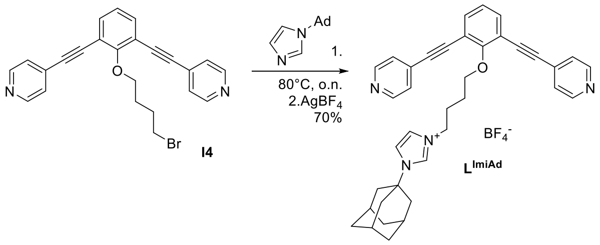

Scheme S5. Synthetic route for intermediate C4-linked building blocks $\mathbf{L^{ImiAd}}$.

$\mathbf{L^{ImiAd}}$: To a solution of **I4** (0.7 g, 1.8 mmol, 1 eq.) in 10 mL MeCN, 1-adamantylimidazole (0.5 mg, 2.5 mmol, 1.5 eq.) was added. The mixture was heated at 90°C for 24 h. The solvent was removed under reduced pressure. The resulting oil was dissolved in a minimum amount of DCM and precipitated in 1L Et_2O to afford **C4IMIAdBr**. To a solution of **C4IMIAdBr** (200 mg, 0.31 mmol, 1 eq.) in 3 mL DCM and 3 mL MeCN, $AgBF_4$ (61 mg, 0.31 mmol, 1 eq.) were added in the dark. The suspension was stirred over night at room temperature. The solvent was removed under reduced pressure. The residue was dissolved in 5 mL DCM and filtered over a pad of celite. The volatiles were removed under reduced pressure to afford of $\mathbf{L^{ImiAd}}$ (142 mg, 0.22 mmol, 70%) as a bright beige oil. ^{1}H NMR (300 MHz, Acetonitrile-d_3) δ 8.97 (s, 1H), 8.71 – 8.56 (m, 5H), 7.66 (dd, *J* = 7.8, 2.5 Hz, 2H), 7.55 (t, *J* = 2.0 Hz, 1H), 7.52 – 7.38 (m, 6H), 7.25 (dd, *J* = 9.0, 6.4 Hz, 1H), 4.35 (dt, *J* = 22.6, 6.5 Hz, 5H), 1.85 – 1.63 (m, 9H), other adamantane and CH_2 signals are located below solvent and water signal. ^{13}C NMR (101 MHz, CD_3CN) δ 150.10, 134.91, 130.45, 125.26, 124.42, 122.25, 119.01, 116.76, 91.01, 89.00, 73.98, 49.33, 41.97, 34.89, 29.42, 26.95, 26.68. ESI-MS, calculated for $C_{37}H_{37}N_4O$ 553.2962, obtained 553.2989.

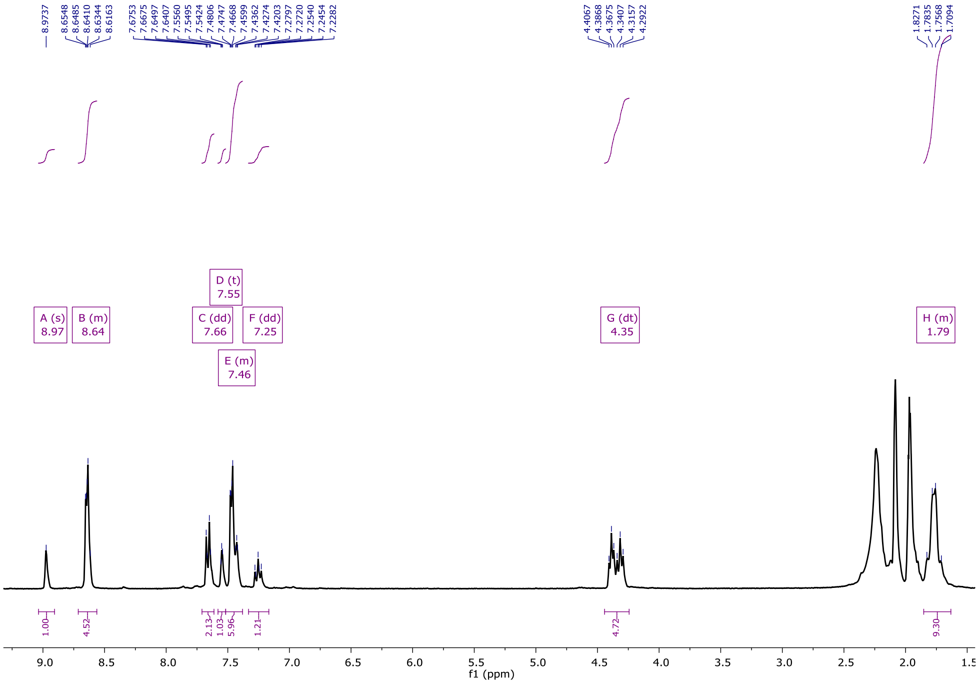

Figure S16. L^ImiAd building block, ^{1}H NMR in MeCN-d3.

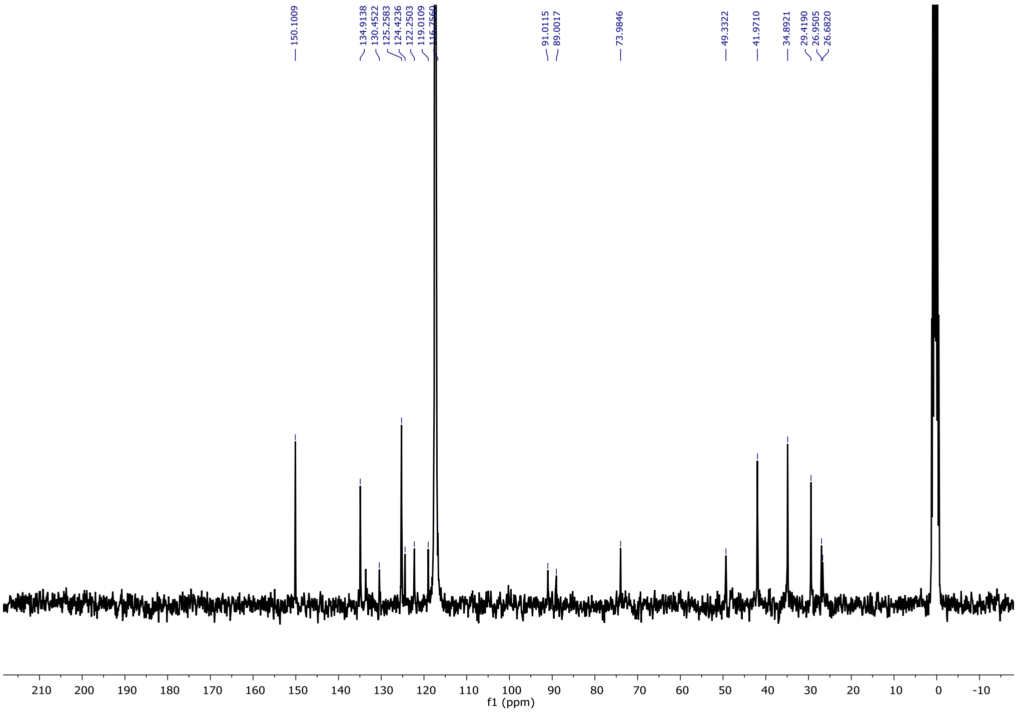

Figure S17. L^ImiAd building block, ^{13}C NMR in MeCN-d3.

Synthesis of $\mathbf{L^{PO}}$

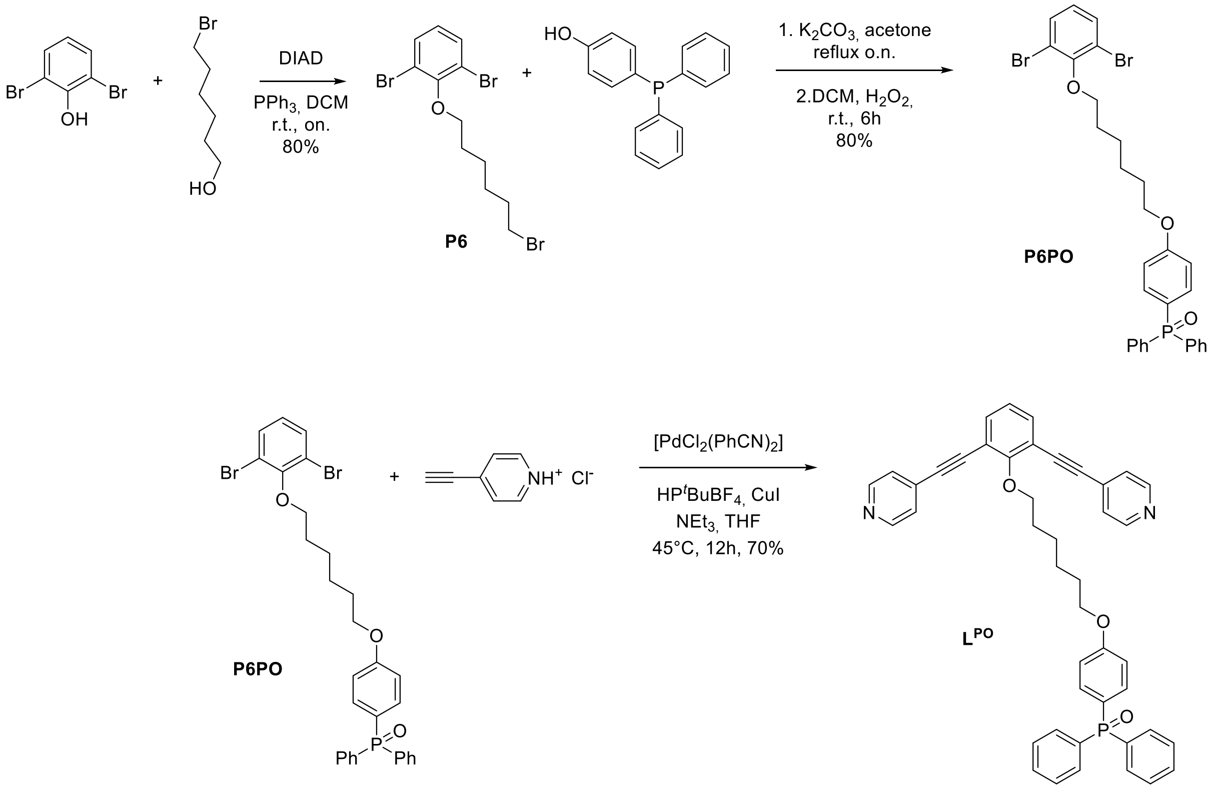

Scheme S6. Synthetic route for the C6-linked building block $\mathbf{L^{PO}}$.

Synthesis of intermediate **P6**

The material was synthesized according to an adopted literature procedure[5], but instead of using 4-methoxyphenol, 2,6-dibromophenol was used as staring material. To a solution of 2,6-dibromophenol (1.1g, 4.36 mmol, 1eq.), 6-bromo-hexa-1-ol (0.79 g, 4.36 mmol, 1 eq.) and PPh_3 (1.14 g, 4.36 mmol, 1 eq.) in 10 mL dry THF, DIAD (1 mL, 0.97g, 4.8 mmol, 1.1 eq.) was added dropwise at 0°C. The solution was let to heat up to room temperature and stirred overnight. The volatiles were removed under reduced pressure. The crude material was purified by column chromatography (SiO2, hexane : ethyl acetate, 99:1 to 90:10) to afford 1.4 g (3.5 mmol, 80%) of **P6** as a colorless oil. ^{1}H NMR (300 MHz, Chloroform-*d*) δ 7.52 (d, *J* = 8.0 Hz, 2H), 6.87 (t, *J* = 8.0 Hz, 1H), 4.04 (t, *J* = 6.4 Hz, 2H), 3.47 (t, *J* = 6.8 Hz, 2H), 2.02 – 1.82 (m, 5H), 1.70 – 1.49 (m, 5H). ^{13}C NMR (75 MHz, $CDCl_3$) δ 153.45, 132.72, 126.14, 118.55, 73.15, 33.91, 32.78, 29.86, 28.00, 25.15.

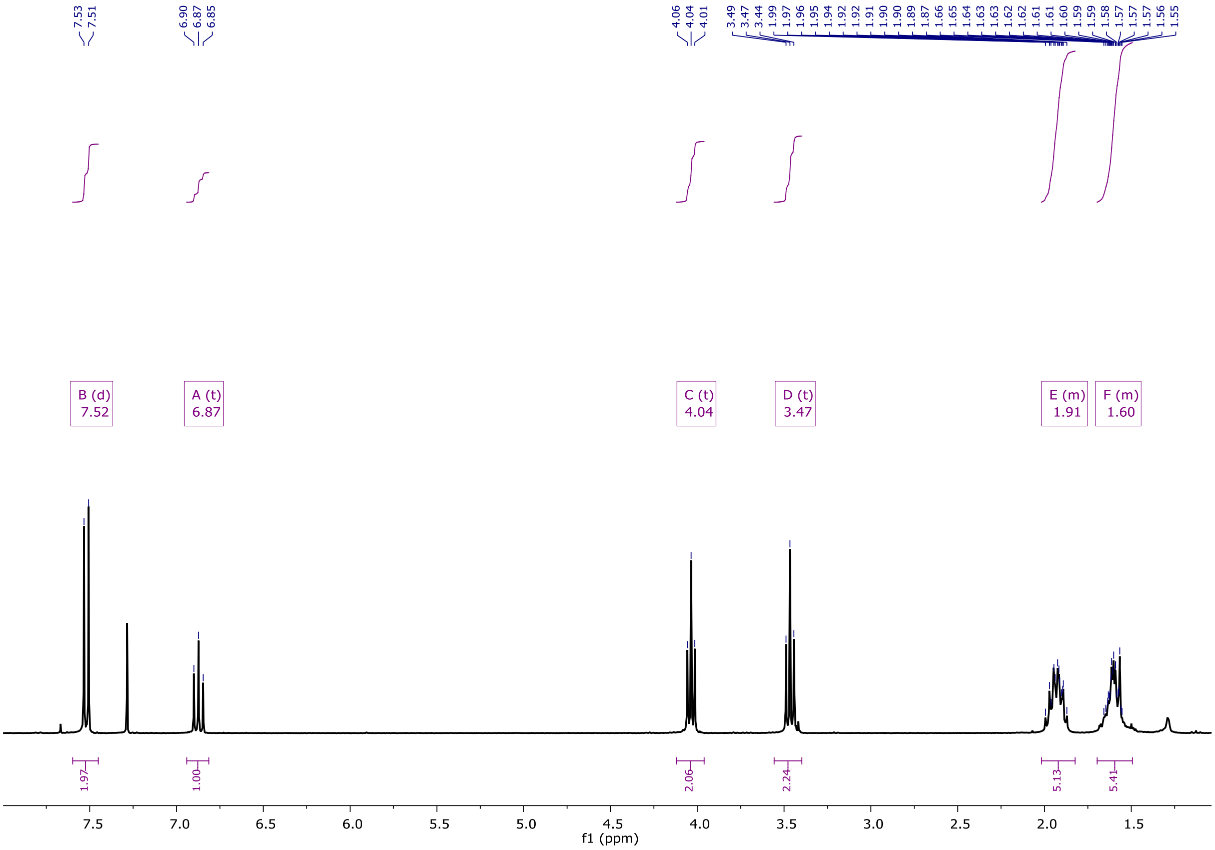

Figure S18. P6 precursor, ^{1}H NMR in $CDCl_3$.

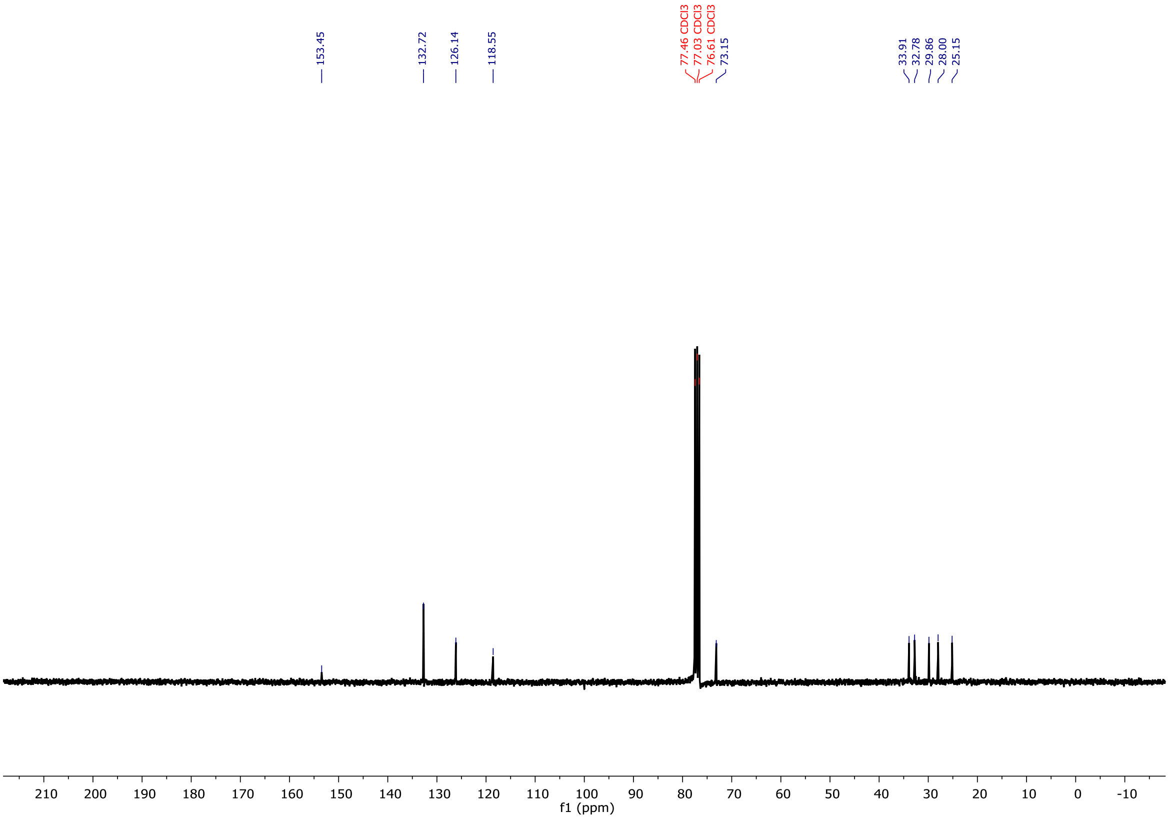

Figure S19. P6 precursor, ^{13}C NMR in $CDCl_3$.

Synthesis of intermediate **P6PO**

To a solution of **P6** (680 mg, 1.64 mmol, 1 eq.) in 50 mL acetone, 4-(diphenylphosphaneyl)phenol (510 mg, 1.8 mmol, 1.1 eq.) and K_2CO_3 (700 mg, 5 mmol, 3 eq.) were added. The solution was refluxed overnight. After evaporation of the solvent, the crude material was taken up into 100 mL DCM. The organic phase was washed with $NaOH_{aq}$ (1M) (5x50 mL). The organic phase was separated and the solvent evaporated. The crude material was used for the next step without any further purification. To a solution of **P6P** in 60 mL DCM, 30 mL of H_2O_2 (35% in water) was added. The solution was stirred for 6h at room temperature. Afterwards, the organic phase was separated, dried with Na_2SO_4 and the volatiles removed under reduced pressure to afford **P6PO** (920 mg, 91%) as a slowly crystalizing colorless oil. ^1H NMR (400 MHz, Chloroform-*d*) δ 7.72 – 7.65 (m, 4H), 7.62 – 7.53 (m, 3H), 7.51 (d, *J* = 8.0 Hz, 2H), 7.49 – 7.44 (m, 3H), 6.98 (dd, *J* = 8.9, 2.3 Hz, 2H), 6.87 (t, *J* = 8.0 Hz, 1H), 4.04 (td, *J* = 6.5, 2.3 Hz, 4H), 1.99 – 1.80 (m, 5H), 1.73 – 1.53 (m, 7H). ^{13}C NMR (101 MHz, $CDCl_3$) δ 134.06, 133.95, 132.71, 132.15, 132.05, 131.88, 128.53, 128.41, 126.14, 118.55, 114.67, 114.54, 73.19, 68.00, 53.44, 29.93, 29.06, 25.85, 25.66. ^{31}P NMR (162 MHz, $CDCl_3$) δ 29.78. HR-ESI-MS, calculated for $C_{30}H_{29}Br_2O_3P+H^+$ 629.0273, obtained 629.0355.

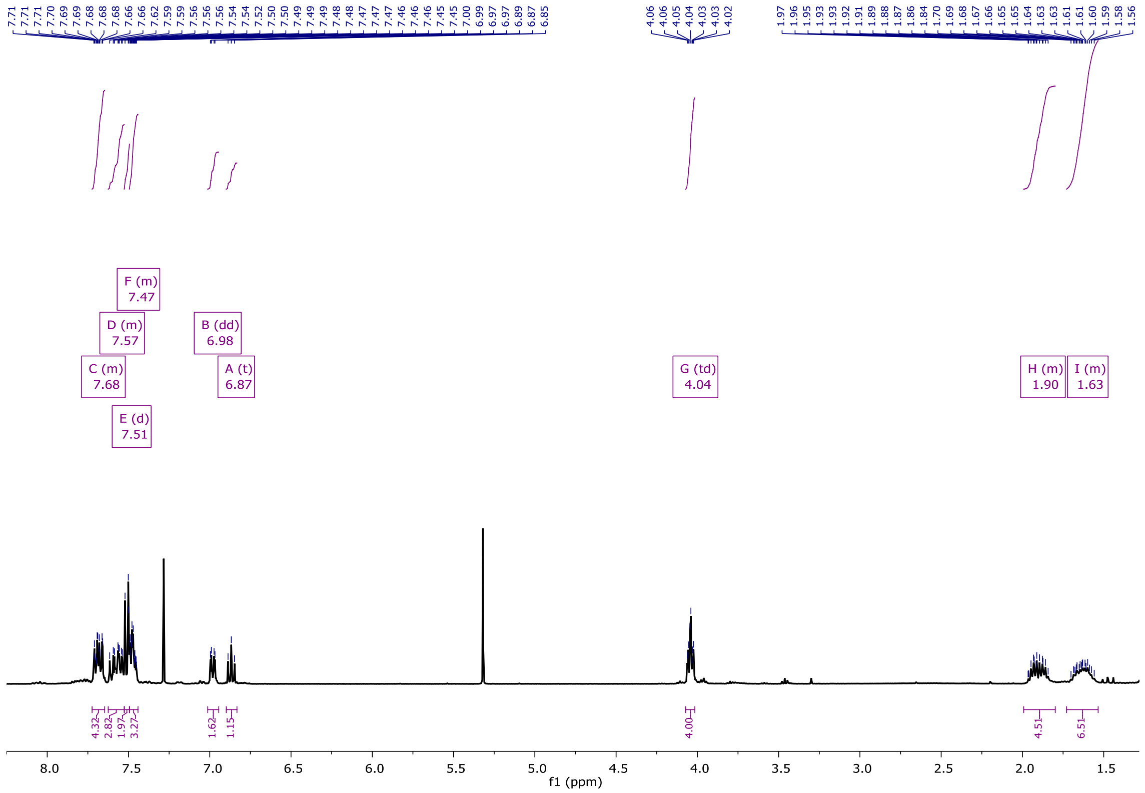

Figure S20. P6PO intermediate, ^1H NMR in $CDCl_3$.

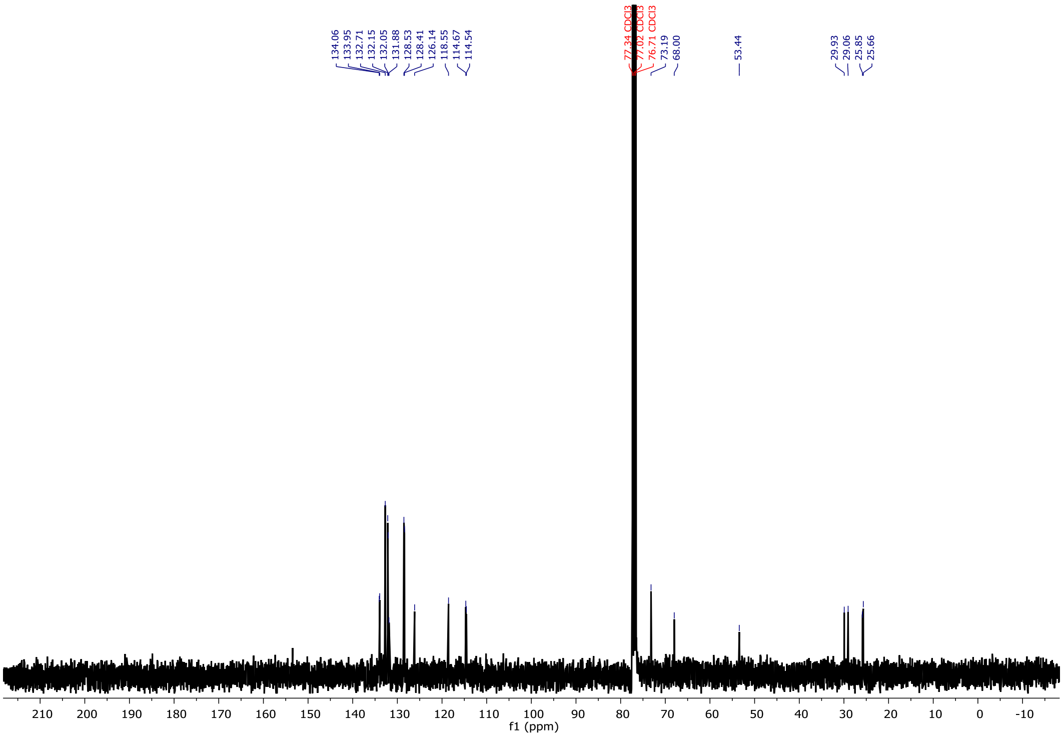

Figure S21. P6PO intermediate, ^{13}C NMR in $CDCl_3$.

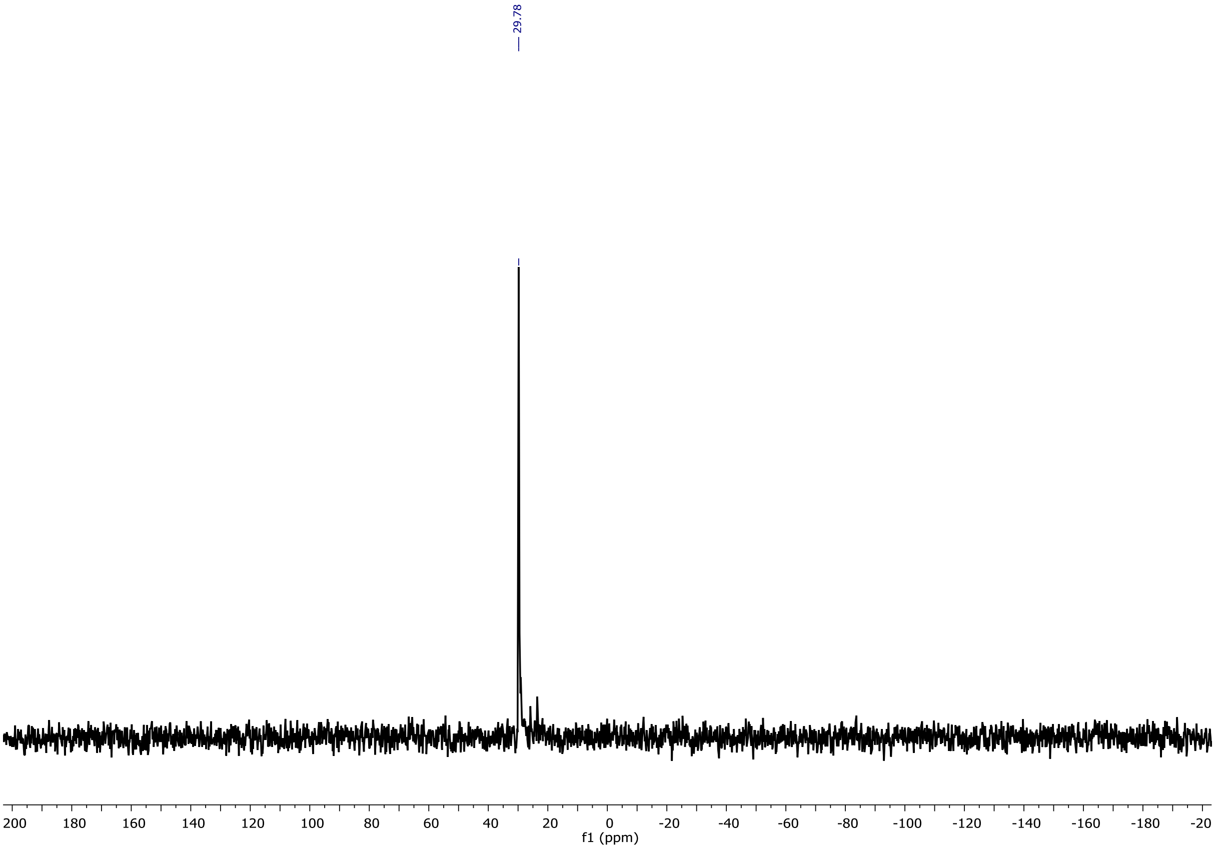

Figure S22. P6PO intermediate, ^{31}P NMR in $CDCl_3$.

Synthesis of **L^PO^**

HP^tBuBF_4 (60 mg, 0.18 mmol, 0.12 eq.) and $Pd(PhCN)_2Cl_2$ (40 mg, 0.09 mmol, 0.06 eq.) were dissolved in a mixture of degassed THF (20 mL) and trimethylamine (20 mL). The solution was stirred 20 min at room temperature before **P6PO** (0.92 g, 1.5 mmol, 1 eq.) and 4-ethynylpyridine hydrochloride (0.45 g, 3.73 mmol, 2.5 eq.) were added. The solution was gently heated up to 45 °C and copper(I) iodide (11 mg, 0.06 mmol, 0.04 eq.) was added. This mixture was stirred at 45 °C for 24 h under nitrogen atmosphere. The reaction mixture was poured into 400 mL ethylacetate, filtrated over a pad of sand and evaporated in *vacuo*. The crude product was purified by column chromatography on silica gel (DCM/MeOH = 99/1) to give **L^{PO}** as a slowly crystalizing colorless oil. ^{1}H NMR (400 MHz, Acetonitrile-d_3) δ 8.57 – 8.55 (m, 4H), 7.63 (ddd, *J* = 11.8, 8.3, 1.4 Hz, 4H), 7.60 – 7.54 (m, 4H), 7.53 – 7.46 (m, 6H), 7.42 – 7.38 (m, 4H), 7.13 (t, *J* = 7.7 Hz, 1H), 6.94 – 6.90 (m, 2H), 4.31 (t, *J* = 6.2 Hz, 2H), 3.89 (t, *J* = 6.5 Hz, 2H), 1.86 (dq, *J* = 8.1, 6.3 Hz, 2H), 1.73 – 1.63 (m, 2H), 1.63 – 1.55 (m, 2H), 1.52 – 1.45 (m, 1H). ^{13}C NMR (101 MHz, CD_3CN) δ 161.99, 161.96, 161.68, 149.99, 134.82, 134.10, 133.63, 133.52, 133.07, 131.85, 131.82, 131.74, 131.69, 131.59, 130.58, 128.65, 128.59, 128.54, 128.47, 125.20, 124.28, 123.96, 123.19, 116.64, 114.67, 114.54, 90.96, 89.34, 74.75, 67.90, 30.14, 28.69, 26.63, 25.76, 25.50. ^{31}P NMR (162 MHz, CD_3CN) δ 26.02. LogD(m^2/s) at 25°C (MeCN-d_3): -9.116 (d = 1.66 nm). HR-ESI-MS, calculated for $C_{44}H_{37}N_2O_3P+H^+$ 673.2615, obtained 673.2632.

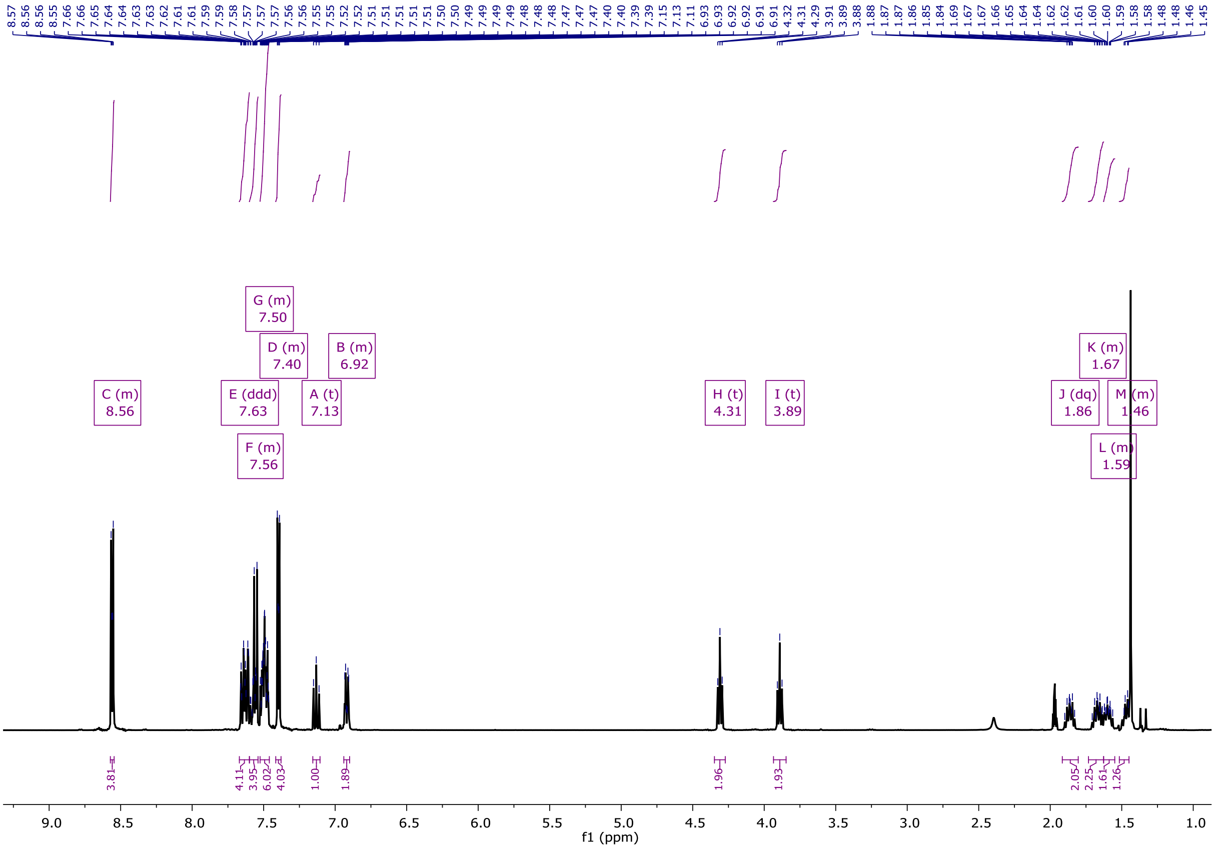

Figure S23. L^{PO} building block, ^{1}H NMR in MeCN-d_3.

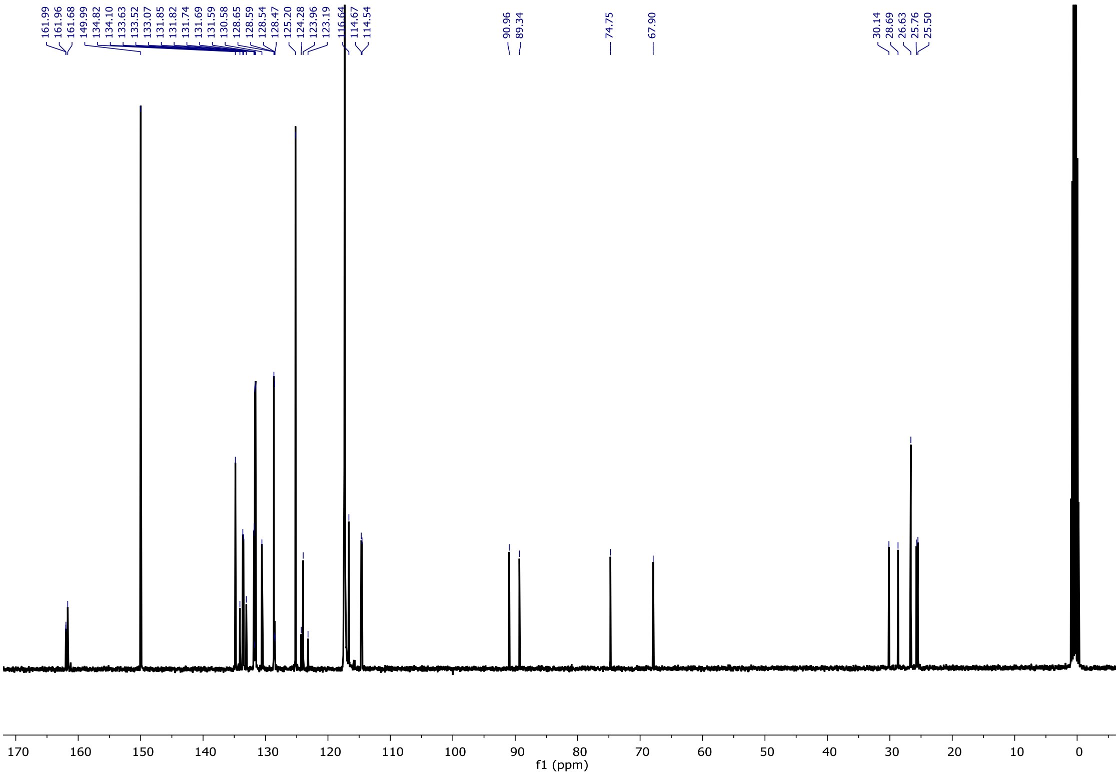

Figure S24. L[PO] building block, ^{13}C NMR in MeCN-d$_3$.

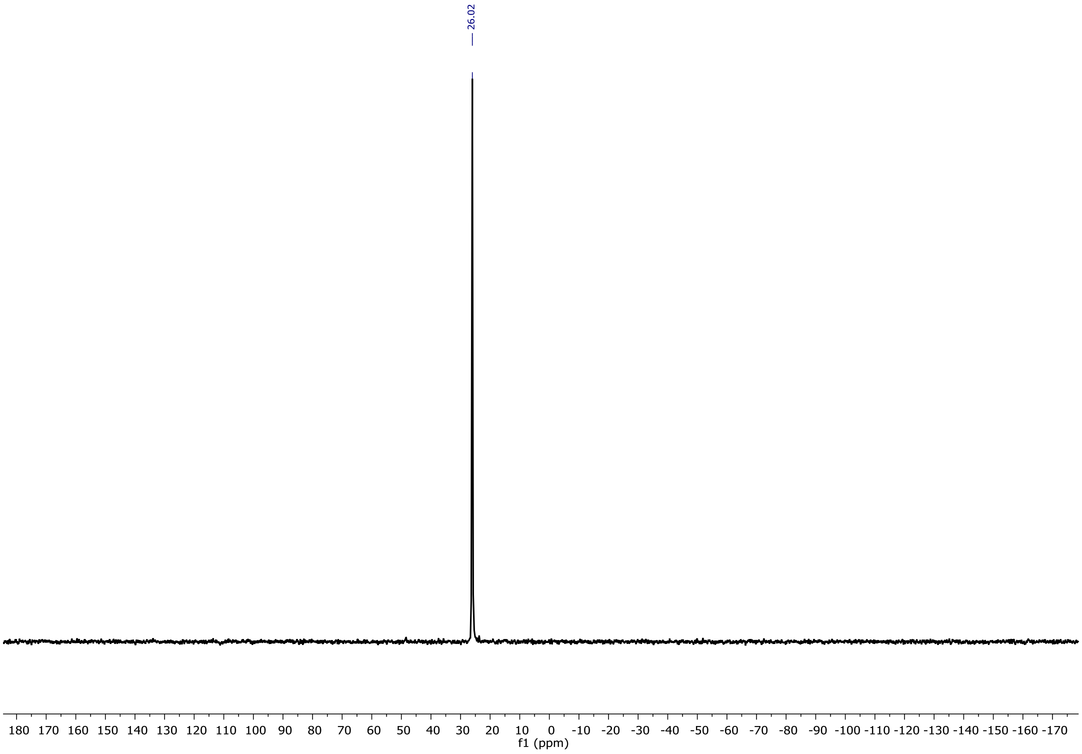

Figure S25. L[PO] building block, ^{31}P NMR in MeCN-d$_3$.

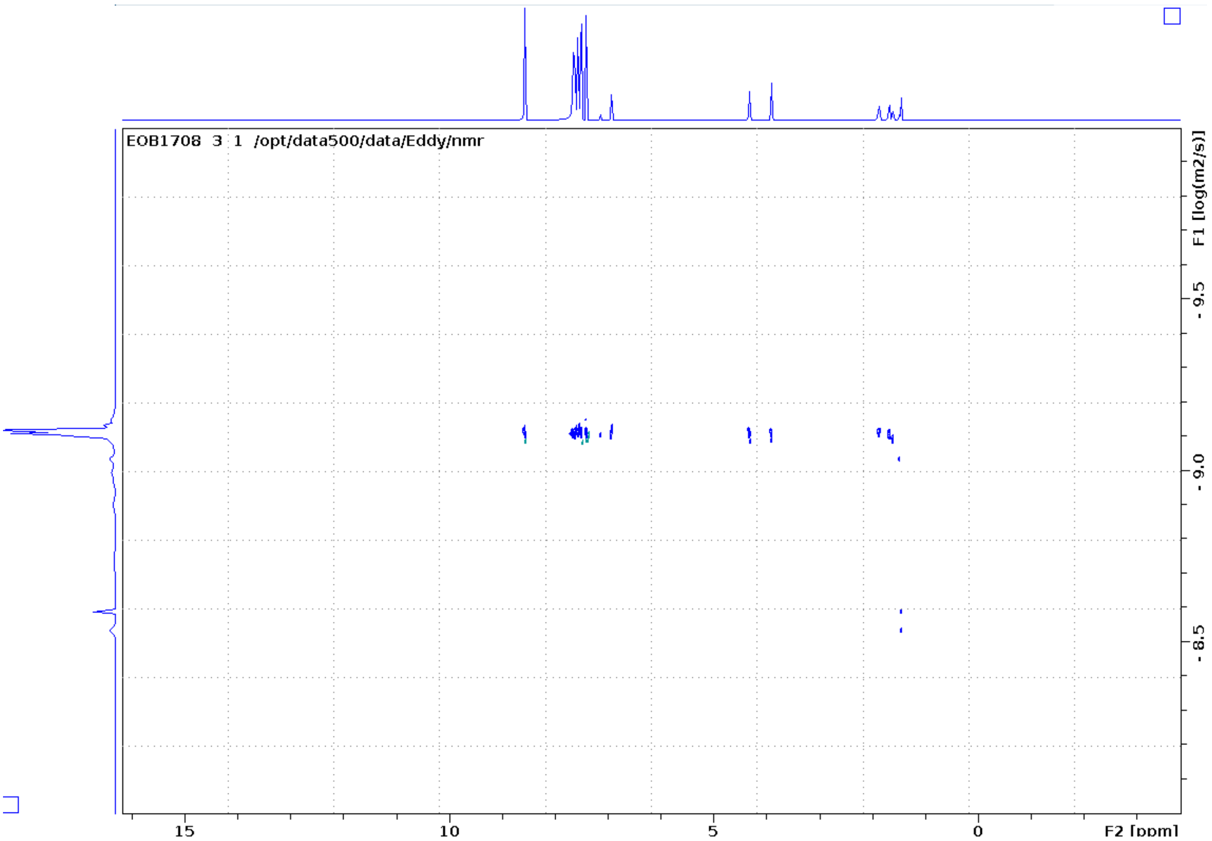

Figure S26. L^{PO} building block, DOSY in MeCN-d$_3$ at 25°C.

Synthesis of **L^{exoPy}**

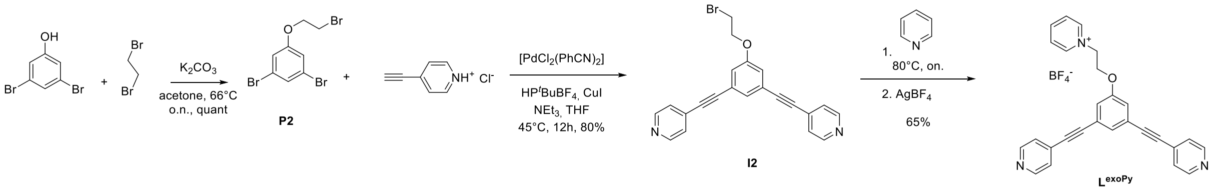

Scheme S7. Synthetic route for **L^{exoPy}**.

P2 was synthesized according to the procedure described before for **P4.** Briefly, 3,5-dibromophenol (5g, 19.84 mmol, 1 eq.) was refluxed with 1,2-dibromoethane (73 g, 397 mmol, 20 eq.) and K_2CO_3 (8.2 g, 60 mmol, 3 eq.) in 100 mL acetone overnight. Then, all volatiles were removed under reduced pressure at 90°C to afford the first intermediate **P2** in quantitative yield (7.1 g).

To a degassed solution of [Pd(PhCN)Cl_2] (126 mg, 0.33 mmol, 0.08 eq.) and HP^{t}BuBF$_4$ (190 mg, 0.66 mmol, 0.12 eq.) in 20 mL NEt_3 and 20 mL THF, **P2** (1.96 g, 5.5 mmol, 1 eq.) and 4-ethynylpyridine hydrochloride (2 g, 14.38 mmol, 2.5 eq.) were added. The mixture was heated to 45°C and CuI (42 mg, 0.22 mmol, 0.04 eq.) was added. After stirring the mixture at 45°C overnight, the solution was poured into 400 mL EtOAc and filtered over a pad of sand. The volatiles were removed under reduced pressure and the obtained

crude material was purified by column chromatography (SiO_2, MeOH:DCM, 1:99 -> 4:96) to afford **I2** as a colorless solid (1.77 g, 80%).^{1}H NMR (300 MHz, DMSO-d_6) δ 8.70 – 8.63 (m, 4H), 7.60 – 7.52 (m, 4H), 7.48 (t, J = 1.3 Hz, 1H), 7.34 (d, J = 1.3 Hz, 2H), 4.45 (dd, J = 6.2, 4.6 Hz, 2H), 3.84 (dd, J = 6.2, 4.5 Hz, 2H). ^{13}C NMR (75 MHz, DMSO) δ 158.52, 150.50, 130.19, 128.09, 125.88, 125.72, 123.62, 121.28, 119.54, 92.51, 87.91, 68.74, 31.63.

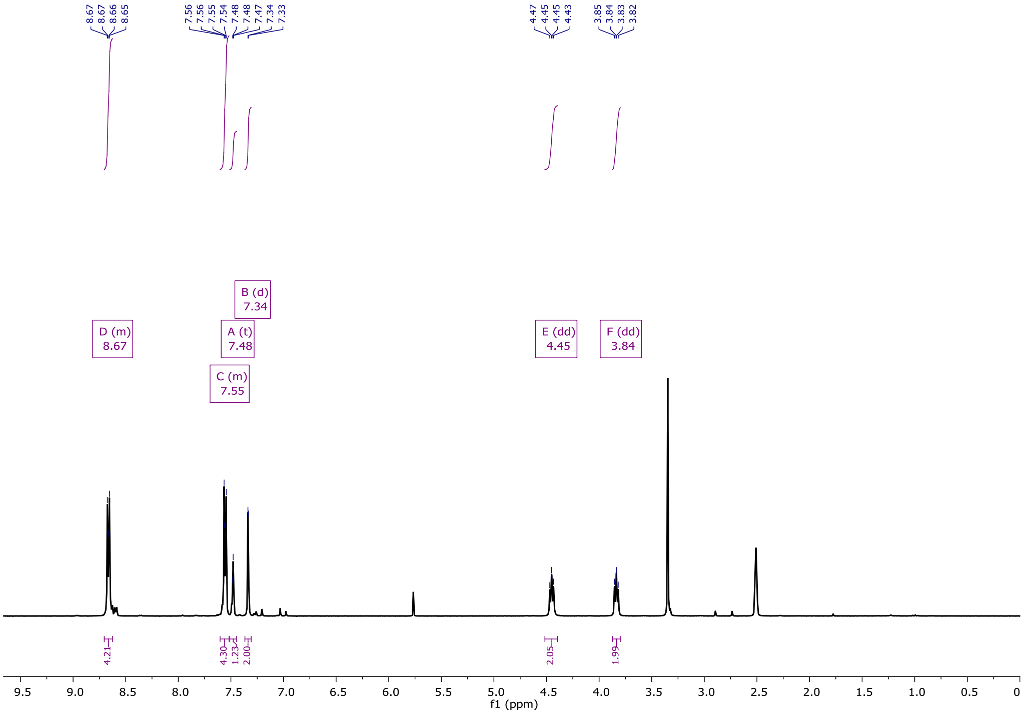

Figure S27. I2 intermediate, ^{1}H NMR in dmso-d_6.

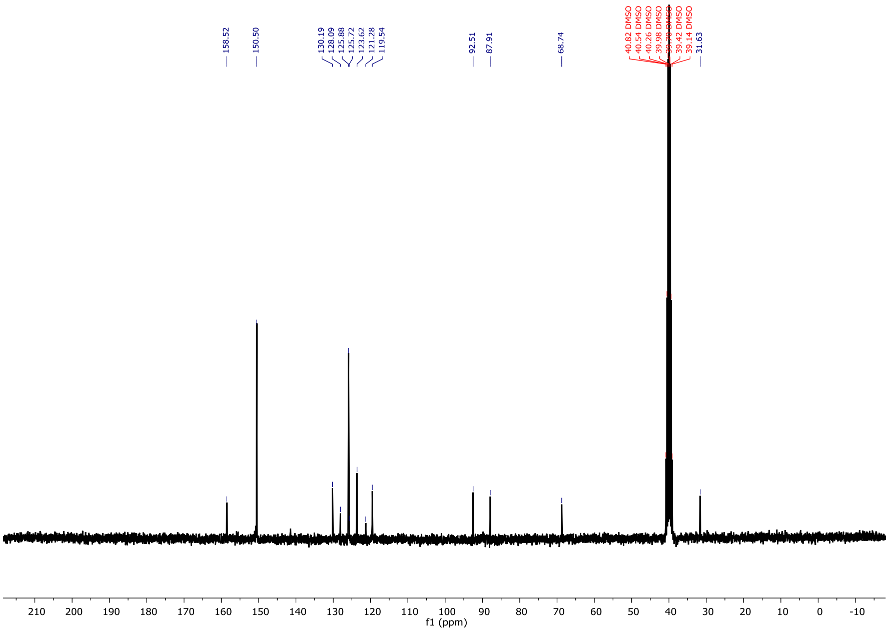

Figure S28. I2 intermediate, ^{13}C NMR in dmso-d_6.

L^{exoPy}: A solution of **I2** (0.2 g, 0.5 mmol, 1 eq.) in 10 mL pyridine was heated at 90°C for 24 h. The solvent was removed under reduced pressure. The resulting oil was dissolved in a minimum amount of DCM and precipitated in 1L Et_2O to afford the hydroscopic bromide salt. To a solution of this intermediate (230 mg, 0.47 mmol, 1 eq.) in 3 mL DCM and 3 mL MeCN, $AgBF_4$ (91 mg, 0.47 mmol, 1 eq.) were added in the dark. The suspension was stirred over night at room temperature. The solvent was removed under reduced pressure. The residue was dissolved in 5 mL DCM and filtered over a pad of celite. The volatiles were removed under reduced pressure to afford of **L^{exoPy}** (161 mg, 0.33 mmol, 70%) as a bright beige oil. ^{1}H NMR (400 MHz, Acetonitrile-*d*$_3$) δ 8.88 – 8.83 (m, 2H), 8.66 – 8.62 (m, 4H), 8.59 (ddt, *J* = 9.2, 7.9, 1.5 Hz, 2H), 8.15 – 8.06 (m, 2H), 7.51 – 7.45 (m, 4H), 7.44 (s, 1H), 7.23 (d, *J* = 1.3 Hz, 2H), 5.05 – 4.94 (m, 2H), 4.58 – 4.50 (m, 2H). ^{13}C NMR (101 MHz, CD_3CN) δ 158.24, 151.00, 150.66, 150.59, 147.00, 145.87, 141.22, 130.95, 128.87, 128.82, 126.02, 125.97, 124.35, 121.39, 119.41, 112.67, 92.18, 87.85, 66.94, 61.25. HR-ESI-MS, calculated for $C_{27}H_{20}N_3O$ 402.1601, obtained 402.1600.

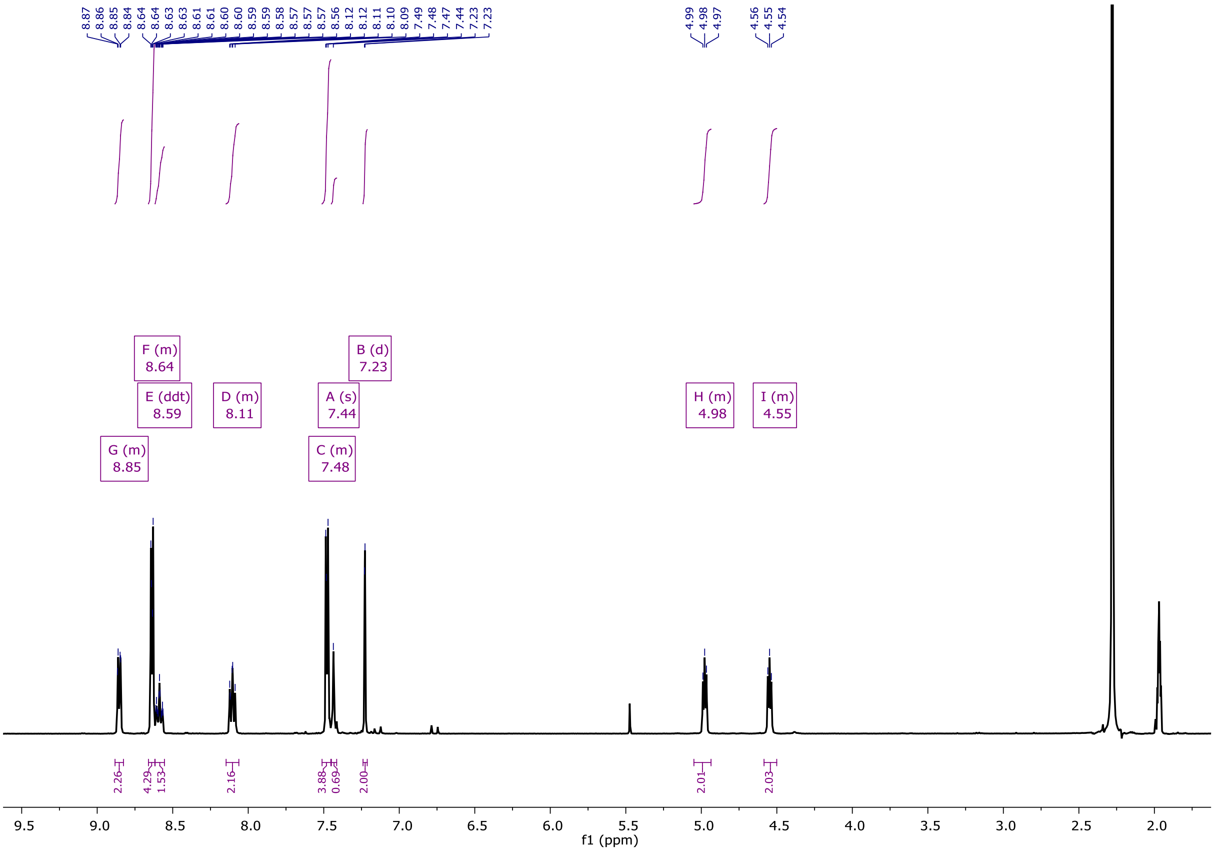

Figure S29. **L^{exoPy}** building block, ^{1}H NMR in MeCN-d$_3$.

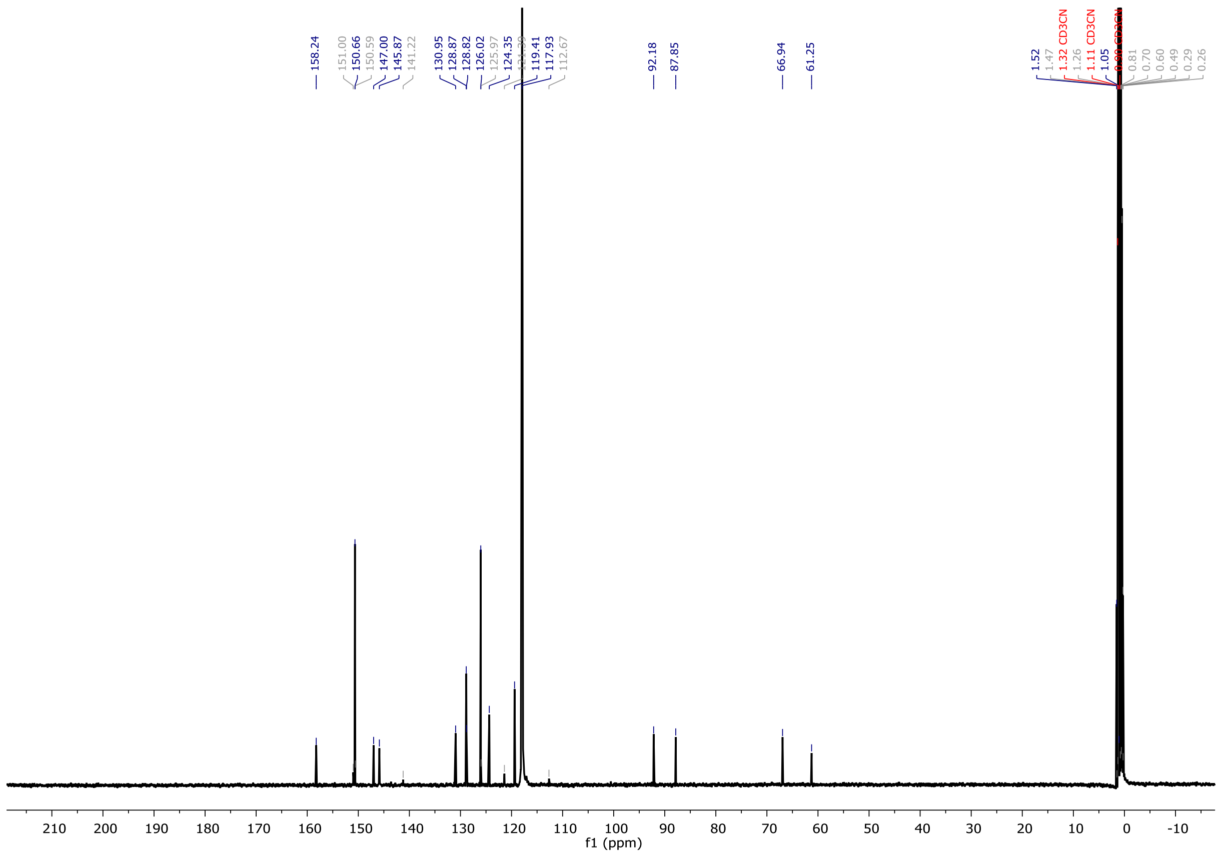

Figure S30. L^{exoPy} building block, ^{13}C NMR in MeCN-d$_3$.

Sphere Synthesis (SI2)

All spheres were prepared as 0.41mM solutions in 1 mL dry and degassed acetonitrile at noted temperature and time in 10 mL high pressure tubes. The tubes were heated using an oil bath with the oil level not exceeding the solvent level. The experimental setup for sphere formation is depicted herewith:

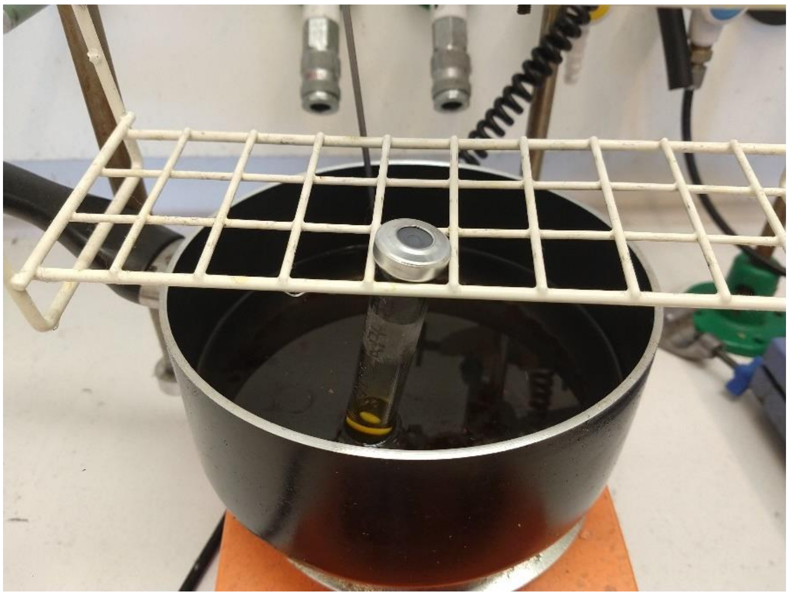

[Pt_n(**L^{OMe}**)$_{2n}$]$^{2n+}$(BF_4^-)$_{2n}$

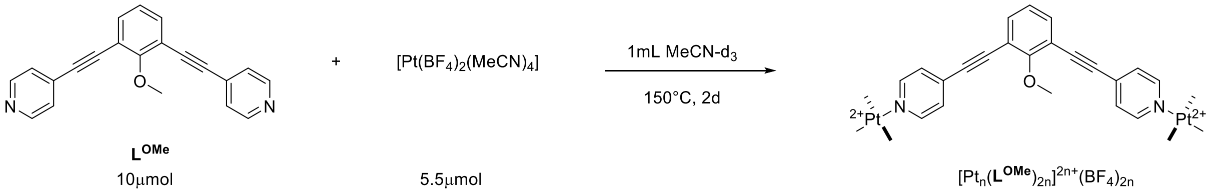

To a solution of **L^{OMe}** (3.1 mg, 10 μmol, 1 eq.) in 0.5 mL MeCN-d_3, [Pt(BF_4)$_2$(MeCN)$_4$] (2.93 mg, 5.5 μmol. 0.55 eq.) in 0.5 mL MeCN-d_3 was added. The solution was then stirred at 150°C for 2d in a 10 mL high pressure tube. ^{1}H NMR (400 MHz, Acetonitrile-d_3) δ 8.95 (s, 4H), 7.68 (s, 6H), 7.35 – 7.13 (m, 1H), 4.14 (s, 3H). LogD (m^2/s) at 25°C (MeCN-d_3): -9.565 (d = 4,67 nm).

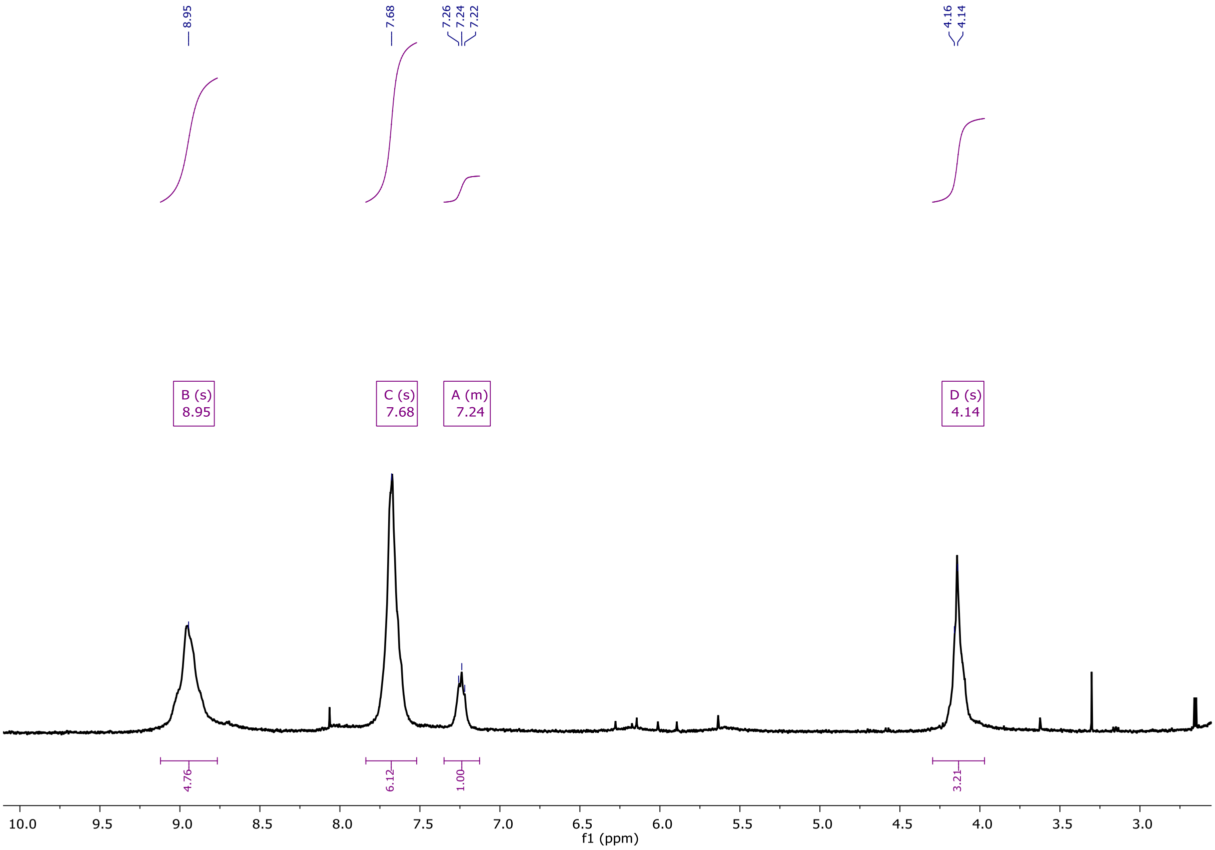

Figure S31. $[Pt_n(\mathbf{L^{OMe}})_{2n}]^{2n+}(BF_4^-)_{2n}$ sphere, ^1H NMR in MeCN-d_3.

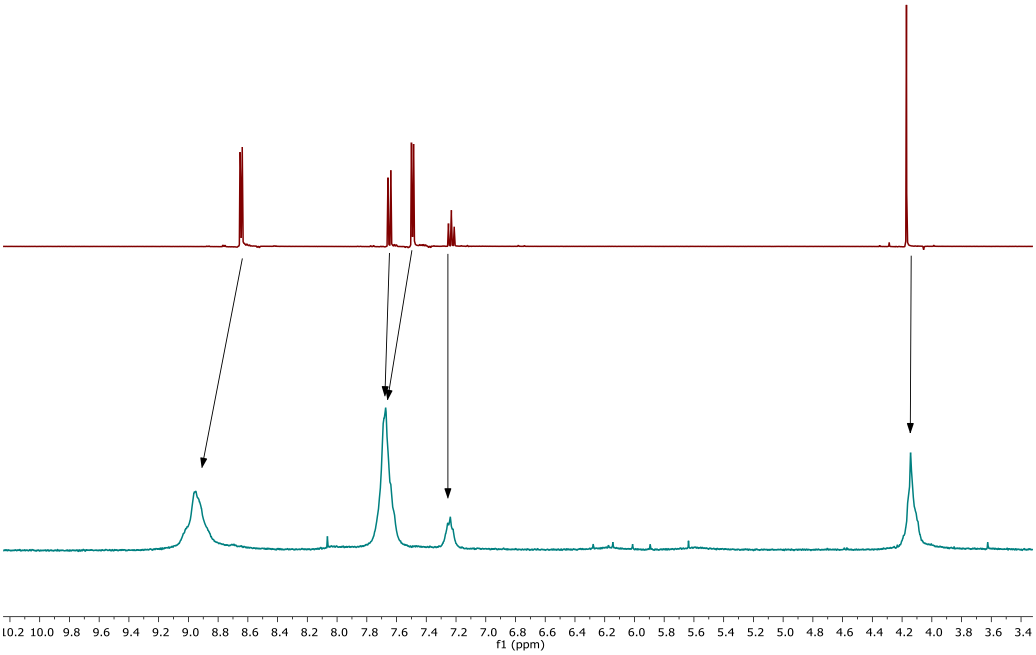

Figure S32. Comparison of free $\mathbf{L^{OMe}}$ building block (top) and $[Pt_n(\mathbf{L^{OMe}})_{2n}]^{2n+}(BF4^-)_{2n}$ sphere (bottom), ^1H NMR in MeCN-d_3.

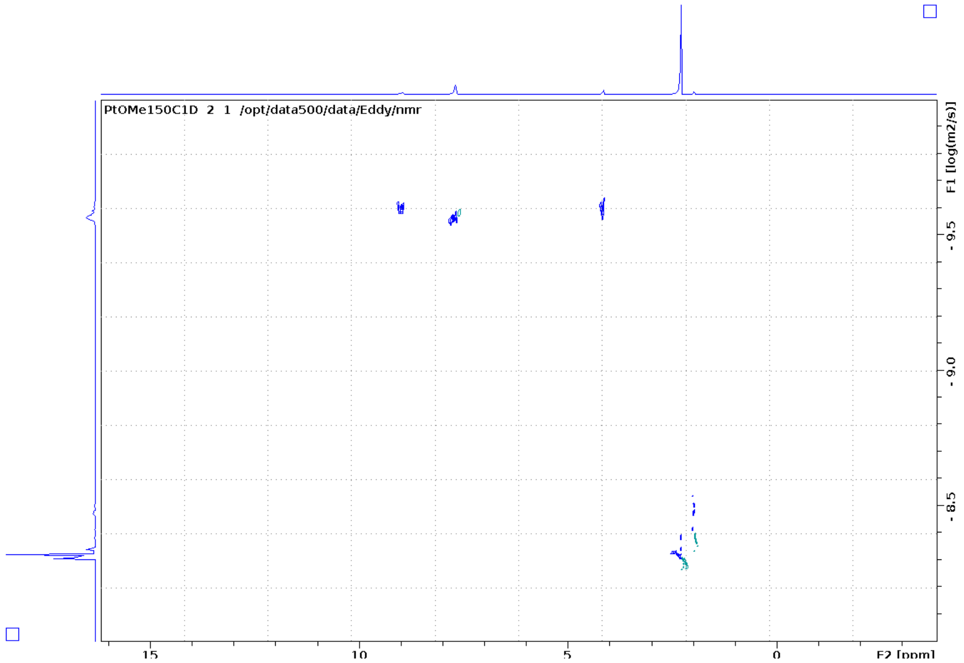

Figure S33. $[Pt_n(\mathbf{L}^{OMe})_{2n}]^{2n+}(BF4^-)_{2n}$ cage prepared at 150°C, DOSY in MeCN-d3 at 25°C.

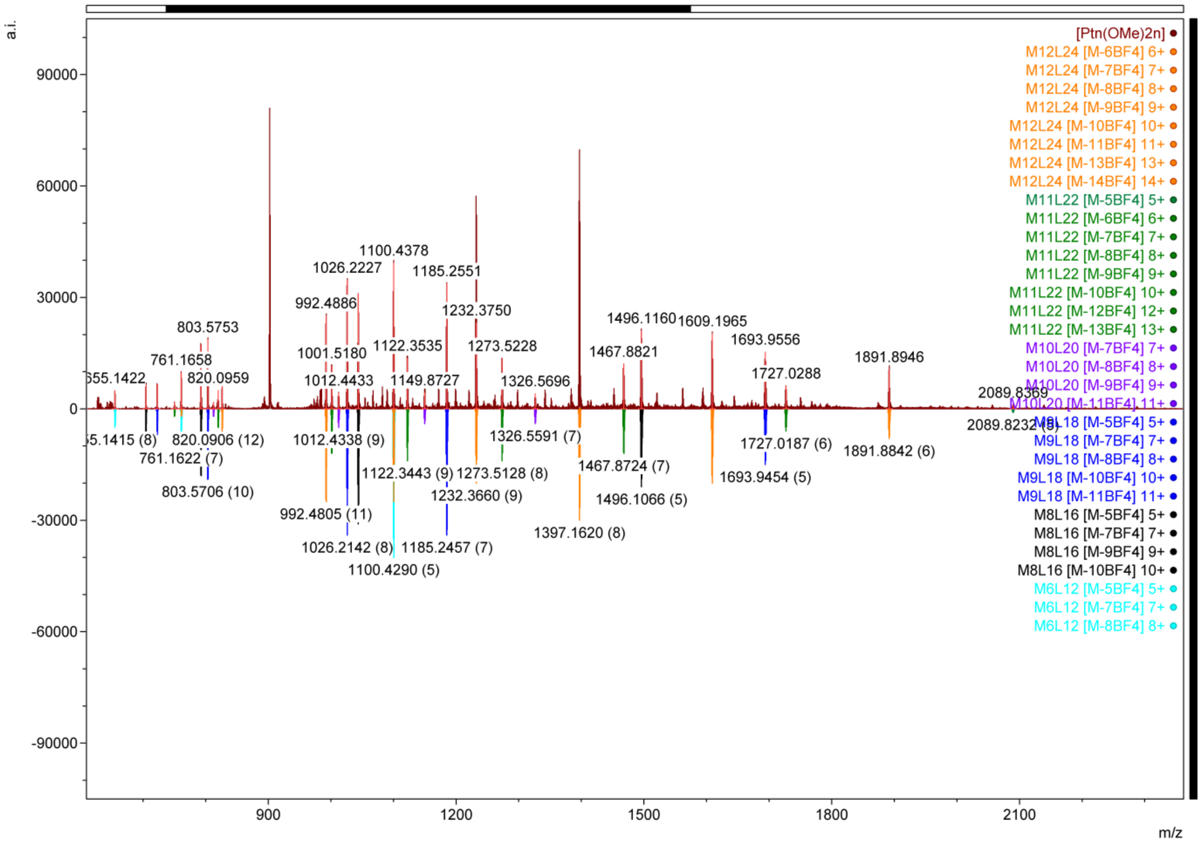

Figure S34. Full ESI-MS spectra of $[Pt_n(\mathbf{L}^{OMe})_{2n}]^{2n+}(BF4^-)_{2n}$ prepared at 110°C for 3d. Below the simulated spectra of different sphere types, above obtained spectra.

Table S1. Calculated and observed species of the $[Pt_n(\mathbf{L^{OMe}})_{2n}]^{2n+}$ assemblies of a sample prepared at 110°C for 3d.

Cage Type	Composition	Calculated	Found
M_6L_{12}	$[Pt_6(\mathbf{L^{OMe}})_{12}(BF_4^-)_4]^{8+}$	655.1415	655.1422
	$[Pt_6(\mathbf{L^{OMe}})_{12}(BF_4^-)_5]^{7+}$	761.1622	761.1658
	$[Pt_6(\mathbf{L^{OMe}})_{12}(BF_4^-)_7]^{5+}$	1100.4290	1100.4378
M_7L_{14}	$[Pt_7(\mathbf{L^{OMe}})_{14}(BF_4^-)_8]^{6+}$	1067.3890	1067.3957
	$[Pt_7(\mathbf{L^{OMe}})_{14}(BF_4^-)_9]^{5+}$	1298.2678	1298.2773
	$[Pt_7(\mathbf{L^{OMe}})_{14}(BF_4^-)_{10}]^{4+}$	1644.5858	1644.5965
M_8L_{16}	$[Pt_8(\mathbf{L^{OMe}})_{16}(BF_4^-)_6]^{10+}$	704.6512	704.6535
	$[Pt_8(\mathbf{L^{OMe}})_{16}(BF_4^-)_7]^{9+}$	792.6129	792.6177
	$[Pt_8(\mathbf{L^{OMe}})_{16}(BF_4^-)_9]^{7+}$	1043.7892	1043.7972
	$[Pt_8(\mathbf{L^{OMe}})_{16}(BF_4^-)_{11}]^{5+}$	1496.1066	1496.1160
M_9L_{18}	$[Pt_9(\mathbf{L^{OMe}})_{18}(BF_4^-)_7]^{11+}$	722.6092	722.6121
	$[Pt_9(\mathbf{L^{OMe}})_{18}(BF_4^-)_8]^{10+}$	803.5706	803.5753
	$[Pt_9(\mathbf{L^{OMe}})_{18}(BF_4^-)_{10}]^{8+}$	1026.2142	1026.2227
	$[Pt_9(\mathbf{L^{OMe}})_{18}(BF_4^-)_{11}]^{7+}$	1185.2457	1185.2551
	$[Pt_9(\mathbf{L^{OMe}})_{18}(BF_4^-)_{13}]^{5+}$	1693.9454	1693.9556
$M_{10}L_{20}$	$[Pt_{10}(\mathbf{L^{OMe}})_{20}(BF_4^-)_9]^{11+}$	812.6270	812.6319
	$[Pt_{10}(\mathbf{L^{OMe}})_{20}(BF_4^-)_{11}]^{9+}$	1012.4338	1012.4433
	$[Pt_{10}(\mathbf{L^{OMe}})_{20}(BF_4^-)_{12}]^{8+}$	1149.8637	1149.8727
	$[Pt_{10}(\mathbf{L^{OMe}})_{20}(BF_4^-)_{13}]^{7+}$	1326.5591	1326.5696
$M_{11}L_{22}$	$[Pt_{11}(\mathbf{L^{OMe}})_{22}(BF_4^-)_9]^{13+}$	750.3140	750.3177
	$[Pt_{11}(\mathbf{L^{OMe}})_{22}(BF_4^-)_{10}]^{12+}$	820.0906	820.0959
	$[Pt_{11}(\mathbf{L^{OMe}})_{22}(BF_4^-)_{12}]^{10+}$	1001.5096	1001.5180
	$[Pt_{11}(\mathbf{L^{OMe}})_{22}(BF_4^-)_{13}]^{9+}$	1122.3443	1122.3535
	$[Pt_{11}(\mathbf{L^{OMe}})_{22}(BF_4^-)_{14}]^{8+}$	1273.5128	1273.5228
	$[Pt_{11}(\mathbf{L^{OMe}})_{22}(BF_4^-)_{15}]^{7+}$	1467.8724	1467.8821
	$[Pt_{11}(\mathbf{L^{OMe}})_{22}(BF_4^-)_{16}]^{6+}$	1727.0187	1727.0288
	$[Pt_{11}(\mathbf{L^{OMe}})_{22}(BF_4^-)_{17}]^{5+}$	2089.8232	2089.8369
$M_{12}L_{24}$	$[Pt_{12}(\mathbf{L^{OMe}})_{24}(BF_4^-)_{10}]^{14+}$	761.2338	761.2381
	$[Pt_{12}(\mathbf{L^{OMe}})_{24}(BF_4^-)_{11}]^{13+}$	826.4059	826.4113
	$[Pt_{12}(\mathbf{L^{OMe}})_{24}(BF_4^-)_{13}]^{11+}$	992.4805	992.4886
	$[Pt_{12}(\mathbf{L^{OMe}})_{24}(BF_4^-)_{14}]^{10+}$	1100.4290	1100.4378
	$[Pt_{12}(\mathbf{L^{OMe}})_{24}(BF_4^-)_{15}]^{9+}$	1232.3660	1232.3750
	$[Pt_{12}(\mathbf{L^{OMe}})_{24}(BF_4^-)_{17}]^{7+}$	1609.1860	1609.1965
	$[Pt_{12}(\mathbf{L^{OMe}})_{24}(BF_4^-)_{18}]^{6+}$	1891.8842	1891.8946

Table S2. Zoom into different charged species of $[Pt_n(\mathbf{L}^{OMe})_{2n}]^{2n+}$ assemblies of a sample prepared at 110°C for 3d.

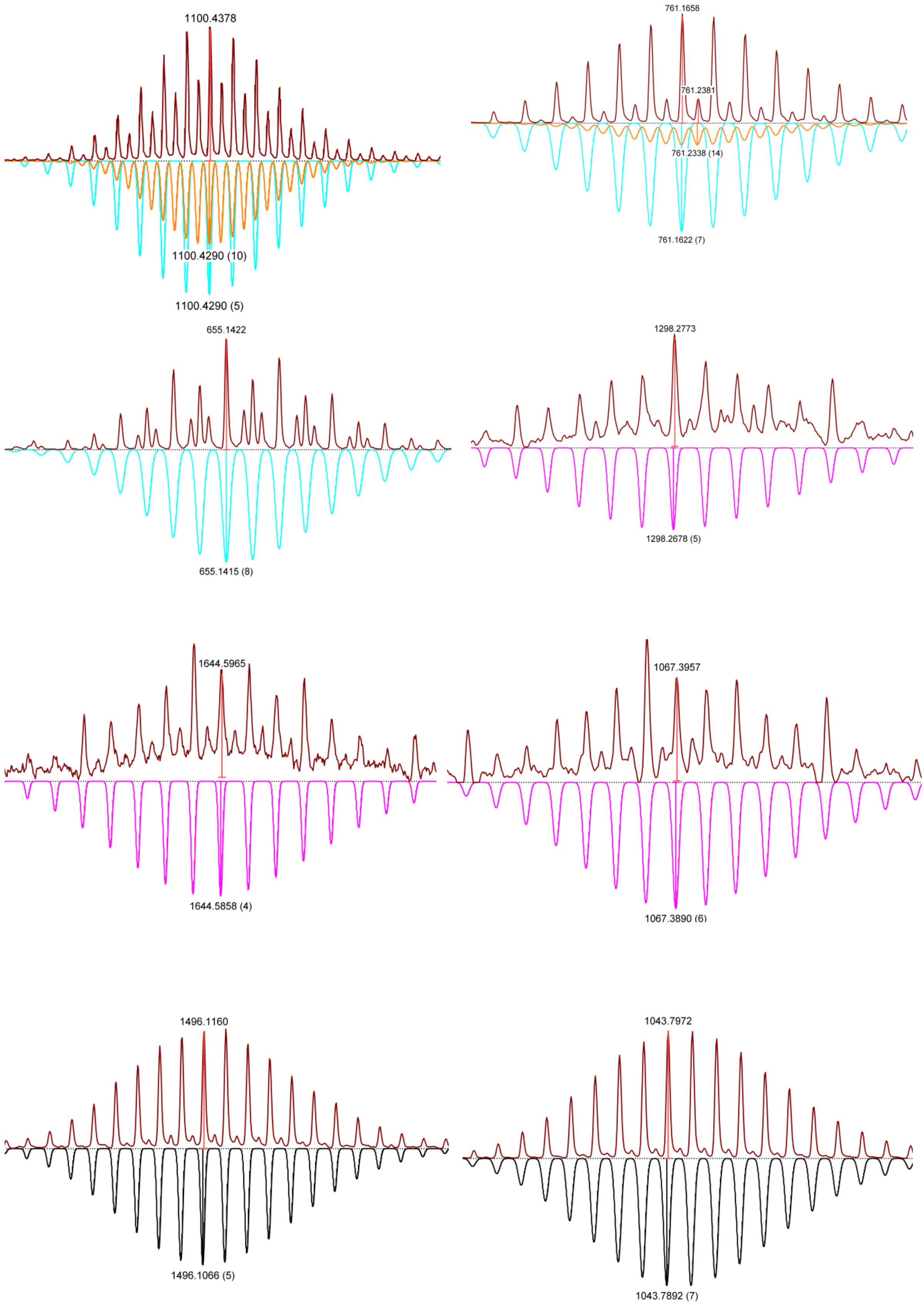

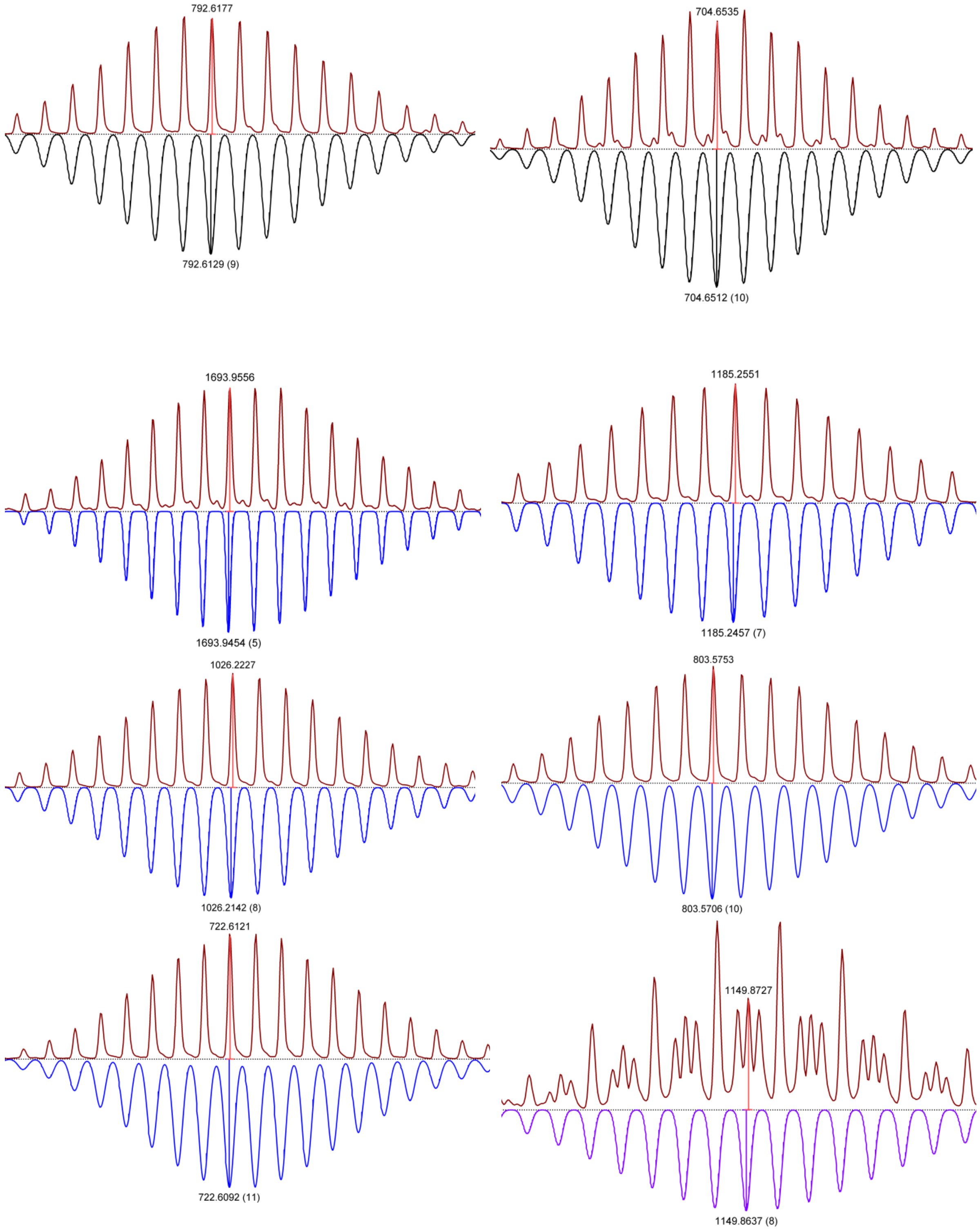
792.6177
792.6129 (9)
704.6535
704.6512 (10)
1693.9556
1693.9454 (5)
1185.2551
1185.2457 (7)
1026.2227
1026.2142 (8)
803.5753
803.5706 (10)
722.6121
722.6092 (11)
1149.8727
1149.8637 (8)

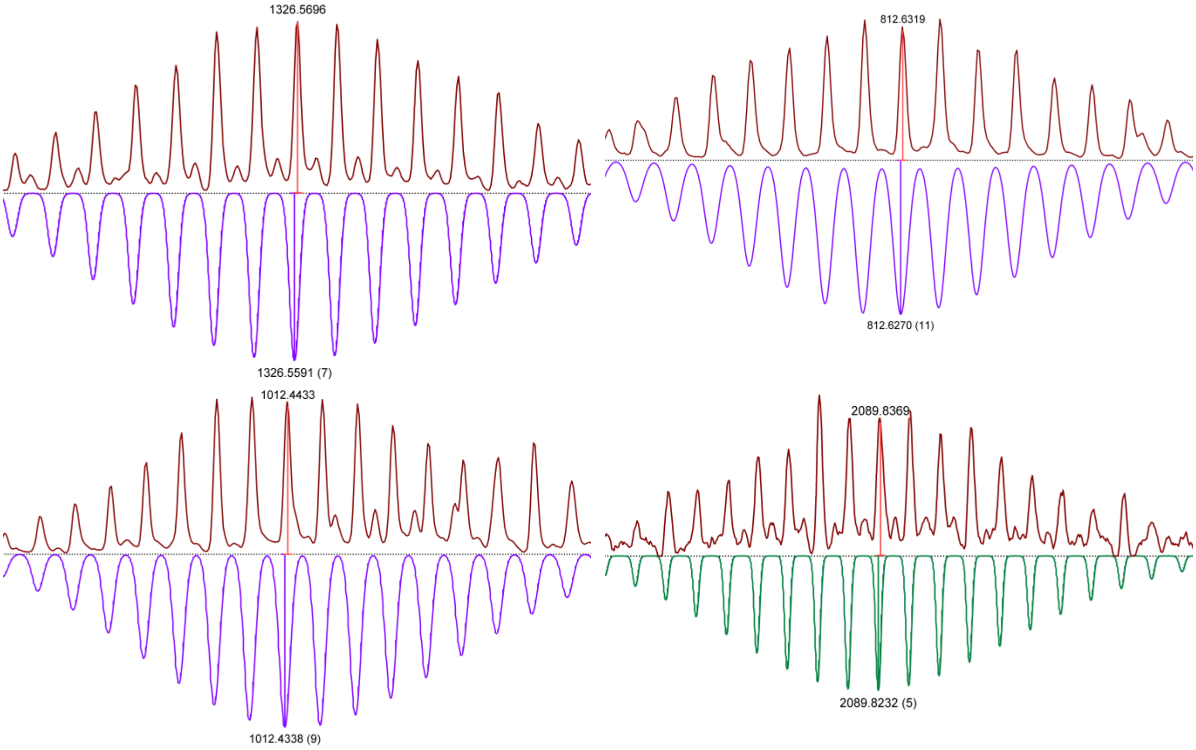
1326.5696
1326.5591 (7)
812.6319
812.6270 (11)
1012.4433
1012.4338 (9)
2089.8369
2089.8232 (5)

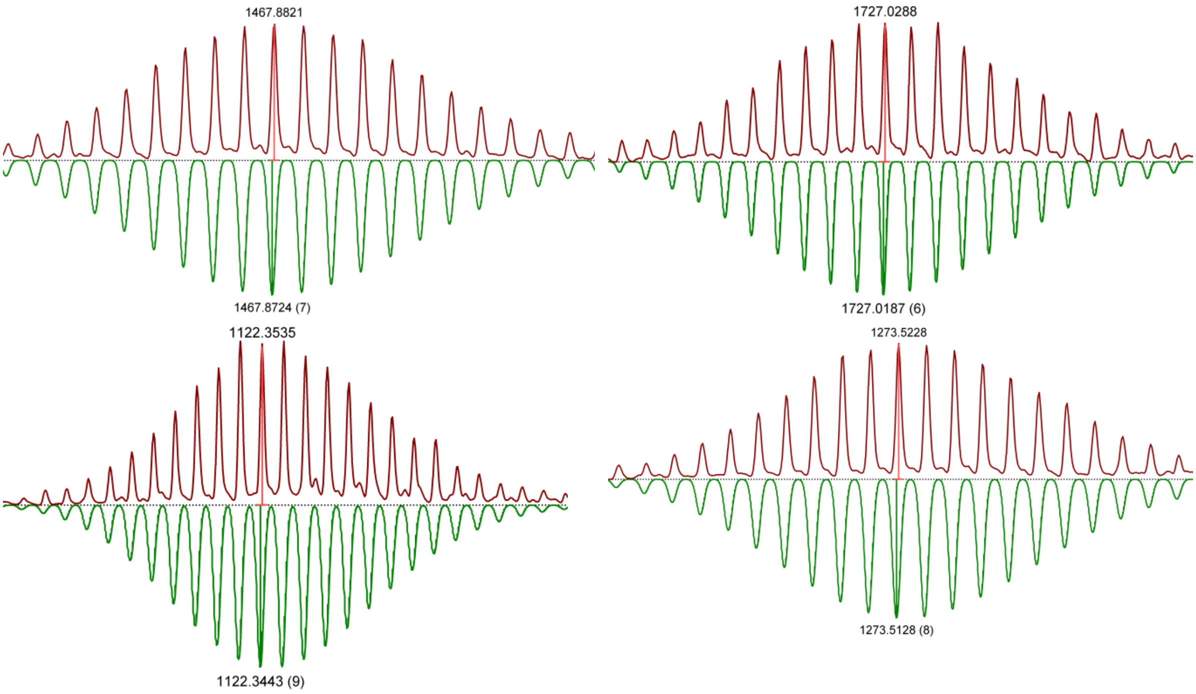
1467.8821
1467.8724 (7)
1727.0288
1727.0187 (6)
1122.3535
1122.3443 (9)
1273.5228
1273.5128 (8)

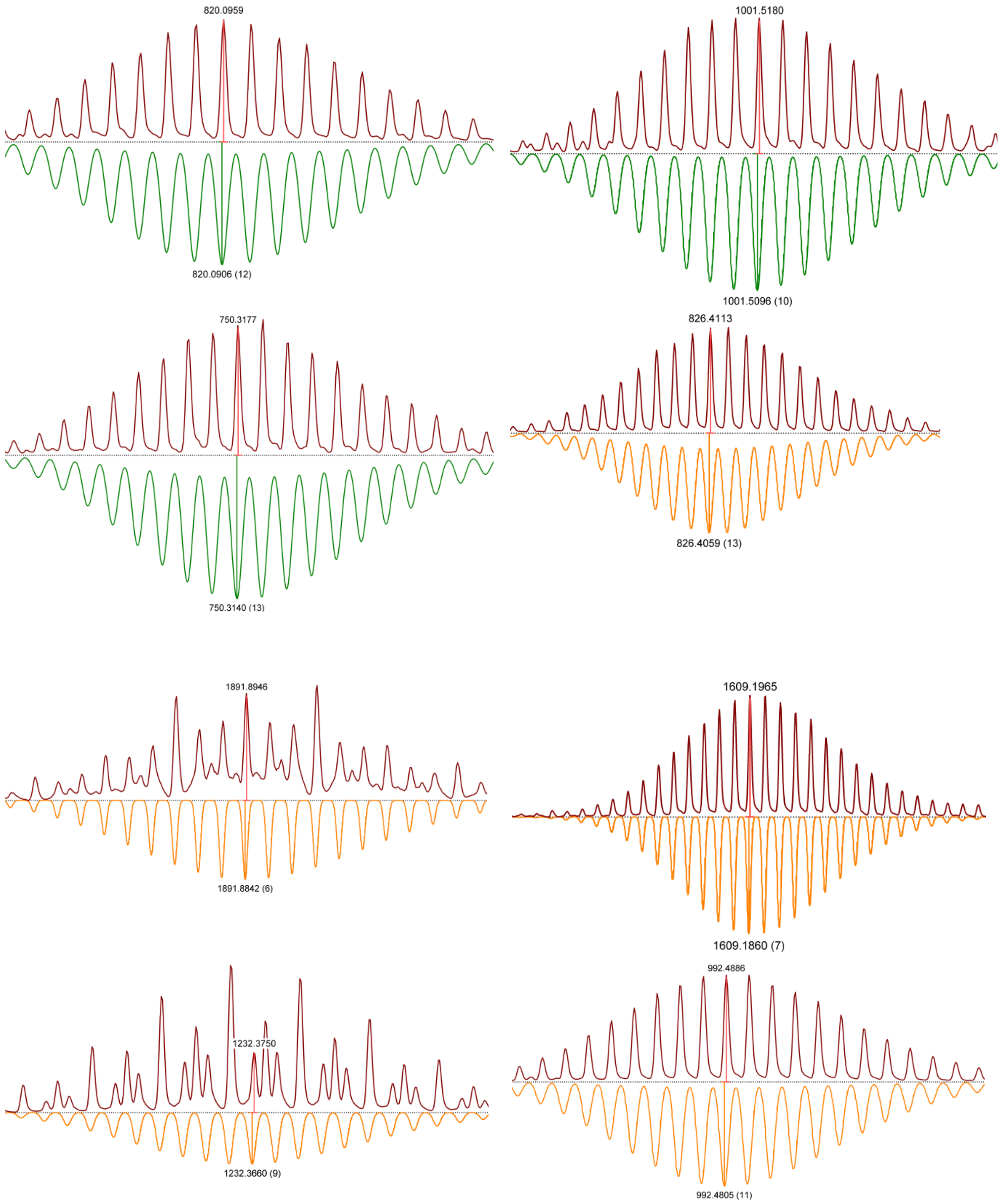

820.0959
820.0906 (12)
1001.5180
1001.5096 (10)
750.3177
750.3140 (13)
826.4113
826.4059 (13)
1891.8946
1891.8842 (6)
1609.1965
1609.1860 (7)
1232.3750
1232.3660 (9)
992.4886
992.4805 (11)

[Pt$_n$($\mathbf{L^{OBn}}$)$_{2n}$]$^{2n+}$(BF_4^-)$_{2n}$

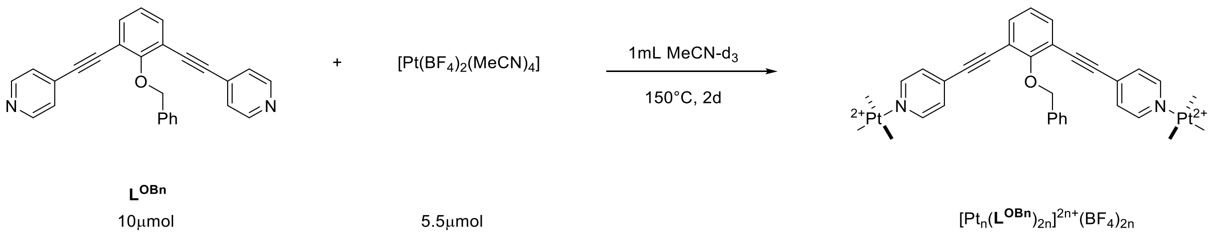

To a solution of $\mathbf{L^{OBn}}$ (3.86 mg, 10 μmol, 1 eq.) in 0.5 mL MeCN-d$_3$, [Pt(BF_4)$_2$(MeCN)$_4$] (2.93 mg, 5.5 μmol. 0.55 eq.) in 0.5 mL MeCN-d$_3$ was added. The solution was then stirred at 150°C for 2d in a 10 mL high pressure tube. ^{1}H NMR (400 MHz, Acetonitrile-d_3) δ 8.87 (d, *J* = 37.4 Hz, 4H), 7.88 – 7.60 (m, 1H), 7.60 – 7.32 (m, 5H), 7.32 – 7.11 (m, 2H), 5.52 – 5.30 (m, 2H). LogD(m^2/s) at 25°C (MeCN-d$_3$): -9.433 (d = 3.45 nm).

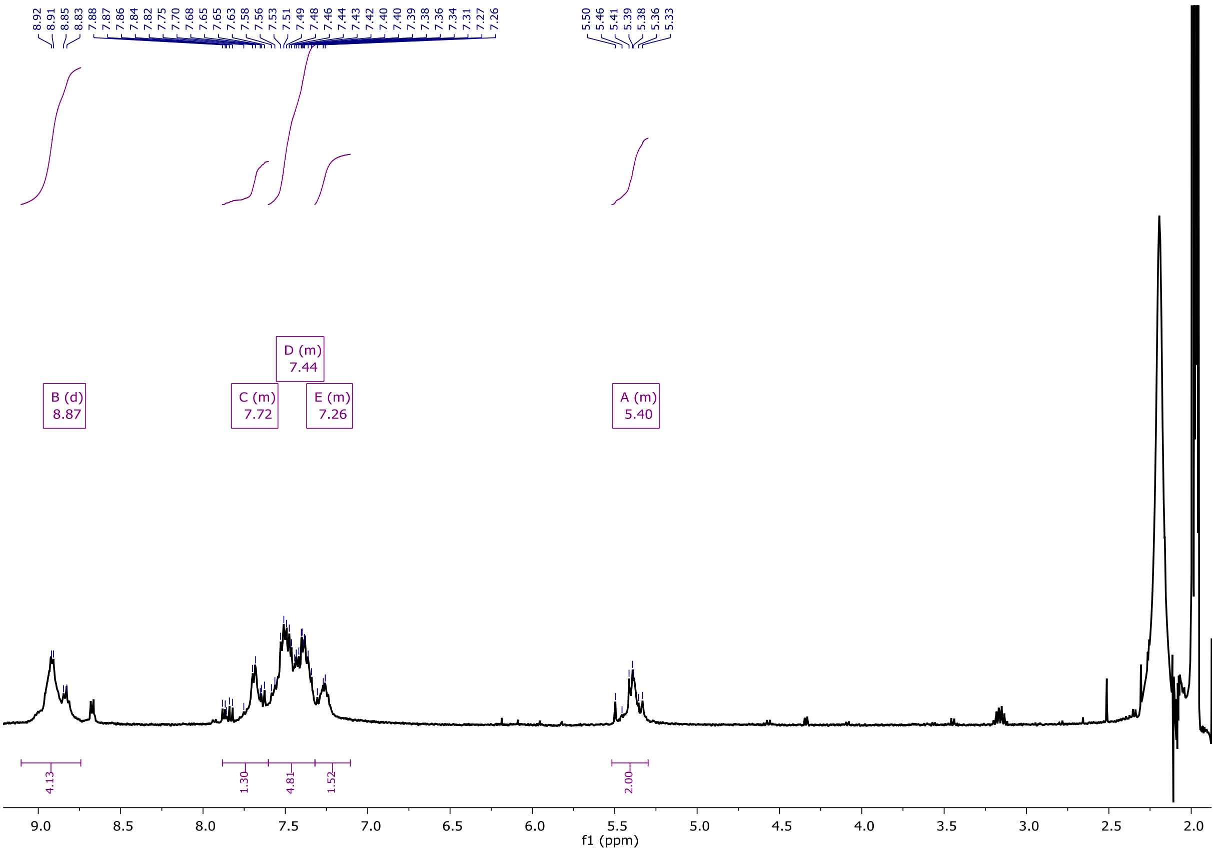

Figure S35. [Pt$_n$($\mathbf{L^{OBn}}$)$_{2n}$]$^{2n+}$(BF_4^-)$_{2n}$ sphere, ^{1}H NMR in MeCN-d$_3$.

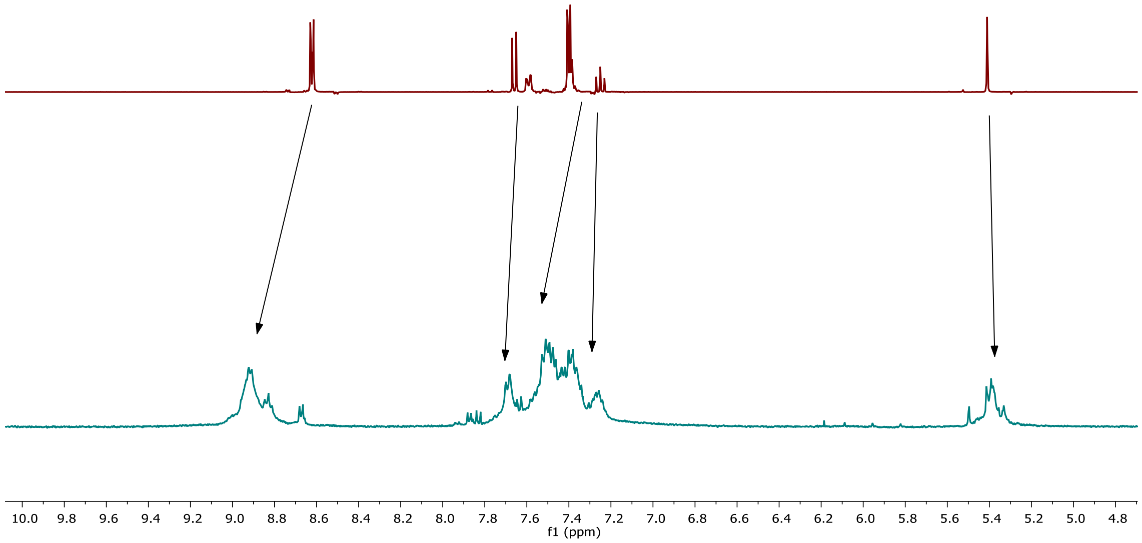

Figure S36. Comparison of free $\mathbf{L}^{\mathbf{OBn}}$ building block (top) and $[Pt_n(\mathbf{L}^{\mathbf{OBn}})_{2n}]^{2n+}(BF_4^-)_{2n}$ sphere (bottom), ^1H NMR in MeCN-d_3.

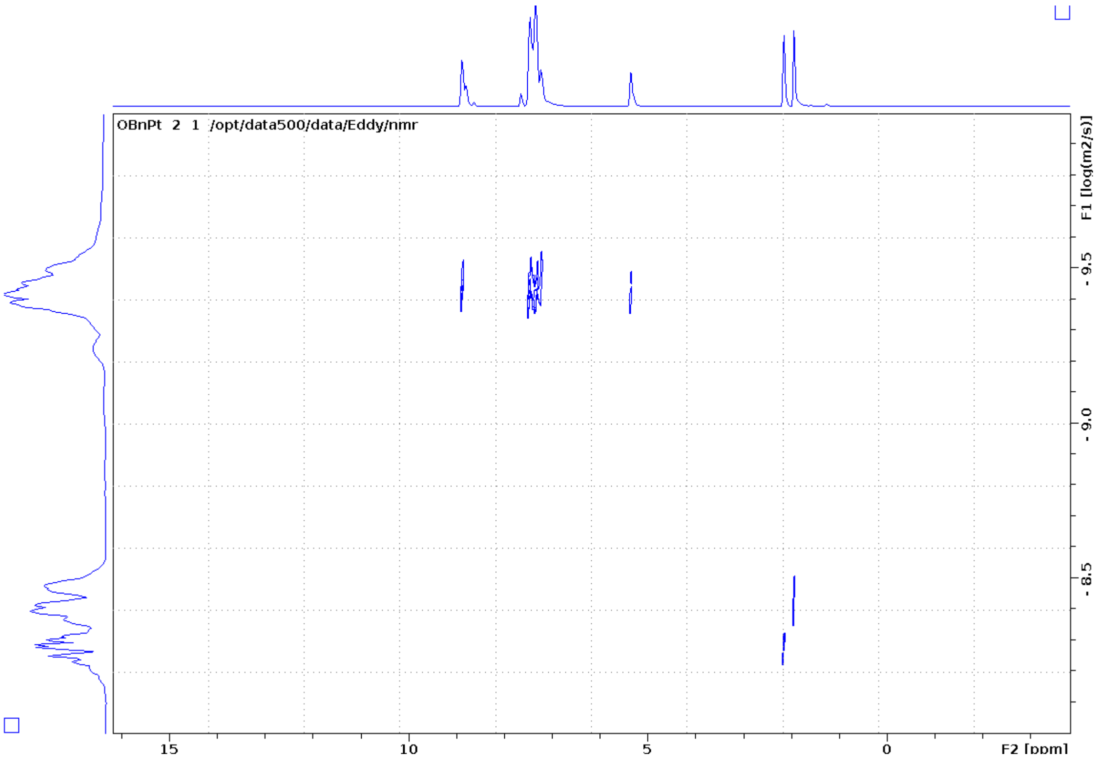

Figure S37. $[Pt_n(\mathbf{L}^{\mathbf{OBn}})_{2n}]^{2n+}(BF_4^-)_{2n}$ cage prepared at 150°C, DOSY in MeCN-d_3 at 25°C.

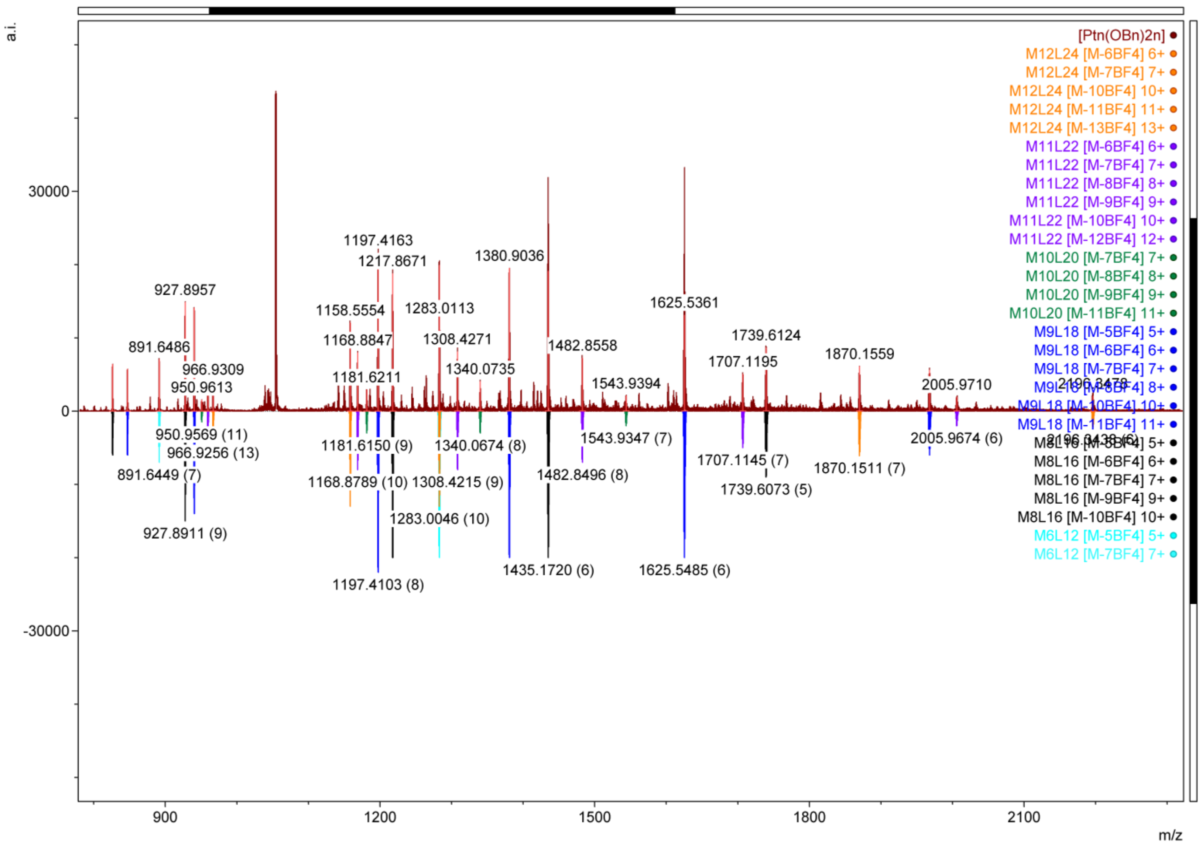

Figure S38. Full ESI-MS spectra of $[Pt_n(\mathbf{L}^{OBn})_{2n}]^{2n+}(BF4^-)_{2n}$ prepared at 150°C for 2d. Below the simulated spectra of different sphere types, above obtained spectra.

Table S3. Calculated and observed species of the $[Pt_n(\mathbf{L^{OBn}})_{2n}]^{2n+}$ assemblies of a sample prepared at 150°C for 2d.

Cage Type	Composition	Calculated	Found
M_6L_{12}	$[Pt_6(\mathbf{L^{OBn}})_{12}(BF_4^-)_5]^{7+}$	891.644	891.6486
	$[Pt_6(\mathbf{L^{OBn}})_{12}(BF_4^-)_7]^{5+}$	1282.9044	1282.9102
M_8L_{16}	$[Pt_8(\mathbf{L^{OBn}})_{16}(BF_4^-)_6]^{10+}$	826.4017	826.4046
	$[Pt_8(\mathbf{L^{OBn}})_{16}(BF_4^-)_7]^{9+}$	927.8911	927.8957
	$[Pt_8(\mathbf{L^{OBn}})_{16}(BF_4^-)_9]^{7+}$	1217.8613	1217.8671
	$[Pt_8(\mathbf{L^{OBn}})_{16}(BF_4^-)_{10}]^{6+}$	1435.1720	1435.1778
	$[Pt_8(\mathbf{L^{OBn}})_{16}(BF_4^-)_{11}]^{5+}$	1739.6073	1739.6124
M_9L_{18}	$[Pt_9(\mathbf{L^{OBn}})_{18}(BF_4^-)_7]^{11+}$	847.2063	847.2099
	$[Pt_9(\mathbf{L^{OBn}})_{18}(BF_4^-)_8]^{10+}$	940.5274	940.5316
	$[Pt_9(\mathbf{L^{OBn}})_{18}(BF_4^-)_{10}]^{8+}$	1197.4103	1197.4163
	$[Pt_9(\mathbf{L^{OBn}})_{18}(BF_4^-)_{11}]^{7+}$	1380.8981	1380.9036
	$[Pt_9(\mathbf{L^{OBn}})_{18}(BF_4^-)_{12}]^{6+}$	1625.5485	1625.5361
	$[Pt_9(\mathbf{L^{OBn}})_{18}(BF_4^-)_{13}]^{5+}$	1967.8587	1967.8636
$M_{10}L_{20}$	$[Pt_{10}(\mathbf{L^{OBn}})_{20}(BF_4^-)_9]^{11+}$	950.9569	950.9613
	$[Pt_{10}(\mathbf{L^{OBn}})_{20}(BF_4^-)_{11}]^{9+}$	1181.6150	1181.6211
	$[Pt_{10}(\mathbf{L^{OBn}})_{20}(BF_4^-)_{12}]^{8+}$	1340.0674	1340.0735
	$[Pt_{10}(\mathbf{L^{OBn}})_{20}(BF_4^-)_{13}]^{7+}$	1543.9347	1543.9394
$M_{11}L_{22}$	$[Pt_{11}(\mathbf{L^{OBn}})_{22}(BF_4^-)_{10}]^{12+}$	959.5650	959.5700
	$[Pt_{11}(\mathbf{L^{OBn}})_{22}(BF_4^-)_{12}]^{10+}$	1168.8789	1168.8847
	$[Pt_{11}(\mathbf{L^{OBn}})_{22}(BF_4^-)_{13}]^{9+}$	1308.4215	1308.4271
	$[Pt_{11}(\mathbf{L^{OBn}})_{22}(BF_4^-)_{14}]^{8+}$	1482.8496	1482.8558
	$[Pt_{11}(\mathbf{L^{OBn}})_{22}(BF_4^-)_{15}]^{7+}$	1707.1145	1707.1195
	$[Pt_{11}(\mathbf{L^{OBn}})_{22}(BF_4^-)_{16}]^{6+}$	2005.9674	2005.9710
$M_{12}L_{24}$	$[Pt_{12}(\mathbf{L^{OBn}})_{24}(BF_4^-)_{11}]^{13+}$	966.9256	966.9309
	$[Pt_{12}(\mathbf{L^{OBn}})_{24}(BF_4^-)_{13}]^{11+}$	1158.5493	1158.5554
	$[Pt_{12}(\mathbf{L^{OBn}})_{24}(BF_4^-)_{14}]^{10+}$	1283.0046	1283.0113
	$[Pt_{12}(\mathbf{L^{OBn}})_{24}(BF_4^-)_{17}]^{7+}$	1870.1511	1870.1559
	$[Pt_{12}(\mathbf{L^{OBn}})_{24}(BF_4^-)_{18}]^{6+}$	2196.3438	2196.3478

Table S4. Zoom into different charged species of $[Pt_n(\mathbf{L}^{OBn})_{2n}]^{2n+}$ assemblies of a sample prepared at 150°C for 2d.

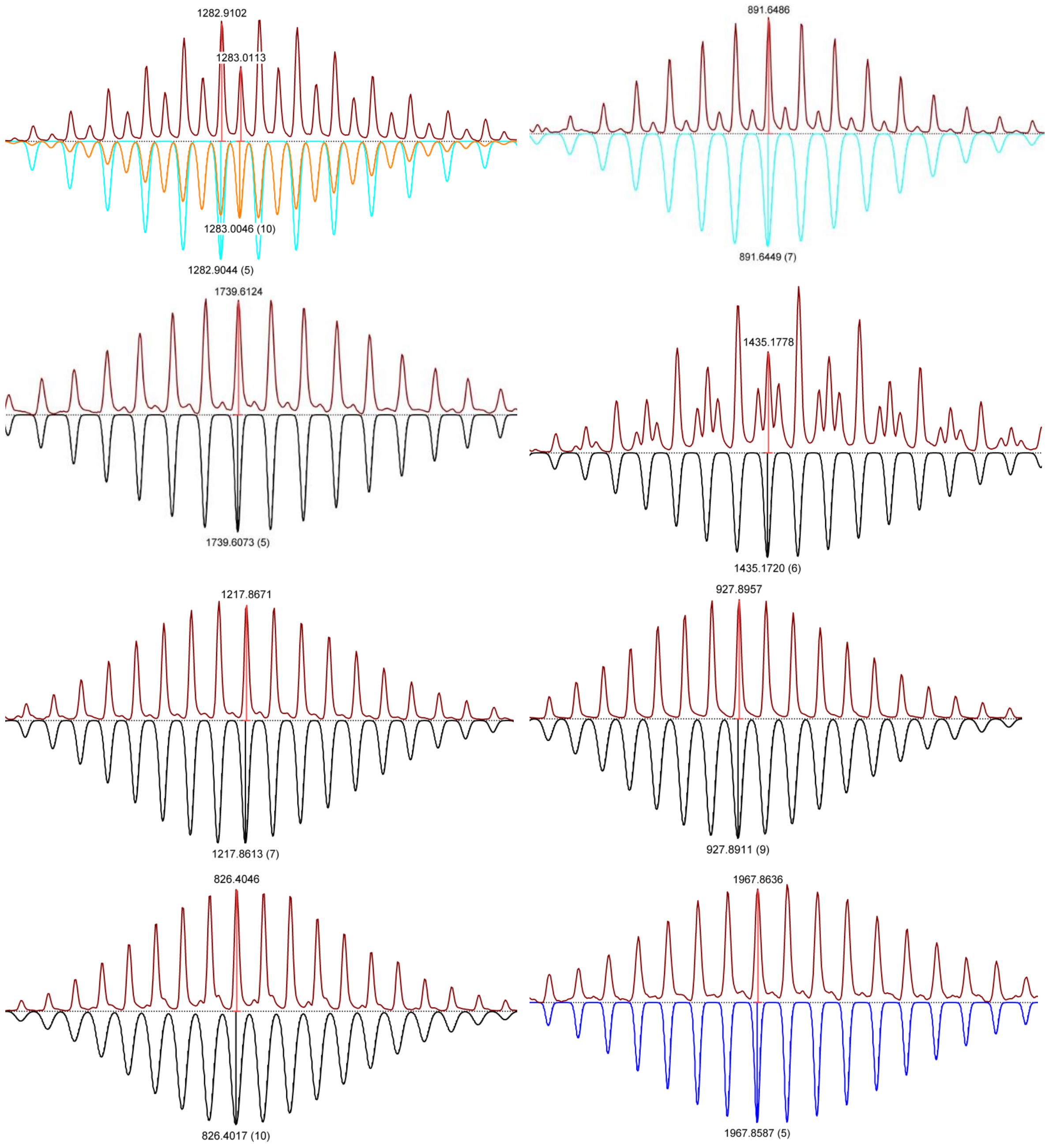

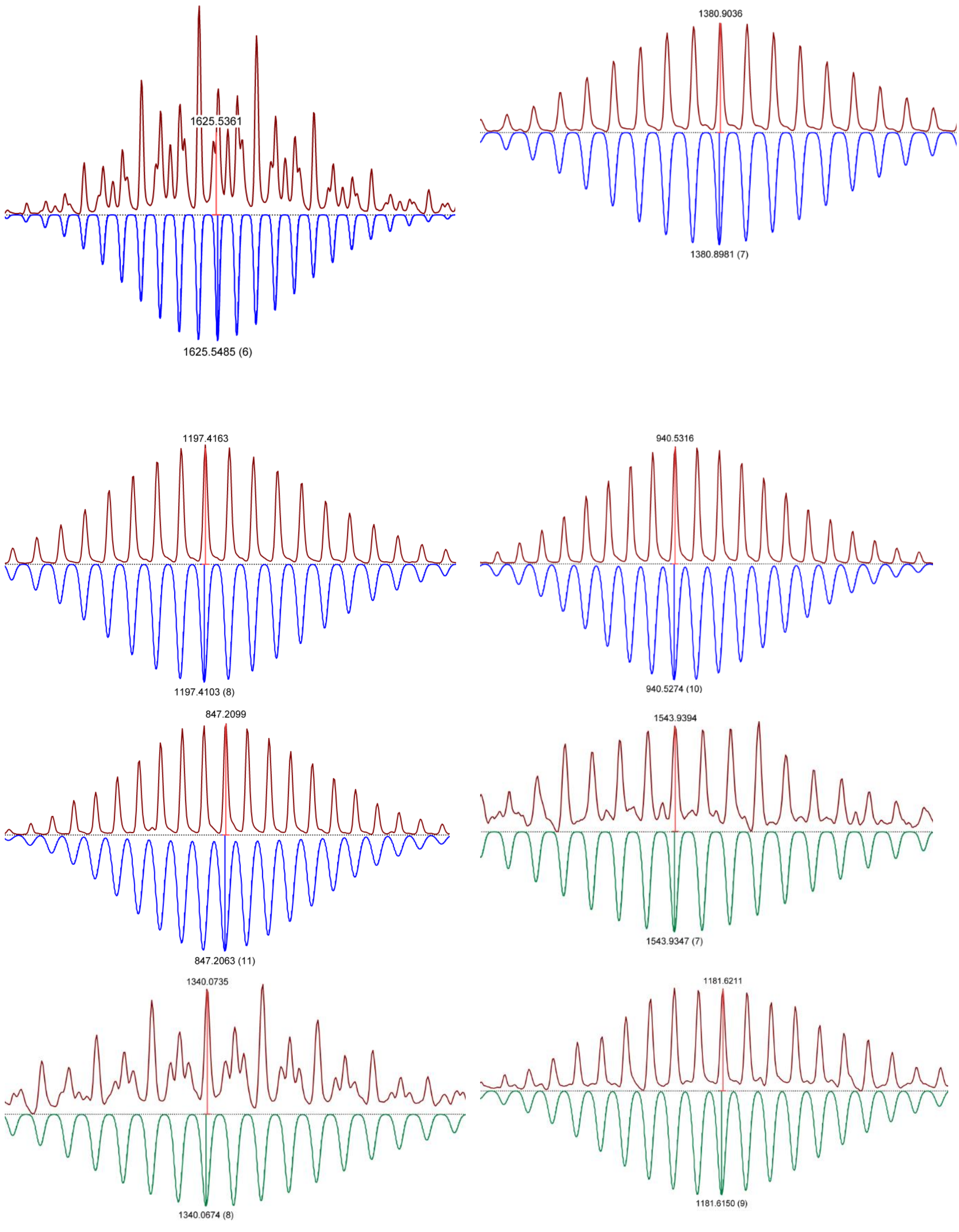
1625.5361
1625.5485 (6)
1380.9036
1380.8981 (7)
1197.4163
1197.4103 (8)
940.5316
940.5274 (10)
847.2099
847.2063 (11)
1543.9394
1543.9347 (7)
1340.0735
1340.0674 (8)
1181.6211
1181.6150 (9)

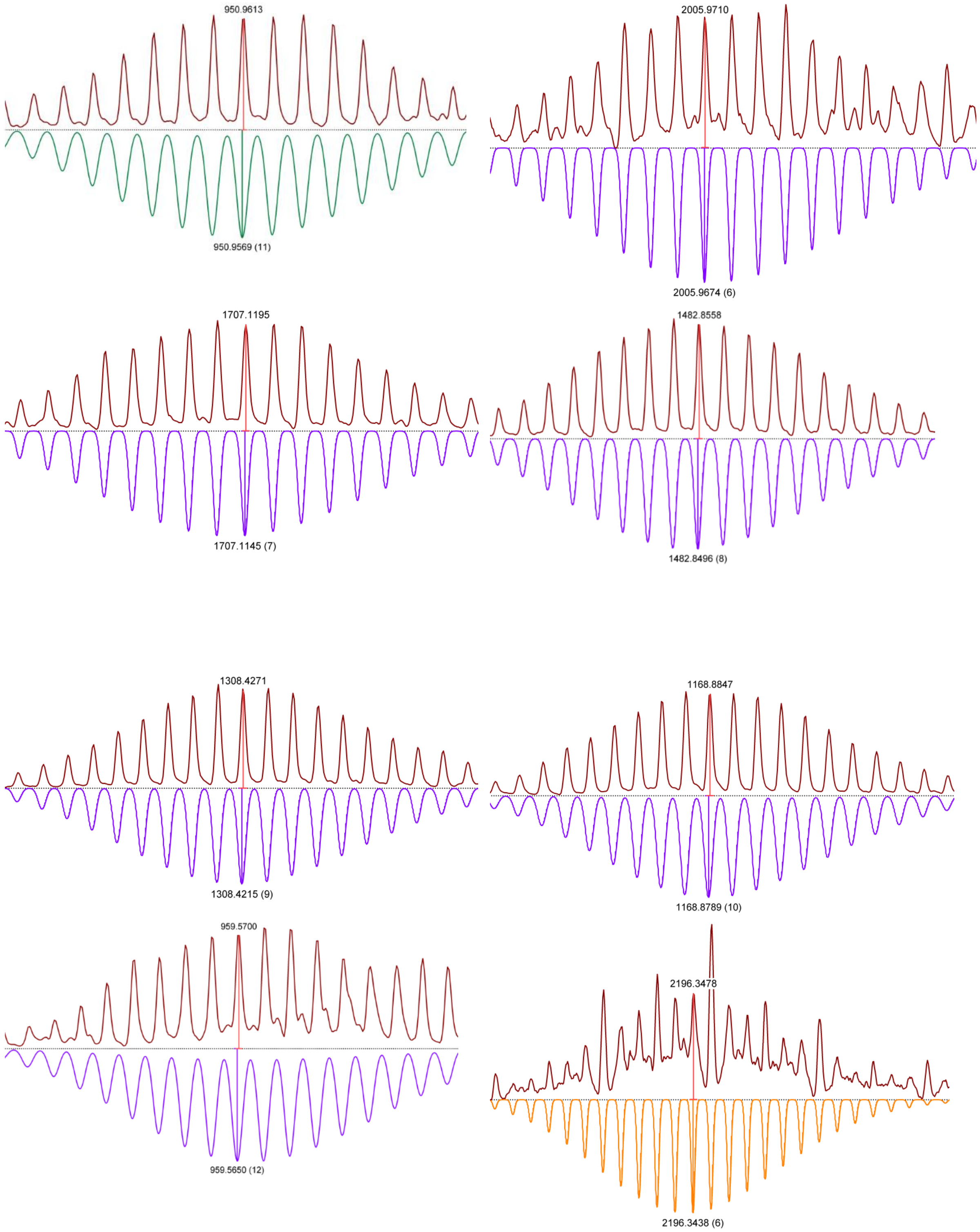
950.9613
950.9569 (11)
2005.9710
2005.9674 (6)
1707.1195
1707.1145 (7)
1482.8558
1482.8496 (8)
1308.4271
1308.4215 (9)
1168.8847
1168.8789 (10)
959.5700
959.5650 (12)
2196.3478
2196.3438 (6)

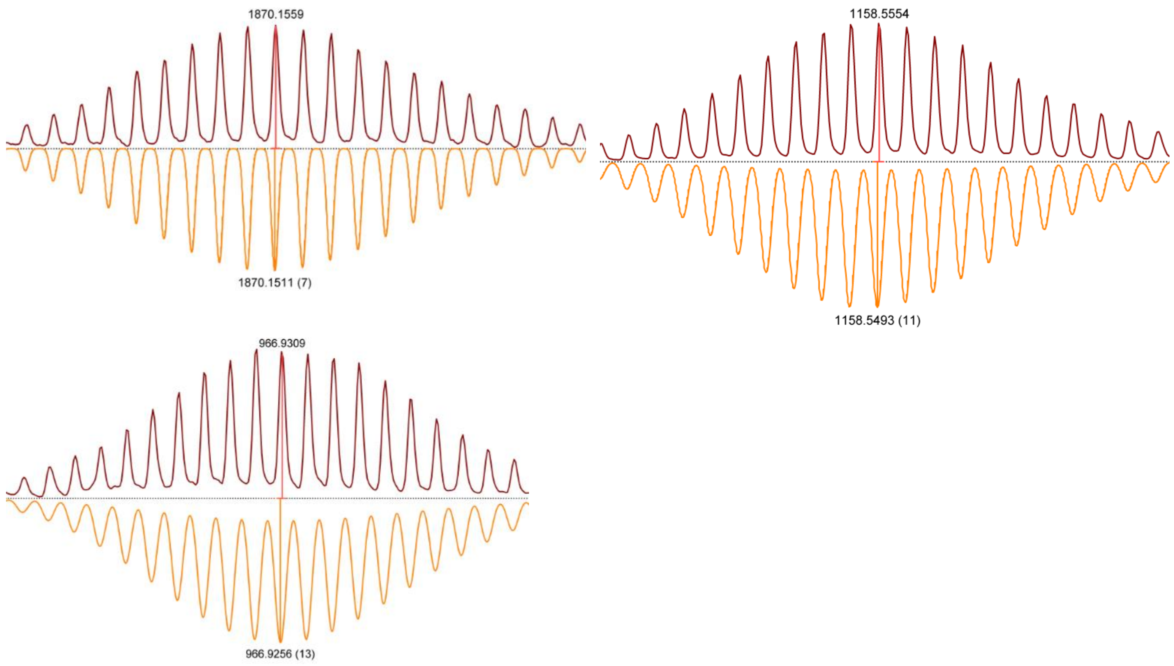
1870.1559
1870.1511 (7)
1158.5554
1158.5493 (11)
966.9309
966.9256 (13)

[$Pt_n(\mathbf{L^{Cou}})_{2n}]^{2n+}(BF_4^-)_{2n}$

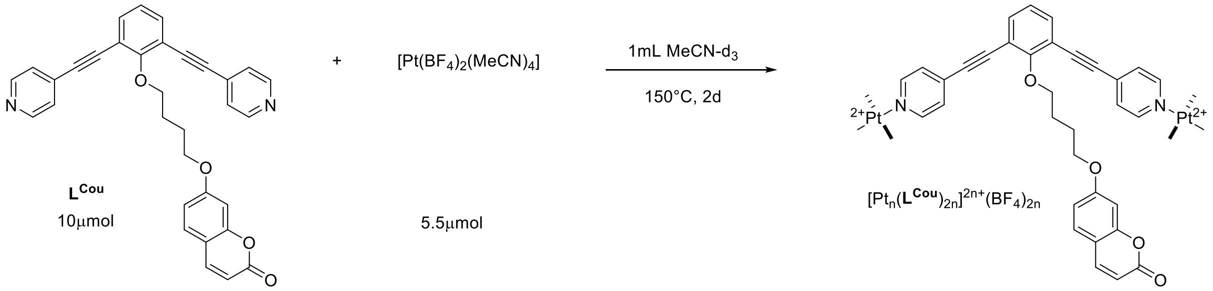

To a solution of $\mathbf{L^{Cou}}$ (5.12 mg, 10 µmol, 1 eq.) in 0.5 mL MeCN-d3, [$Pt(BF_4)_2(MeCN)_4$] (2.93 mg, 5.5 µmol. 0.55 eq.) in 0.5 mL MeCN-d3 was added. The solution was then stirred at 150°C for 2d in a 10 mL high pressure tube. ^{1}H NMR (400 MHz, Acetonitrile-*d*3) δ 9.16 – 8.79 (m, 4H), 7.83 – 7.49 (m, 7H), 7.43 (dd, *J* = 27.9, 8.8 Hz, 0H), 7.31 – 7.16 (m, 1H), 6.83 – 6.42 (m, 2H), 6.25 – 6.02 (m, 1H), 4.29 (s, 1H), 4.20 – 3.89 (m, 2H). LogD(m^2/s) at 25°C (MeCN-d3): -9.304 (d = 2.56 nm).

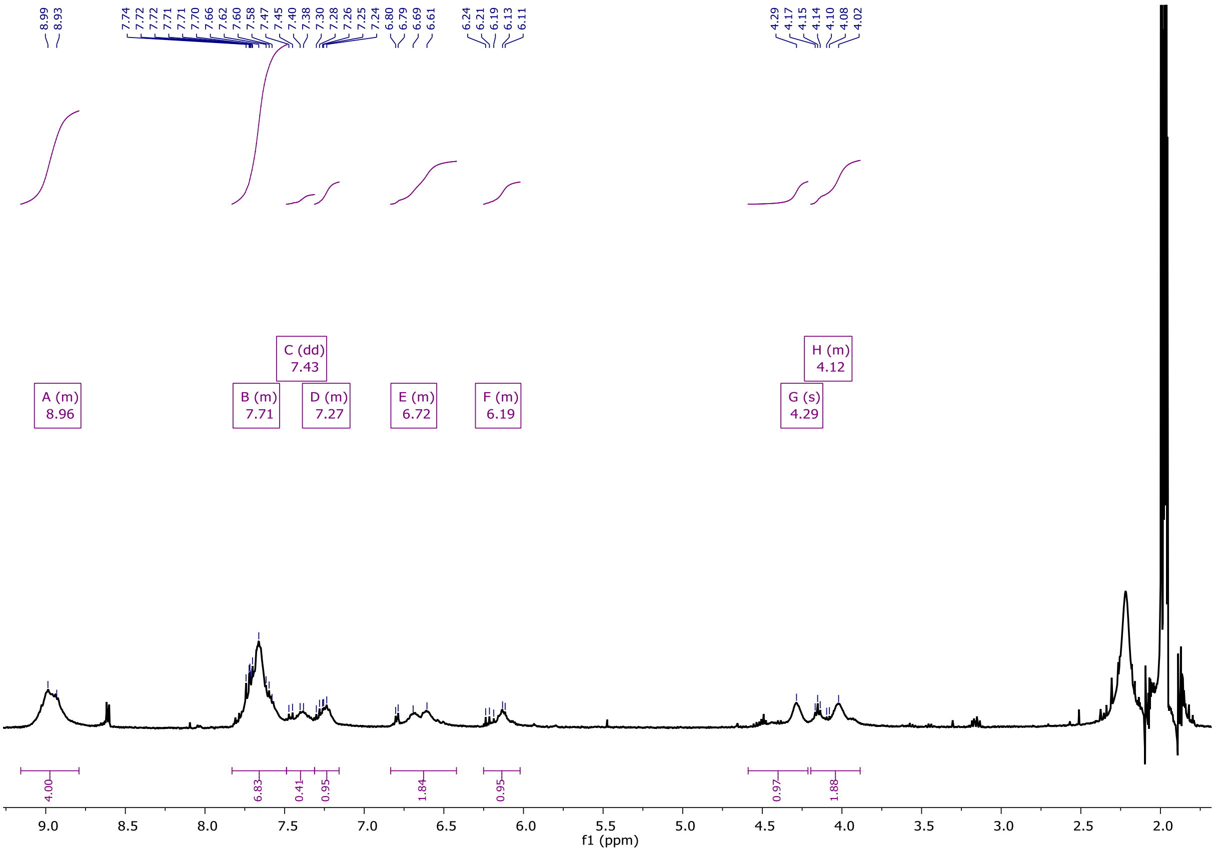

Figure S39. [$Pt_n(\mathbf{L^{Cou}})_{2n}]^{2n+}(BF_4^-)_{2n}$ sphere, ^{1}H NMR in MeCN-d3.

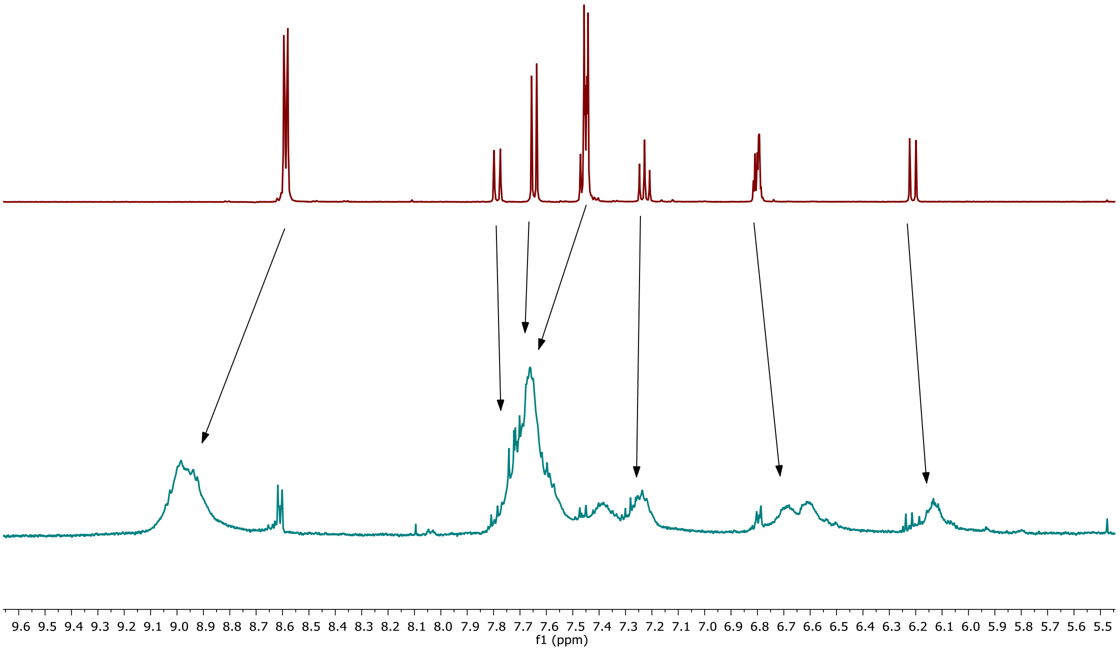

Figure S40. Comparison of free $\mathbf{L}^{\mathbf{Cou}}$ building block (top) and $[Pt_n(\mathbf{L}^{\mathbf{Cou}})_{2n}]^{2n+}(BF_4^-)_{2n}$ sphere (bottom), ^1H NMR in MeCN-d_3.

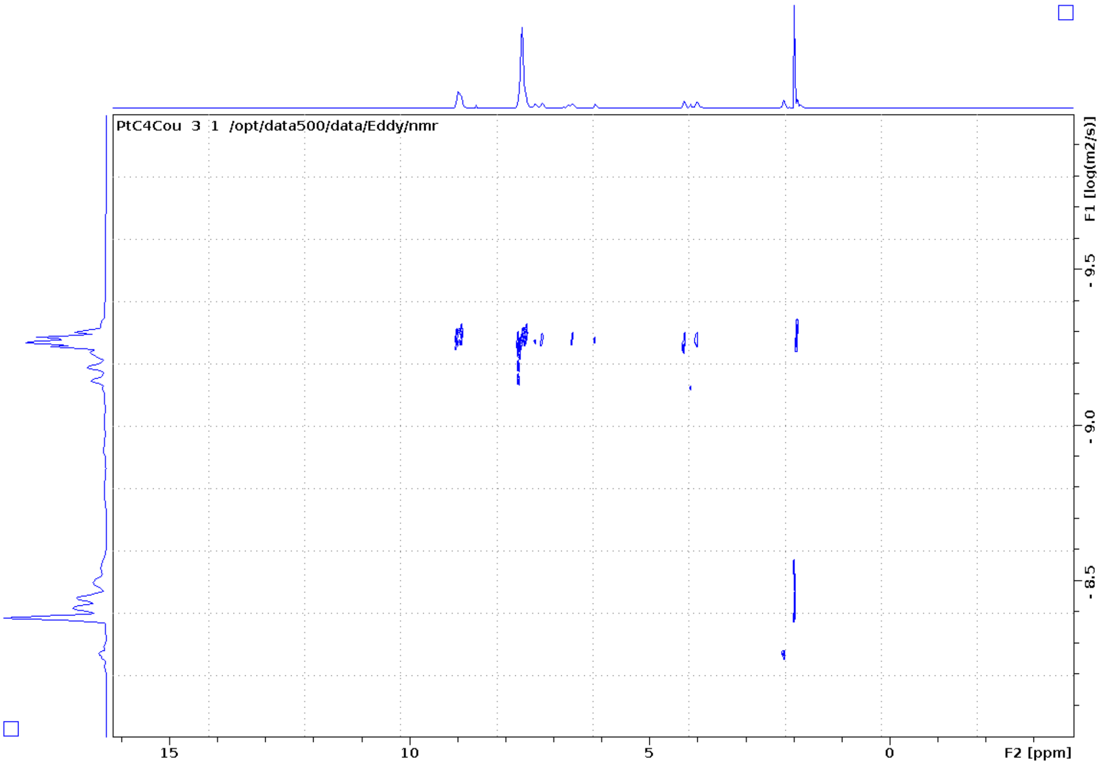

Figure S41. $[Pt_n(\mathbf{L}^{\mathbf{Cou}})_{2n}]^{2n+}(BF_4^-)_{2n}$ cage prepared at 150°C, DOSY in MeCN-d_3 at 25°C.

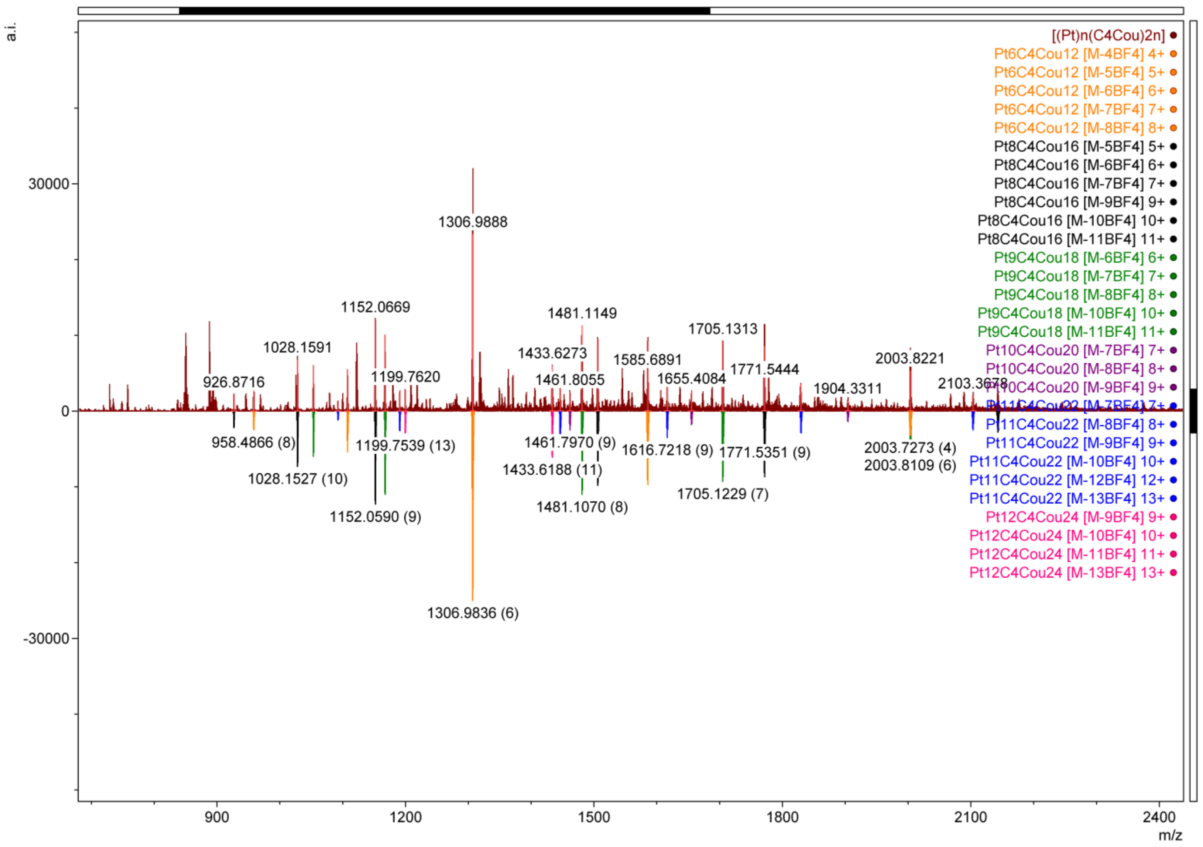

Figure S42. Full ESI-MS spectra of $[Pt_n(\mathbf{L^{Cou}})_{2n}]^{2n+}(BF4^{-})_{2n}$ prepared at 150°C for 2d. Below the simulated spectra of different sphere types, above obtained spectra.

Table S5. Calculated and observed species of the $[Pt_n(\mathbf{L^{Cou}})_{2n}]^{2n+}$ assemblies of a sample prepared at 150°C for 2d.

Cage Type	Composition	Calculated	Found
M_6L_{12}	$[Pt_6(\mathbf{L^{Cou}})_{12}(BF_4^-)_4]^{8+}$	958.4866	958.4929
	$[Pt_6(\mathbf{L^{Cou}})_{12}(BF_4^-)_5]^{7+}$	1107.8426	1107.8498
	$[Pt_6(\mathbf{L^{Cou}})_{12}(BF_4^-)_6]^{6+}$	1306.9836	1306.9888
	$[Pt_6(\mathbf{L^{Cou}})_{12}(BF_4^-)_7]^{5+}$	1585.5809	1585.5899
	$[Pt_6(\mathbf{L^{Cou}})_{12}(BF_4^-)_8]^{4+}$	2003.7273	2003.7320
M_8L_{16}	$[Pt_8(\mathbf{L^{Cou}})_{16}(BF_4^-)_5]^{11+}$	926.8659	926.8716
	$[Pt_8(\mathbf{L^{Cou}})_{16}(BF_4^-)_6]^{10+}$	1028.1527	1028.1591
	$[Pt_8(\mathbf{L^{Cou}})_{16}(BF_4^-)_7]^{9+}$	1152.0590	1152.0669
	$[Pt_8(\mathbf{L^{Cou}})_{16}(BF_4^-)_9]^{7+}$	1506.0771	1506.0859
	$[Pt_8(\mathbf{L^{Cou}})_{16}(BF_4^-)_{10}]^{6+}$	1771.5908	1771.5986
M_9L_{18}	$[Pt_9(\mathbf{L^{Cou}})_{18}(BF_4^-)_7]^{11+}$	1053.5312	1053.5382
	$[Pt_9(\mathbf{L^{Cou}})_{18}(BF_4^-)_8]^{10+}$	1167.5849	1167.5927
	$[Pt_9(\mathbf{L^{Cou}})_{18}(BF_4^-)_{10}]^{8+}$	1481.1070	1481.1149
	$[Pt_9(\mathbf{L^{Cou}})_{18}(BF_4^-)_{11}]^{7+}$	1705.1229	1705.1313
$M_{10}L_{20}$	$[Pt_{10}(\mathbf{L^{Cou}})_{20}(BF_4^-)_{11}]^{9+}$	1461.7970	1461.8055
	$[Pt_{10}(\mathbf{L^{Cou}})_{20}(BF_4^-)_{12}]^{8+}$	1655.3973	1655.4084
	$[Pt_{10}(\mathbf{L^{Cou}})_{20}(BF_4^-)_{13}]^{7+}$	1904.3118	1904.3311
$M_{11}L_{22}$	$[Pt_{11}(\mathbf{L^{Cou}})_{22}(BF_4^-)_9]^{13+}$	1092.4984	1092.5063
	$[Pt_{11}(\mathbf{L^{Cou}})_{22}(BF_4^-)_{10}]^{12+}$	1190.7902	1190.7980
	$[Pt_{11}(\mathbf{L^{Cou}})_{22}(BF_4^-)_{12}]^{10+}$	1446.3491	1446.3590
	$[Pt_{11}(\mathbf{L^{Cou}})_{22}(BF_4^-)_{13}]^{9+}$	1616.7218	1616.7333
	$[Pt_{11}(\mathbf{L^{Cou}})_{22}(BF_4^-)_{14}]^{8+}$	1829.5623	1829.5700
	$[Pt_{11}(\mathbf{L^{Cou}})_{22}(BF_4^-)_{15}]^{7+}$	2103.3576	2103.3678
$M_{12}L_{24}$	$[Pt_{12}(\mathbf{L^{Cou}})_{24}(BF_4^-)_{11}]^{13+}$	1199.7539	1199.7620
	$[Pt_{12}(\mathbf{L^{Cou}})_{24}(BF_4^-)_{13}]^{11+}$	1433.6188	1433.6273

Table S6. Zoom into different charged species of $[Pt_n(\mathbf{L^{Cou}})_{2n}]^{2n+}$ assemblies of a sample prepared at 150°C for 2d.

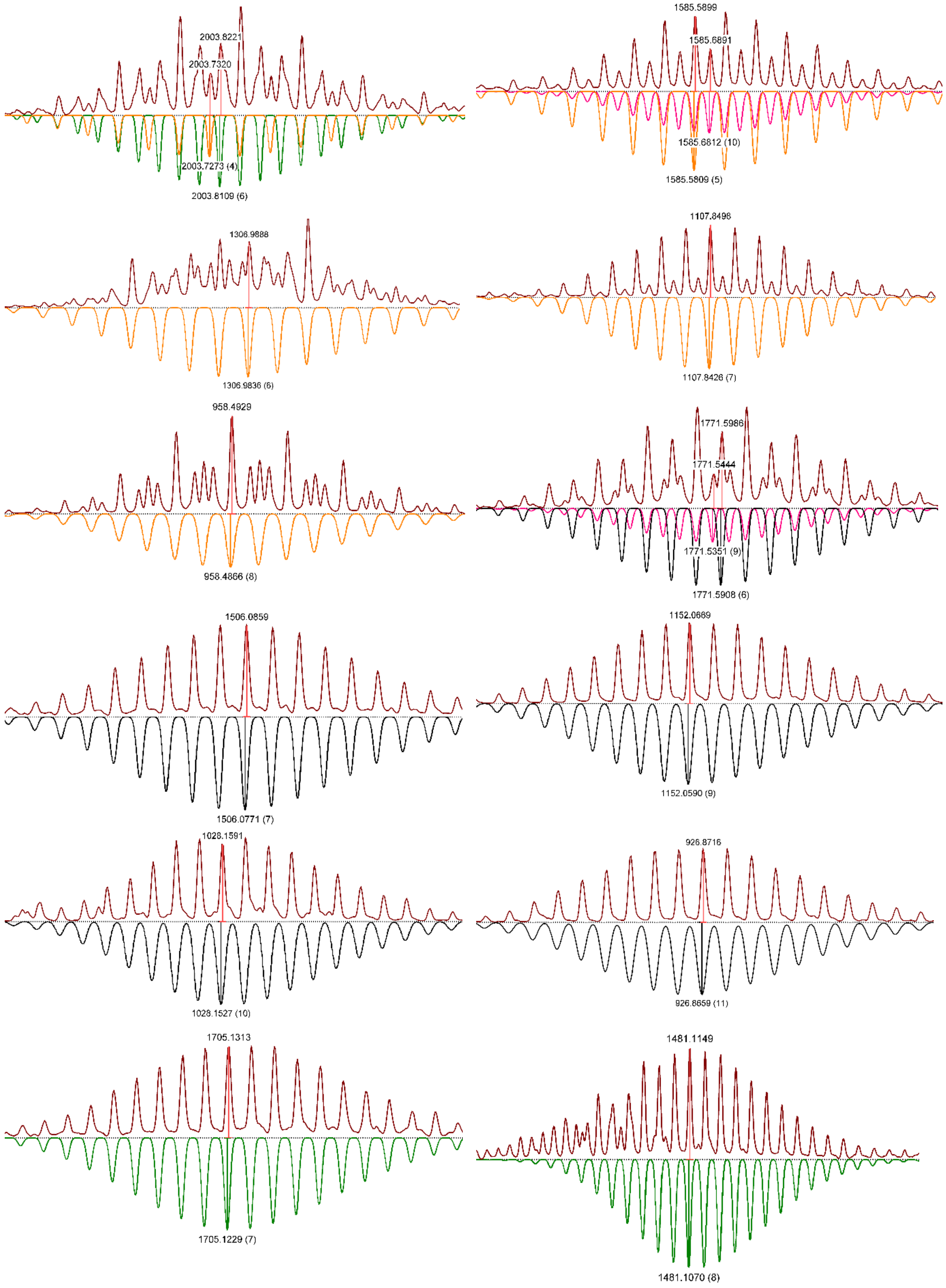

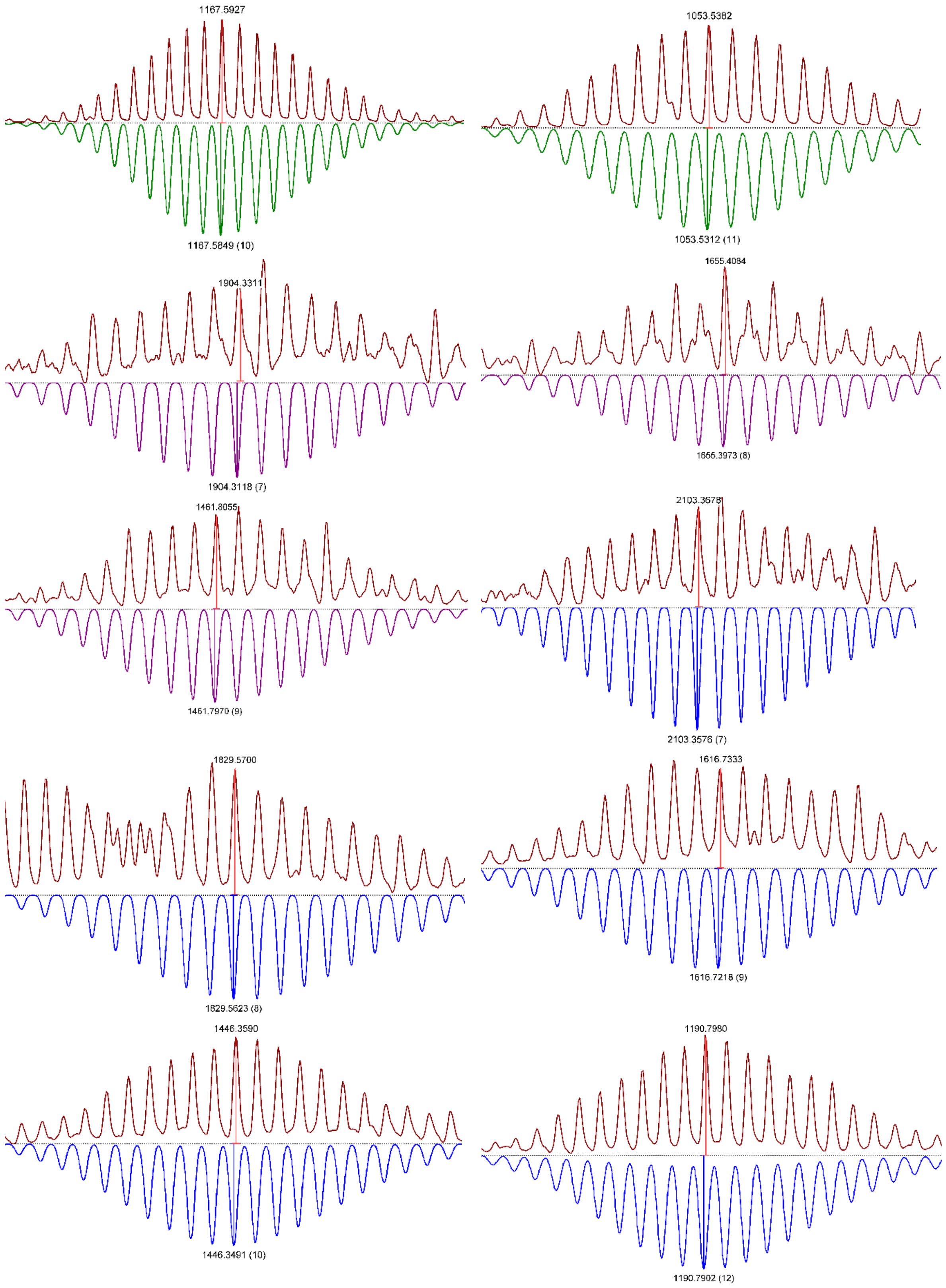
1167.5927
1167.5849 (10)
1053.5382
1053.5312 (11)
1904.3311
1904.3118 (7)
1655.4084
1655.3973 (8)
1461.8055
1461.7970 (9)
2103.3678
2103.3576 (7)
1829.5700
1829.5623 (8)
1616.7333
1616.7218 (9)
1446.3590
1446.3491 (10)
1190.7980
1190.7902 (12)

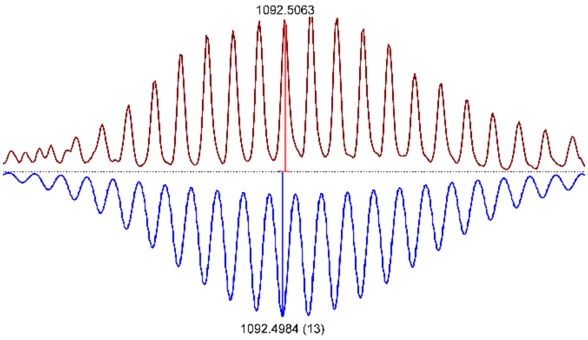
1092.5063
1092.4984 (13)

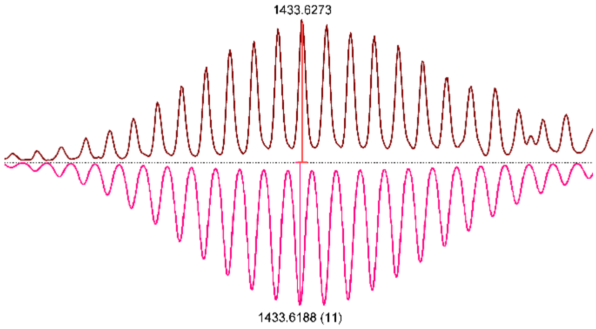
1433.6273
1433.6188 (11)

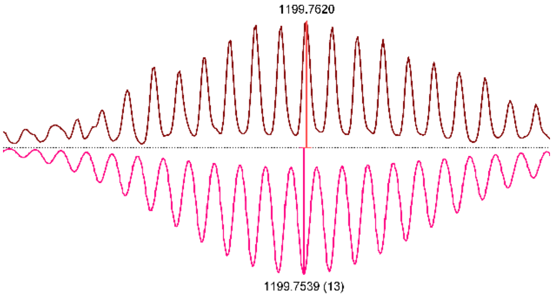
1199.7620
1199.7539 (13)

[Pt$_{12}$(**L^{PO}**)$_{24}$]$^{24+}$(BF$_4^-$)$_{24}$

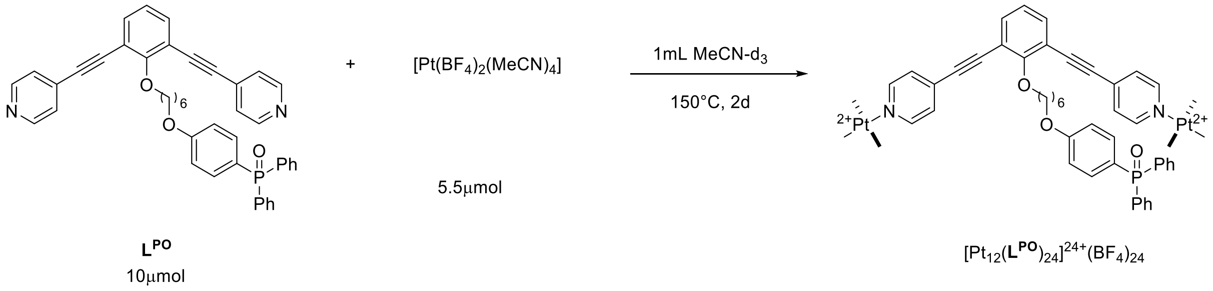

To a solution of **L^{PO}** (6.72 mg, 10 μmol, 1 eq.) in 0.5 mL MeCN-d$_3$, [Pt(BF$_4$)$_2$(MeCN)$_4$] (2.93 mg, 5.5 μmol. 0.55 eq.) in 0.5 mL MeCN-d$_3$ was added. The solution was then stirred at 150°C for 2d in a 10 mL high pressure tube.^{1}H NMR (400 MHz, Acetonitrile-*d*$_3$) δ 9.22 – 8.80 (m, 4H), 7.88 – 7.26 (m, 23H), 7.26 – 7.10 (m, 0H), 4.35 – 4.05 (m, 1H), 3.96 – 3.73 (m, 2H). ^{13}C NMR (101 MHz, CD$_3$CN) δ 172.08, 162.00, 151.76, 141.61, 137.67, 135.47, 133.86, 133.68, 131.59, 128.66, 126.71, 115.51, 114.69, 95.14, 89.33, 75.41, 67.92, 30.05, 28.54, 25.54, 21.01, 20.25. LogD(m^2/s) at 25°C (MeCN-d$_3$): -9.569 (d = 4.72 nm).

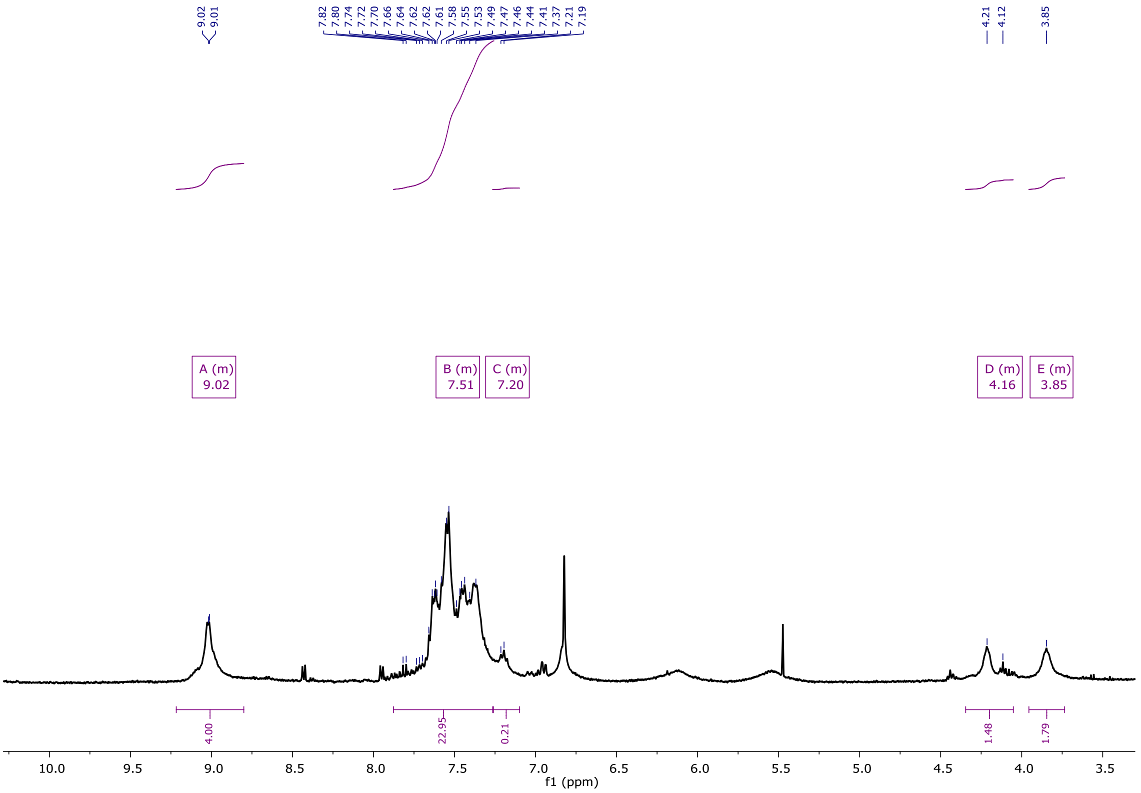

Figure S43. [Pt$_{12}$(**L^{PO}**)$_{24}$]$^{24+}$(BF$_4^-$)$_{24}$ sphere, ^{1}H NMR in MeCN-d$_3$.

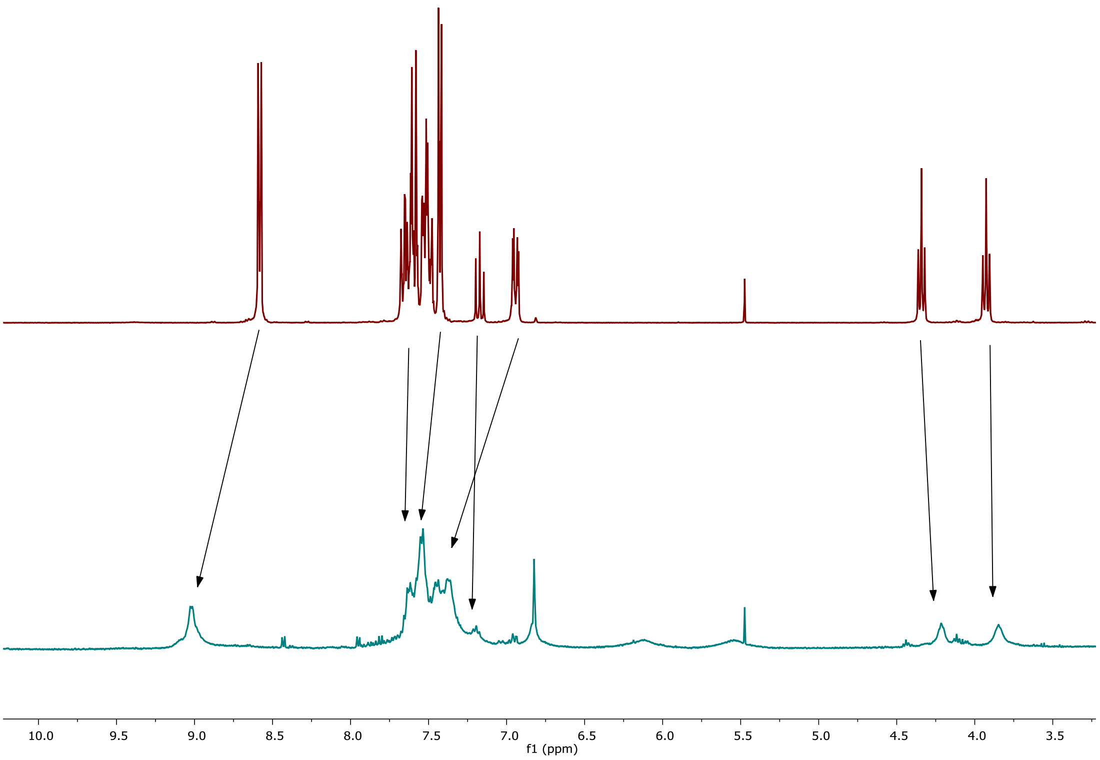

Figure S44. Comparison of free **L^{PO}** building block (top) and $[Pt_{12}($**L^{PO}**$)_{24}]^{24+}(BF_4^-)_{24}$ sphere (bottom), ^{1}H NMR in MeCN-d$_3$.

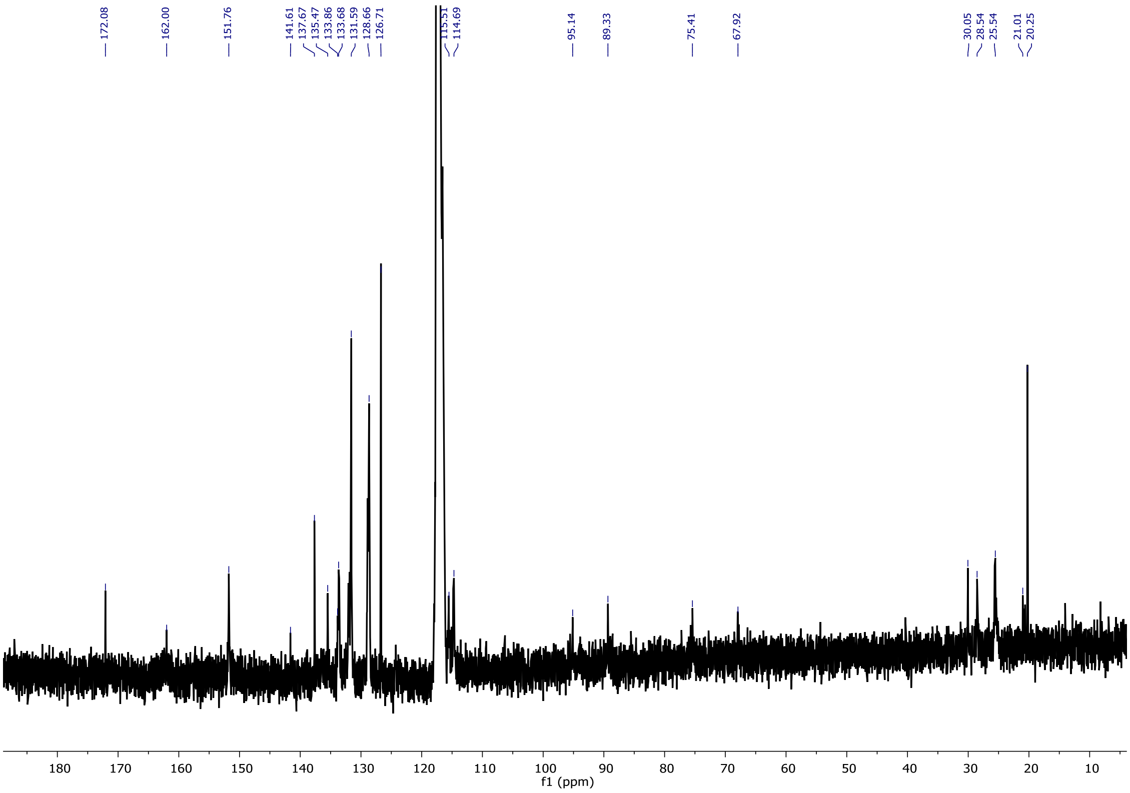

Figure S45. $[Pt_{12}($**L^{PO}**$)_{24}]^{24+}(BF_4^-)_{24}$ sphere, ^{13}C NMR in MeCN-d$_3$.

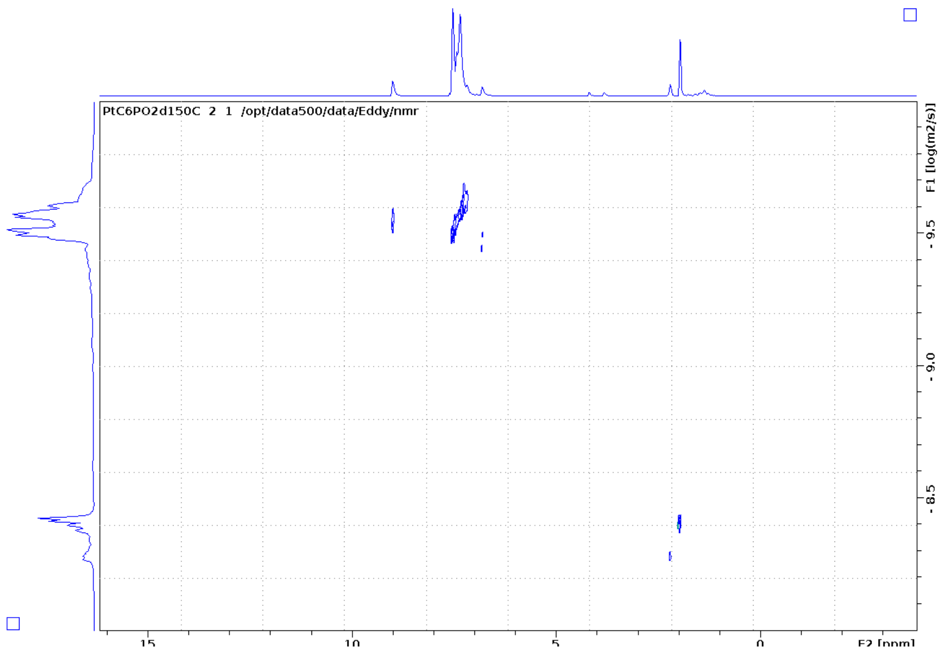

Figure S46. $[Pt_{12}(\mathbf{L^{PO}})_{24}]^{24+}(BF_4^-)_{24}$ cage prepared at 150°C, DOSY in MeCN-d_3 at 25°C.

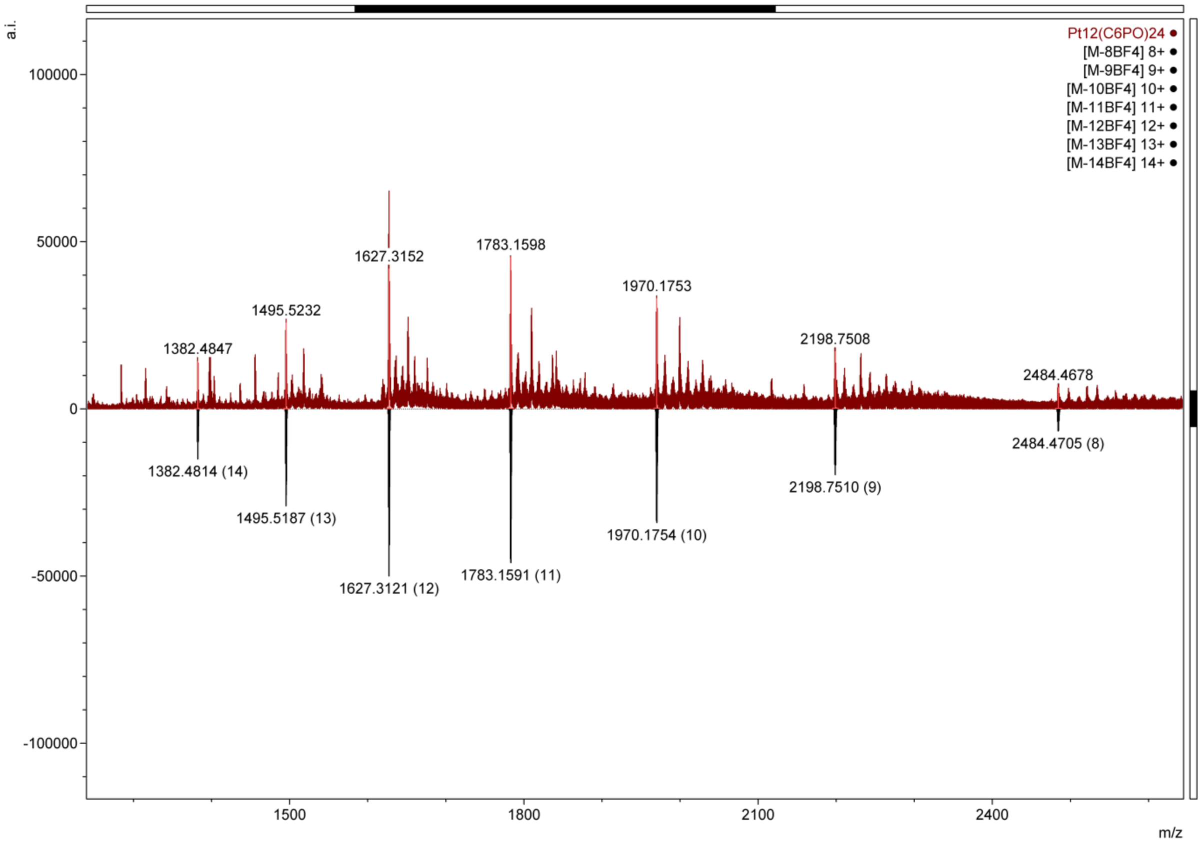

Figure S47. Full ESI-MS spectra of $[Pt_{12}(\mathbf{L^{PO}})_{24}]^{24+}(BF_4^-)_{24}$ prepared at 150°C for 2d. Below the simulated spectra, above obtained spectra. All visible extra signals can be attributed to $Pt_{12}L_{24}$ assemblies with different amounts of solvent trapped in them due to the hydrogen bonding with PO.

Table S7. Calculated and observed species of the $[Pt_{12}(\mathbf{L^{PO}})_{24}]^{24+}$ assembly prepared at 150°C for 2d.

Formula	calculated	obtained
$[Pt_{12}(\mathbf{L^{PO}})_{24}(BF_4^-)_{16}]^{8+}$	2484.4705	2484.4678
$[Pt_{12}(\mathbf{L^{PO}})_{24}(BF_4^-)_{15}]^{9+}$	2198.7510	2198.7508
$[Pt_{12}(\mathbf{L^{PO}})_{24}(BF_4^-)_{14}]^{10+}$	1970.1754	1970.1753
$[Pt_{12}(\mathbf{L^{PO}})_{24}(BF_4^-)_{13}]^{11+}$	1783.1591	1783.1598
$[Pt_{12}(\mathbf{L^{PO}})_{24}(BF_4^-)_{12}]^{12+}$	1627.3121	1627.3152
$[Pt_{12}(\mathbf{L^{PO}})_{24}(BF_4^-)_{11}]^{13+}$	1495.5187	1495.5232
$[Pt_{12}(\mathbf{L^{PO}})_{24}(BF_4^-)_{10}]^{14+}$	1382.4814	1382.4847

Table S8. Zoom into different charged species of $[Pt_{12}(\mathbf{L^{PO}})_{24}]^{24+}$ assembly of a sample prepared at 150°C for 2d.

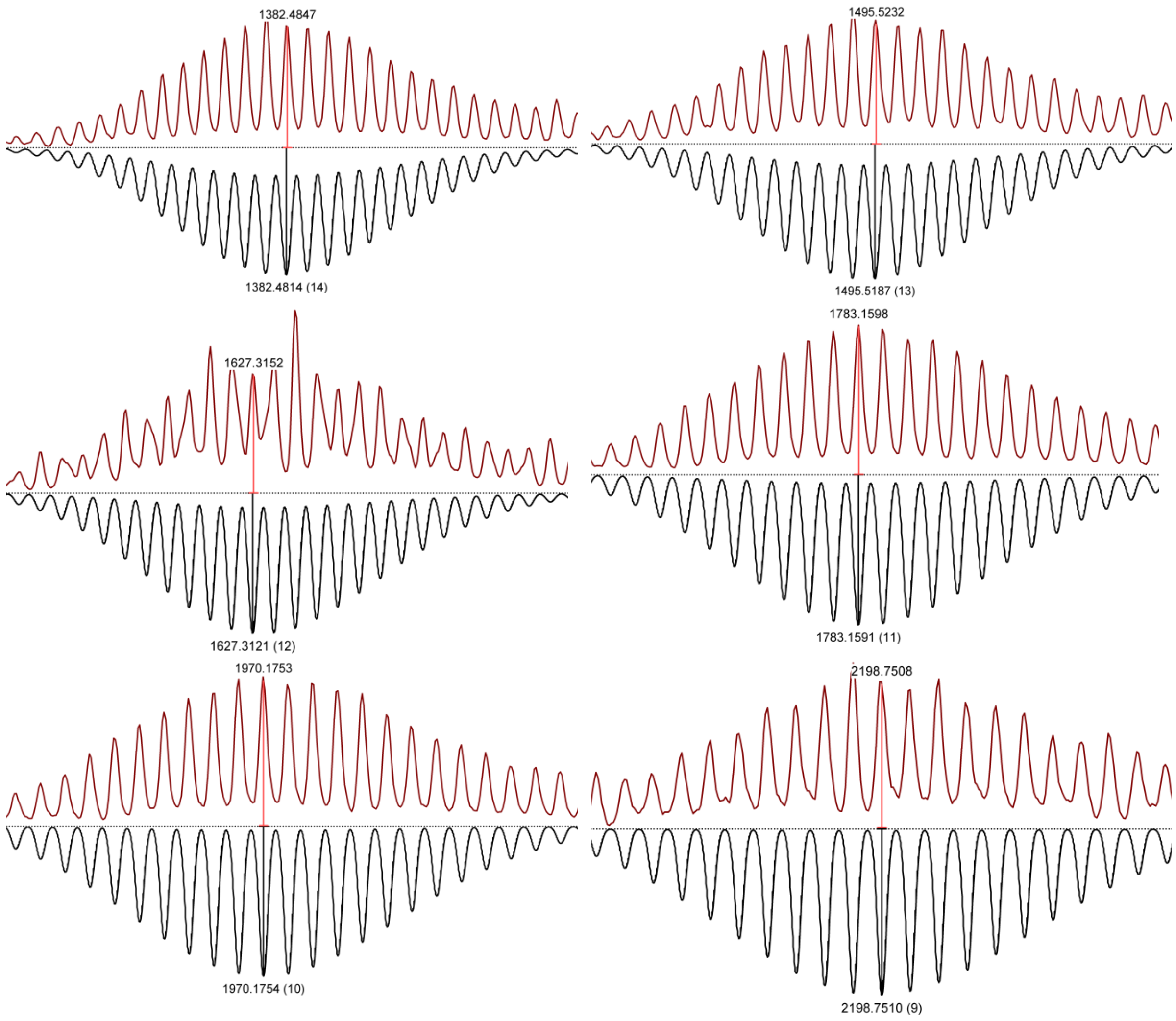

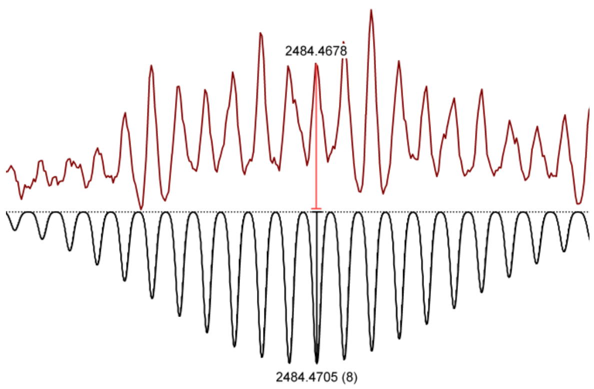

[Pt_{12}(L^{Imi})$_{24}$]$^{48+}$(BF_4^-)$_{48}$

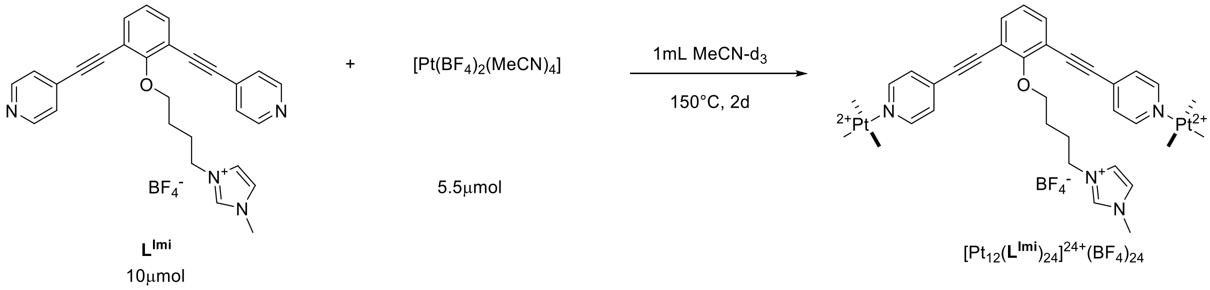

To a solution of **L^Imi** (5.20 mg, 10 μmol, 1 eq.) in 0.5 mL MeCN-d3, [Pt(BF_4)$_2$(MeCN)$_4$] (2.93 mg, 5.5 μmol. 0.55 eq.) in 0.5 mL MeCN-d3 was added. The solution was then stirred at 150°C for 2d in a 10 mL high pressure tube.^{1}H NMR (400 MHz, Acetonitrile-d_3) δ 8.99 (d, *J* = 6.6 Hz, 4H), 8.61 (s, 1H), 7.70 (d, *J* = 7.8 Hz, 1H), 7.65 (d, *J* = 6.1 Hz, 3H), 7.57 – 7.42 (m, 1H), 7.26 (t, *J* = 7.8 Hz, 1H), 4.40 – 4.19 (m, 7H), 3.89 (s, 3H). ^{13}C NMR (101 MHz, CD_3CN) δ 151.84, 137.67, 136.36, 135.41, 129.04, 126.71, 123.76, 122.33, 115.74, 94.64, 89.45, 74.59, 49.25, 35.84, 26.75, 20.26. ^{19}F NMR (282 MHz, $CDCl_3$) δ -148.48, -148.53. LogD(m^2/s) at 25°C (MeCN-d3): -9.459 (d = 3.66 nm).

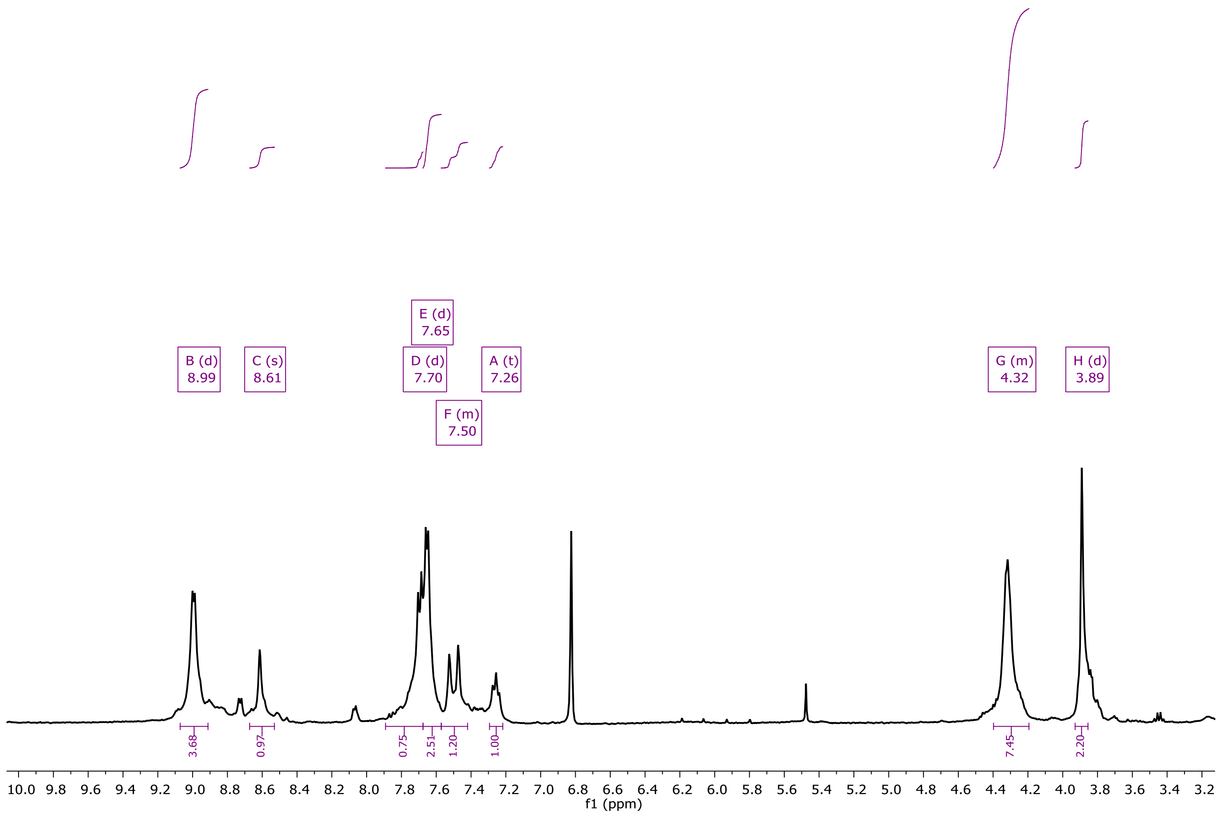

Figure S48. $[Pt_{12}(\mathbf{L^{Imi}})_{24}]^{24+}(BF_4^-)_{24}$ sphere, ^1H NMR in MeCN-d3.

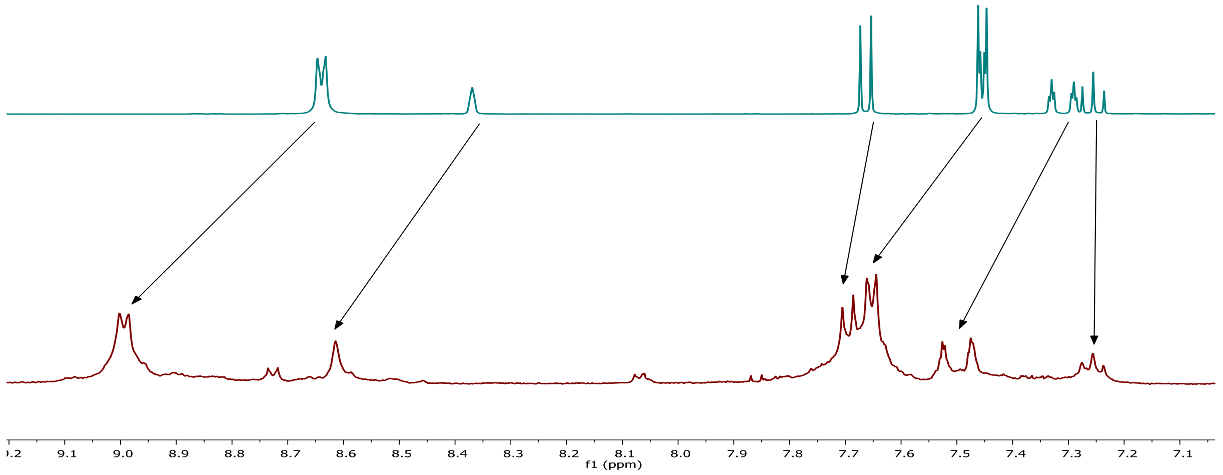

Figure S49. Comparison of free $\mathbf{L^{Imi}}$ building block (top) and $[Pt_{12}(\mathbf{L^{Imi}})_{24}]^{24+}(BF_4^-)_{24}$ sphere (bottom), ^1H NMR in MeCN-d3.

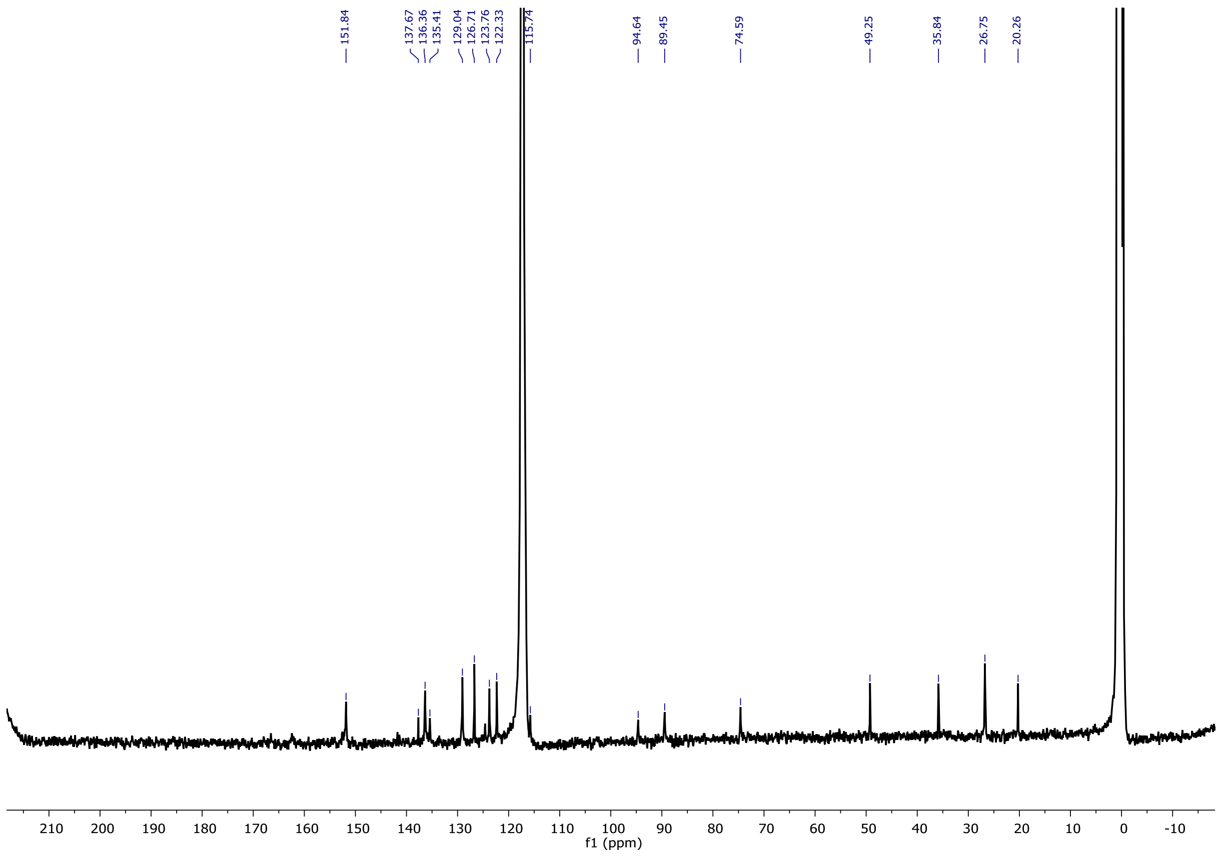

Figure S50. $[Pt_{12}(\mathbf{L^{Imi}})_{24}]^{24+}(BF_4^-)_{24}$ sphere, ^{13}C NMR in MeCN-d$_3$.

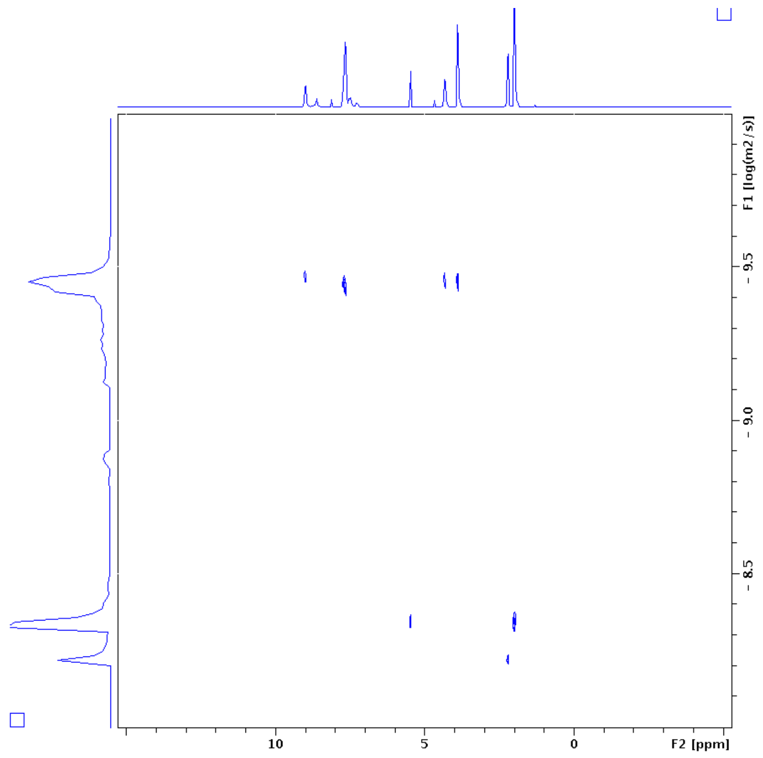

Figure S51. $[Pt_{12}(\mathbf{L^{Imi}})_{24}]^{48+}(BF_4^-)_{48}$ cage prepared at 150°C, DOSY in MeCN-d$_3$ at 25°C.

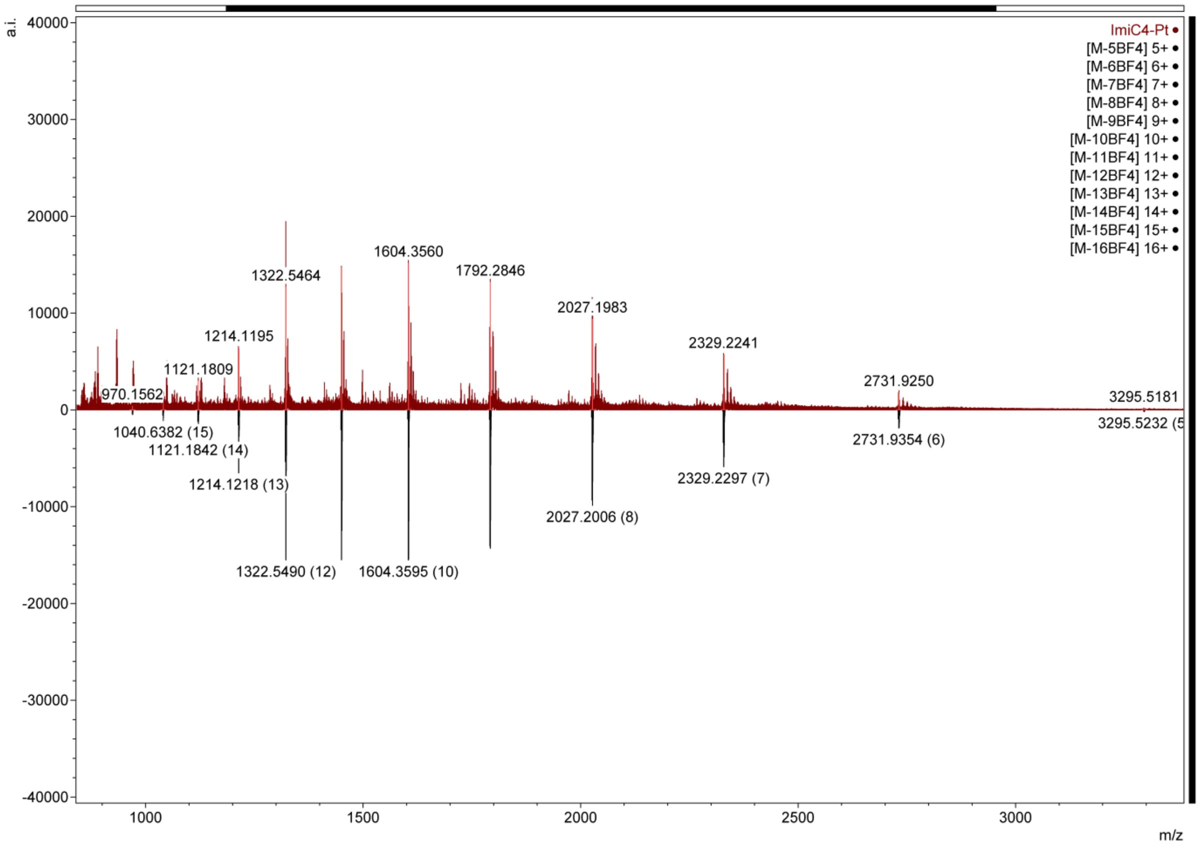

Figure S52. Full ESI-MS spectra of $[Pt_{12}(\mathbf{L^{Imi}})_{24}]^{48+}(BF_4^-)_{48}$ prepared at 150°C for 2d. Below the simulated spectra, above obtained spectra.

Table S9. Calculated and observed species of the $[Pt_{12}(\mathbf{L^{Imi}})_{24}]^{48+}$ assembly prepared at 150°C for 2d.

Formula	calculated	obtained
$[Pt_{12}(\mathbf{C4IMI})_{24}(BF_4^-)_{43}]^{5+}$	3295.5232	3295.5181
$[Pt_{12}(\mathbf{C4IMI})_{24}(BF_4^-)_{42}]^{6+}$	2731.9354	2731.9250
$[Pt_{12}(\mathbf{C4IMI})_{24}(BF_4^-)_{41}]^{7+}$	2329.2297	2329.2241
$[Pt_{12}(\mathbf{C4IMI})_{24}(BF_4^-)_{40}]^{8+}$	2027.2006	2027.1983
$[Pt_{12}(\mathbf{C4IMI})_{24}(BF_4^-)_{39}]^{9+}$	1792.2888	1792.2846
$[Pt_{12}(\mathbf{C4IMI})_{24}(BF_4^-)_{38}]^{10+}$	1604.3595	1604.3560
$[Pt_{12}(\mathbf{C4IMI})_{24}(BF_4^-)_{37}]^{11+}$	1450.6902	1450.6878
$[Pt_{12}(\mathbf{C4IMI})_{24}(BF_4^-)_{36}]^{12+}$	1322.5490	1322.5464
$[Pt_{12}(\mathbf{C4IMI})_{24}(BF_4^-)_{35}]^{13+}$	1214.1218	1214.1195
$[Pt_{12}(\mathbf{C4IMI})_{24}(BF_4^-)_{34}]^{14+}$	1121.1842	1121.1809

Table S10. Zoom into different charged species of $[Pt_{12}(\mathbf{L^{Imi}})_{24}]^{24+}$ assembly of a sample prepared at 150°C for 2d.

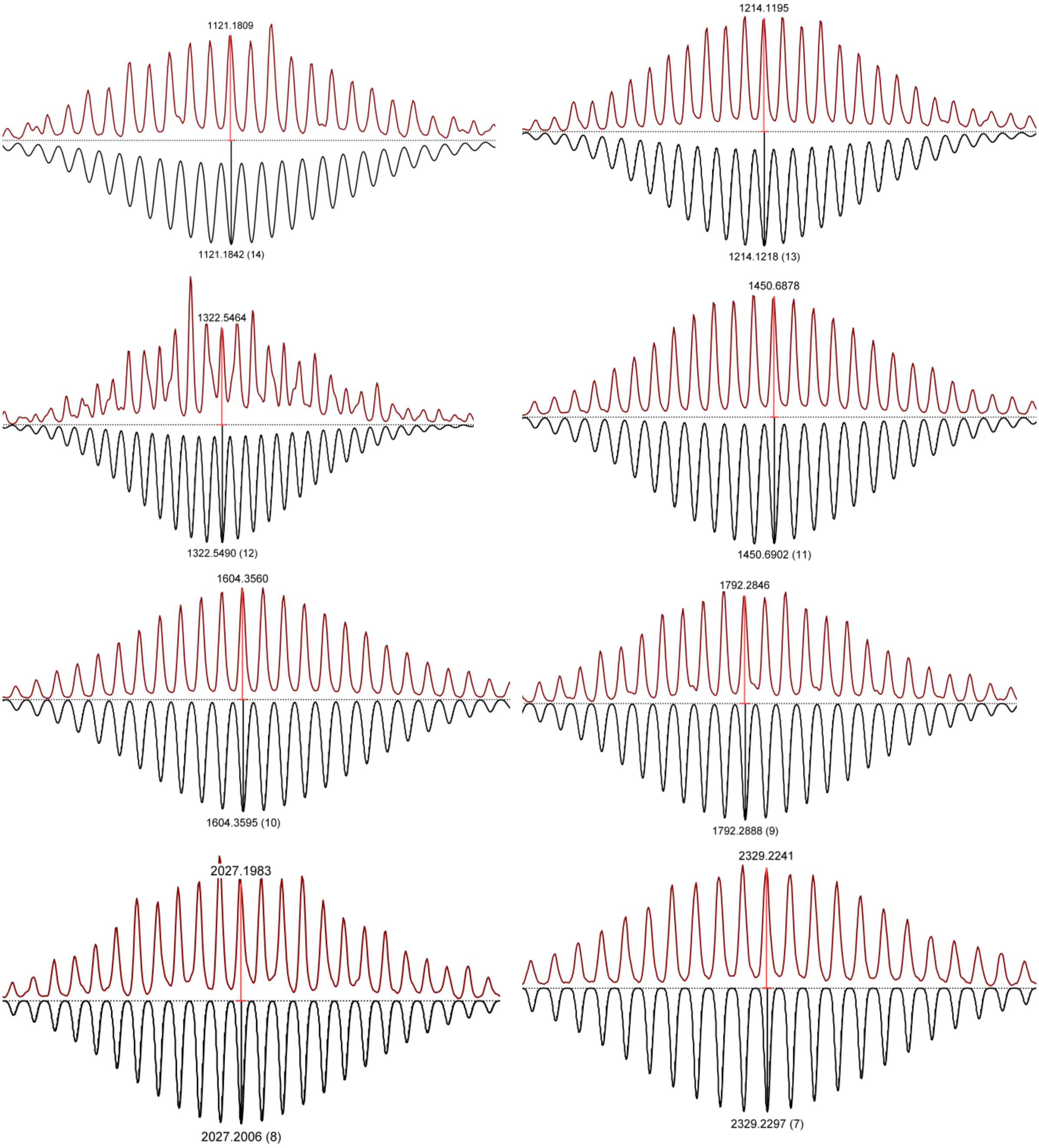

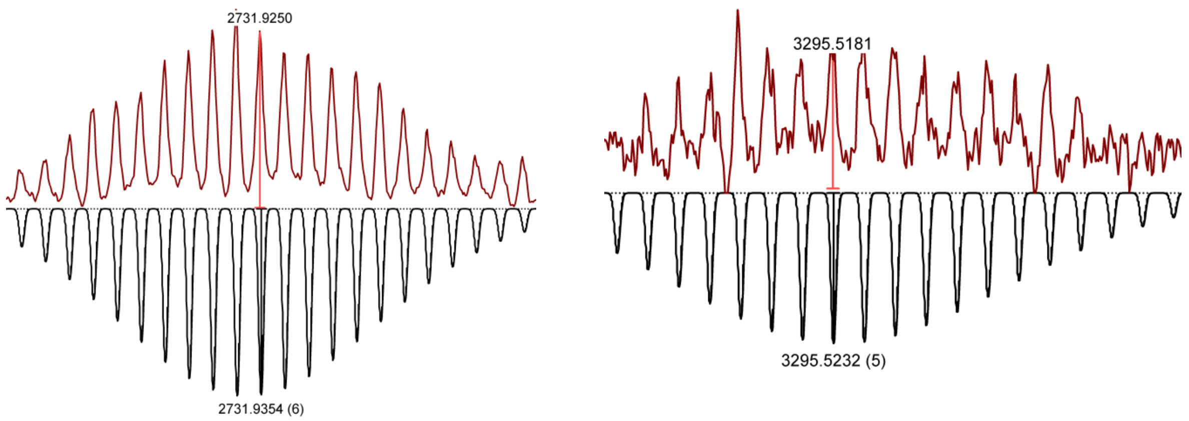

[Pt_{12}(**L**^{Py})$_{24}$]$^{48+}$(BF_4^-)$_{48}$

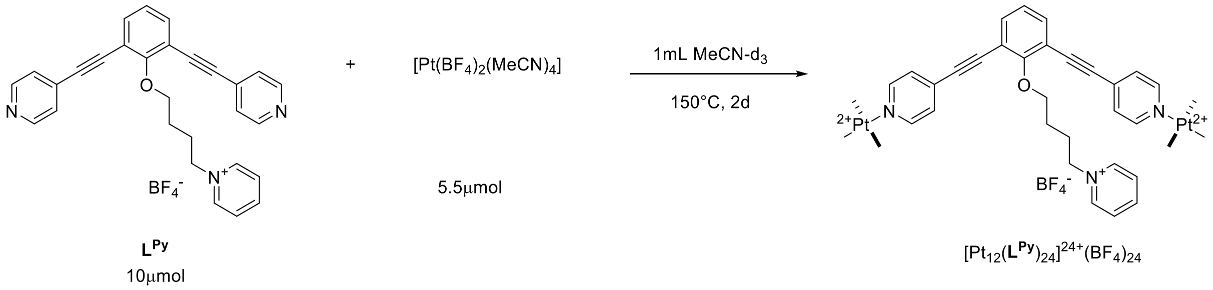

To a solution of **L**^{Py} (5.18 mg, 10 μmol, 1 eq.) in 0.5 mL MeCN-d$_3$, [Pt(BF_4)$_2$(MeCN)$_4$] (2.93 mg, 5.5 μmol. 0.55 eq.) in 0.5 mL MeCN-d$_3$ was added. The solution was then stirred at 150°C for 2d in a 10 mL high pressure tube.^{1}H NMR (300 MHz, Acetonitrile-*d*$_3$) δ 9.00 (d, *J* = 6.3 Hz, 3H), 8.85 (d, *J* = 6.1 Hz, 2H), 8.71 (d, *J* = 6.3 Hz, 1H), 8.55 (d, *J* = 7.9 Hz, 1H), 8.15 – 7.96 (m, 3H), 7.69 (d, *J* = 7.7 Hz, 1H), 7.67 – 7.59 (m, 5H), 7.26 (t, *J* = 7.8 Hz, 1H), 4.72 (d, *J* = 7.6 Hz, 2H), 4.32 (s, 0H). ^{13}C NMR (126 MHz, CD_3CN) δ 161.00, 161.00, 150.08, 145.76, 144.39, 134.90, 130.44, 128.37, 128.32, 125.22, 124.47, 116.75, 91.01, 88.97, 73.86, 73.79, 73.79, 61.41, 28.37, 26.46. LogD(m^2/s) at 25°C (MeCN-d$_3$): -9.544 (d = 4.45 nm).

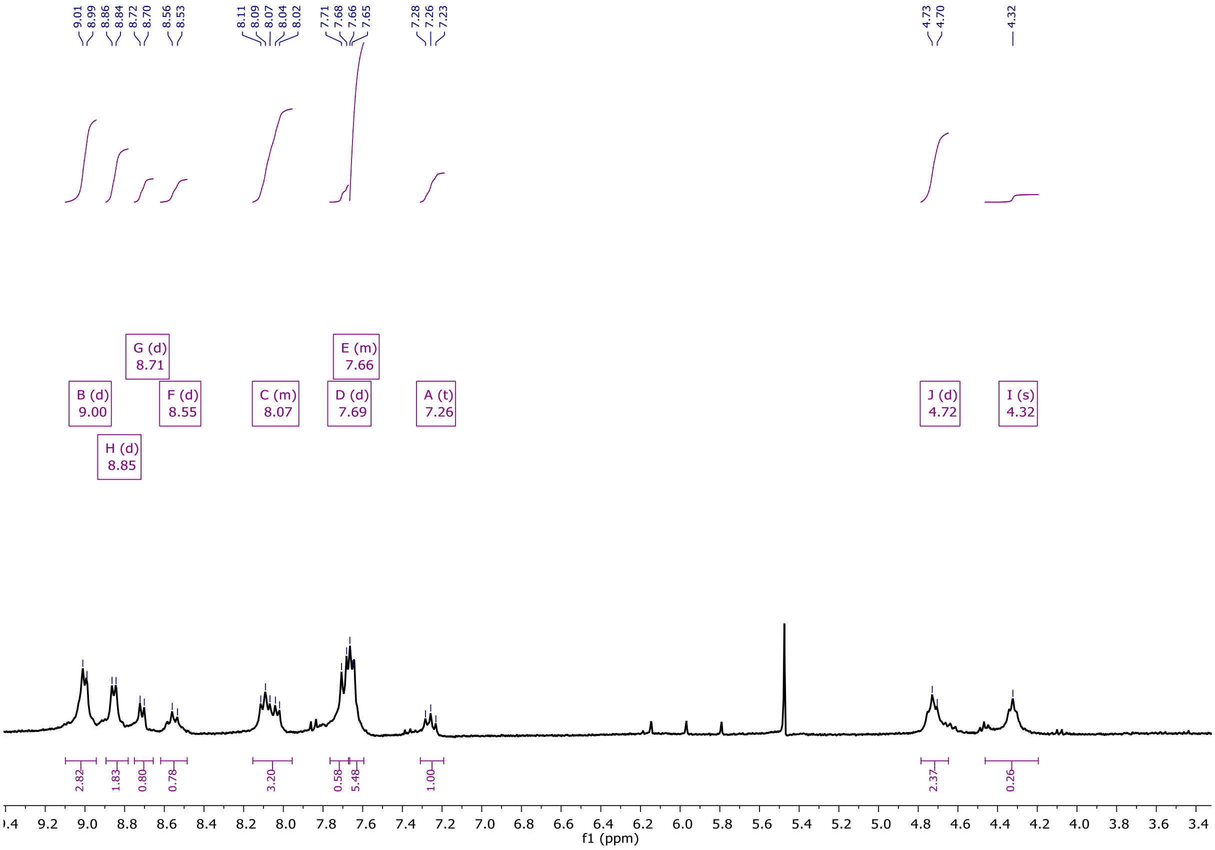

Figure S53. $[Pt_{12}(\mathbf{L^{Py}})_{24}]^{24+}(BF_4^-)_{24}$ sphere, ^1H NMR in MeCN-d_3.

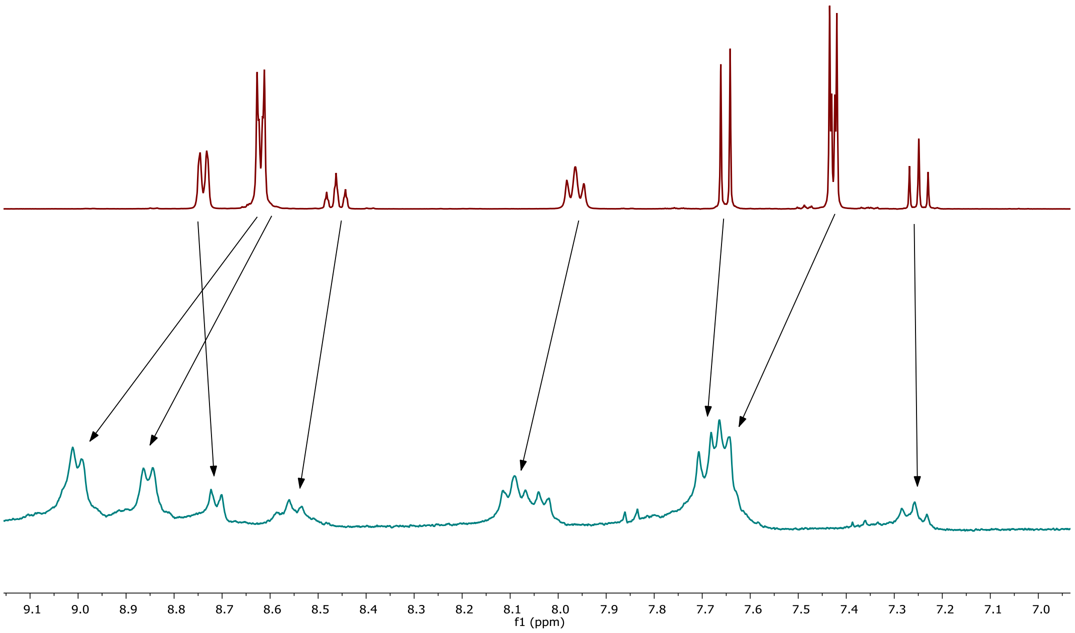

Figure S54. Comparison of free $\mathbf{L^{Py}}$ building block (top) and $[Pt_{12}(\mathbf{L^{Py}})_{24}]^{24+}(BF_4^-)_{24}$ sphere (bottom), ^1H NMR in MeCN-d_3.

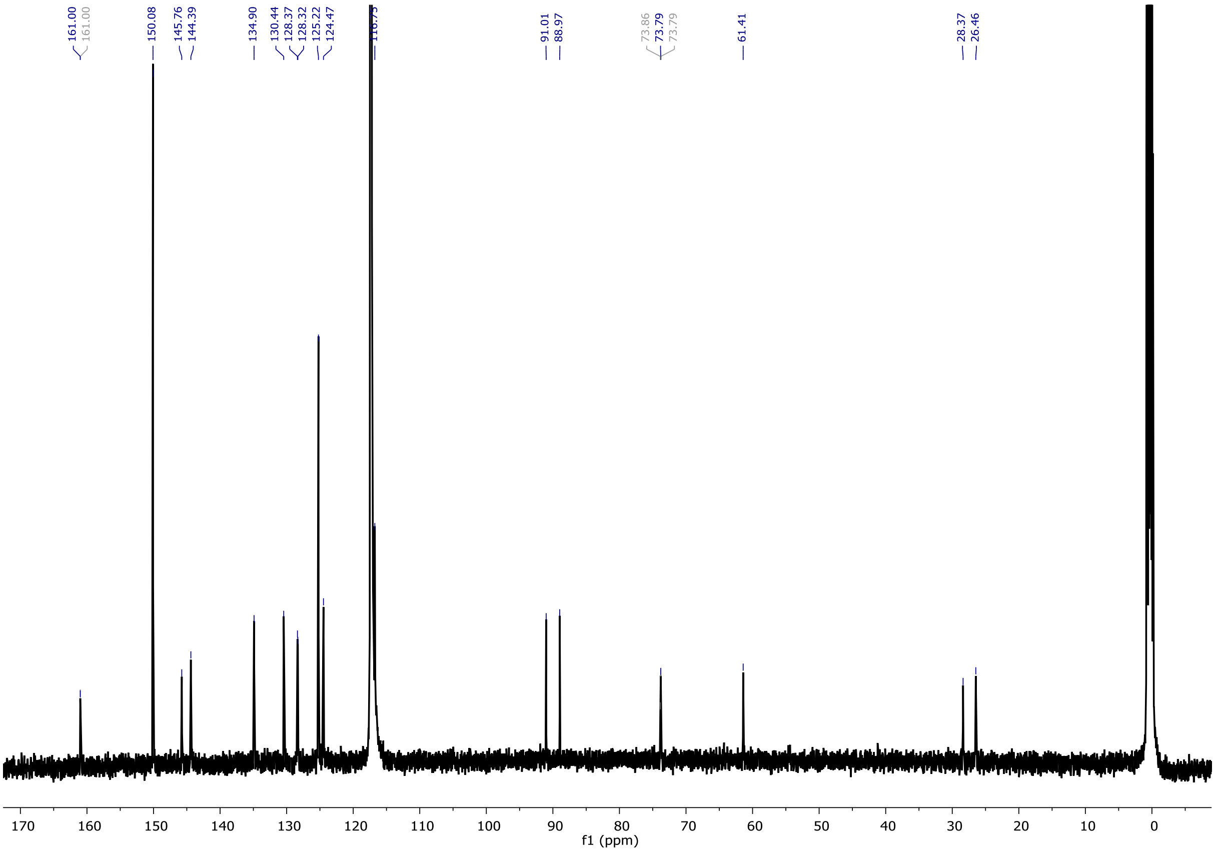

Figure S55. $[Pt_{12}(\mathbf{L^{Py}})_{24}]^{24+}(BF_4^-)_{24}$ sphere, ^{13}C NMR in MeCN-d_3.

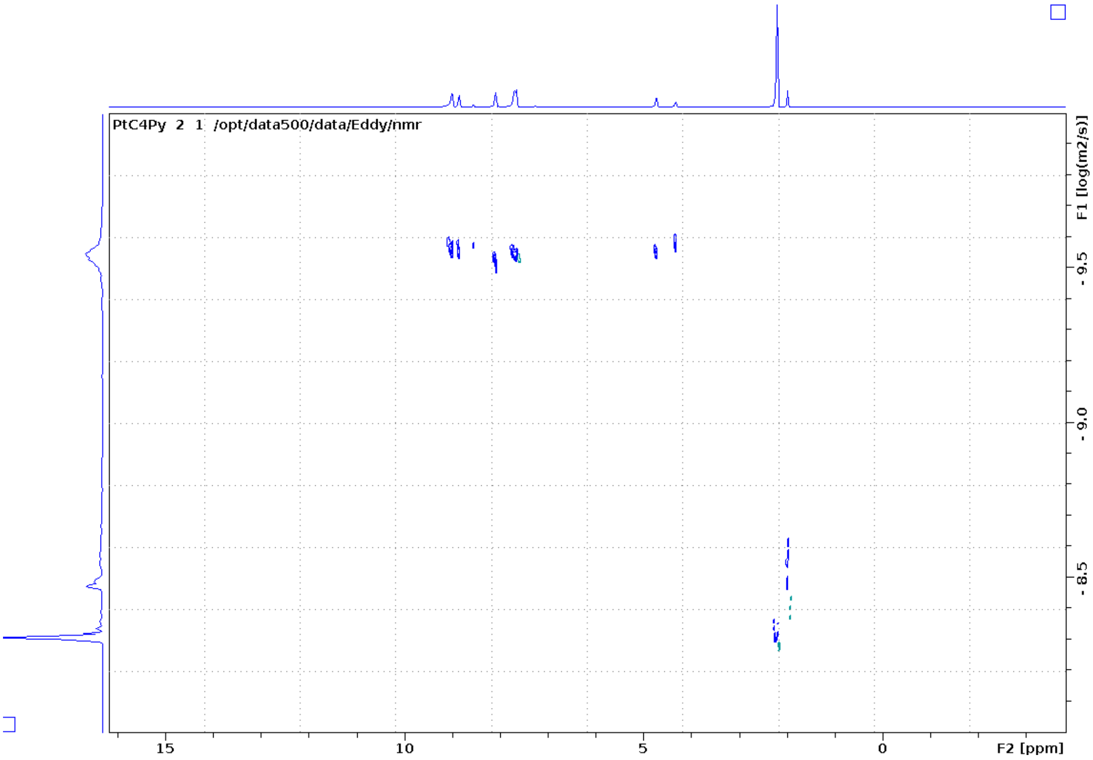

Figure S56. $[Pt_{12}(\mathbf{L^{Py}})_{24}]^{48+}(BF_4^-)_{48}$ cage prepared at 150°C, DOSY in MeCN-d_3 at 25°C.

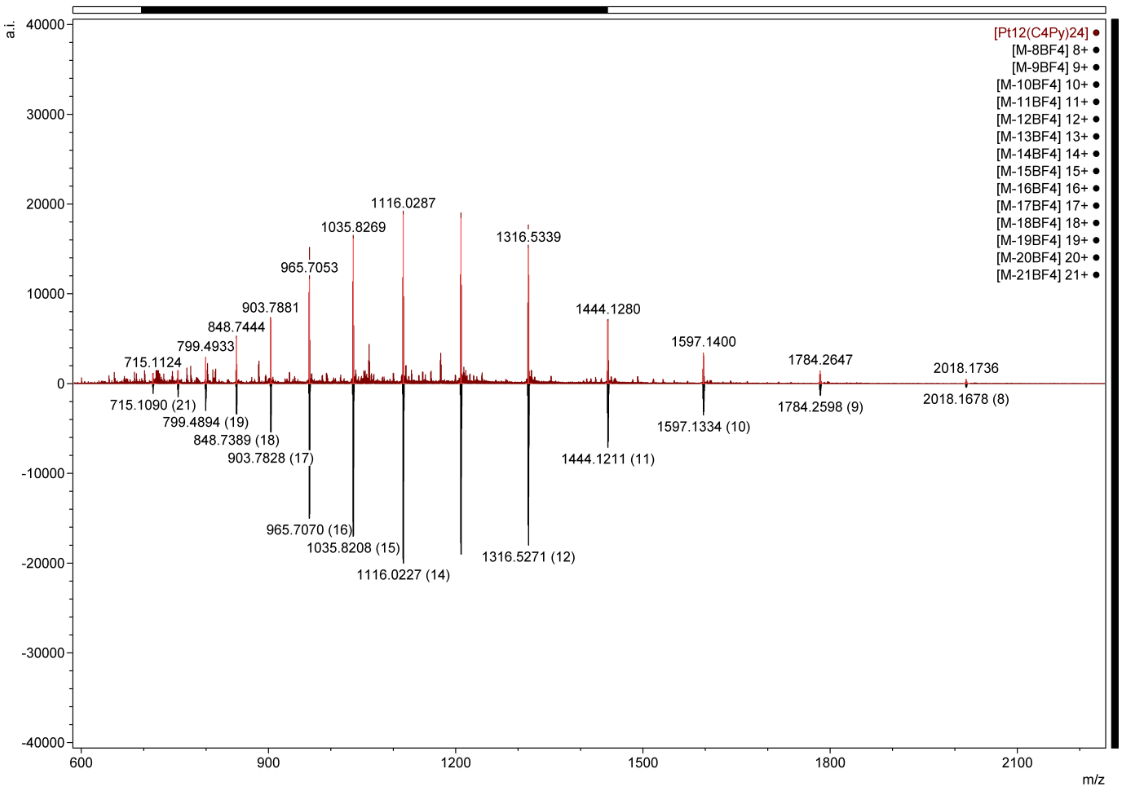

Figure S57. Full ESI-MS spectra of $[Pt_{12}(\mathbf{L^{Py}})_{24}]^{48+}(BF_4^-)_{48}$ prepared at 150°C for 2d. Below the simulated spectra, above obtained spectra.

Table S11. Calculated and observed species of the $[Pt_{12}(\mathbf{L^{Py}})_{24}]^{48+}$ assembly prepared at 150°C for 2d.

Formula	calculated	obtained
$[Pt_{12}(\mathbf{C4Py})_{24}(BF_4^-)_{43}]^{5+}$	3295.5232	3295.5181
$[Pt_{12}(\mathbf{C4Py})_{24}(BF_4^-)_{40}]^{8+}$	2018.1678	2018.1736
$[Pt_{12}(\mathbf{C4Py})_{24}(BF_4^-)_{39}]^{9+}$	1784.2598	1784.2647
$[Pt_{12}(\mathbf{C4Py})_{24}(BF_4^-)_{38}]^{10+}$	1597.1334	1597.1400
$[Pt_{12}(\mathbf{C4Py})_{24}(BF_4^-)_{37}]^{11+}$	1444.1211	1444.1280
$[Pt_{12}(\mathbf{C4Py})_{24}(BF_4^-)_{36}]^{12+}$	1316.5271	1316.5339
$[Pt_{12}(\mathbf{C4Py})_{24}(BF_4^-)_{35}]^{13+}$	1208.5633	1208.5697
$[Pt_{12}(\mathbf{C4Py})_{24}(BF_4^-)_{34}]^{14+}$	1116.0227	1116.0287
$[Pt_{12}(\mathbf{C4Py})_{24}(BF_4^-)_{33}]^{15+}$	1035.8208	1035.8269
$[Pt_{12}(\mathbf{C4Py})_{24}(BF_4^-)_{32}]^{16+}$	965.7070	965.7053
$[Pt_{12}(\mathbf{C4Py})_{24}(BF_4^-)_{31}]^{17+}$	903.7828	903.7881
$[Pt_{12}(\mathbf{C4Py})_{24}(BF_4^-)_{30}]^{18+}$	848.7389	848.7444
$[Pt_{12}(\mathbf{C4Py})_{24}(BF_4^-)_{29}]^{19+}$	799.4898	799.4933
$[Pt_{12}(\mathbf{C4Py})_{24}(BF_4^-)_{28}]^{20+}$	755.1646	755.1655
$[Pt_{12}(\mathbf{C4Py})_{24}(BF_4^-)_{27}]^{21+}$	715.1090	715.1124

Table S12. Zoom into different charged species of $[Pt_{12}(\mathbf{L^{Py}})_{24}]^{24+}$ assembly of a sample prepared at 150°C for 2d.

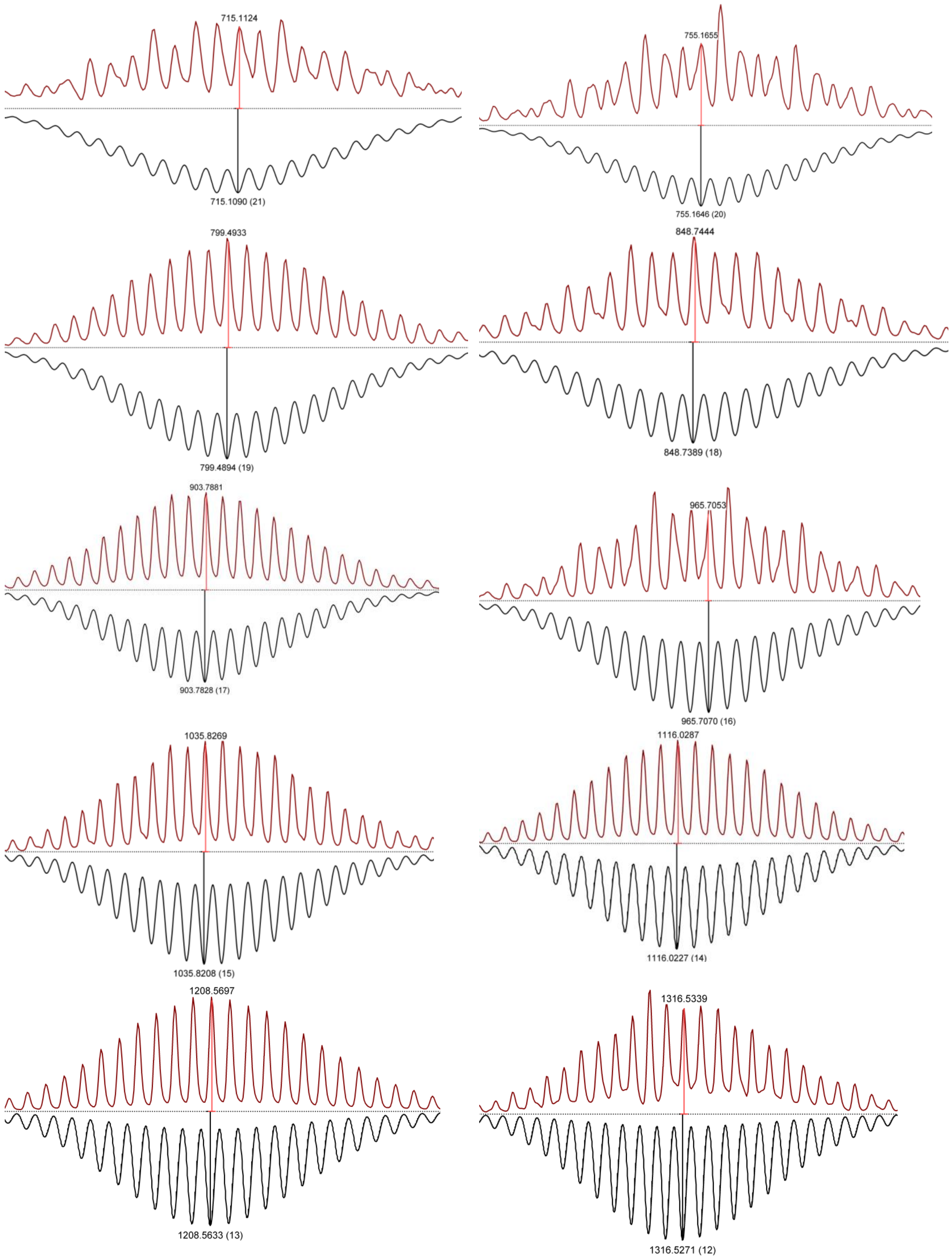

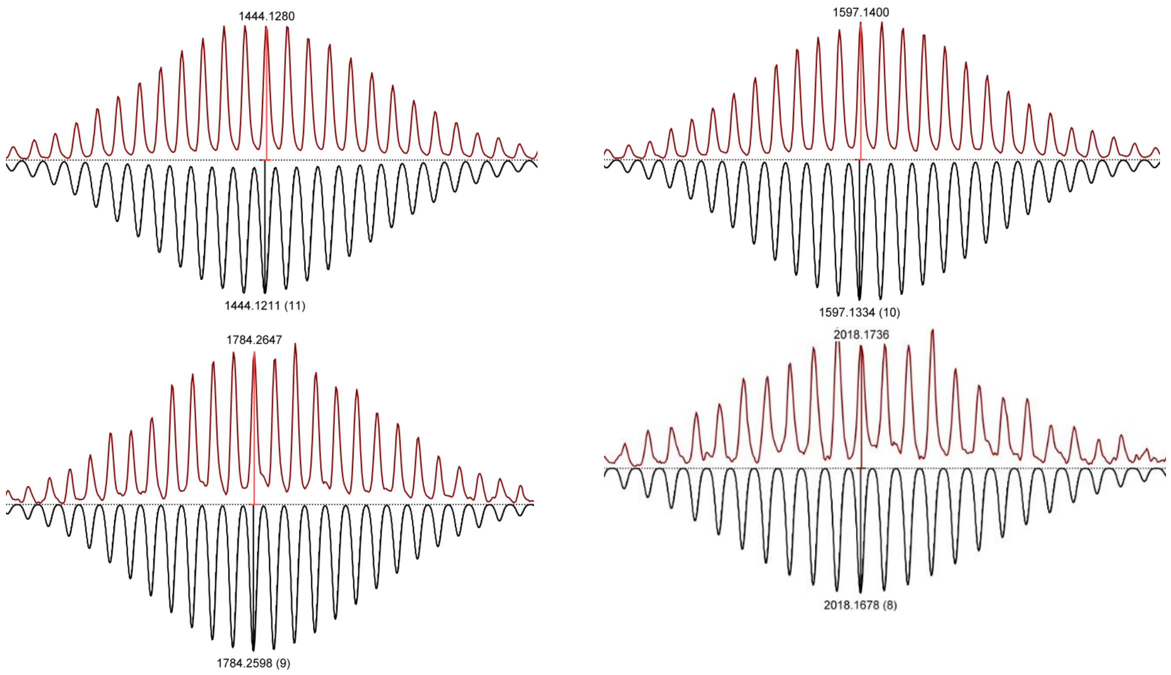

$[Pt_n(\mathbf{L}^{exoPy})_{2n}]^{2n+}(BF_4^-)_{2n}$

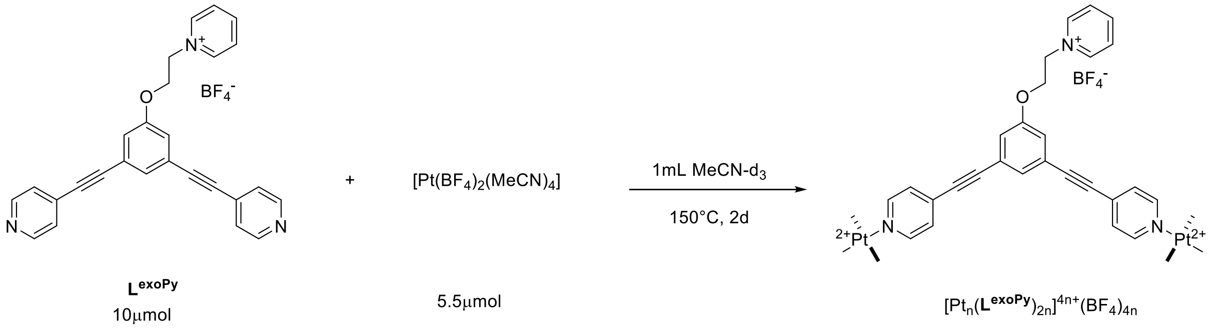

To a solution of $\mathbf{L}^{\mathbf{exoPy}}$ (4.90 mg, 10 μmol, 1 eq.) in 0.5 mL MeCN-d3, $[Pt(BF_4)_2(MeCN)_4]$ (2.93 mg, 5.5 μmol. 0.55 eq.) in 0.5 mL MeCN-d3 was added. The solution was then stirred at 150°C for 2d in a 10 mL high pressure tube. ^1H NMR (300 MHz, Acetonitrile-*d*3) δ 8.94 (s, 6H), 8.69 – 8.40 (m, 1H), 8.07 (s, 2H), 7.63 (d, *J* = 35.7 Hz, 7H), 7.29 (s, 1H), 5.06 (s, 2H), 4.82 (s, 2H).

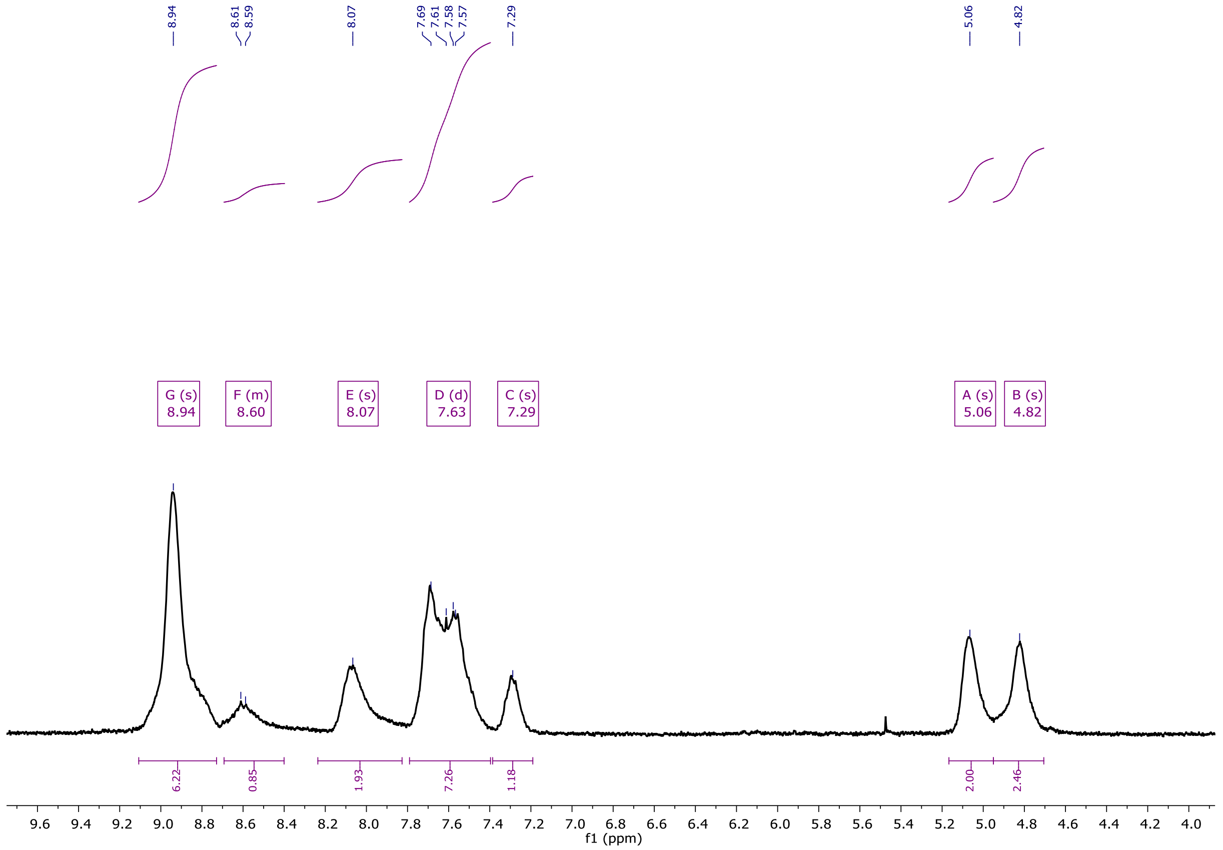

Figure S58. [Pt$_n$(**L**^{exoPy})$_{2n}$]$^{4n+}$(BF$_4^-$)$_{4n}$ sphere, ^{1}H NMR in MeCN-d$_3$.

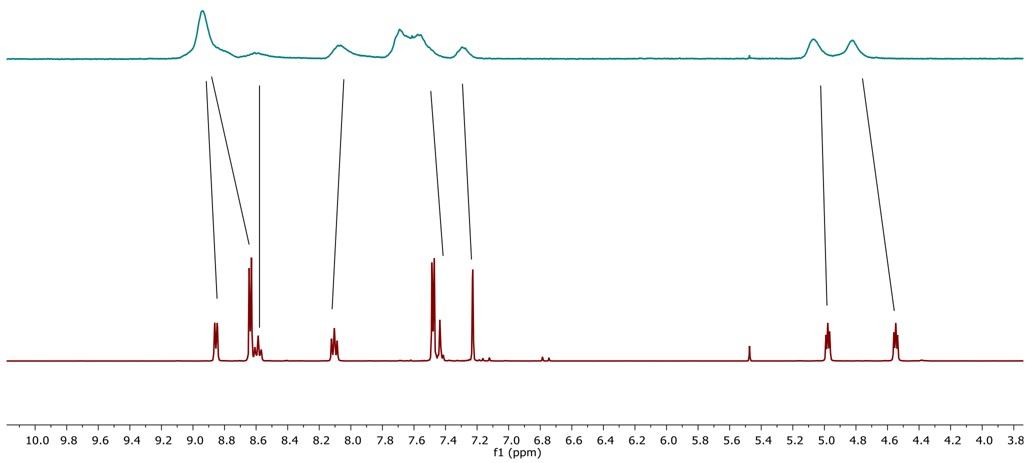

Figure S59. Comparison of free **L**^{exoPy} building block (bottom) and [Pt$_n$(**L**^{exoPy})$_{2n}$]$^{4n+}$(BF$_4^-$)$_{4n}$ sphere (top), ^{1}H NMR in MeCN-d$_3$.

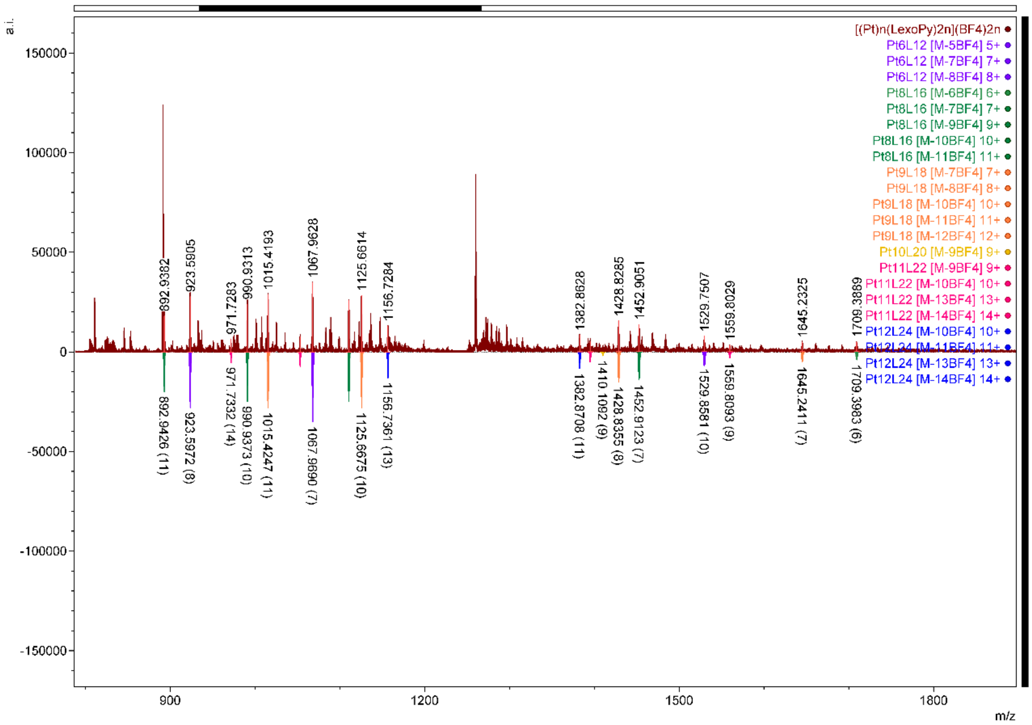

Figure S60. Full ESI-MS spectra of $[Pt_n(\mathbf{L}^{exoPy})_{2n}]^{4n+}(BF_4^-)_{4n}$ prepared at 150°C for 2d. Below the simulated spectra of different sphere types, above obtained spectra.

Table S13. Calculated and observed species of the $[Pt_n(\mathbf{L}^{exopy})_{2n}]^{2n+}$ assemblies of a sample prepared at 150°C for 2d.

Cage Type	Composition	Calculated	Found
M_6L_{12}	$[Pt_6(\mathbf{L}^{exoPy})_{12}(BF_4^-)_{16}]^{8+}$	923.5972	923.5905
	$[Pt_6(\mathbf{L}^{exoPy})_{12}(BF_4^-)_{17}]^{7+}$	1067.9690	1067.9628
	$[Pt_6(\mathbf{L}^{exoPy})_{12}(BF_4^-)_{19}]^{5+}$	1529.7581	1529.7507
M_8L_{16}	$[Pt_8(\mathbf{L}^{exoPy})_{16}(BF_4^-)_{21}]^{11+}$	892.9426	892.9382
	$[Pt_8(\mathbf{L}^{exoPy})_{16}(BF_4^-)_{22}]^{10+}$	990.9373	990.9313
	$[Pt_8(\mathbf{L}^{exoPy})_{16}(BF_4^-)_{23}]^{9+}$	1110.7086	1110.7025
	$[Pt_8(\mathbf{L}^{exoPy})_{16}(BF_4^-)_{25}]^{7+}$	1452.9123	1452.9051
	$[Pt_8(\mathbf{L}^{exoPy})_{16}(BF_4^-)_{26}]^{6+}$	1709.3983	1709.3889
M_9L_{18}	$[Pt_9(\mathbf{L}^{exoPy})_{18}(BF_4^-)_{25}]^{11+}$	1015.4247	1015.4193
	$[Pt_9(\mathbf{L}^{exoPy})_{18}(BF_4^-)_{26}]^{10+}$	1125.6675	11.25.6614
	$[Pt_9(\mathbf{L}^{exoPy})_{18}(BF_4^-)_{28}]^{8+}$	1428.8355	1428.8285
	$[Pt_9(\mathbf{L}^{exoPy})_{18}(BF_4^-)_{29}]^{7+}$	1645.2411	1645.2325
$M_{11}L_{22}$	$[Pt_{11}(\mathbf{L}^{exoPy})_{22}(BF_4^-)_{32}]^{14+}$	971.7332	971.7283
	$[Pt_{11}(\mathbf{L}^{exoPy})_{22}(BF_4^-)_{31}]^{13+}$	1053.0974	1053.0922
	$[Pt_{11}(\mathbf{L}^{exoPy})_{22}(BF_4^-)_{34}]^{10+}$	1395.1279	1395.1235
	$[Pt_{11}(\mathbf{L}^{exoPy})_{22}(BF_4^-)_{35}]^{9+}$	1559.8093	1559.8029
$M_{12}L_{24}$	$[Pt_{12}(\mathbf{L}^{exoPy})_{24}(BF_4^-)_{35}]^{13+}$	1156.7361	1156.7284
	$[Pt_{12}(\mathbf{L}^{exoPy})_{24}(BF_4^-)_{37}]^{11+}$	1382.8708	1382.8628

Table S14. Zoom into different charged species of $[Pt_n(\mathbf{L}^{exoPy})_{2n}]^{2n+}$ assemblies of a sample prepared at 150°C for 2d.

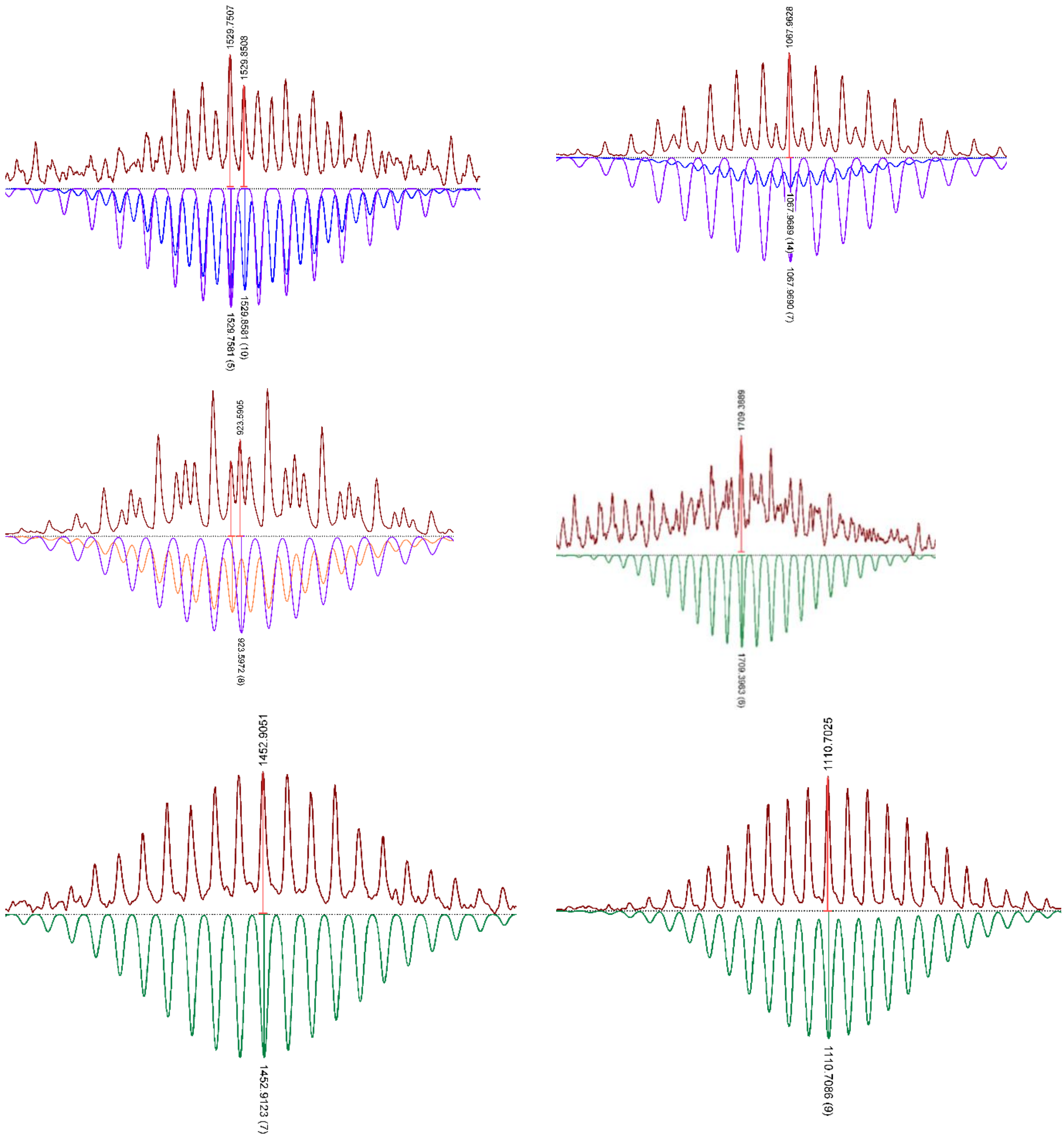

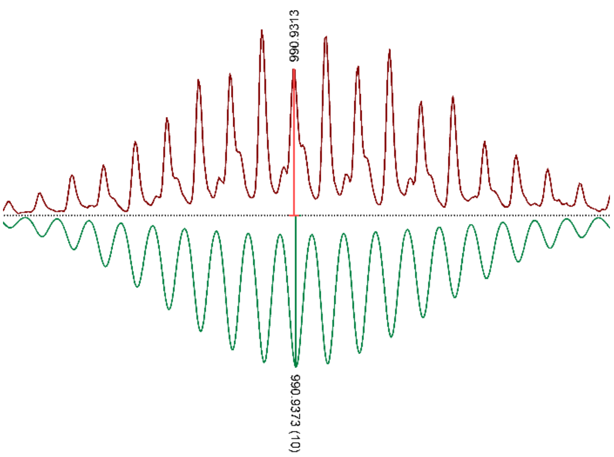
990.9313
990.9373 (10)

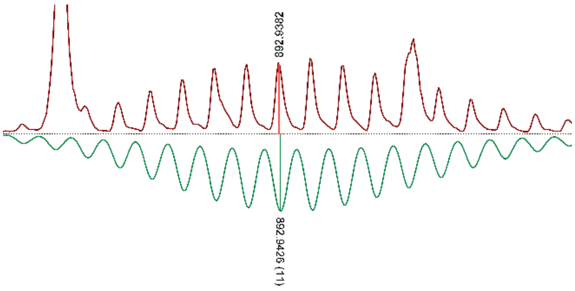
892.9382
892.9426 (11)

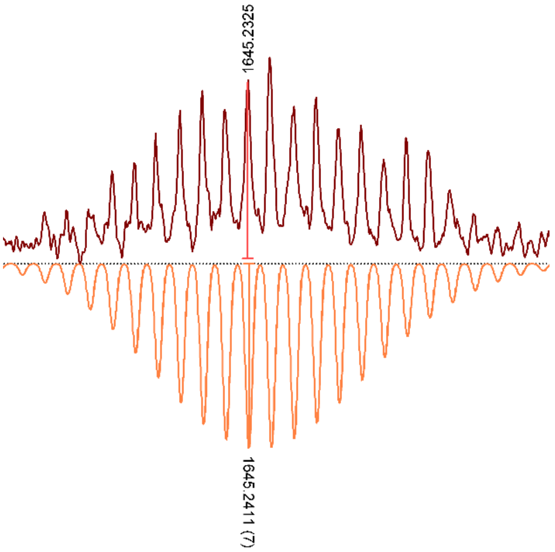
1645.2325
1645.2411 (7)

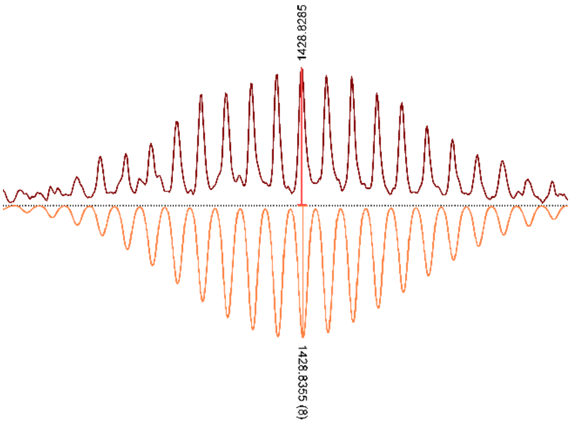
1428.8285
1428.8355 (8)

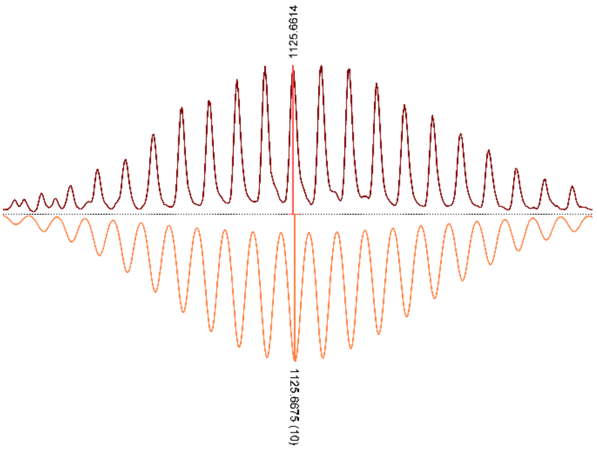
1125.6614
1125.6675 (10)

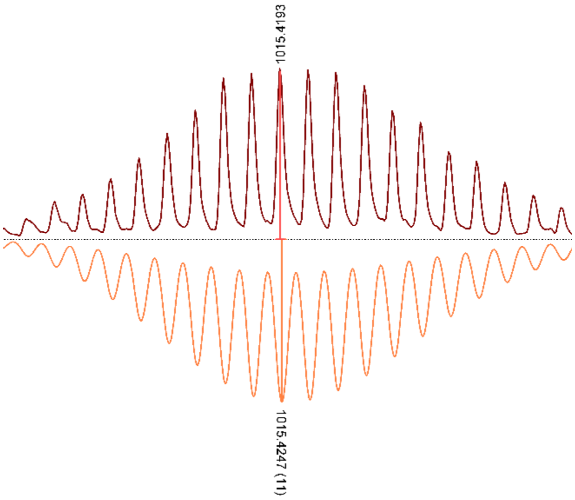
1015.4193
1015.4247 (11)

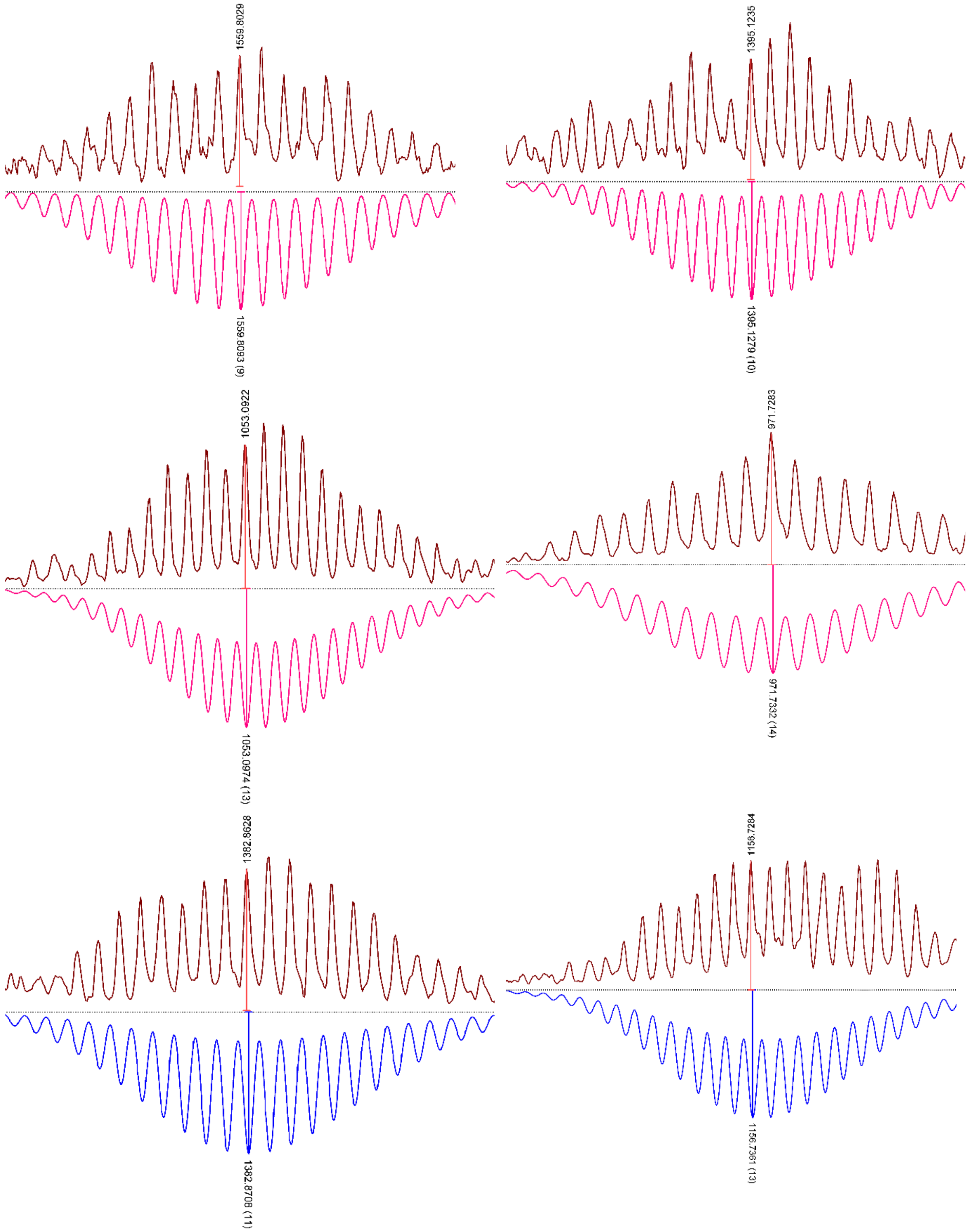
1559.8029
1559.8093 (9)
1395.1235
1395.1279 (10)
1053.0922
1053.0974 (13)
971.7283
971.7332 (14)
1382.8628
1382.8708 (11)
1156.7284
1156.7361 (13)

[$Pt_{12}(\mathbf{L}^{\mathbf{ImiAd}})_{24}]^{48+}(BF_4^-)_{48}$

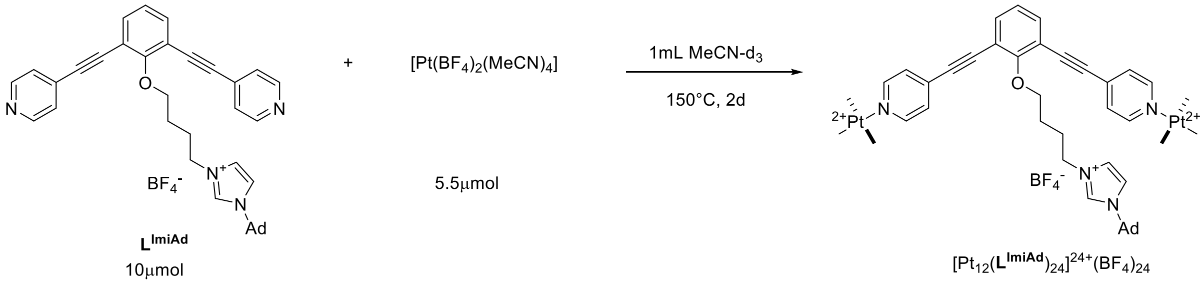

To a solution of $\mathbf{L}^{\mathbf{ImiAd}}$ (6.40 mg, 10 μmol, 1 eq.) in 0.5 mL MeCN-d3, $[Pt(BF_4)_2(MeCN)_4]$ (2.93 mg, 5.5 μmol. 0.55 eq.) in 0.5 mL MeCN-d3 was added. The solution was then stirred at 150°C for 2d in a 10 mL high pressure tube. ^1H NMR (300 MHz, Acetonitrile-*d*3) δ 9.00 (m, 3H), 8.64 (s, 1H), 7.58 (m, 8H), 7.25 (m, 1H), 4.29 (m, 4H). LogD(m^2/s) at 25°C (MeCN-d3): -9.482 (d = 4.45 nm).

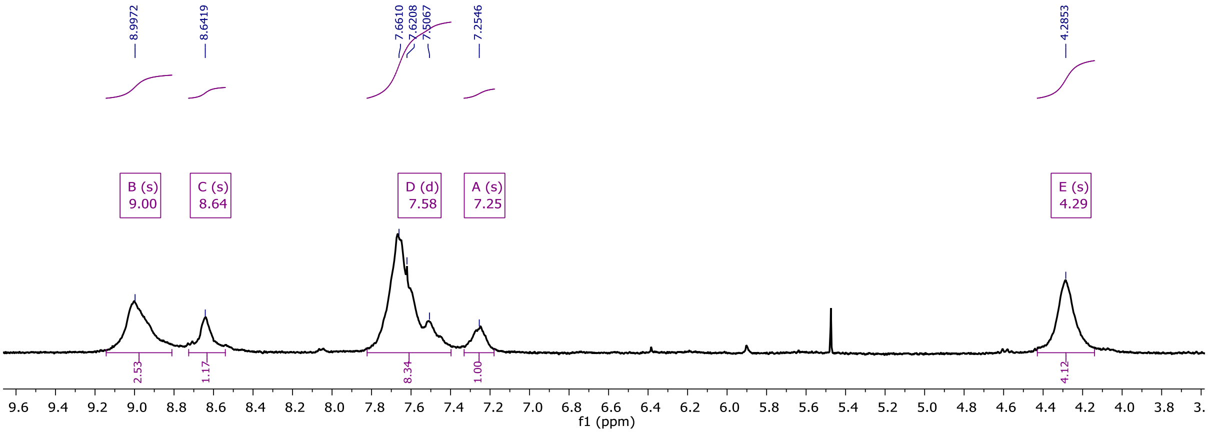

Figure S61. [$Pt_{12}(\mathbf{L}^{\mathbf{ImiAd}})_{24}]^{48+}(BF_4^-)_{48}$ sphere, ^1H NMR in MeCN-d3.

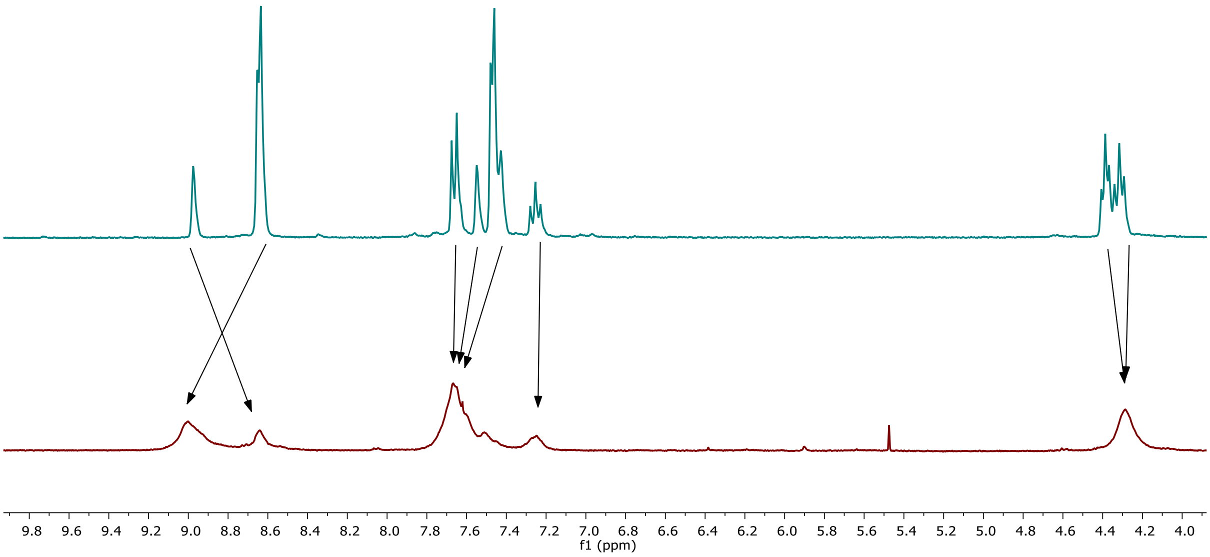

Figure S62. Comparison of free $\mathbf{L}^{\mathbf{ImiAd}}$ building block (top) and [$Pt_{12}(\mathbf{L}^{\mathbf{ImiAd}})_{24}]^{48+}(BF_4^-)_{48}$ sphere (bottom), ^1H NMR in MeCN-d3.

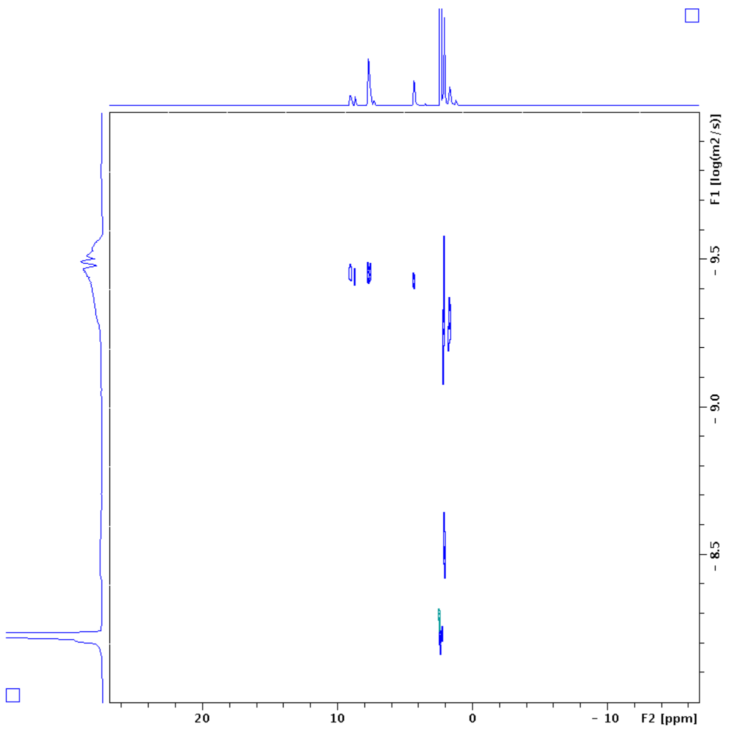

Figure S63. $[Pt_{12}(\mathbf{L}^{\mathbf{ImiAd}})_{24}]^{48+}(BF_4^-)_{24}$ cage prepared at 150°C, DOSY in MeCN-d_3 at 25°C.

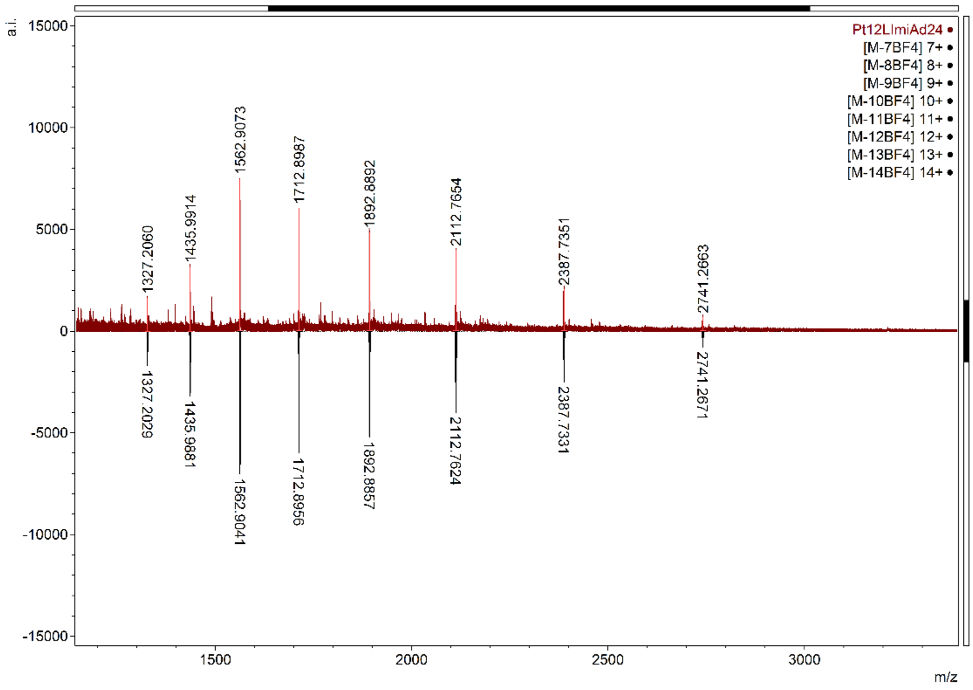

Figure S64. Full ESI-MS spectra of $[Pt_{12}(\mathbf{L}^{\mathbf{ImiAd}})_{24}]^{48+}(BF_4^-)_{24}$ prepared at 150°C for 2d. Below the simulated spectra, above obtained spectra.

Table S15. Calculated and observed species of the $[Pt_{12}(\mathbf{L^{ImiAd}})_{24}]^{48+}$ assembly prepared at 150°C for 2d.

Formula	calculated	obtained
$[Pt_{12}(\mathbf{L^{ImiAd}})_{24}(BF_4^-)_{41}]^{7+}$	2741.2671	2741.2663
$[Pt_{12}(\mathbf{L^{ImiAd}})_{24}(BF_4^-)_{40}]^{8+}$	2387.7331	2387.7351
$[Pt_{12}(\mathbf{L^{ImiAd}})_{24}(BF_4^-)_{39}]^{9+}$	2112.7624	2112.7654
$[Pt_{12}(\mathbf{L^{ImiAd}})_{24}(BF_4^-)_{38}]^{10+}$	1892.8857	1892.8892
$[Pt_{12}(\mathbf{L^{ImiAd}})_{24}(BF_4^-)_{37}]^{11+}$	1712.8956	1712.8987
$[Pt_{12}(\mathbf{L^{ImiAd}})_{24}(BF_4^-)_{36}]^{12+}$	1562.9041	1562.9073
$[Pt_{12}(\mathbf{L^{ImiAd}})_{24}(BF_4^-)_{35}]^{13+}$	1435.9881	1435.9914
$[Pt_{12}(\mathbf{L^{ImiAd}})_{24}(BF_4^-)_{34}]^{14+}$	1327.2029	1327.2060

Table S16. Zoom into different charged species of $[Pt_{12}(\mathbf{L^{ImiAd}})_{24}]^{48+}$ assembly of a sample prepared at 150°C for 2d.

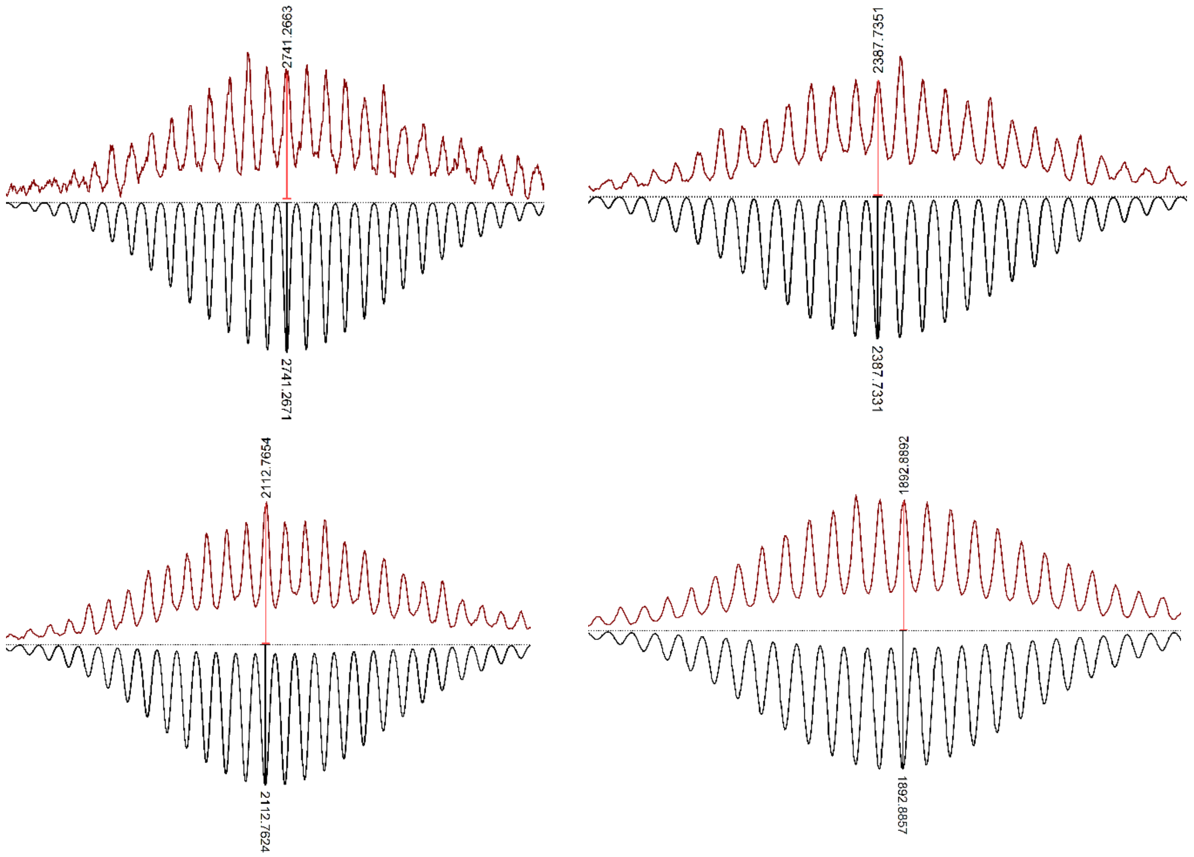

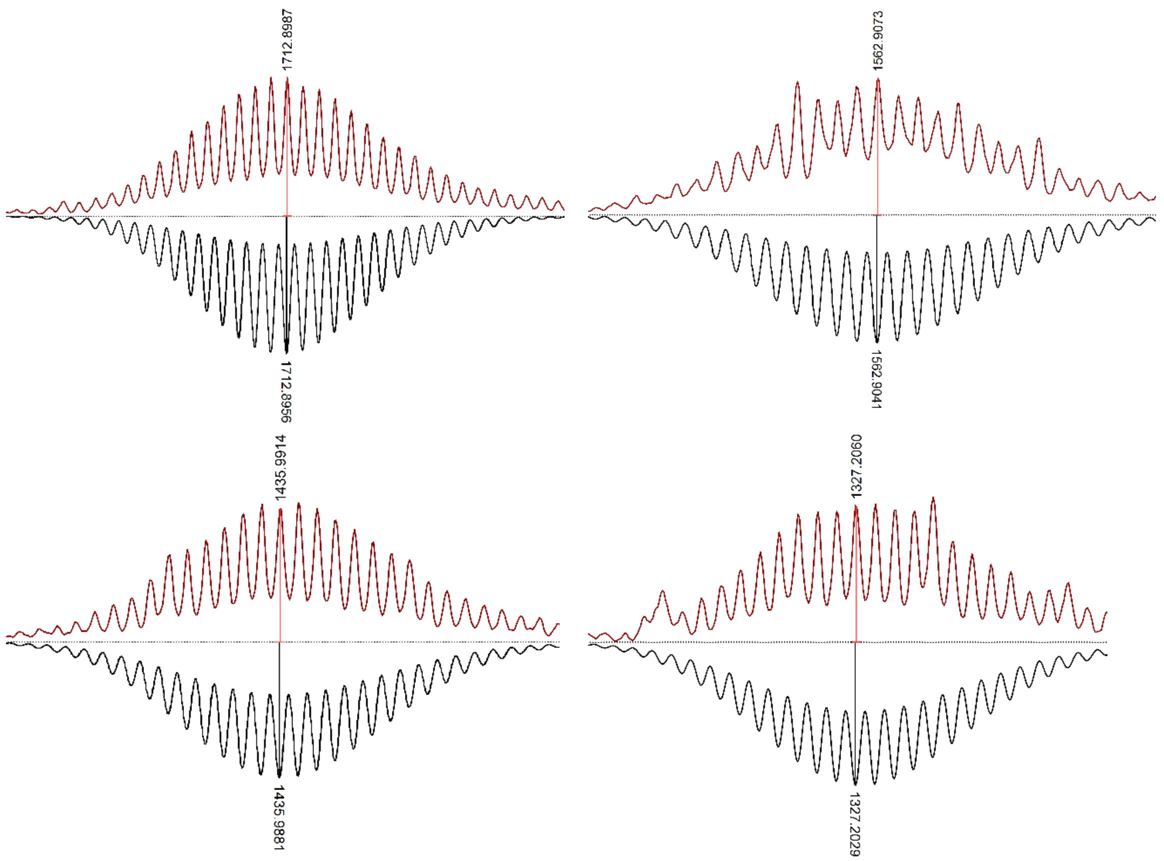

Variable temperature Studies (SI3)

$[Pt_n(\mathbf{L}^{OMe})_{2n}]^{2n+}(BF_4^-)_{2n}$

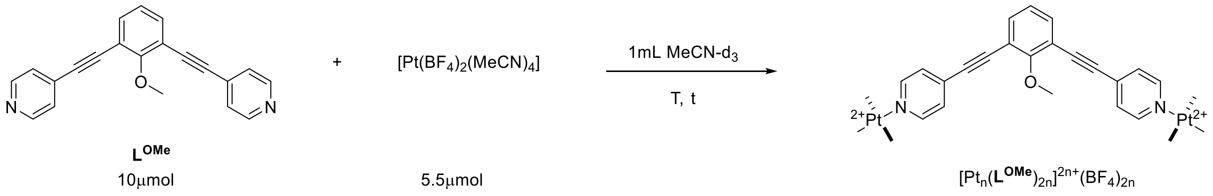

Multiple samples containing a solution of $\mathbf{L}^{OMe}$ (3.1 mg, 10 µmol, 1 eq.) in 0.5 mL MeCN-d_3 and $[Pt(BF_4)_2(MeCN)_4]$ (2.93 mg, 5.5 µmol. 0.55 eq.) in 0.5 mL MeCN-d_3 were prepared. The solutions were then stirred at noted temperature for 24h or longer in 10 mL high pressure tubes. At different time points, the single solutions were taken out and measured with ^1H NMR, DOSY and MS. Exemplary ^1H NMR spectra are displayed in the main text.

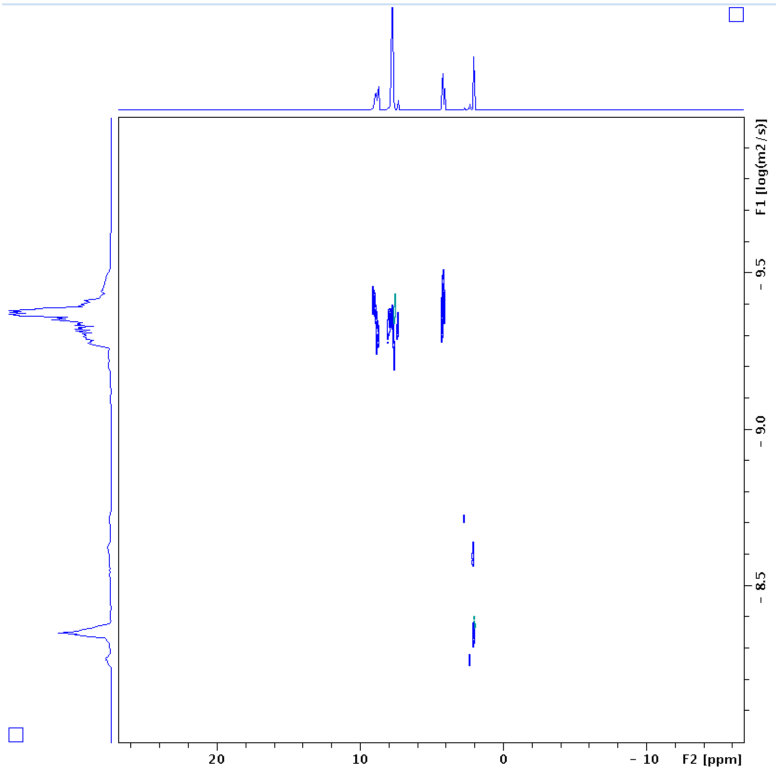

Figure S65. $[Pt_n(\mathbf{L}^{\mathbf{OMe}})_{2n}]^{2n+}(BF4^-)_{2n}$ cage prepared at 25°C, DOSY in MeCN-d$_3$ at 25°C.

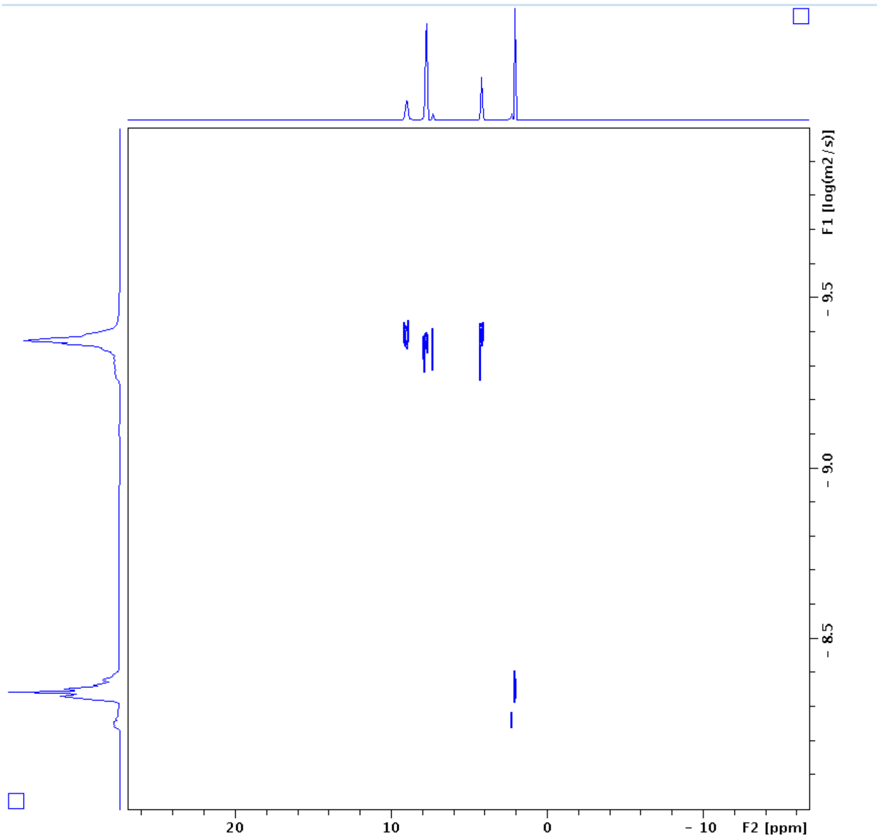

Figure S66. $[Pt_n(\mathbf{L}^{\mathbf{OMe}})_{2n}]^{2n+}(BF4^-)_{2n}$ cage prepared at 85°C, DOSY in MeCN-d$_3$ at 25°C.

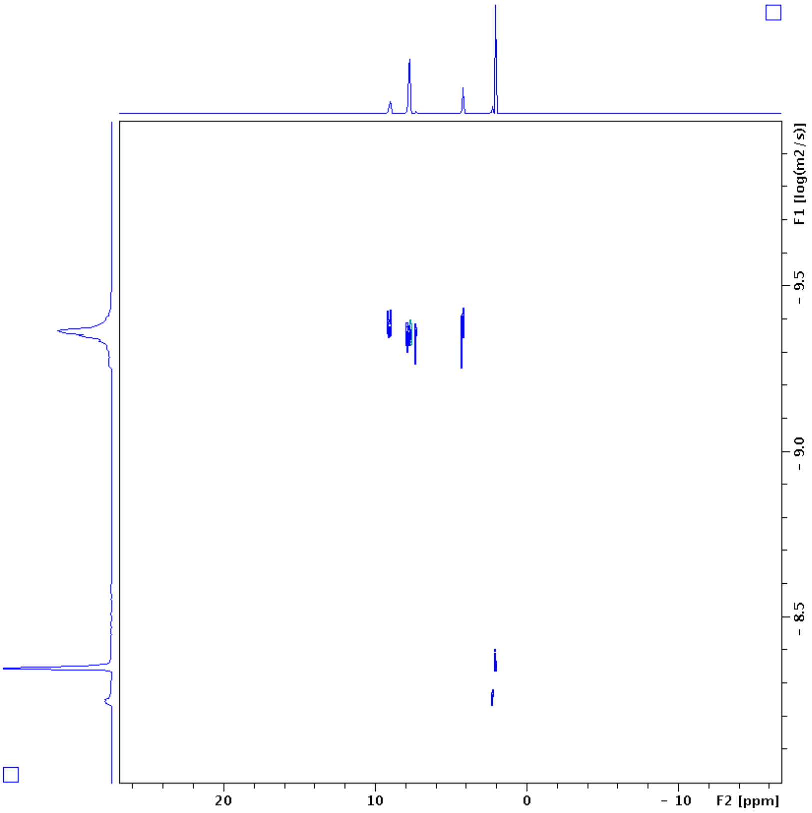

Figure S67. [Pt_n(**L^{OMe}**)$_{2n}$]$^{2n+}$($BF4^-$)$_{2n}$ cage prepared at 110°C, DOSY in MeCN-d_3 at 25°C.

MS analysis was performed on undiluted samples using the same parameters for each solution. The distributions were calculated using the counts for high intensity unique peaks assigned for the individual assemblies as follows:

Table S17. Assigned unique signals of [Pt_n(**L^{OMe}**)$_{2n}$]$^{2n+}$ assemblies whose development was followed over a range of temperature and time.

Structure	Unique signal	M/z
[Pt_6(**L^{OMe}**)$_{12}$]	[Pt_6(**L^{OMe}**)$_{12}$(BF_4^-)$_7$]$^{5+}$	1100*
[Pt_7(**L^{OMe}**)$_{14}$]	[Pt_7(**L^{OMe}**)$_{14}$(BF_4^-)$_9$]$^{5+}$	1297
[Pt_8(**L^{OMe}**)$_{16}$]	[Pt_8(**L^{OMe}**)$_{16}$(BF_4^-)$_9$]$^{7+}$	1043
[Pt_9(**L^{OMe}**)$_{18}$]	[Pt_9(**L^{OMe}**)$_{18}$(BF_4^-)$_{11}$]$^{7+}$	1185
[Pt_{10}(**L^{OMe}**)$_{20}$]	[Pt_{10}(**L^{OMe}**)$_{20}$(BF_4^-)$_{11}$]$^{9+}$	1012
[Pt_{11}(**L^{OMe}**)$_{22}$]	[Pt_{11}(**L^{OMe}**)$_{22}$(BF_4^-)$_{13}$]$^{9+}$	1122
[Pt_{12}(**L^{OMe}**)$_{24}$]	[Pt_{12}(**L^{OMe}**)$_{24}$(BF_4^-)$_{13}$]$^{11+}$	992

*due to an overlap with the 10+ signal of the $M_{12}L_{24}$ assembly, the difference between both assemblies was considered.

The obtained individual values were then divided by the total amount of counts obtained by adding all individual counts together and multiplied by 100 to obtain the fraction of the individual spheres. A baseline correction was not performed prior to analysis.

Deconvolution of the investigated peaks was not necessary because they represent unique signals of individual assemblies (only for the peak at 1100, intensity of $M_{12}L_{24}$ assembly was subtracted from the observed value for the M_6L_{12} species).

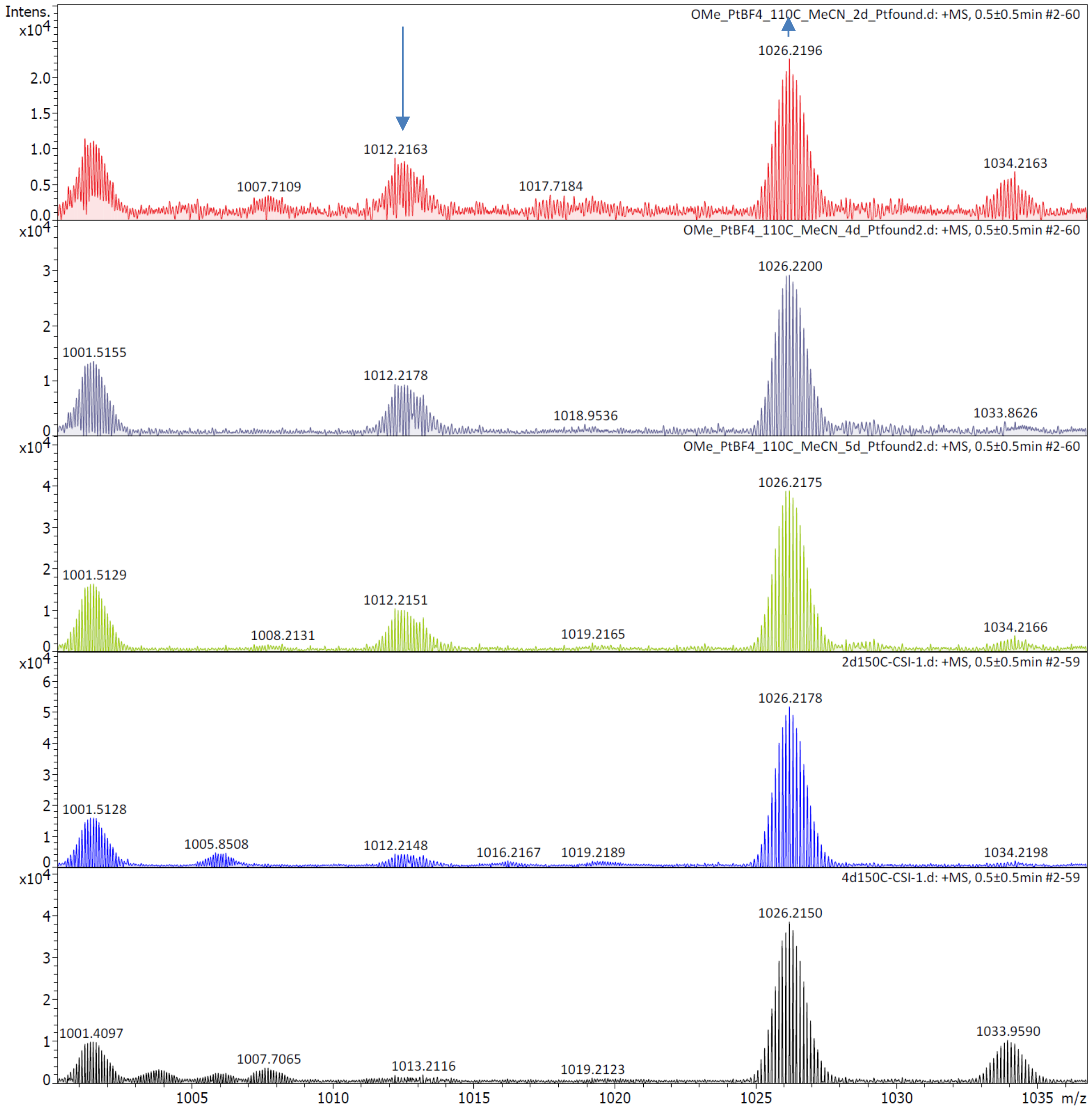

Figure S68. Example of collected MS spectra at different stages of self-assembly showing the disappearance of the $[Pt_{10}(\mathbf{L}^{OMe})_{20}]^{9+}$ signal at 1012 Da with increasing temperature and time and the increase of the $[Pt_9(\mathbf{L}^{OMe})_{18}]^{8+}$ signal at 1026 Da.

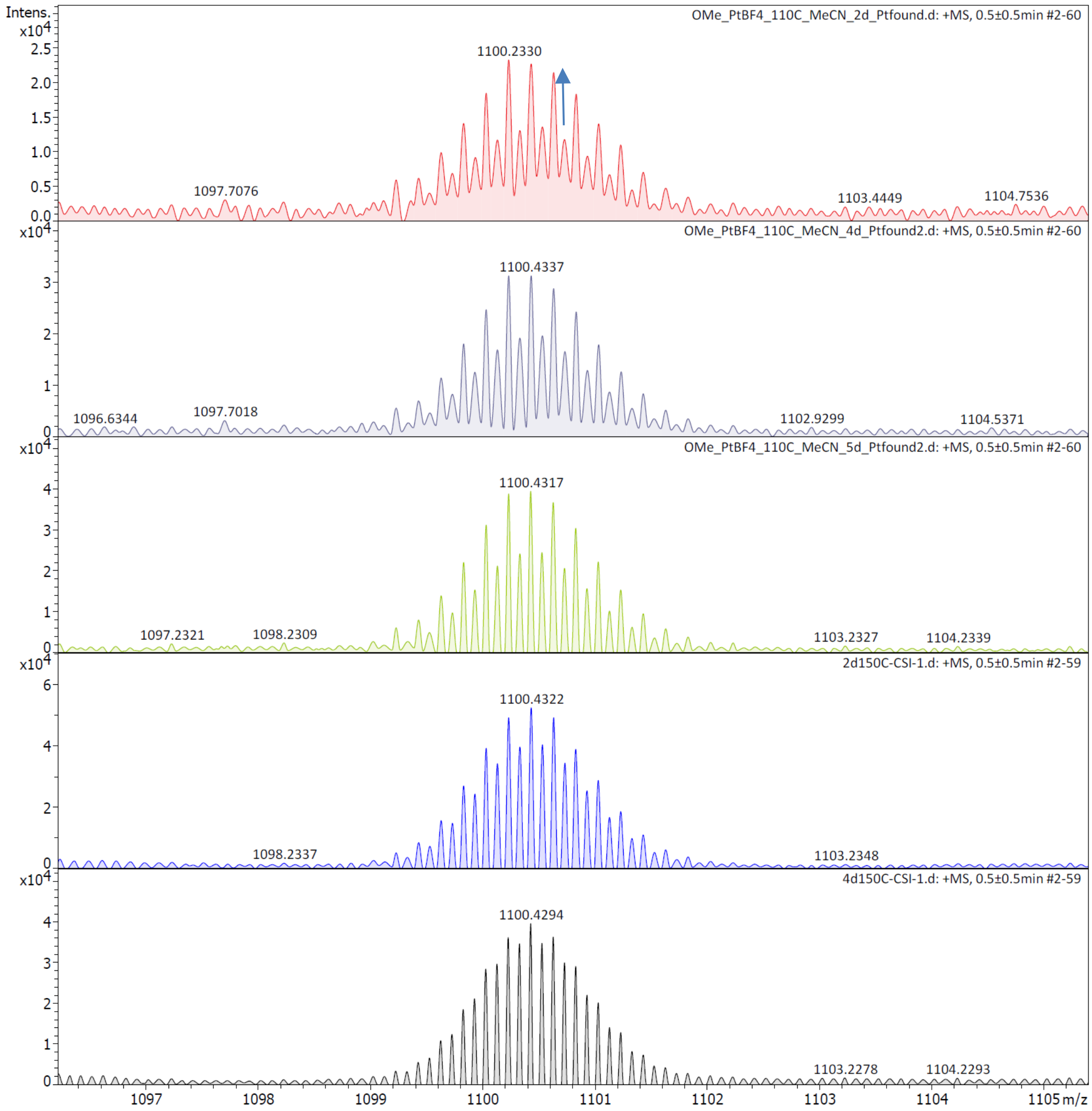

Figure S69. Example of collected MS spectra at different stages of self-assembly showing the increase of the $[Pt_{12}(\mathbf{L^{OMe}})_{24}]^{10+}$ signal at 1100 Da with increasing temperature and time and the decrease of the $[Pt_6(\mathbf{L^{OMe}})_{12}]^{5+}$ signal.

Table S18. Amount of counts of the $[Pt_{12}(\mathbf{L^{OMe}})_{24}]^{11+}$ signal over a range of temperature and time.

T	counts $[Pd_{12}L_{24}]^{11+}$ 992Da
70°C-1d	0
85°C-3d	4848
110°C-3d	15462
130°C-3d	33775
150°C-3d	56446
150°C-5d	26653

Table S19. Counts of the individual signals of different type of $[Pt_n(\mathbf{L^{OMe}})_{2n}]^{2n+}$ assemblies over time.

Sphere type M_nL_{2n}	110°C 1d	110°C 2d	110°C 3d	110°C 4d	110°C 5d	150°C 1d	150°C 2d	150°C 3d	150°C 4d
5	0	0	0	0	0	0	0	0	0
6	10153	9563	8791	11943	14927	15186	12050	9683	3903
7	482	0	0	0	0	0	0	0	0
8	12502	19873	21257	26109	32709	40954	39905	41377	26230
9	12726	22501	24639	29876	38570	49144	51826	55628	38459
10	6906	8687	8446	9144	9992	7213	4268	3265	0
11	5874	9568	9814	12337	15534	16672	14822	14160	7757
12	7403	13596	15462	19611	24454	38605	49367	56446	46554

Error considerations of mass quantification

In order to estimate an error which can be expected for the individual assemblies, three different sphere solutions were prepared by heating two different batches of $\mathbf{L^{OMe}}$ with two different batches of $[Pt(BF_4)_2(MeCN)_4]$ at 110°C for 1d. The relative intensity of the individual assemblies varied in the obtained spectra ±3% which corresponds to an error of 10% for the individual assemblies. Whereas this error is rather big in the context of quantification of the individual assemblies, the obtained data was qualitatively three times reproduced. A similar trend is also the case if a different set of signals is used for the quantification of the assemblies. As our setting allowed well resolved peaks in the region between 900-1200 Da, we focused on individual signals in this region. Instead also other signals can be used for the quantification leading to similar results as depicted in S64.

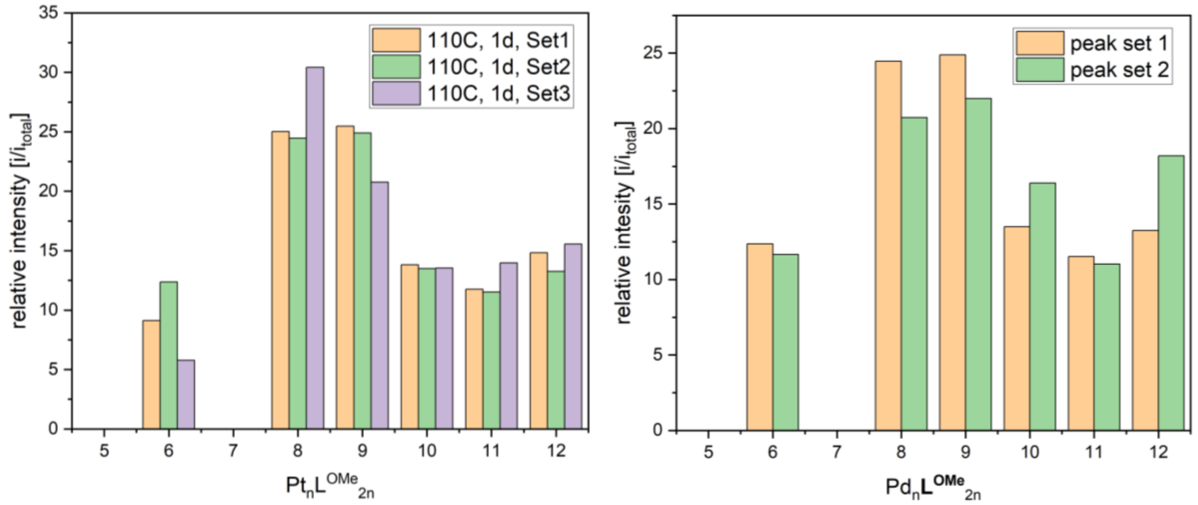

Figure S70. Left: relative intensity for different sized assemblies in three unique performed experiments. Right: Distribution of different sized assemblies depending on the individual peaks used for their quantification.

The machine intensity error was estimated by performing 10 individual measurements on the same sample and comparison of the obtained intensities.

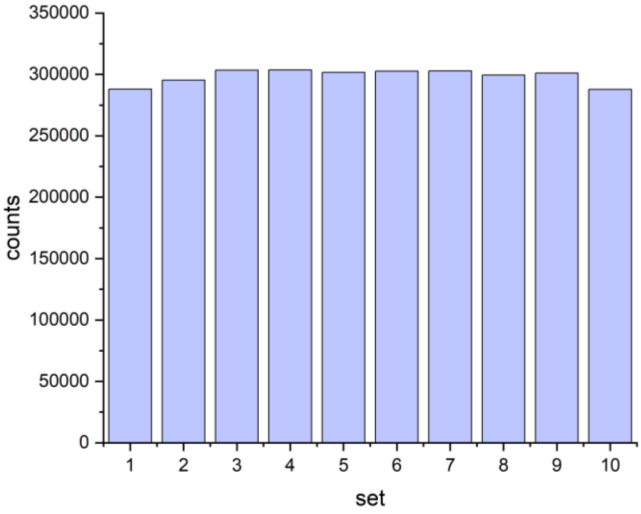

Figure S71. Measured intensity for a certain peak of a self-assembly. After each measurement, the machine was cleaned and flushed with acetonitrile before the next set was measured.

This set of measurement suggest a machine error of 3%, which is below the individual error obtained from different set of experiments (which is 10% as suggested before).

$[Pt_n(\mathbf{L^{OMe}})_{2n}]^{2n+}(BF_4^-)_{2n}$ with addition of 2-chloropyridine

A samples containing a solution of $\mathbf{L^{OMe}}$ (3.1 mg, 10 µmol, 1 eq.) and 2-chloropyridine (1.1 mg, 1 µmol, 0.1 eq.) in 0.5 mL MeCN-d_3 and $[Pt(BF_4)_2(MeCN)_4]$ (2.93 mg, 5.5 µmol. 0.55 eq.) in 0.5 mL MeCN-d_3 was prepared. The solution was then stirred at 150°C for 24h in a 10 mL high pressure tube. The MS spectra of the resulting solution was compared to a sample not containing 2-chloropyridine. Both samples contained a similar distribution of spheres.

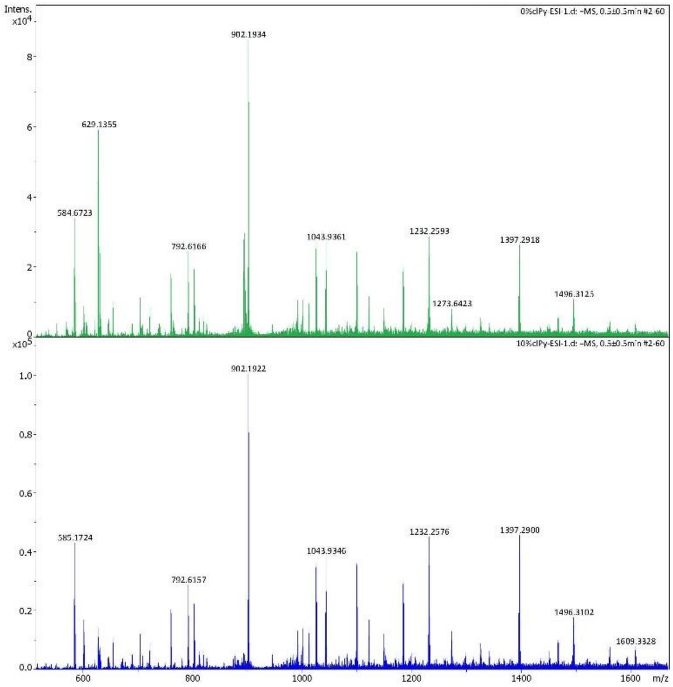

Figure S72. Comparison of two samples of $[Pt_n(\mathbf{L}^{\mathbf{OMe}})_{2n}]^{2n+}(BF_4^-)_{2n}$ containing either 10% 2-chloropyridine as destabilizing agent (bottom) or no destabilizing agent (top).

<u>$[Pt_n(\mathbf{L}^{\mathbf{OMe}})_{2n}]^{2n+}(BF_4^-)_{2n}$ decomposition at elevated temperatures</u>

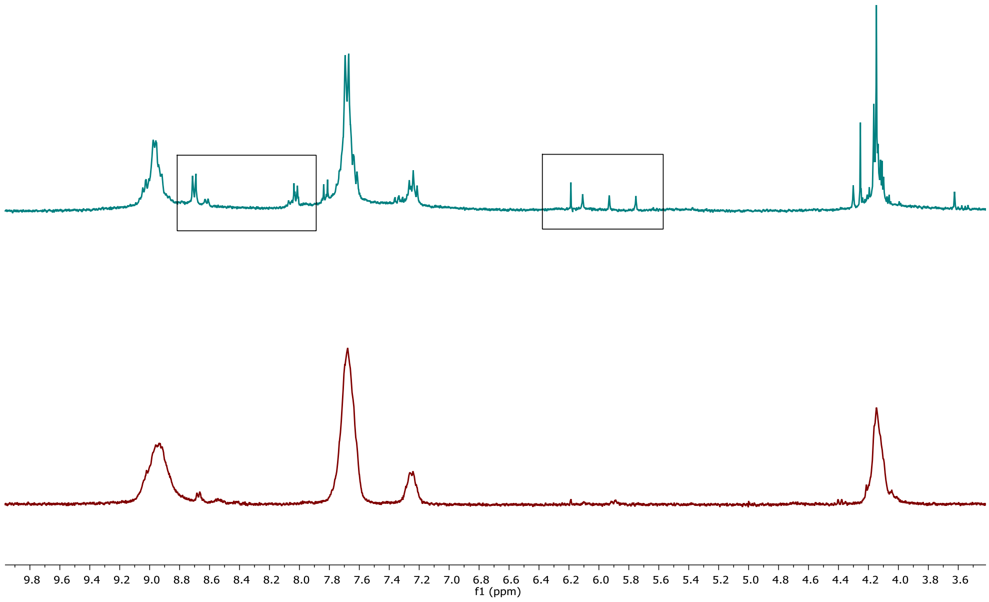

Figure S73. Comparison of free $[Pt_n(\mathbf{L}^{\mathbf{OMe}})_{2n}]^{4n+}(BF_4^-)_{4n}$ sphere prepared at 110°C for 3d (botom) and $[Pt_n(\mathbf{L}^{\mathbf{OMe}})_{2n}]^{4n+}(BF_4^-)_{4n}$ sphere prepared at 170°C for 3d (top), ^1H NMR in MeCN-d_3. Squares mark free building block and organic decomposition products.

$[Pt_n(\mathbf{L^{OMe}})_{2n}]^{2n+}(BF_4^-)_{2n}$ prepared at 70°C

A samples containing a solution of $\mathbf{L^{OMe}}$ (3.1 mg, 10 µmol, 1 eq.) in 0.5 mL MeCN-d_3 and $[Pt(BF_4)_2(MeCN)_4]$ (2.93 mg, 5.5 µmol. 0.55 eq.) in 0.5 mL MeCN-d_3 was prepared. The solution was then stirred at 70°C for 3d in a 10 mL high pressure tube. ^1H NMR (300 MHz, Acetonitrile-d_3) δ 9.20 – 8.53 (m, 5H), 7.88 – 7.46 (m, 6H), 7.26 (s, 1H), 4.32 – 3.87 (m, 3H).

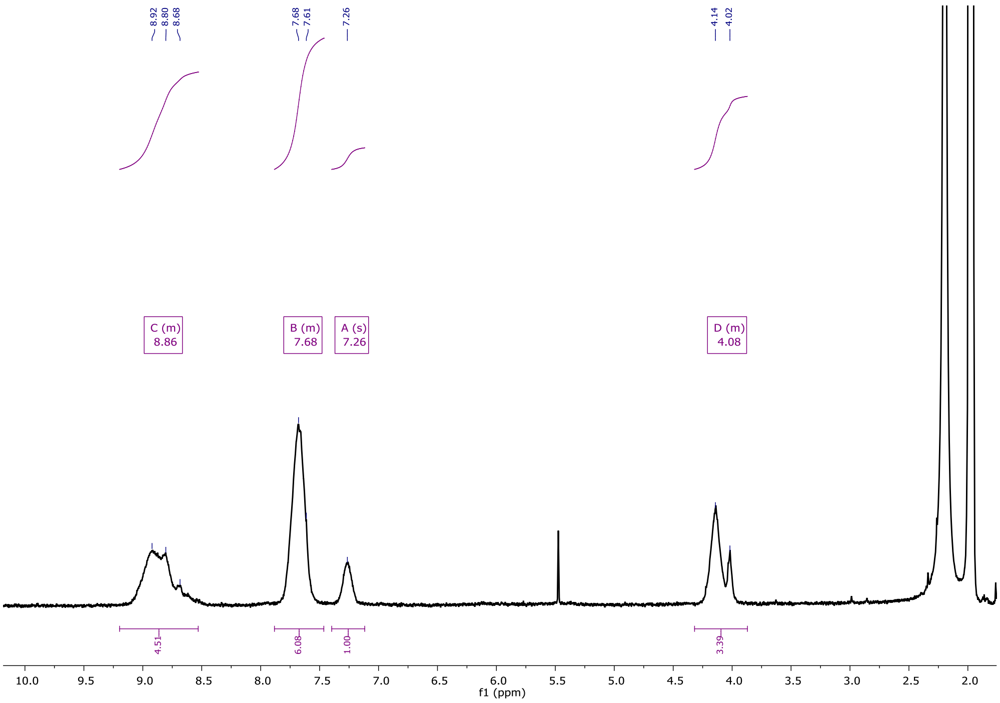

Figure S74. $\mathbf{L^{OMe}}$ + "Pt" precursor heated at 70°C for 3d, ^1H NMR in MeCN-d_3.

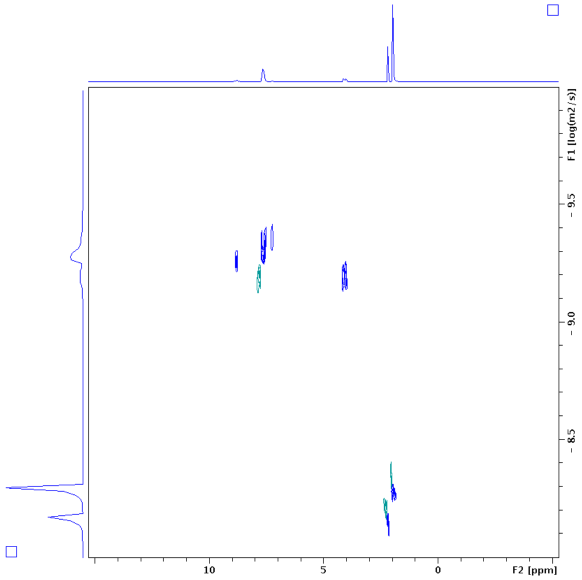

Figure S75. "Pt" + $\mathbf{L^{OMe}}$ complex prepared at 70°C, DOSY in MeCN-d_3 at 25°C.

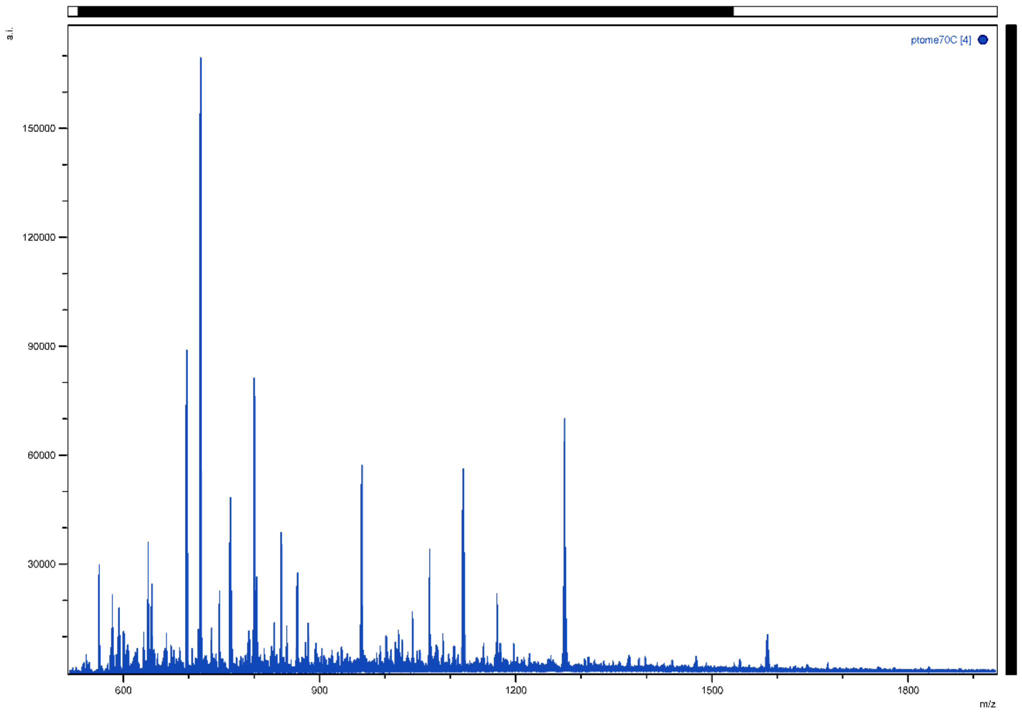

Figure S76. MS spectra of a sample of “Pt” + $\mathbf{L^{OMe}}$ mixed and heated at 70°C for 3d, showing absence of characteristic sphere signals.

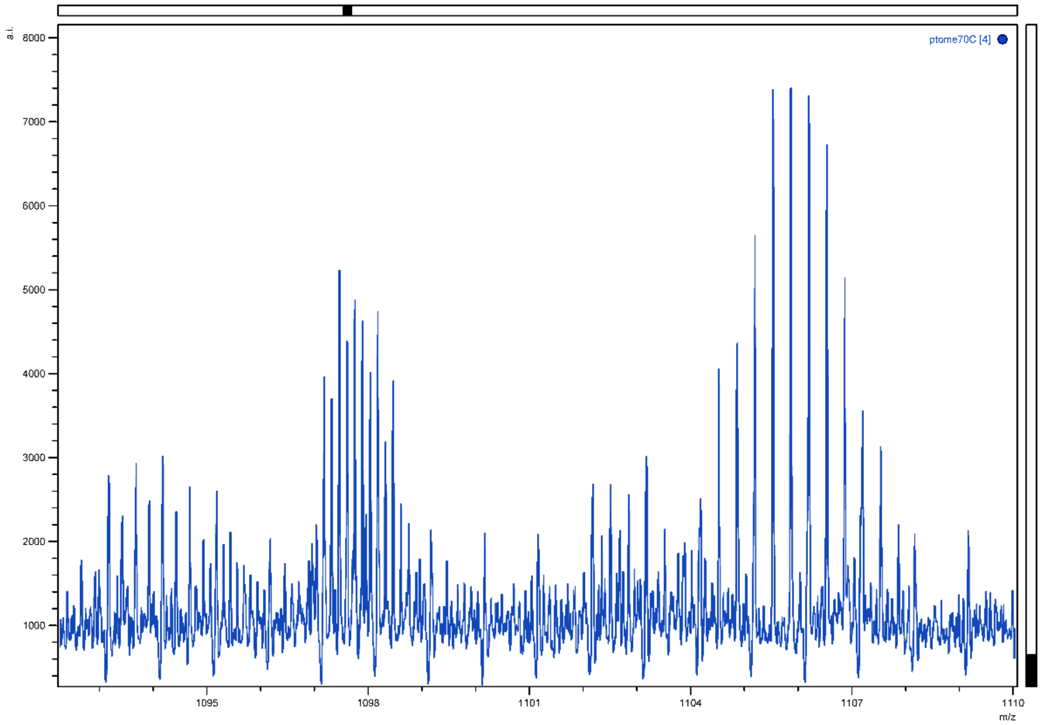

Figure S77. Zoom in into the 1100 region of the MS spectra of a sample of “Pt” + $\mathbf{L^{OMe}}$ mixed and heated at 70°C for 3d, showing absence of the characteristic 1100 signal otherwise obtained for $[Pt_6(\mathbf{L^{OMe}})_{12}]^{5+}$.

[Pt$_n$(**L^{Imi}**)$_{2n}$]$^{2n+}$(BF$_4^-$)$_{2n}$ prepared at 85°C

To a solution of **L^{Imi}** (5.20 mg, 10 µmol, 1 eq.) in 0.5 mL MeCN-d$_3$, [Pt(BF$_4$)$_2$(MeCN)$_4$] (2.93 mg, 5.5 µmol. 0.55 eq.) in 0.5 mL MeCN-d$_3$ was added. The solution was then stirred at 85°C for 1d in a 10 mL high pressure tube.

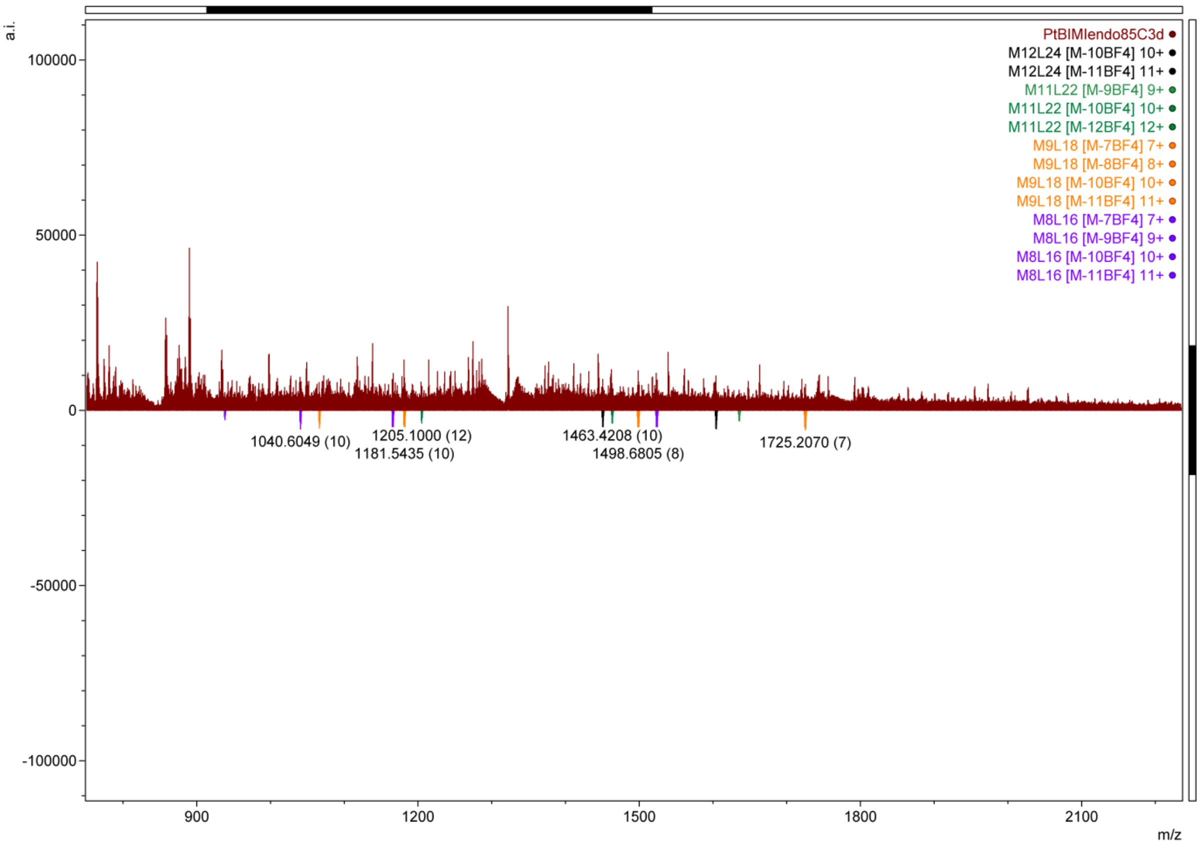

Figure S78. Full ESI-MS spectra of [Pt$_n$(**L^{Imi}**)$_{2n}$]$^{2n+}$(BF$_4^-$)$_{2n}$ prepared at 85°C for 1d. Below the simulated spectra, above obtained spectra.

Table S20. Zoom into different charged species of $[Pt_{12}(\mathbf{L}^{\mathbf{Imi}})_{24}]^{24+}$ assembly of a sample prepared at 85°C for 1d.

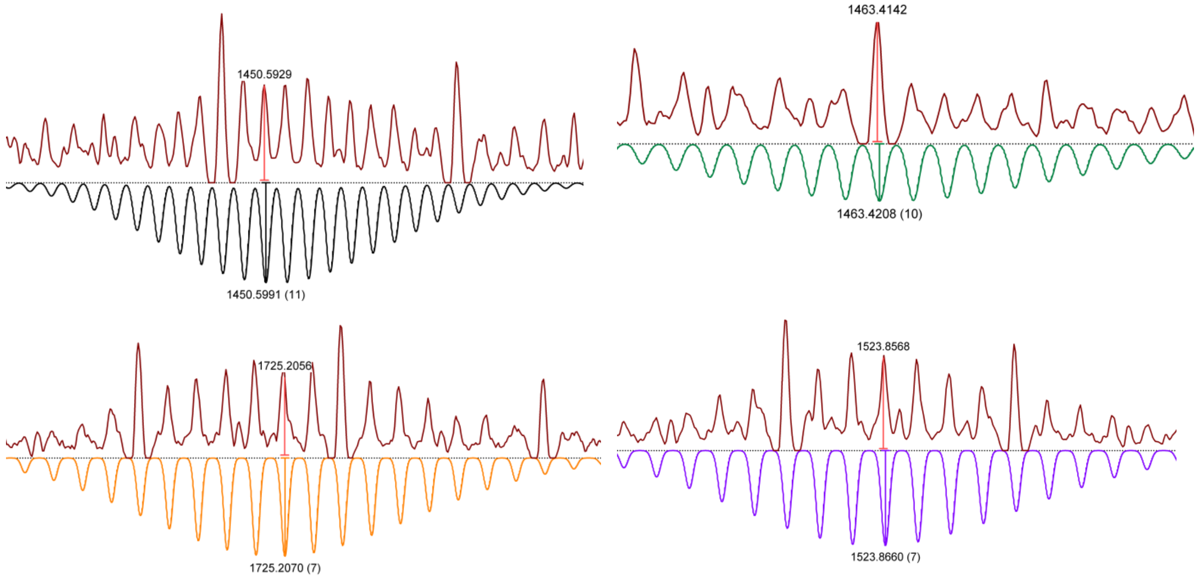

Ligand exchange studies (SI4)

$[Pt_n(\mathbf{L}^{\mathbf{OMe}})_{2n}]^{2n+}(BF_4^-)_{2n}$ + $[Pt_n(\mathbf{L}^{\mathbf{OBn}})_{2n}]^{2n+}(BF_4^-)_{2n}$

Different samples containing a solution of $\mathbf{L}^{\mathbf{OMe}}$ (3.1 mg, 10 µmol, 1 eq.) in 0.5 mL MeCN-d_3 and $[Pt(BF_4)_2(MeCN)_4]$ (2.93 mg, 5.5 µmol. 0.55 eq.) in 0.5 mL MeCN-d_3 or $\mathbf{L}^{\mathbf{OBn}}$ (3.86 mg, 10 µmol, 1 eq.) in 0.5 mL MeCN-d_3 and $[Pt(BF_4)_2(MeCN)_4]$ (2.93 mg, 5.5 µmol. 0.55 eq.) in 0.5 mL MeCN-d_3 were prepared. The solution was then stirred at either room temperature, 85°C or 110°C for 24h. The resulting solutions were analyzed before being mixed in a 1 to 1 ratio and heated to 150°C for 24h. After that all individual solutions were analyzed again with ^1H NMR, DOSY and MS.

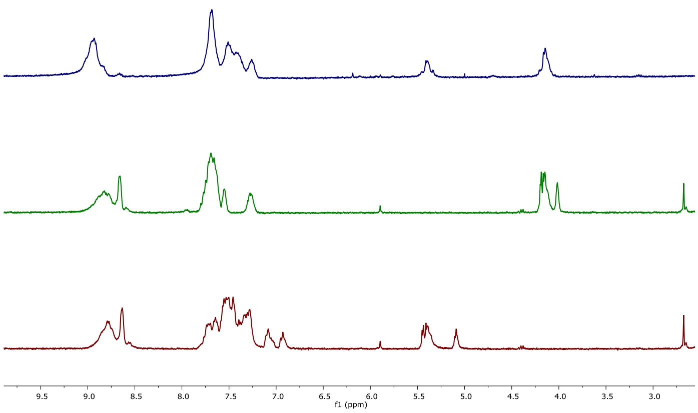

Figure S79. Stacked spectra of $[Pt_n(\mathbf{L^{OBn}})_{2n}]^{2n+}(BF_4^-)_{2n}$ (bottom) prepared at 25°C, $[Pt_n(\mathbf{L^{OMe}})_{2n}]^{2n+}(BF_4^-)_{2n}$ (middle) prepared at 25°C and 1 to 1 solution heated at 150°C for 24h (top), ^{1}H NMR in MeCN-d$_3$.

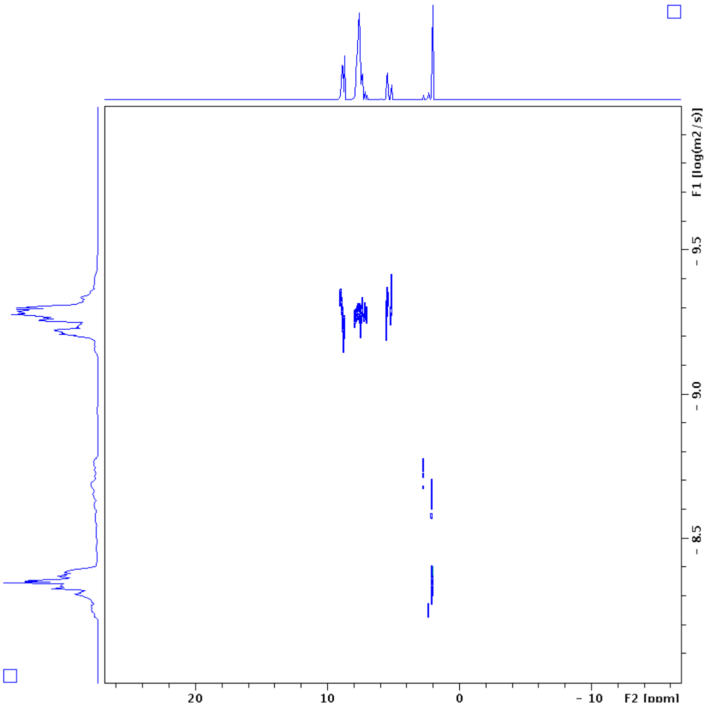

Figure S80. $[Pt_n(\mathbf{L^{OBn}})_{2n}]^{2n+}(BF4^-)_{2n}$ cage prepared at 25°C, DOSY in MeCN-d$_3$ at 25°C.

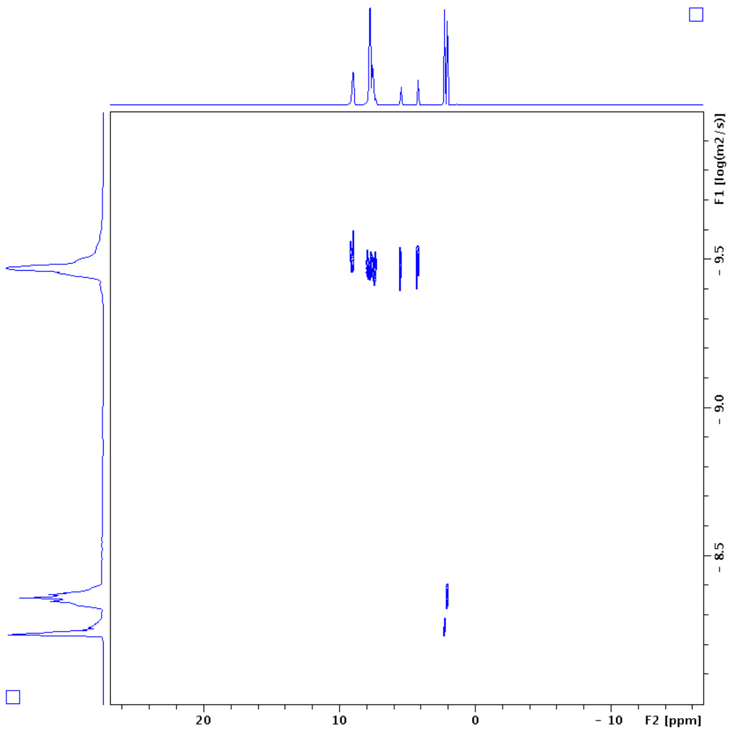

Figure S81. $[Pt_n(\mathbf{L}^{\mathbf{OBn}})_{2n}]^{2n+}(BF4^-)_{2n}$ + $[Pt_n(\mathbf{L}^{\mathbf{OMe}})_{2n}]^{2n+}(BF4^-)_{2n}$ cages (1:1) prepared at 25°C mixed and heated to 150°C for 24 h, DOSY in MeCN-d_3 at 25°C.

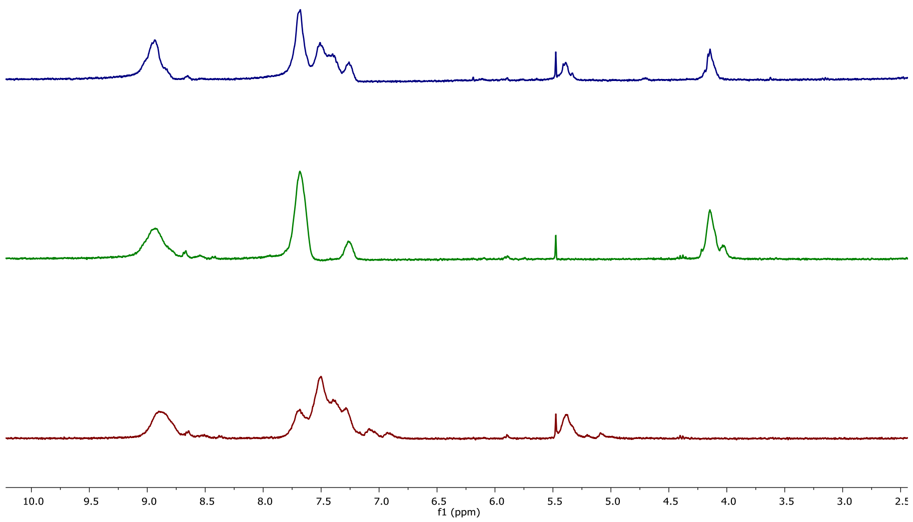

Figure S82. Stacked spectra of $[Pt_n(\mathbf{L}^{\mathbf{OBn}})_{2n}]^{2n+}(BF_4^-)_{2n}$ (bottom) prepared at 85°C, $[Pt_n(\mathbf{L}^{\mathbf{OMe}})_{2n}]^{2n+}(BF_4^-)_{2n}$ (middle) prepared at 85°C and 1 to 1 solution heated at 150°C for 24h (top), ^{1}H NMR in MeCN-d_3.

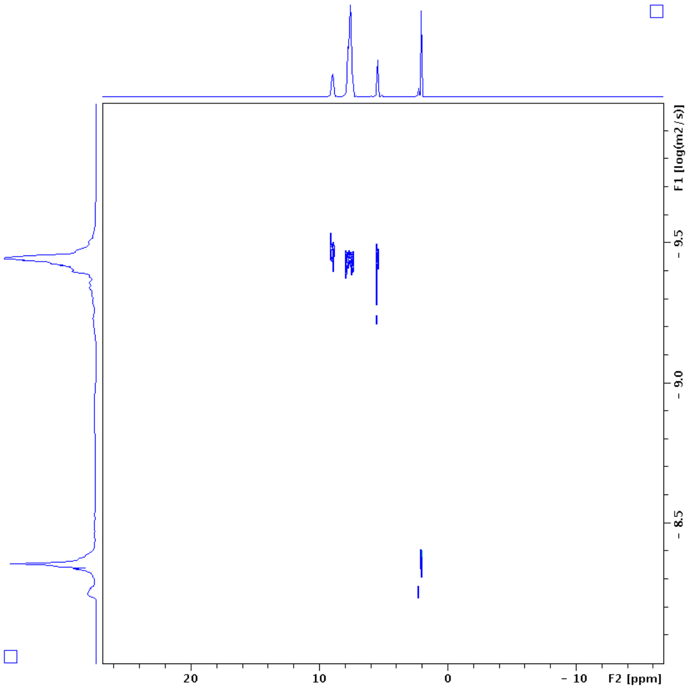

Figure S83. $[Pt_n(\mathbf{L^{OBn}})_{2n}]^{2n+}(BF4^-)_{2n}$ cage prepared at 85°C, DOSY in MeCN-d_3 at 25°C.

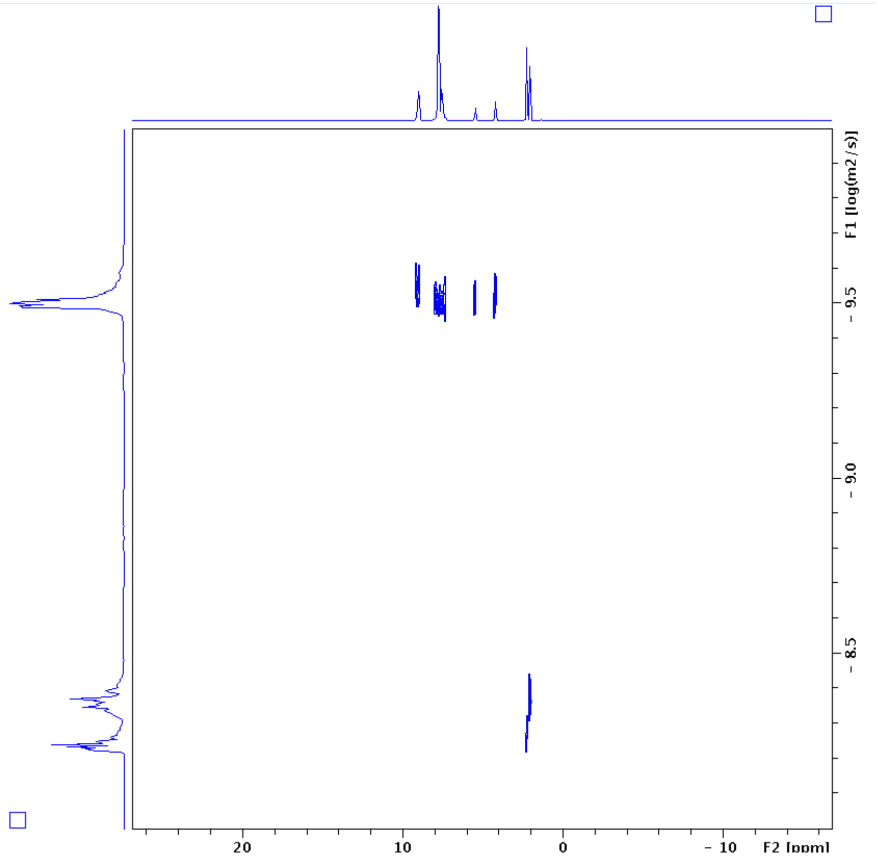

Figure S84. $[Pt_n(\mathbf{L^{OBn}})_{2n}]^{2n+}(BF4^-)_{2n}$ + $[Pt_n(\mathbf{L^{OMe}})_{2n}]^{2n+}(BF4^-)_{2n}$ cages (1:1) prepared at 85°C mixed and heated to 150°C for 24 h, DOSY in MeCN-d_3 at 25°C.

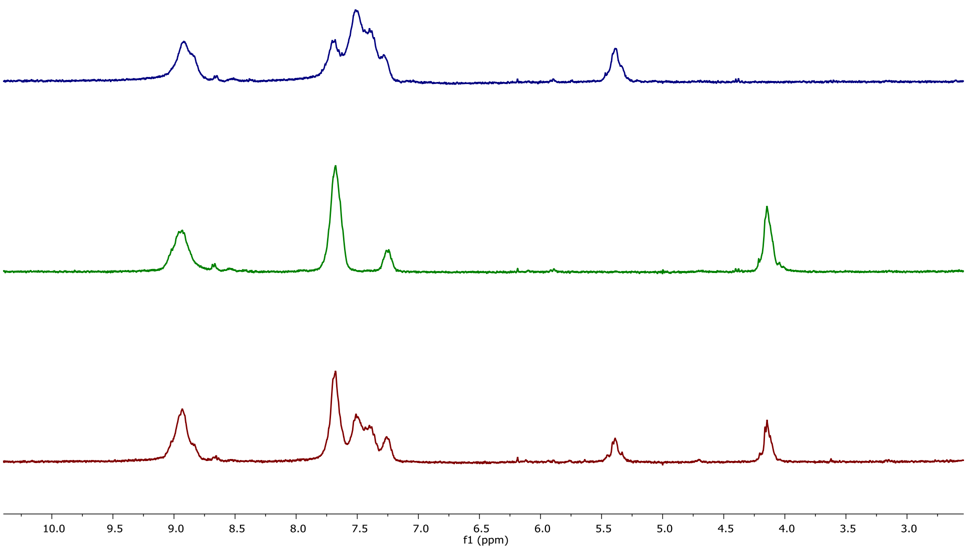

Figure S85. Stacked spectra of $[Pt_n(\mathbf{L^{OBn}})_{2n}]^{2n+}(BF_4^-)_{2n}$ (bottom) prepared at 110°C, $[Pt_n(\mathbf{L^{OMe}})_{2n}]^{2n+}(BF_4^-)_{2n}$ (middle) prepared at 110°C and 1 to 1 solution heated at 150°C for 24h (top), ^{1}H NMR in MeCN-d$_3$.

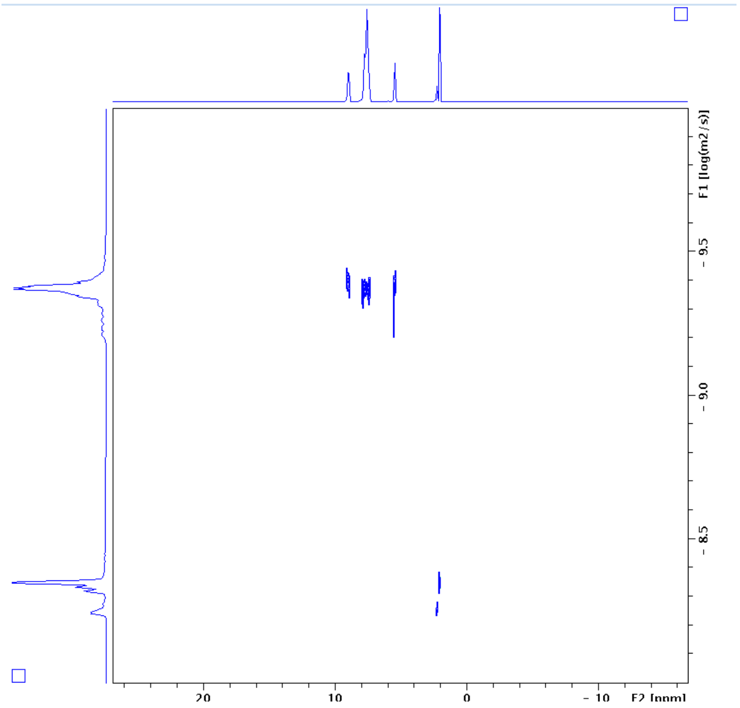

Figure S86. $[Pt_n(\mathbf{L^{OBn}})_{2n}]^{2n+}(BF4^-)_{2n}$ cage prepared at 110°C, DOSY in MeCN-d$_3$ at 25°C.

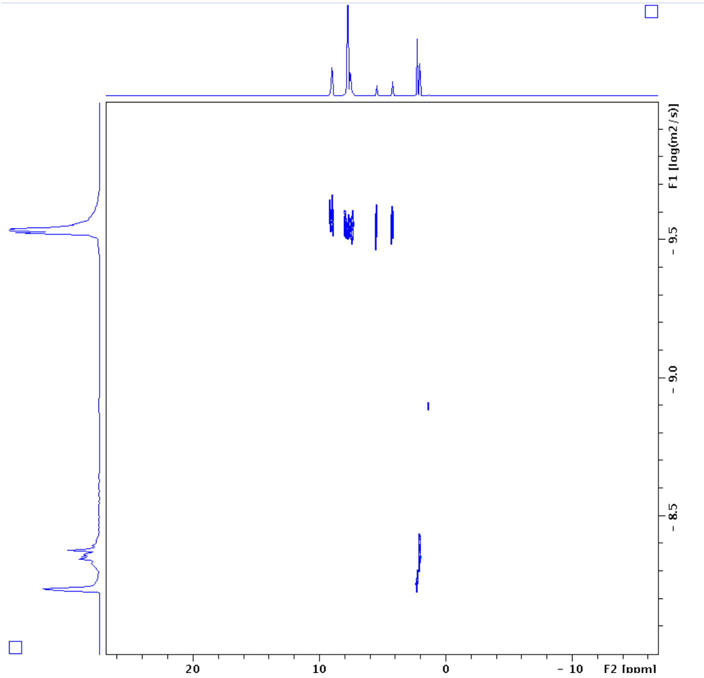

Figure S87. $[Pt_n(\mathbf{L}^{\mathbf{OBn}})_{2n}]^{2n+}(BF4^-)_{2n}$ + $[Pt_n(\mathbf{L}^{\mathbf{OMe}})_{2n}]^{2n+}(BF4^-)_{2n}$ cages (1:1) prepared at 110°C mixed and heated to 150°C for 24 h, DOSY in MeCN-d_3 at 25°C.

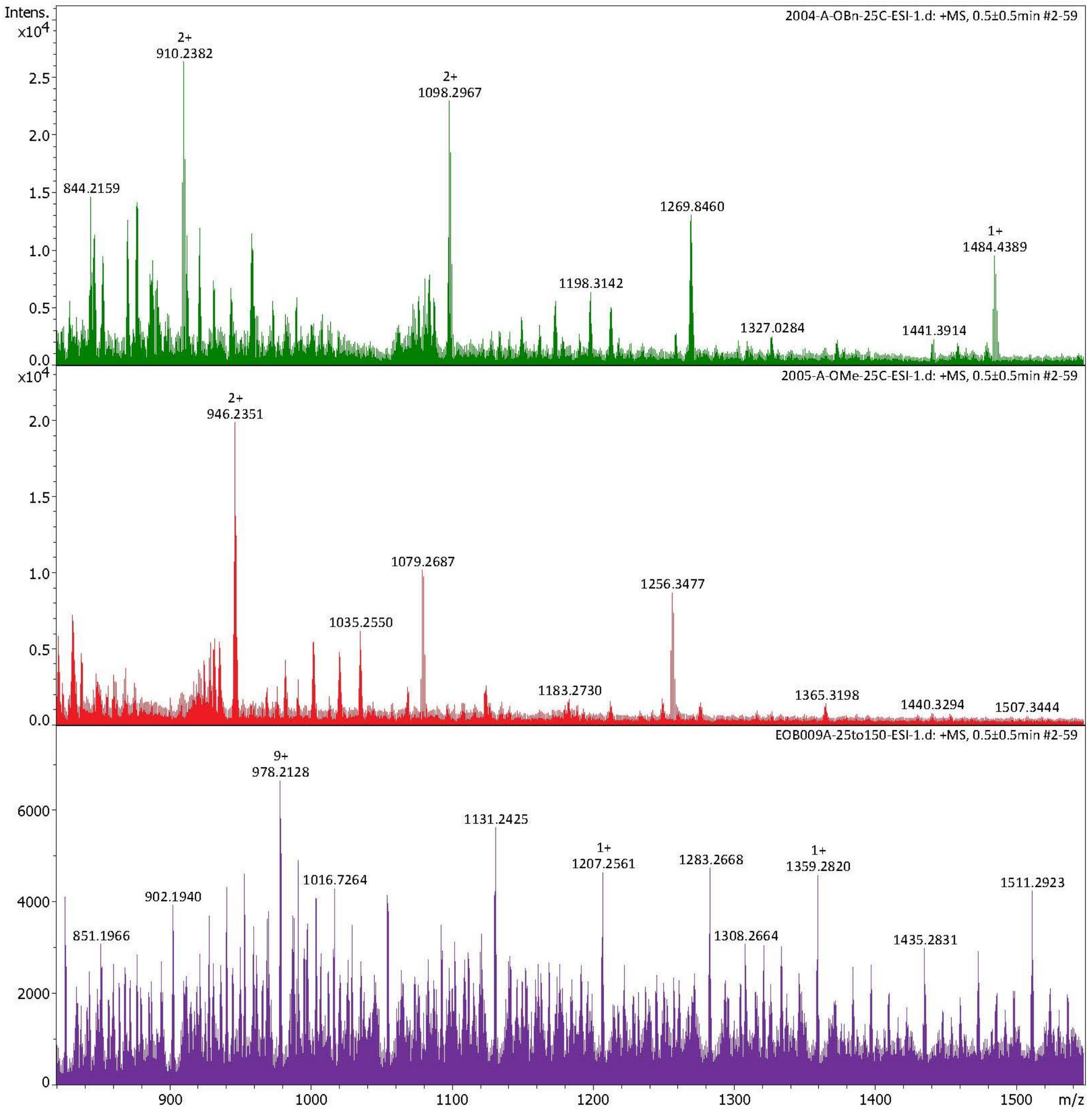

Figure S88. Example of collected MS spectra before and after heating two assemblies which were prepared at room temperature showing the emerge of spherical complexes with random distribution after heating the mixture at 150°C.

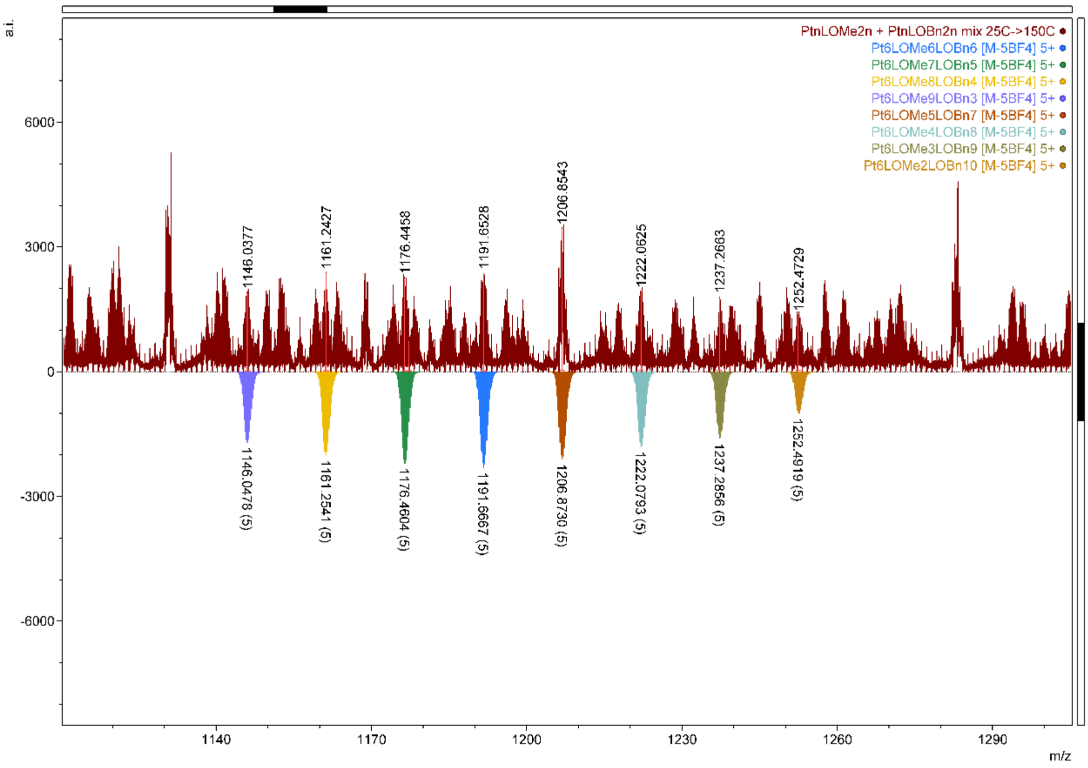

Figure S89. Distribution of the two ligands in M_6L_{12} assemblies (25°C to 150°C).

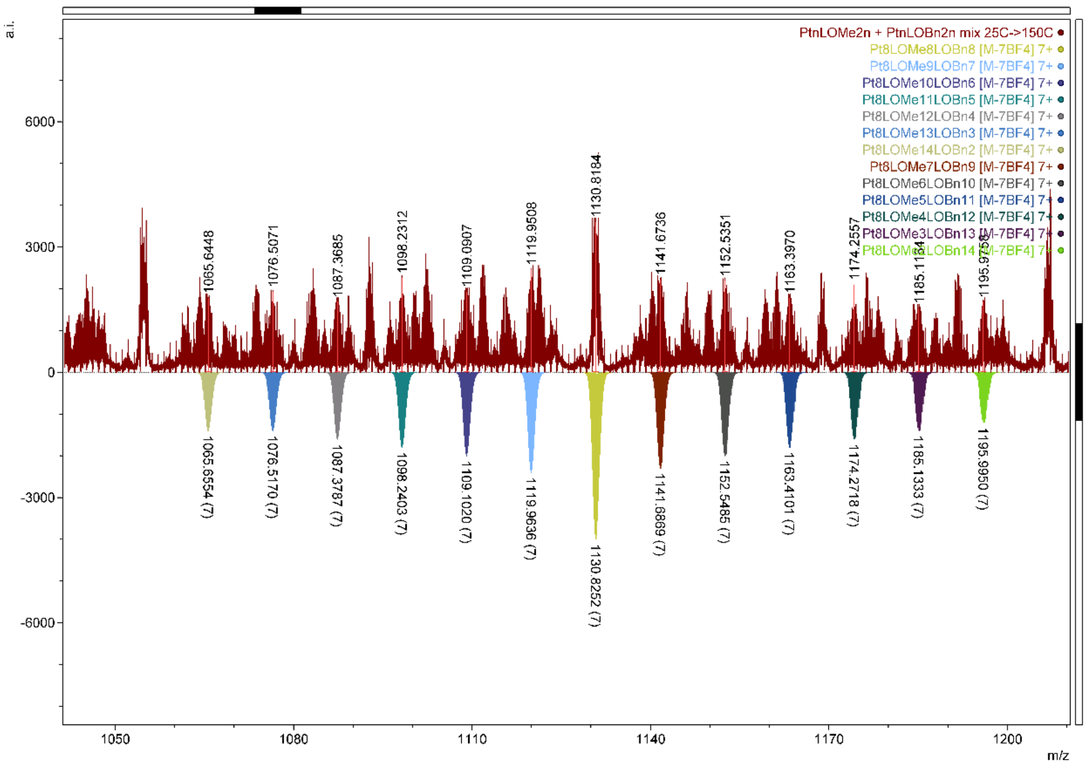

Figure S90. Distribution of the two ligands in M_8L_{16} assemblies (25°C to 150°C).

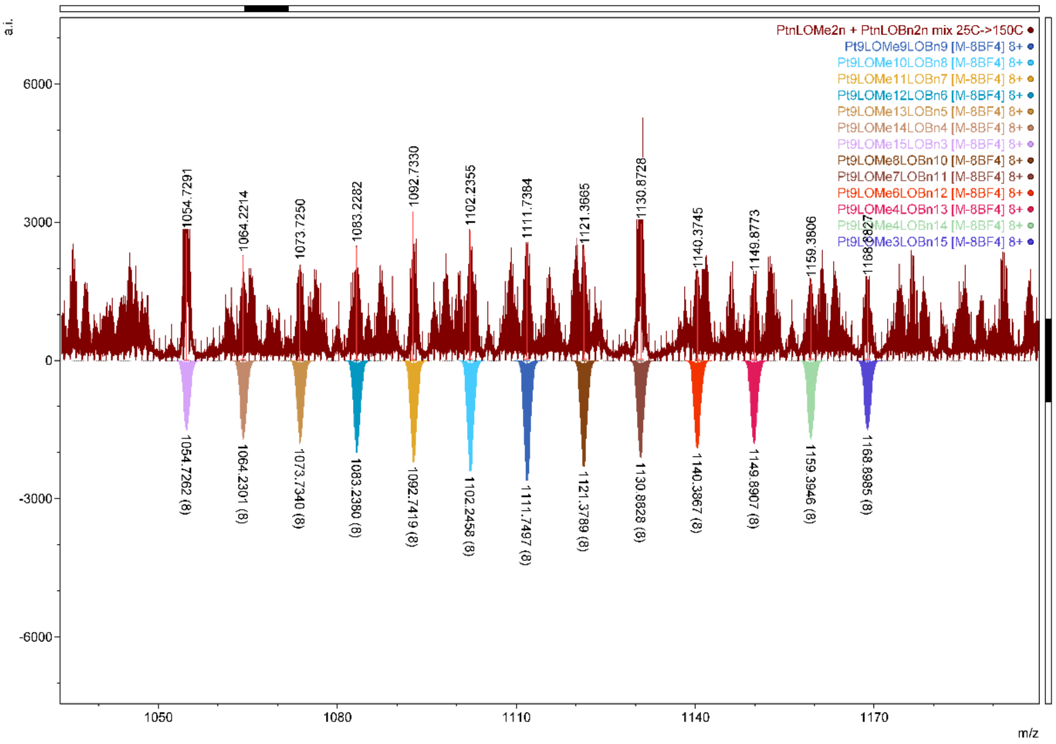

Figure S91. Distribution of the two ligands in M_9L_{18} assemblies (25°C to 150°C).

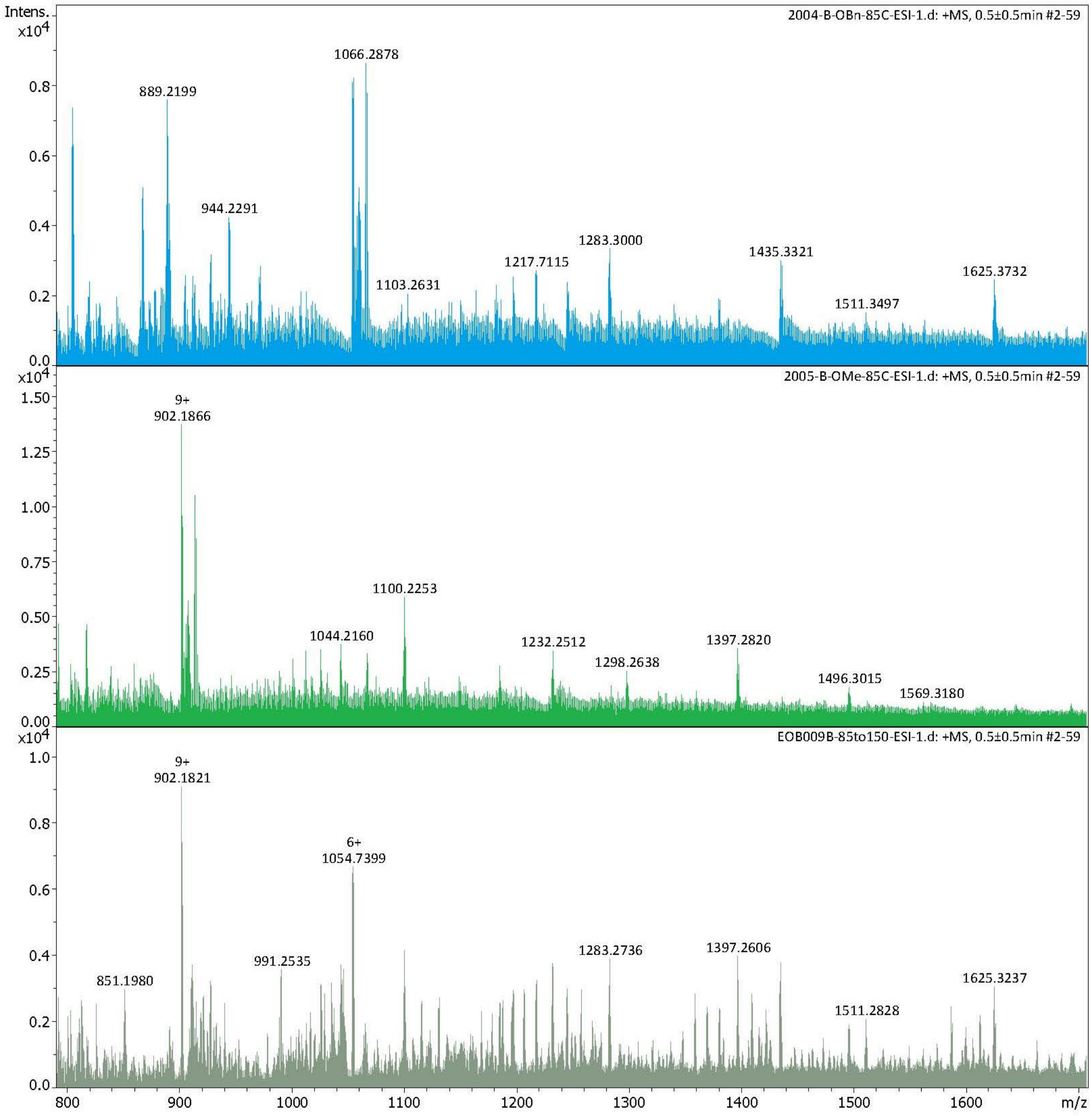

Figure S92. Example of collected MS spectra before and after heating two assemblies which were prepared at 85°C and then heated at 150°C.

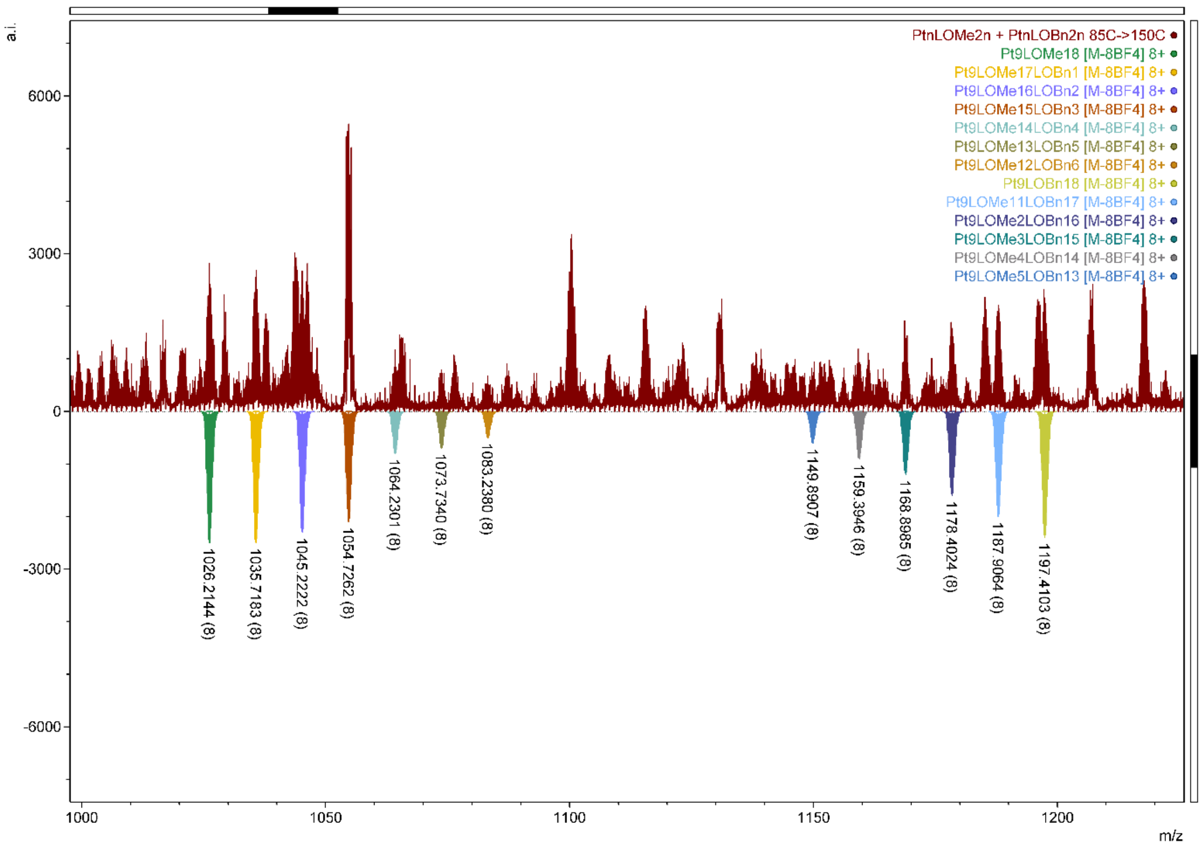

Figure S93. Distribution of the two ligands in M_9L_{18} assemblies (85°C to 150°C).

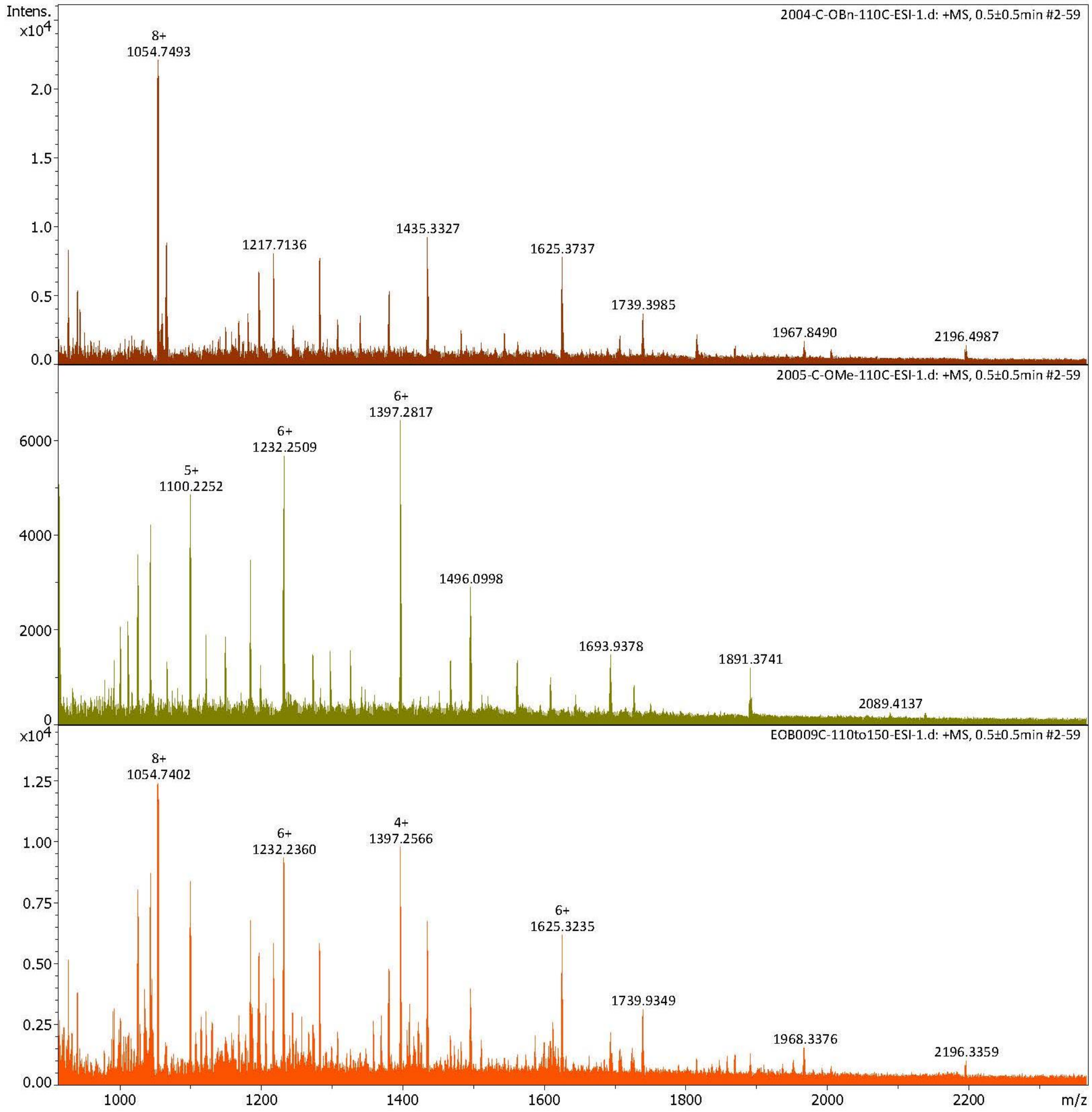

Figure S94. Example of collected MS spectra before and after heating two assemblies which were prepared at 110°C and then heated at 150°C.

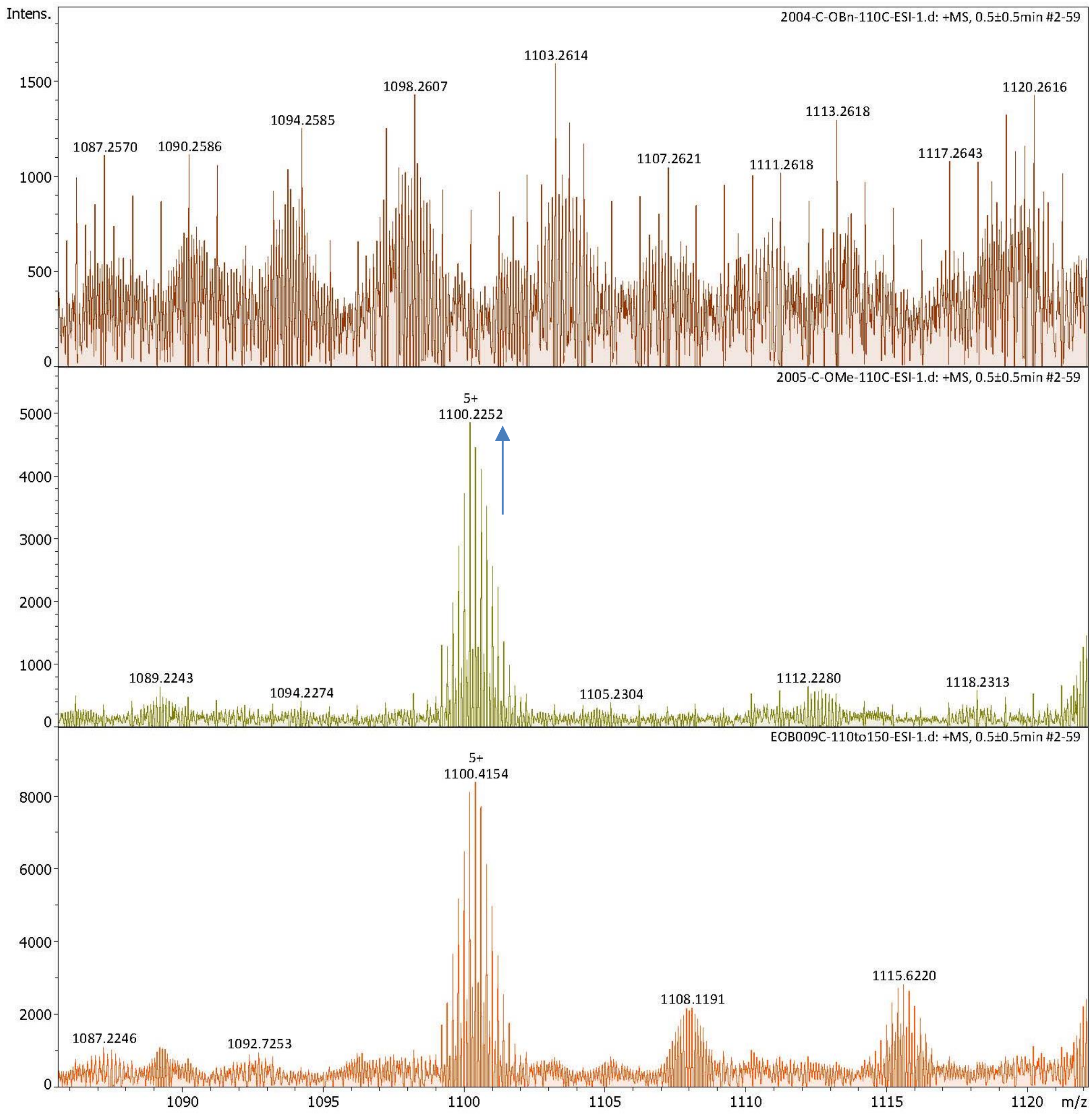

Figure S95. Example of collected MS spectra before and after heating two assemblies which were prepared at 110°C and then heated at 150°C. Zoom into 1100 region to show formation of more spherical objects after heating the mixture at 150°C.

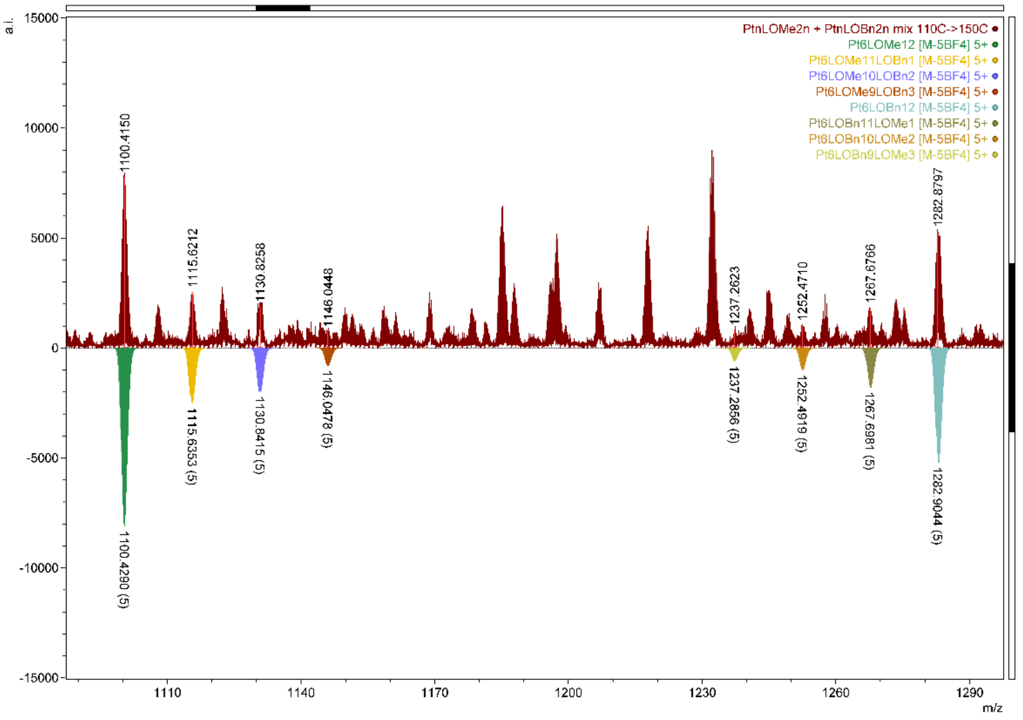

Figure S96. Distribution of the two ligands in M_6L_{12} assemblies (110°C to 150°C).

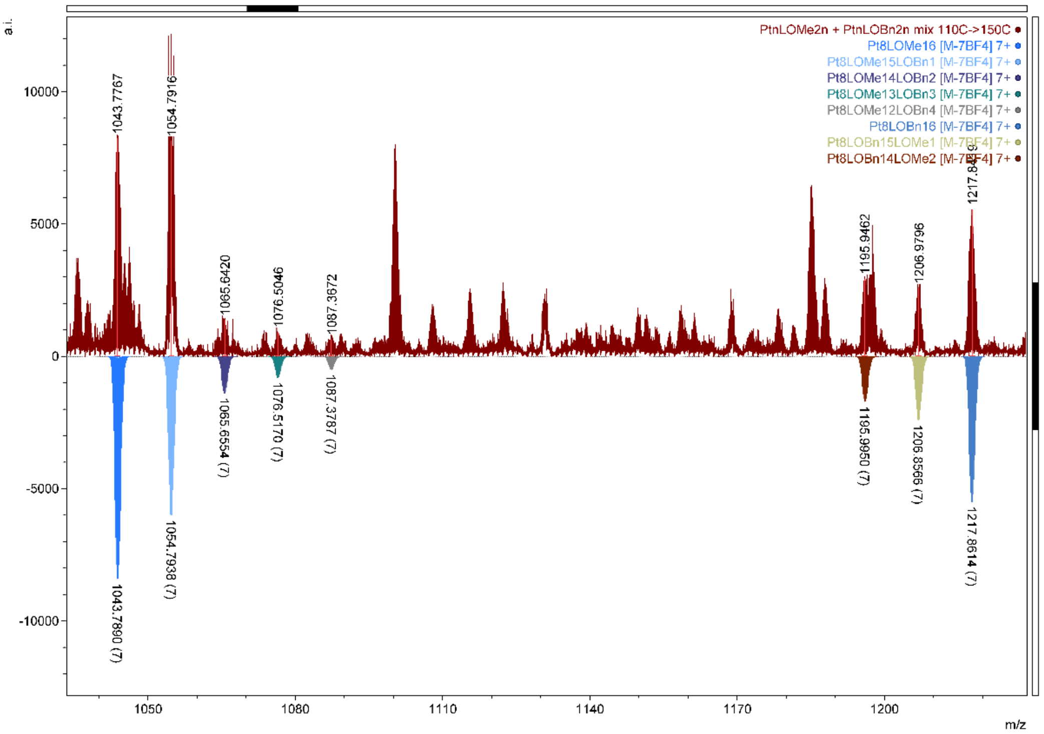

Figure S97. Distribution of the two ligands in M_8L_{16} assemblies (110°C to 150°C).

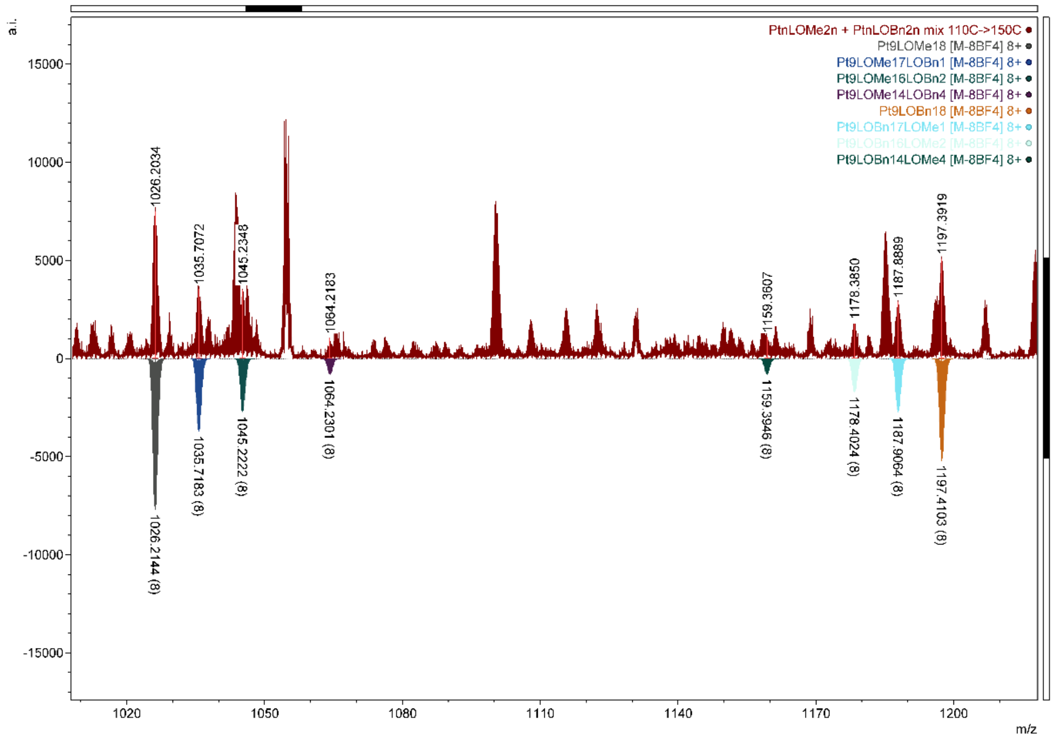

Figure S98. Distribution of the two ligands in M_9L_{18} assemblies (110°C to 150°C).

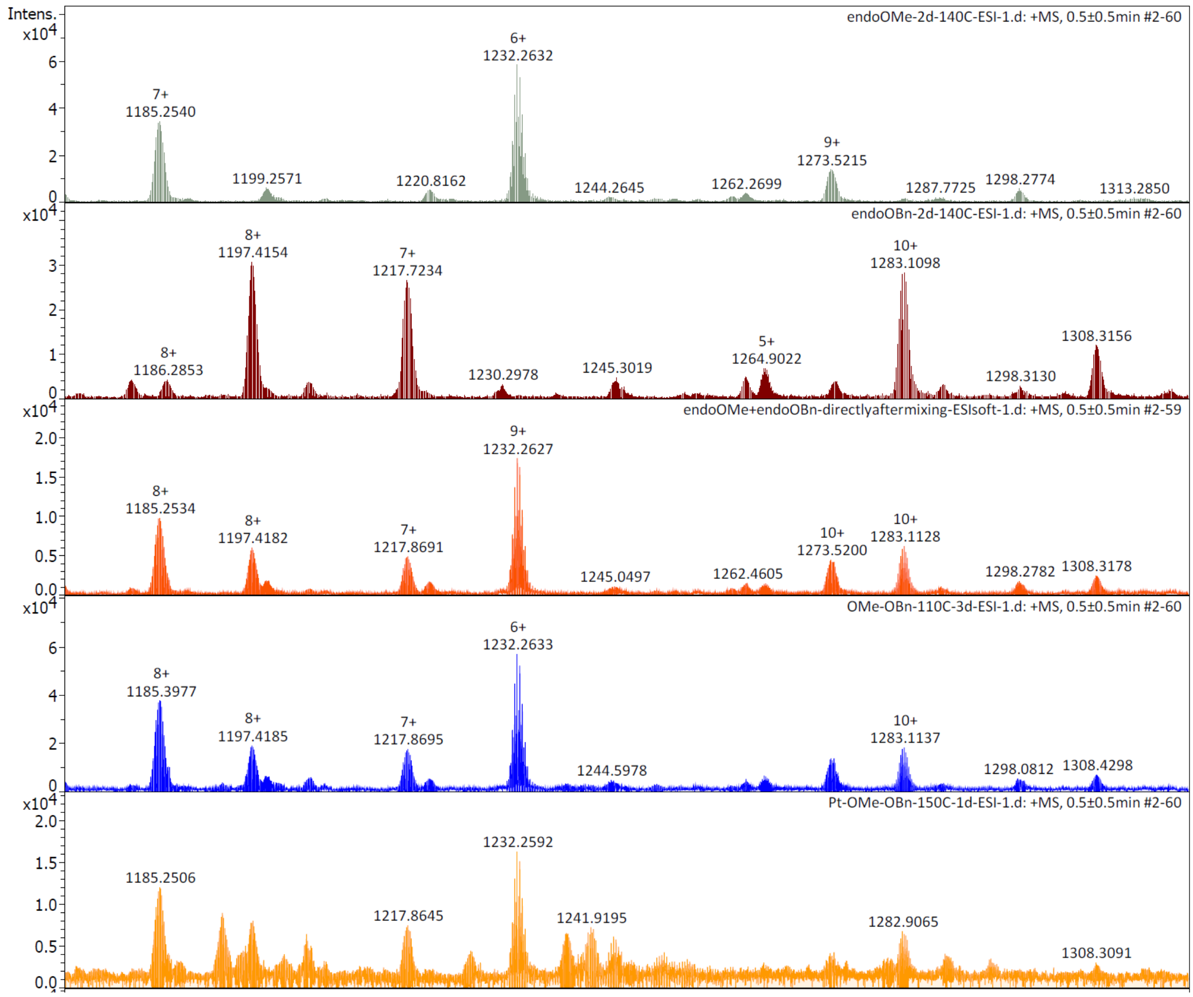

Figure S99. Example of collected MS spectra before and after heating two assemblies which were prepared at 110°C. The zoom in shows no change of the spectra after heating it at 110°C for 3d, only at 150°C the sample shows slow ligand exchange.

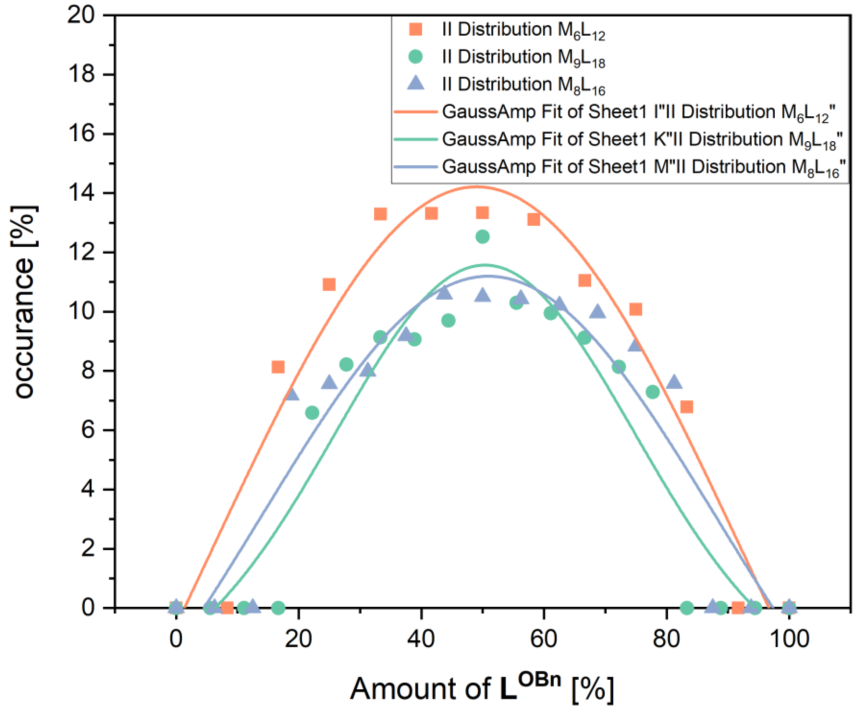

Figure S100. Ligand distribution after heating two assemblies which were prepared at 25°C to 150°C.

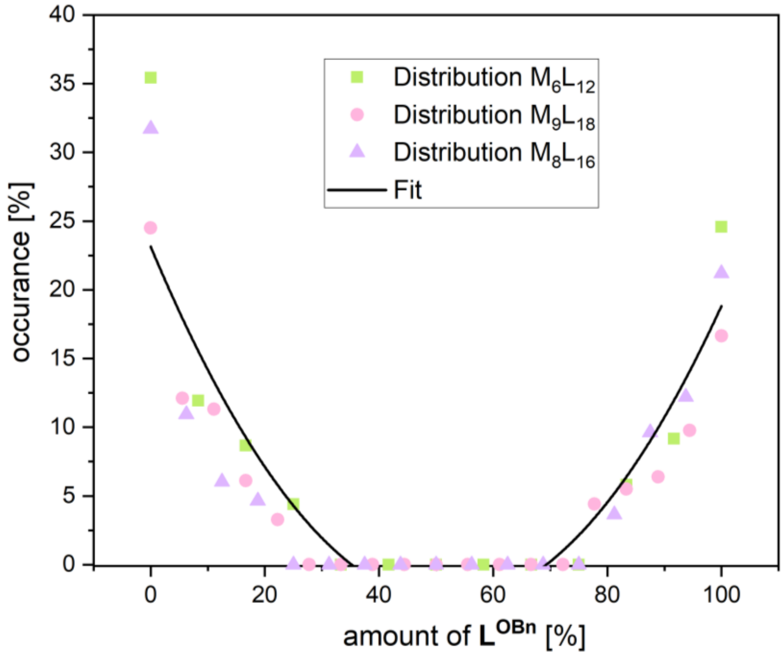

Figure S101. Ligand distribution after heating two assemblies which were prepared at 110°C to 150°C.

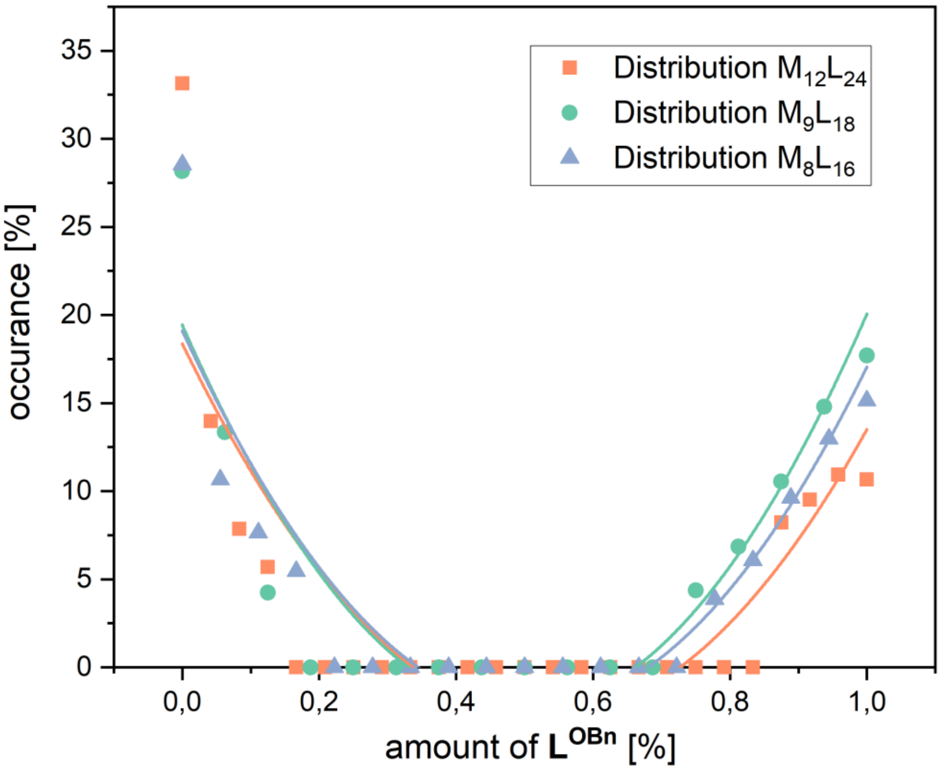

Figure S102. Ligand distribution after heating an assembly of $\mathbf{L^{OMe}}$ (prepared at 110°C) with polymeric material of $\mathbf{L^{OBn}}$ (prepared at 25°C) to 150°C.

$[Pt_n(\mathbf{L^{Py}})_{2n}]^{2n+}(BF_4^-)_{2n}$ + $[Pt_n(\mathbf{L^{ImiAd}})_{2n}]^{2n+}(BF_4^-)_{2n}$

Samples containing a solution of **L^Py^** (5.18 mg, 10 µmol, 1 eq.) in 0.5 mL MeCN-d_3 and $[Pt(BF_4)_2(MeCN)_4]$ (2.93 mg, 5.5 µmol. 0.55 eq.) in 0.5 mL MeCN-d_3 or $\mathbf{L^{ImiAd}}$ (6.40 mg, 10 µmol, 1 eq.) in 0.5 mL MeCN-d_3 and $[Pt(BF_4)_2(MeCN)_4]$ (2.93 mg, 5.5 µmol. 0.55 eq.) in 0.5 mL MeCN-d_3 were prepared. The solutions were then stirred at 110°C for 24h. The resulting solutions were mixed in a 1 to 1 ratio and heated to 150°C for 24h. After that the resulting solution was analyzed with MS.

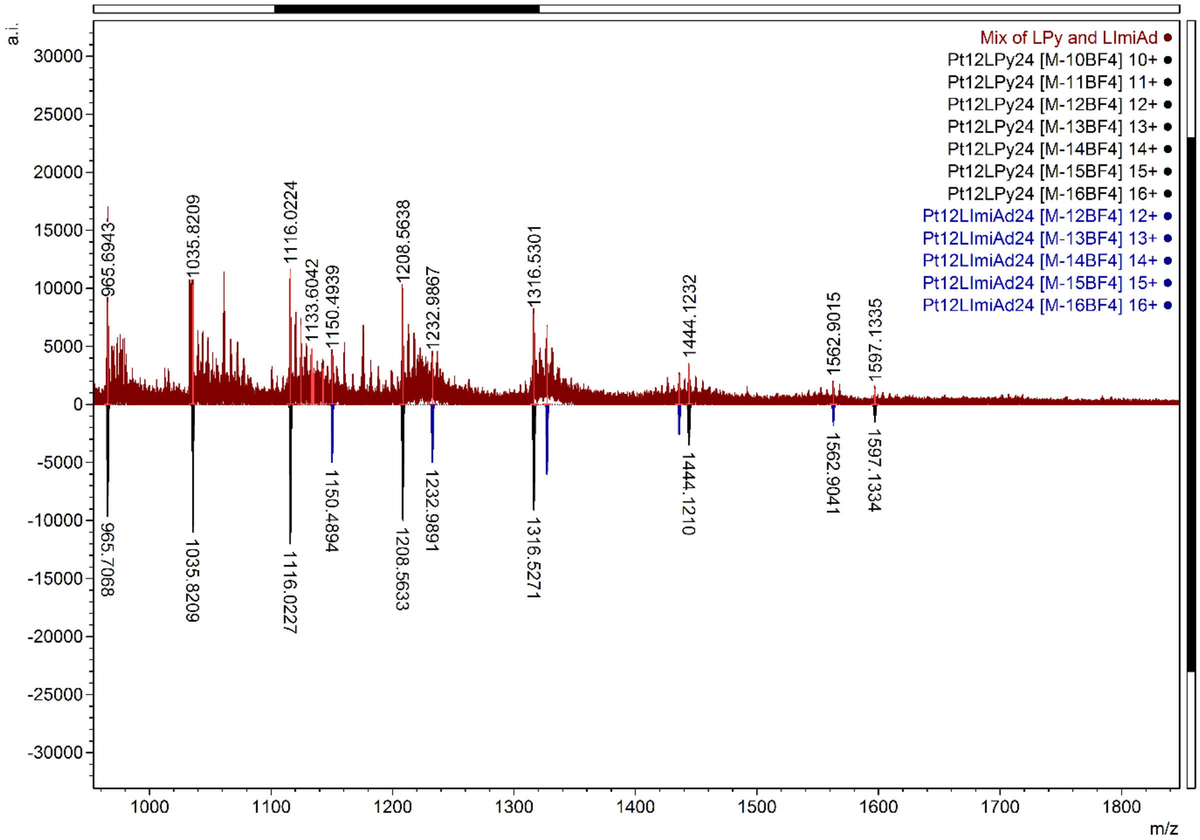

Figure S103. Full ESI-MS spectra of a mixture of $[Pt_{12}(\mathbf{L^{Py}})_{24}]^{48+}(BF_4^-)_{48}$ and $[Pt_{12}(\mathbf{L^{ImiAd}})_{24}]^{48+}(BF_4^-)_{48}$ prepared at 110°C for 1d. Both spheres were mixed in a 1 to 1 ratio and heated at 150°C for 24 h. Below the simulated spectra, above obtained spectra. Mostly homoleptic spheres are obtained as depicted in this MS spectra.

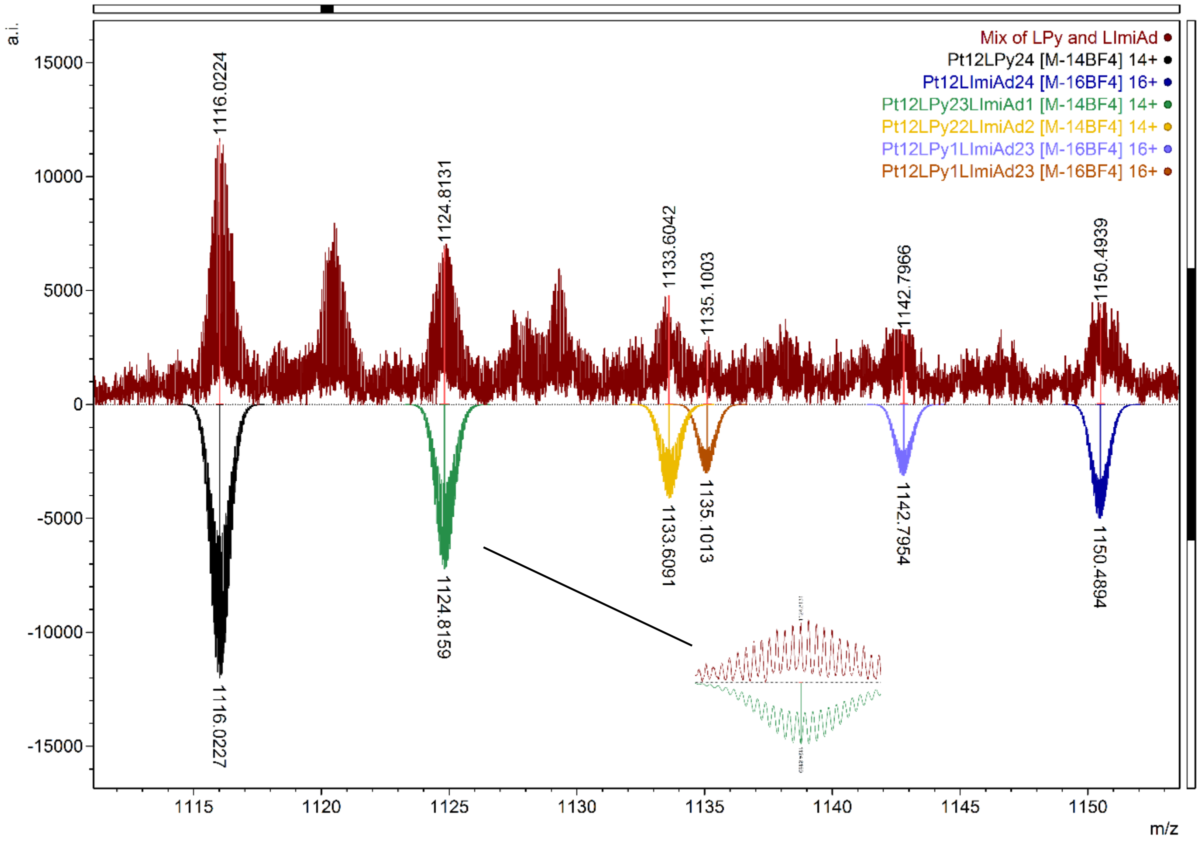

Figure S104. Zoom in into the 14+ species of Pt_{12}**L**$^{Py}{}_{24}$ assembly showing the distribution of **L**^{Py} and **L**^{ImiAd} in the obtained $Pt_{12}L_{24}$ assemblies.

Application for other structures (SI5)

The required ligands were synthesized according to literature (for **L^meta** [6] and for **L^para** [7]).

[Pt_2(**L^meta**)$_4$]$^{4+}$(BF_4^-)$_4$

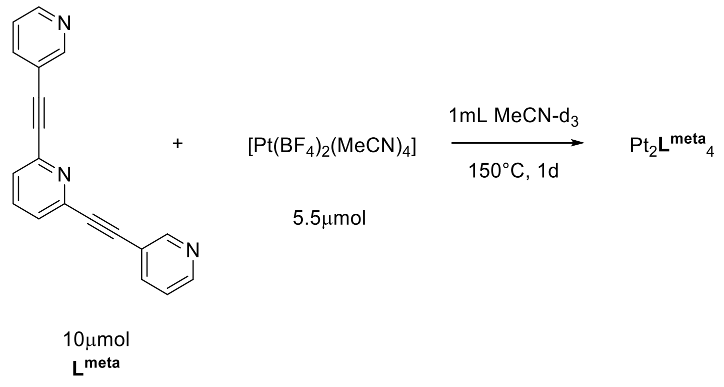

To a solution of **L^meta** (2.81 mg, 10 µmol, 1 eq.) in 0.5 mL MeCN-d3, [Pt(BF_4)$_2$(MeCN)$_4$] (2.93 mg, 5.5 µmol. 0.55 eq.) in 0.5 mL MeCN-d3 was added. The solution was then stirred at 150°C for 1d in a high pressure tube. ^{1}H NMR (300 MHz, Acetonitrile-d_3) δ 9.30 (d, J = 1.8 Hz, 4H), 9.06 (dt, J = 5.7, 1.2 Hz, 4H), 8.18 (dt, J = 8.1, 1.6 Hz, 6H), 7.90 (dd, J = 8.4, 7.3 Hz, 2H), 7.75 – 7.69 (m, 6H), 7.69 – 7.64 (m, 4H).

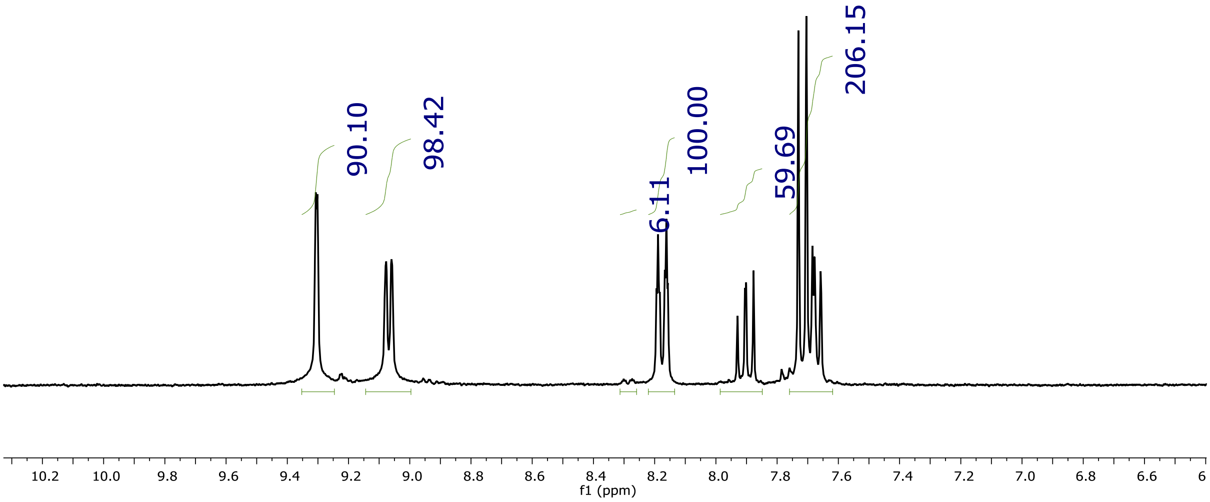

Figure S105. [Pt_2(**L^meta**)$_4$]$^{4+}$(BF_4^-)$_4$ sphere, ^{1}H NMR in MeCN-d3.

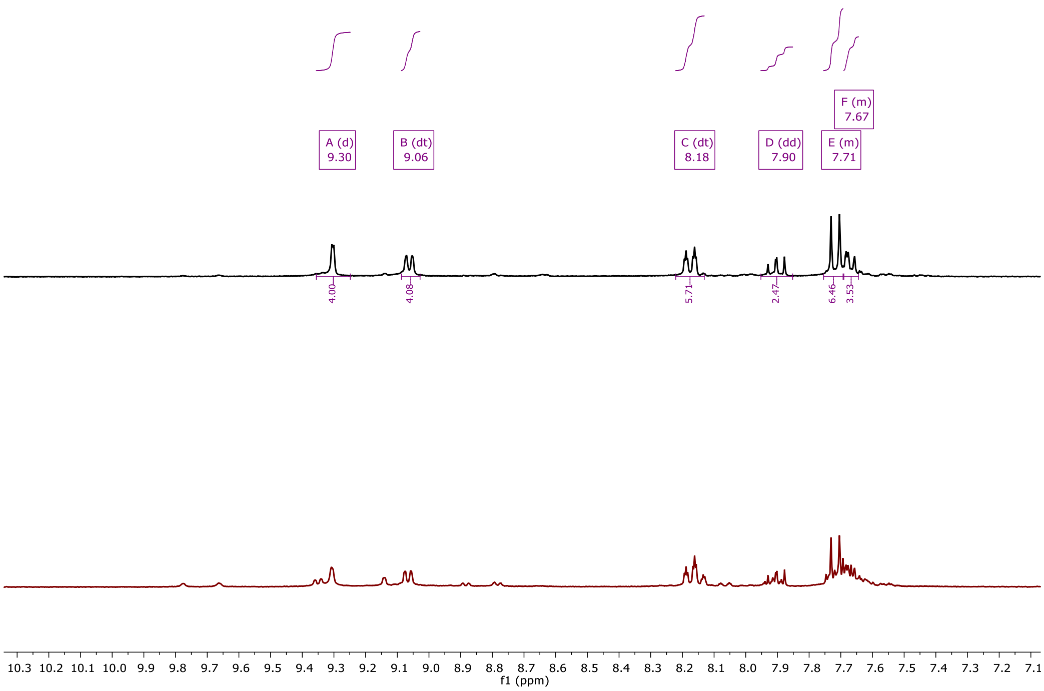

Figure S106. $[Pt_2(\mathbf{L^{meta}})_4]^{4+}(BF_4^-)_4$ sphere, ^1H NMR in MeCN-d_3, comparison of a sample prepared at 110°C (bottom) and 150°C (top).

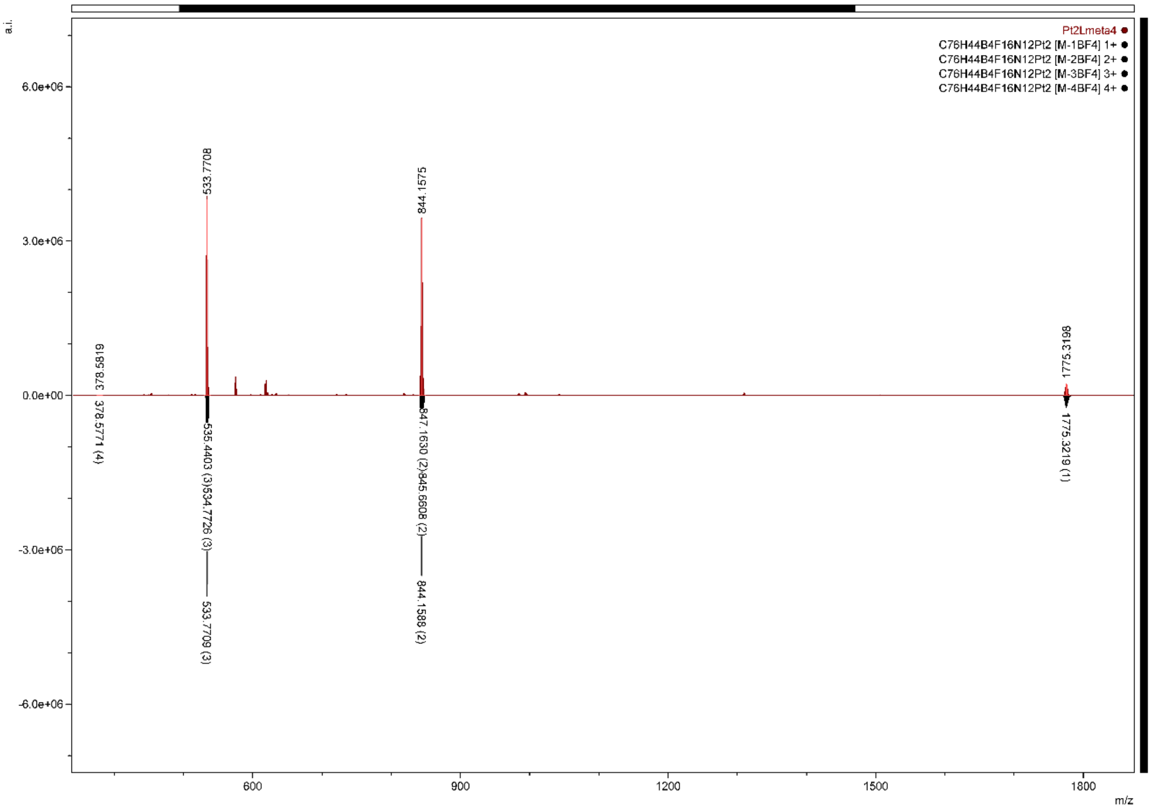

Figure S107. Full ESI-MS spectra of $[Pt_2(\mathbf{L^{meta}})_4]^{4+}(BF_4^-)_4$ prepared at 150°C for 1d. Below the simulated spectra, above obtained spectra.

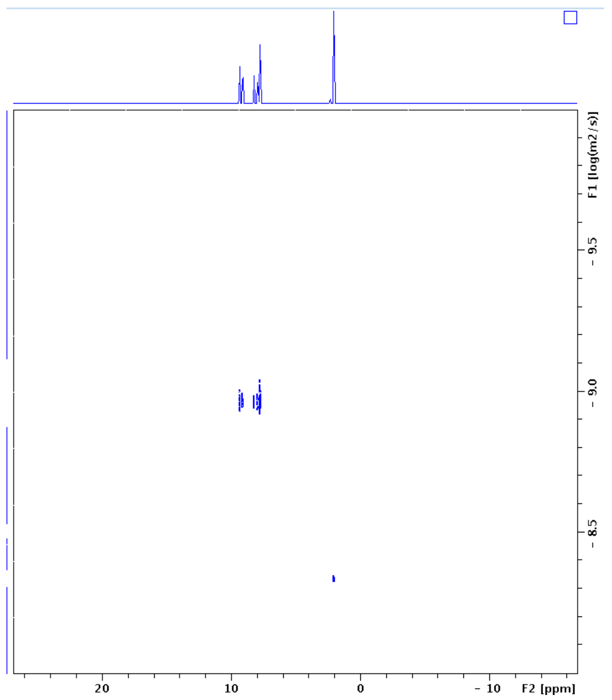

Figure S108. Full DOSY NMR spectra of $[Pt_2(\mathbf{L^{meta}})_4]^{4+}(BF_4^-)_4$ prepared at 150°C for 1d.

$[Pt_3(\mathbf{L^{para}})_6]^{6+}(BF_4^-)_6$ + $[Pt_4(\mathbf{L^{para}})_8]^{8+}(BF_4^-)_8$

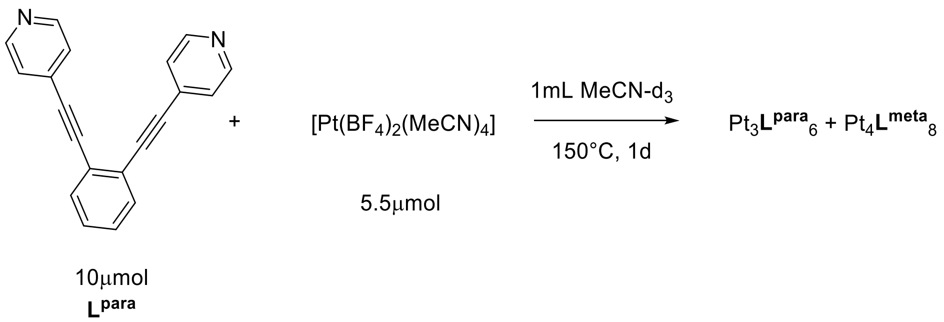

To a solution of $\mathbf{L^{para}}$ (2.80 mg, 10 μmol, 1 eq.) in 0.5 mL MeCN-d_3, $[Pt(BF_4)_2(MeCN)_4]$ (2.93 mg, 5.5 μmol. 0.55 eq.) in 0.5 mL MeCN-d_3 was added. The solution was then stirred at 150°C for 1d in a high pressure tube. ^1H NMR (300 MHz, Acetonitrile-d_3) δ 8.97 (d, J = 6.7 Hz, 6H), 8.86 (d, J = 6.3 Hz, 24H), 7.77 – 7.66 (m, 48H), 7.66 – 7.54 (m, 22H).

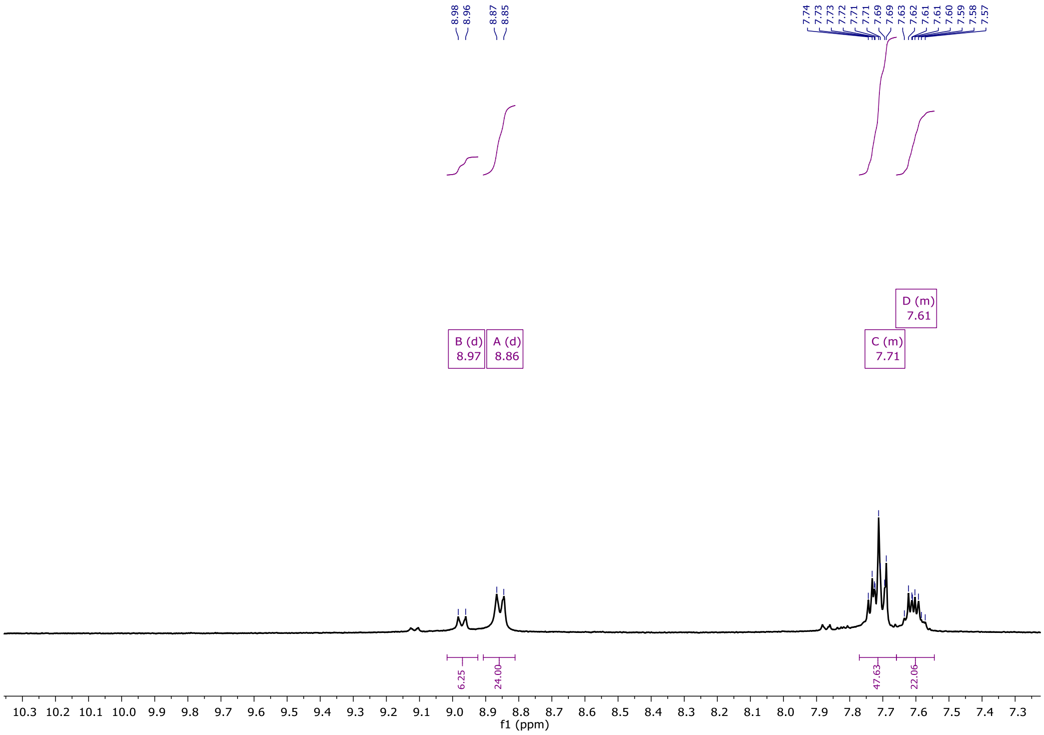

Figure S109. Mixture of $[Pt_3(\mathbf{L}^{para})_6]^{6+}(BF_4^-)_6$ and $[Pt_4(\mathbf{L}^{para})_8]^{8+}(BF_4^-)_8$, ^1H NMR in MeCN-d3.

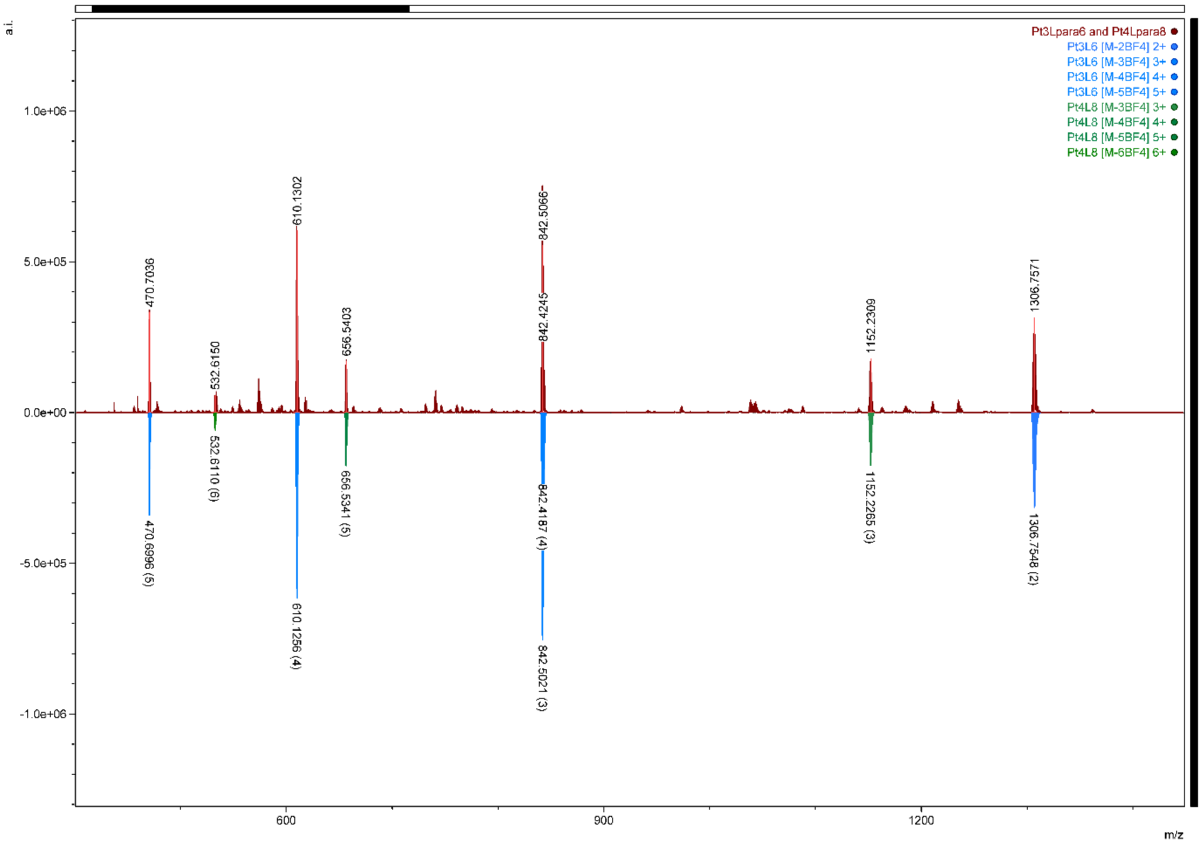

Figure S110. Full ESI-MS spectra of the mix of $[Pt_3(\mathbf{L}^{para})_6]^{6+}(BF_4^-)_6$ and $[Pt_4(\mathbf{L}^{para})_8]^{8+}(BF_4^-)_8$ prepared at 150°C for 1d. Below the simulated spectra, above obtained spectra.

Possible formation pathways (SI6)

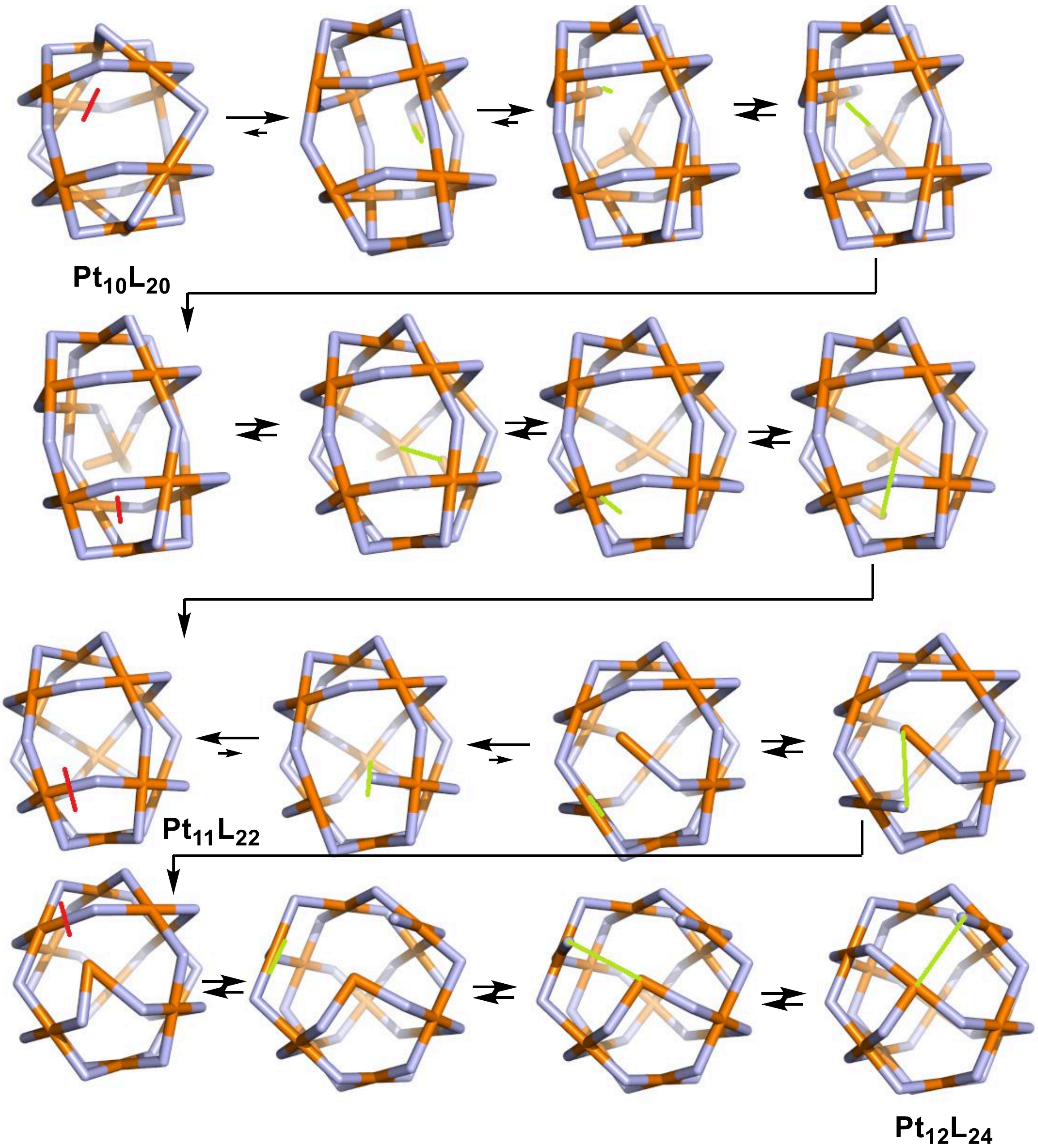

Figure S111. Possible pathway transforming the $Pt_{10}L_{20}$ to the $Pt_{12}L_{24}$ over $Pt_{11}L_{22}$ (Pt=orange, ligand=light blue, red bars indicate where bonds are broken, green bars indicate where bonds are made). This graphic shows only one possible pathway. In this pathway only one bond is broken in the first step, multiple other pathways can be envisioned.

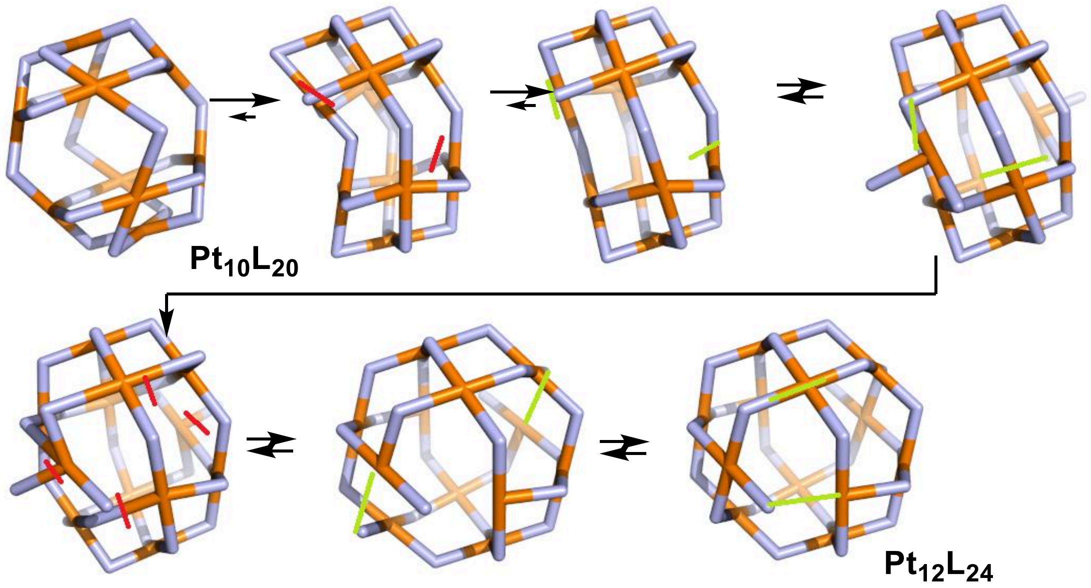

Figure S112. Possible pathway transforming the $Pt_{10}L_{20}$ to the $Pt_{12}L_{24}$ without any other intermediate sphere assembly (Pt=orange, ligand=light blue, red bars indicate where bonds are broken, green bars indicate where bonds are made). This graphic shows only one possible pathway. In this pathway only two bonds have to be broken in the first step, multiple other pathways can be envisioned.

Computational Methods and calculated relative energies for different type of assemblies (SI7)

The relative energies of coordination cage topologies were computed using an extension of previously reported classical-mechanics dynamics model.[8] Amber forcefield parameters were developed for platinum-pyridyl coordination and sp1 carbon bonds by a genetic fitting of DFT single point energies, via *paramfit*,[9] computed at the rB3LYP/def2TZV level of theory, via *Gaussian 16*.[10] Platinum non-bonded interactions were based on previously reported values for divalent platinum ions.[11]

Structures were assembled for coordination cages containing 6–12 metal centers, either palladium or platinum, based on templates using *profit*.[12] These initial structures were optimized with the parameterized forcefield using the single-threaded *sander* program included in the AmberTools16 suite.[13] A molecular dynamics trajectory was then propagated for 5 ns of equilibration and 20 ns of productive molecular dynamics using the GPU-enabled *pmemd.cuda*.[14] Both minimization and molecular dynamics trajectory simulations were carried out with a generalized-Borne model implicit solvation adjusted for the dielectric constant of acetonitrile (ca. 36 F m^{-1}) as shown below in the provided input files, Table S21. The average potential energy of each model, divided by the number of metal centers, was then used to compute the relative free energy for possible M_nL_{2n} topologies formed from either Pd or Pt metal centers. The relative energy for each cage

size was then computed from the Boltzmann weighted contributions of all topological configurations containing the same number of metal centers, e.g. the triangular bicopula or cuboctoheron forms of $M_{12}L_{24}$ cages.

Table S21: Amber input files for single-threaded CPU minimization and minimization.

Minimization	Molecular dynamics
&cntrl<br>imin = 1,<br>ntb = 0,<br>igb = 7,<br>saltcon = 0.1,<br>extdiel = 36,<br>maxcyc = 4000,<br>ioutfm = 1,<br>ncyc = 500,<br>cut = 12,<br>ntwr = 10,<br>ntpr = 10,<br>/	&cntrl<br>imin = 0,<br>ntb = 0,<br>irest = 1, ntx = 7,<br>igb = 7, saltcon = 0.1, extdiel = 36,<br>cut = 9999,<br>ntt = 3, gamma_ln=1.0,<br>tempi = 0.0, temp0 =300.0,<br>nstlim = 5000000, dt = 0.001,<br>ioutfm = 1,<br>ntpr = 1000, ntwx = 1000, ntwr =1000<br>/

Table S22. Summary of relative energies for different type of Pt_nL_{2n} assemblies in kcal/mol.

Size n	**L**^{OMe}; M=Pd	**L**^{OMe}; M=Pt	L=**L**^{Py}; M=Pt	L=**L**^{PO}; M=Pt
6	3.39	4.06	0.61	1.08
8	1.45	2.64	1.12	0.12
9	1.25	1.71	1.42	0.14
10	1.93	2.41	1.42	1.21
11	1.67	1.70	1.64	2.62
12	0.15	0.23	0.38	0

MS calibration of L^{OMe} and L^{Py} and MSMS analysis (SI8)

The calibration of the response of different sized assemblies with L^{OMe} was performed by dilution of a sample of $Pt_nL^{OMe}{}_{2n}$ with $Pd_{12}L^{OMe}{}_{24}$. The intensity of the unique signals corresponding to different types of assemblies *versus* the relative concentration shows a linear response (when the settings on the MS machine are kept the same, same solvent system is used and ion strength of the solution is kept the same). Thus, spherical self-assemblies show a linear correlation between intensity and concentration similar like organic molecules. The same procedure was also applied for L^{Py}.

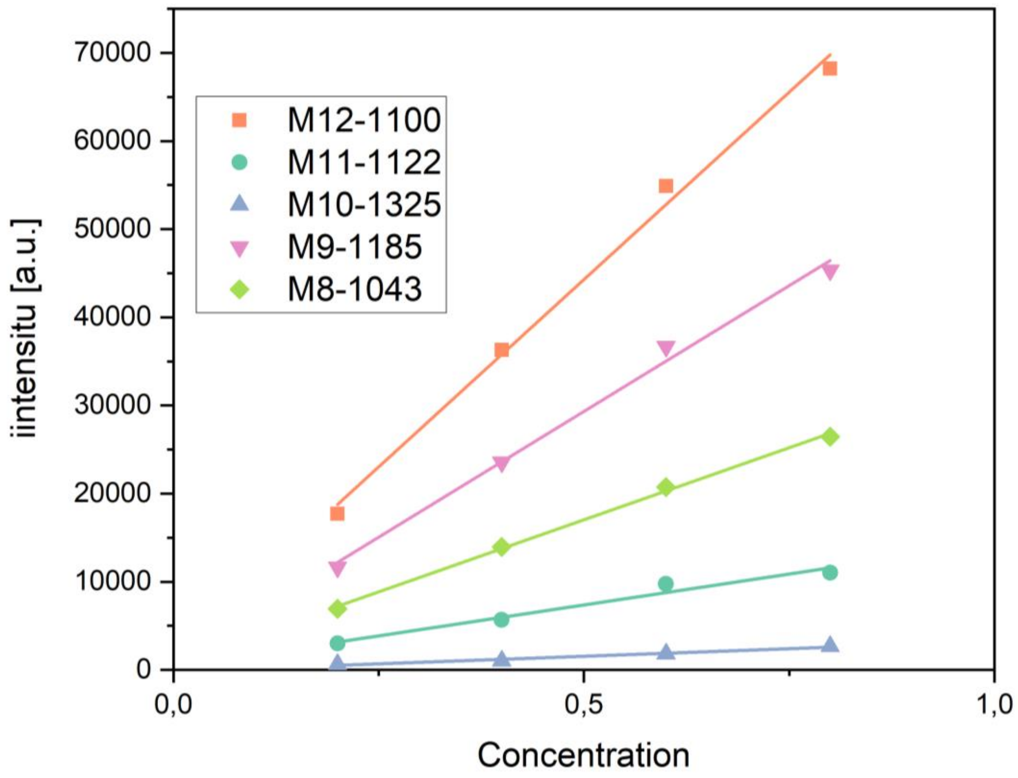

Equation	y = a + b*x				
Plot	M12-1100	M11-1122	M10-1325	M9-1185	M8-1043
Weight	No Weighting				
Intercept	1766,5 ± 2494,	354 ± 1015,74	-166 ± 212,	782,5 ± 1787,0	677,5 ± 579,6
Slope	85015,5 ± 455	14029,5 ± 185	3433 ± 387,	57063,5 ± 326	32697 ± 1058
Residual Sum of	8299468,7	1375652,7	60033,2	4257914,3	448060,2
Pearson's r	0,99714	0,98297	0,9875	0,99675	0,99895
R-Square (COD)	0,99429	0,96623	0,97516	0,9935	0,99791
Adj. R-Square	0,99144	0,94935	0,96275	0,99026	0,99686

Figure S113. Plot of the intensity of different size assemblies with the general structure $Pt_nL^{OMe}{}_{2n}$ *versus* their relative concentration. Pure assemblies could not be obtained, the calibration was performed on a sample prepared at 150°C for 3d.

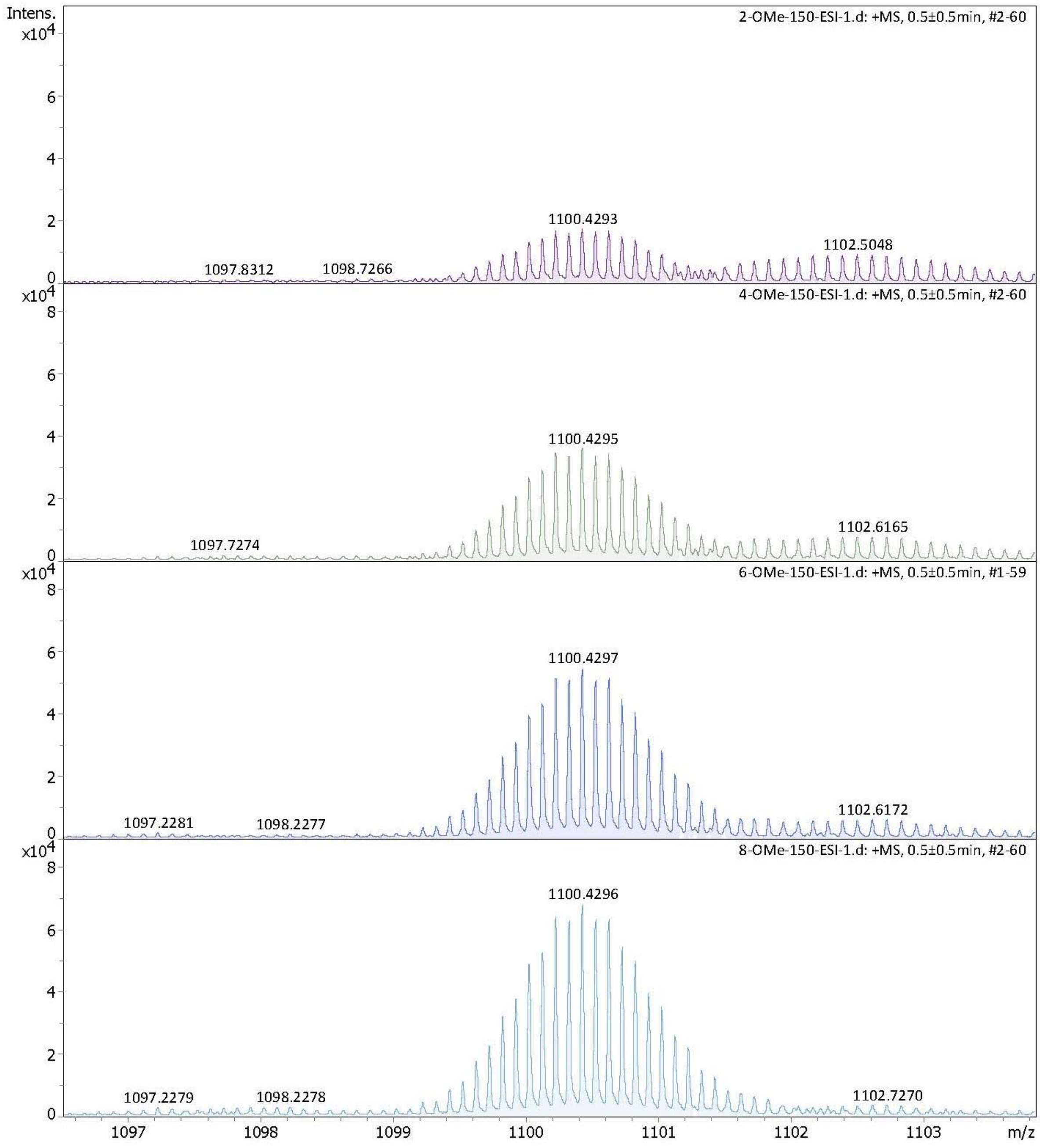

Figure S114. Example of the 10^{+} species of $Pt_{12}\mathbf{L}^{OMe}_{24}$, the intensity decreases linearly upon dilution (from bottom to top, 1.25, 1.66, 2.5 and 5 times diluted).

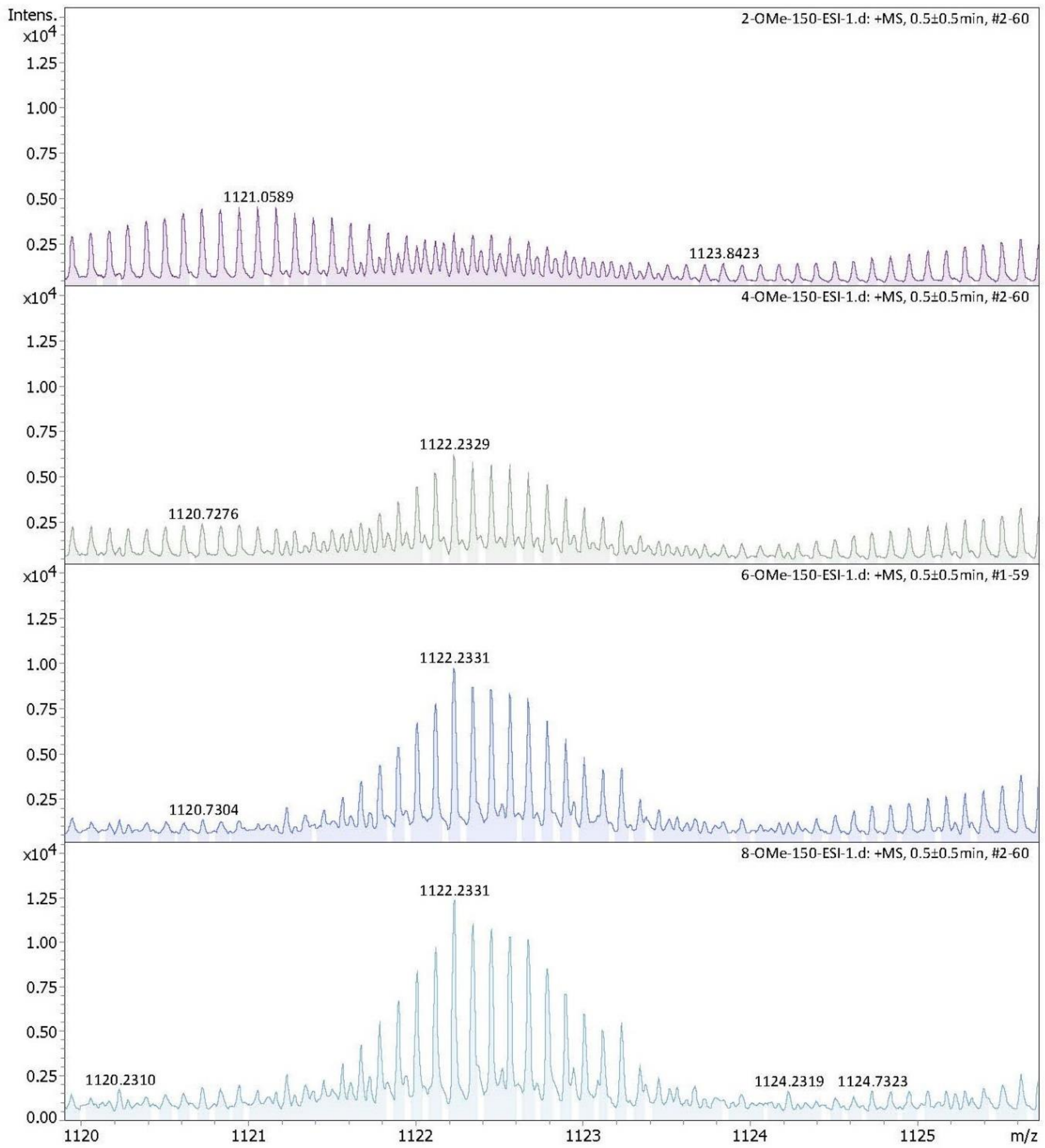

Figure S115. Example of the 8^+ species of Pt_9**L**^{OMe}$_{18}$, the intensity decreases linearly upon dilution (from bottom to top, 1.25, 1.66, 2.5 and 5 times diluted).

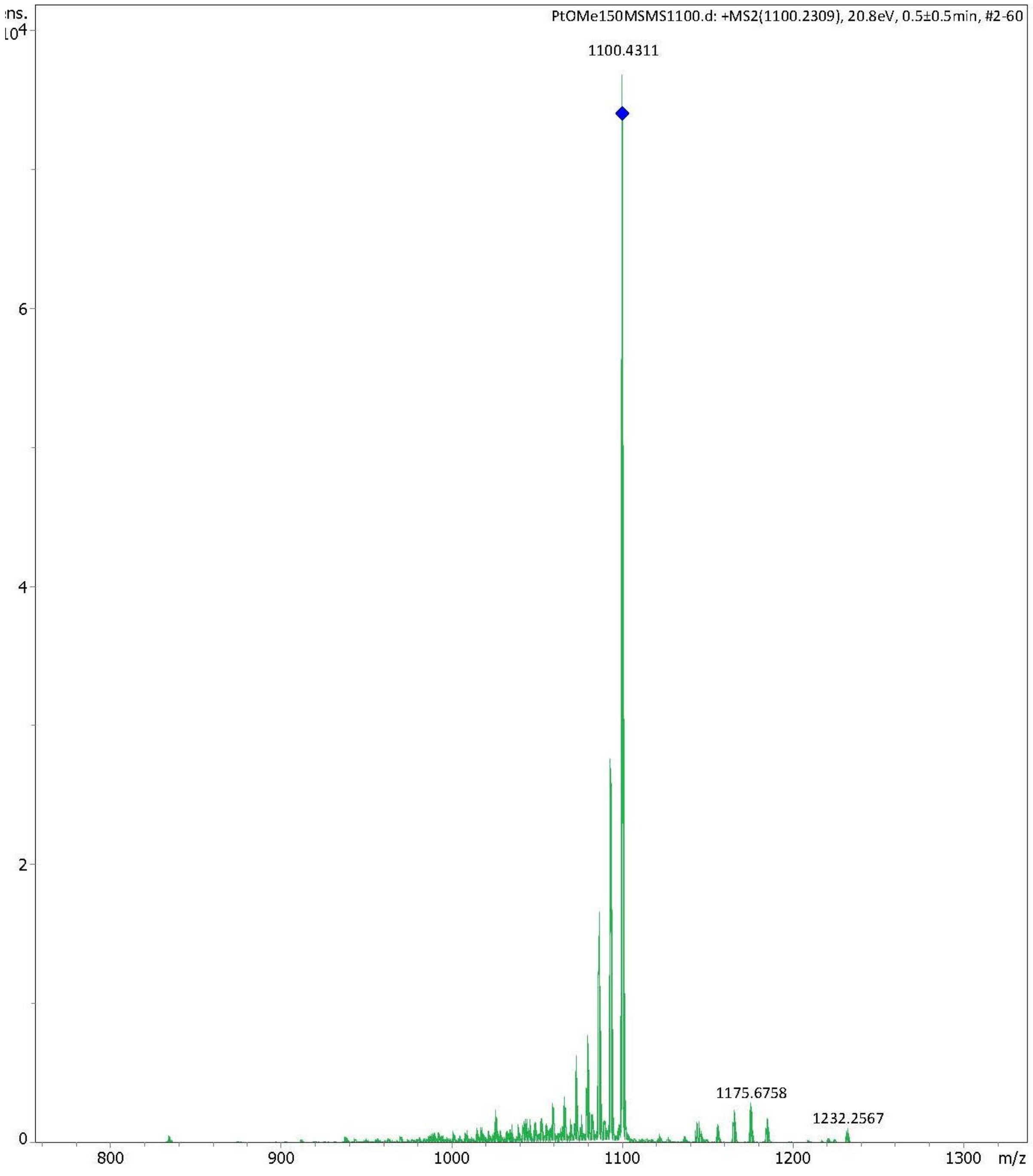

Figure S116. MSMS performed on the 10+ species of $Pt_{12}\mathbf{L}^{\mathbf{OMe}}_{24}$ under high energy input (until 50% of initial signal remain) showing no appearance of other sized assemblies due to MS ionization (measuring conditions 3eV, MSMS 20.8 eV).

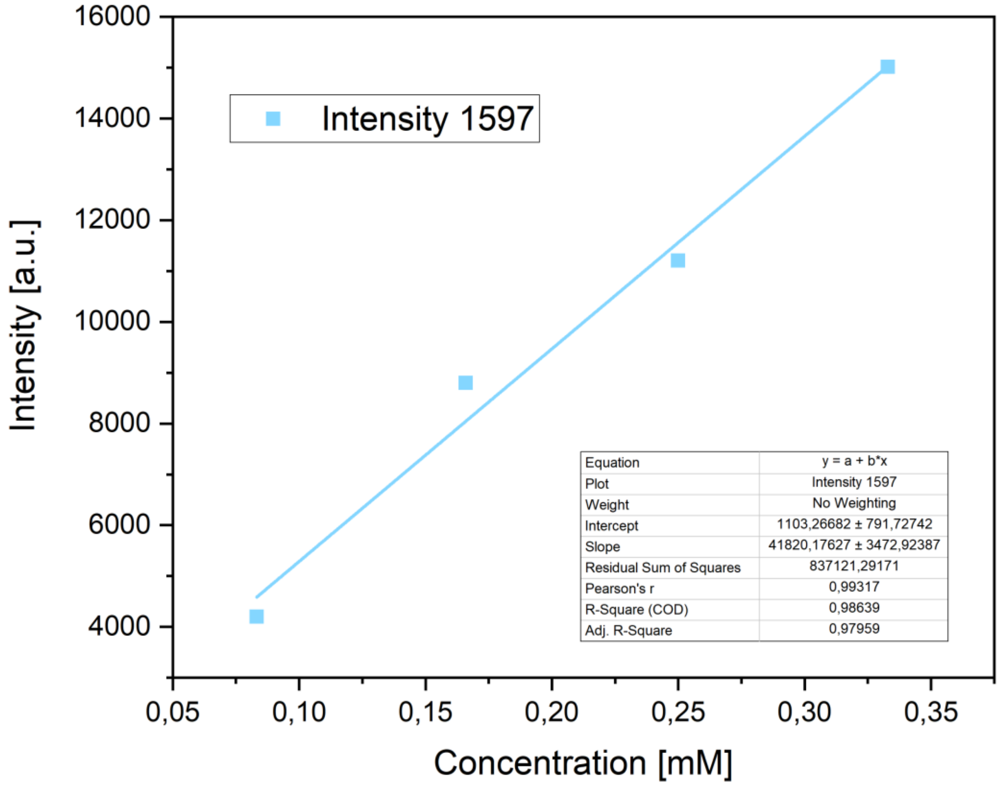

Figure S117. Plot of the intensity of $Pt_{12}$$\mathbf{L}^{\mathbf{Py}}_{24}$ *versus* its concentration.

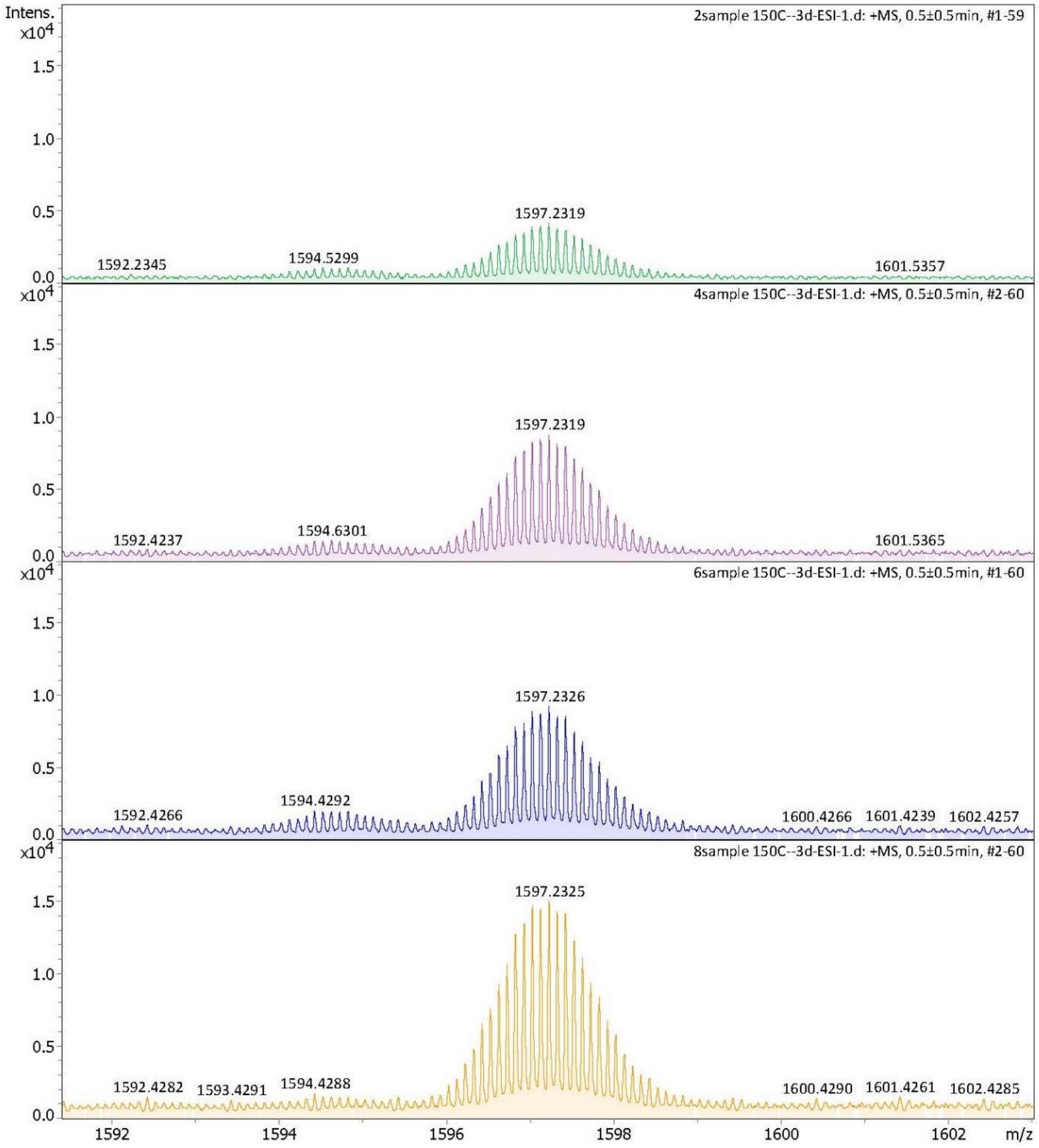

Figure S118. Example of the 10^{+} species of $Pt_{12}\mathbf{L}^{\mathbf{Py}}_{24}$, the intensity decreases linearly upon dilution (from bottom to top, 1.25, 1.66, 2.5 and 5 times diluted).

Colum chromatography on self-assembled $\mathbf{Pt_nL^{OMe}_{2n}}$ (SI9)

10mL 0,41mM solution of $Pt_n\mathbf{L}^{\mathbf{OMe}}_{2n}$ in MeCN was prepared by heating 100 mmol $\mathbf{L}^{\mathbf{OMe}}$ with 55 mmol $[Pt(BF_4)_2(MeCN)_4]$ for 3d at 150°C in a high pressure vial. After the solution was cooled down to room temperature, it was added on 2g of celithe and the solvent was evaporated *in vacuo*. The material was purified *via* column chromatography using methanol as eluent and silica (4 g) as the stationary phase. A yellow-colored fraction was

collected. The solvent was evaporated *in vacuo* and the solid material was dissolved in dmso for MS analysis.

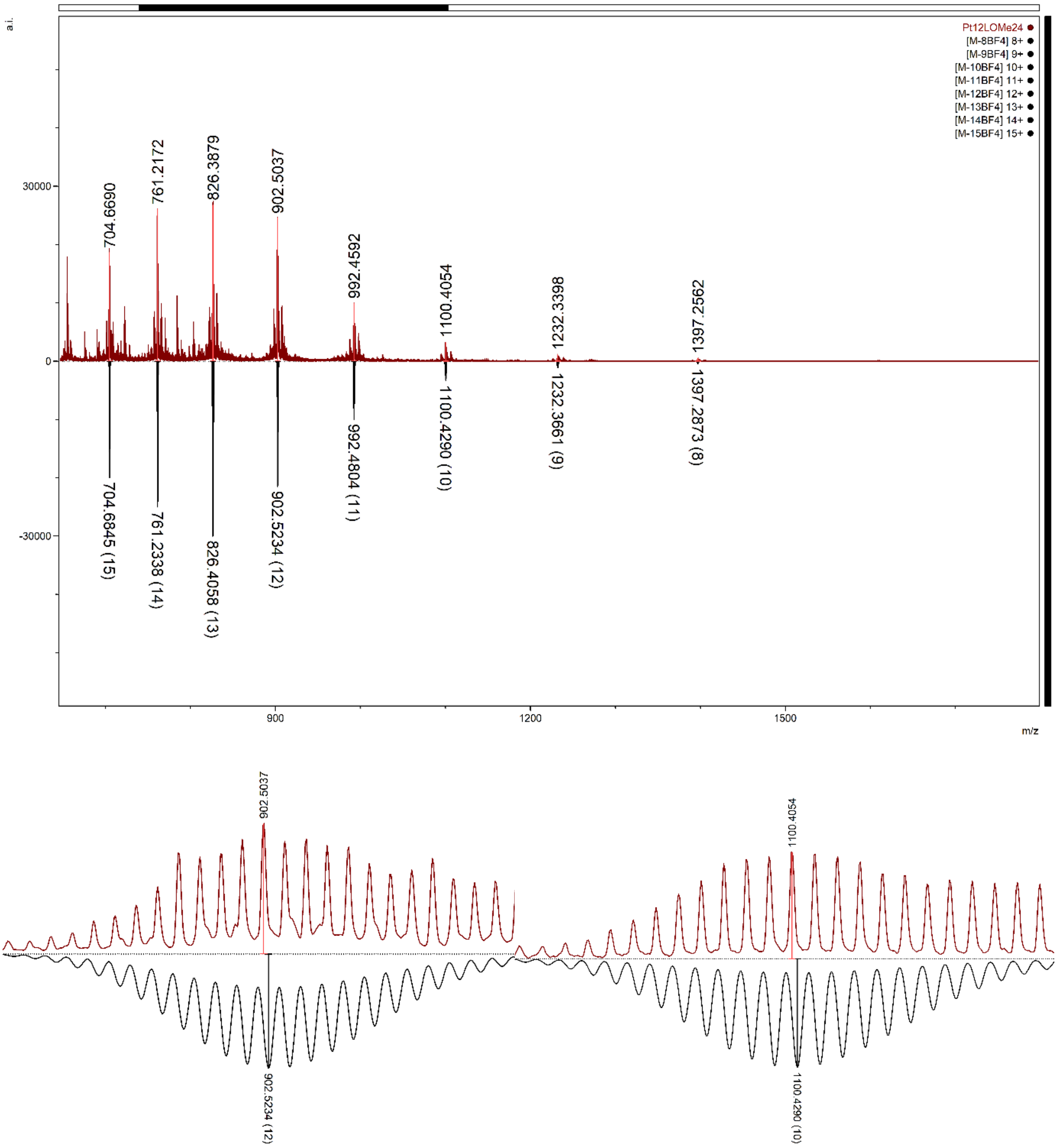

Figure S119. Full spectra of the columed $Pt_nL^{OMe}{}_{2n}$ sample showing only the presence of $Pt_{12}L^{OMe}{}_{24}$. Zoom in into the 10^+ and 12^+ species showing no overlap with other type of assemblies.

Hydrodynamic Radius Considerations (SI10)

Table S23. Summary of calculated hydrodynamic radii for different type of Pt_nL_{2n} assemblies and the expected logD values.

Sphere Size	Calc. Hydrodynamic r [nm]	Calc. Log D
M6L12	1.6	-9.40
M8L16	1.8	-9.45
M9L18	1.9	-9.47
M12L24	2.3	-9.55

Despite the differences in size, the fitting of overlapping, slow diffusing species such as the Pt6L12 and Pt12L24 spheres as two separable components (within instrumental error) remains an experimental limitation of the technique. Because these two most distinguishable assemblies are never in solution in high quantities and other assemblies as well as oligomers are present, only an averaged diffusion coefficient is obtained in all experiments. This averaged diffusion coefficient is located between -9.4 to -9.55 for all formed structures regardless of the applied temperature.

References

[1] Julian M.W. Chan, Timothy M. Swager, *Tet. Lett.*, 49, 33, **2008**, 4912-4914.

[2] D. Fujita, A. Takahashi, M. Fujita, *J. Am. Chem. Soc.* **2011**, 133, 34, 13317–13319.

[3] R. C. Helgeson, B. P. Czech, E. Chapoteau, C. R. Gebauer, A. Kumar, D. J. Cram, *J. Am. Chem. Soc.* **1989**, 111, 16, 6339–6350.

[4] R. Gramage-Doria, J. Hessels, S. H. A. M. Leenders, O. Tröppner, M. Dürr, I. Ivanović-Burmazović, J. N. H. Reek, *Angew. Chem. Int. Ed.,* 126, 49, **2014**, 13598-13602.

[5] J. A. Murphy, F. Schoenebeck, N. J. Findlay, D. W. Thomson, S. Zhou, J. Garnier, *J. Am. Chem. Soc.* **2009**, 131, 18, 6475–6479.

[6] A. M. Johnson, O. Moshe, A. S. Gamboa, B. W. Langloss, J. F. K. Limtiaco, C. K. Larive, R. J. Hooley, *Inorg. Chem.* **2011**, 50, 19, 9430–9442.

[7] K. Suzuki, M. Kawano, M. Fujita, *Angew. Chem. Int. Ed.* 46, 16, **2007**, 2819-2822.

[8] D. Poole, E. O. Bobylev, S. Mathew, J. N. H. Reek, **2020**, *Chem. Sci.*, 11, 12350-12357.

[9] R. M. Betz, R. C. Walker, *J. Comput. Chem.*, **2015**, 36, 2, 79–87.

[10] Gaussian 16, Revision C.01, M. J. Frisch, G. W. Trucks, H. B. Schlegel, G. E. Scuseria, M. A. Robb, J. R. Cheeseman, G. Scalmani, V. Barone, G. A. Petersson, H. Nakatsuji, X. Li, M. Caricato, A. V. Marenich, J. Bloino, B. G. Janesko, R. Gomperts, B. Mennucci, H. P. Hratchian, J. V. Ortiz, A. F. Izmaylov, J. L. Sonnenberg, D. Williams-Young, F. Ding, F. Lipparini, F. Egidi, J. Goings, B. Peng, A. Petrone, T. Henderson, D. Ranasinghe, V. G. Zakrzewski, J. Gao, N.

Rega, G. Zheng, W. Liang, M. Hada, M. Ehara, K. Toyota, R. Fukuda, J. Hasegawa, M. Ishida, T. Nakajima, Y. Honda, O. Kitao, H. Nakai, T. Vreven, K. Throssell, J. A. Montgomery, Jr., J. E. Peralta, F. Ogliaro, M. J. Bearpark, J. J. Heyd, E. N. Brothers, K. N. Kudin, V. N. Staroverov, T. A. Keith, R. Kobayashi, J. Normand, K. Raghavachari, A. P. Rendell, J. C. Burant, S. S. Iyengar, J. Tomasi, M. Cossi, J. M. Millam, M. Klene, C. Adamo, R. Cammi, J. W. Ochterski, R. L. Martin, K. Morokuma, O. Farkas, J. B. Foresman, and D. J. Fox, Gaussian, Inc., Wallingford CT, 2016.

[11] P. Li, B. P. Roberts, D. K. Chakravorty, K. M. Merz, *J. Chem. Theory Comput.,* **2013**, 9, 6, 2733–2748.

[12] A. D. McLachlan, *Acta Crystallogr. Sect. A*, **1982**, 38, 6, 871–873.

[13] D. A. Case, T. E. Cheatham, T. Darden, H. Gohlke, R. Luo, K. M. Merz, A. Onufriev, C. Simmerling, B. Wang, R. J. Woods, *J. Comput. Chem.*, **2005**, 26, 16, 1668–1688.

[14] A. W. Goetz, M. J. Williamson, D. Xu, D. Poole, S. Le Grand, R. C. Walker, *J. Chem. Theory Comput.*, **2012**, 8, 1542-1555.
